# Alternating copolymerization of l-lactide and ε-caprolactone *via* enantiomorphic site and chain-end synergistic control

**DOI:** 10.1039/d6sc04630d

**Published:** 2026-06-22

**Authors:** Ji Xian, Guojie Li, Linyun Wu, Hongjun Fu, Chunmei Wang, Shaoyu Lü, Xiaobo Pan, Jincai Wu

**Affiliations:** a State Key Laboratory of Natural Product Chemistry (Lanzhou University), Key Laboratory of Nonferrous Metal Chemistry and Resources Utilization of Gansu Province, College of Chemistry and Chemical Engineering, Lanzhou University Lanzhou 730000 China wujc@lzu.edu.cn

## Abstract

The alternating copolymerization of chiral and achiral cyclic esters, such as commercial l-lactide (l-LA) and ε-caprolactone (CL), remains a significant challenge in polymer synthesis. Here, we introduce an enantiomorphic site and chain-end synergistic control strategy to achieve the first highly alternating copolymerization of l-LA and CL. The polymerization proceeds *via* a dynamic mechanism: first, the chiral l-LA-terminated chain end and the enantiomorphic site of the catalyst synergistically preclude the homopropagation of highly reactive l-LA; subsequently, the rate-determining insertion of the less reactive achiral CL after the l-LA-terminated chain end, generating an achiral CL-terminated chain end, relieves steric hindrance, which allows the subsequent insertion of highly reactive l-LA. This dynamic control yields highly alternating poly(l-LA-*alt*-CL) (*P*_alt_ up to 0.91). The highly alternating copolymerization of l-ethylglycolide (l-EG) and CL (*P*_alt_ = 0.96) further validates the universality of this synergistic control for constructing alternating cyclic ester copolymers.

## Abstract 2

Highly alternating copolymerization of l-lactide and ε-caprolactone is achieved *via* an enantiomorphic site and chain-end synergistic control strategy. This strategy yields sequence-controlled polyesters with exceptional regularity (*P*_alt_ > 0.90).

## Graphical abstract

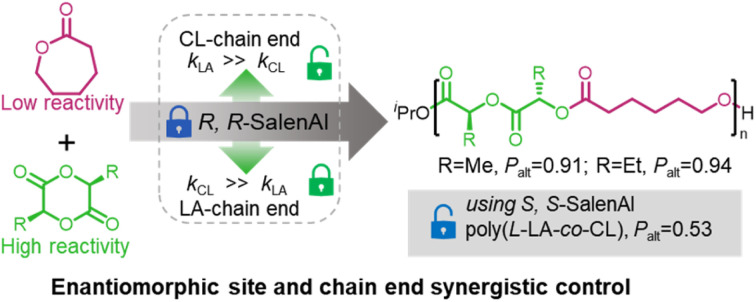

## Introduction

Alternating copolymerization of commercially available monomers is a powerful and economical method to afford new types of polymers, exhibiting potentially distinct physical and chemical properties compared to their homopolymers, block, gradient, and random copolymers.^1–4^ However, the alternating polymerization of different cyclic esters for synthesizing biodegradable polymers has not been explored widely,^5–11^ especially the alternating copolymerization of commercial l-lactide and ε-caprolactone (CL) remains a challenge. Regioselective ring-opening polymerization (ROP) of asymmetric cyclic esters is a feasible way to yield alternating polyesters of different hydroxyl acids,^11–16^ and the segmer growth method is a more precise way to synthesize alternating or repeating-sequence polyesters;^17–20^ nevertheless, direct copolymerization of different commercial monomers can avoid the cost of asymmetric monomer synthesis or predesigned sequence-defined monomers or oligomeric segment synthesis. Until now, highly alternating direct copolymerization of different cyclic esters is just limited to two monomers with opposite chiral configurations *via* heterospecific/syndiospecific ROP systems.^5–11^ Thus, the methodology for alternating copolymerization of chiral and achiral cyclic esters, like commercial l-LA and CL, requires to be further developed.

**Scheme 1:**
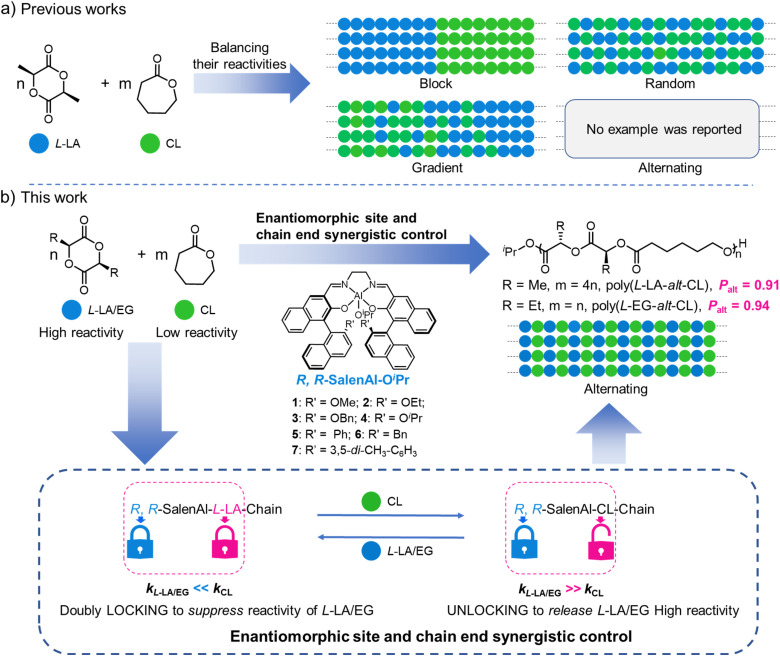
(a) Previous studies on the copolymerization of l-LA and CL have only yielded block, gradient, or random structures, while an alternating copolymer has remained a significant challenge. (b) Enantiomorphic site and chain end synergistic control for the alternating copolymerization of chiral cyclic diesters and CL.

In fact, due to the biodegradability and biocompatibility of polylactide (PLA) and polycaprolactone (PCL) and their important applications,^21–27^ much effort was made to control the monomer sequence in the copolymerization of l-LA and CL. Normally, gradient or block copolymers of l-LA and CL can be obtained because of the significantly higher reactivity of l-LA compared to CL in most copolymerization systems (Scheme 1a).^28–35^ To balance their copolymerization reactivities, stereochemically “mismatched”^36^ or sterically hindered^37^ catalysts were reported to suppress l-LA reactivity, and random copolymers could be effectively produced. But these approaches failed to obtain an alternating sequence-controlled copolymer. Notably, the transesterification strategy cannot yield alternating copolymers, as sequence scrambling invariably produces mixed lactidyl (two lactic acid units) and lactyl (one lactic acid unit) segments.^38^

Thus, previous studies illustrated that simply balancing monomer reactivities is insufficient for the alternating copolymerization of l-LA and CL; the real challenge lies in the improvement of the cross-propagation rate. Given the high reactivity of l-LA, its insertion to a CL-terminated chain end is kinetically facile. The key challenge, therefore, is the selectivity-determining step: the insertion of the less reactive CL into an l-LA-terminated chain end. To achieve strict alternation, the homopolymerization of highly reactive l-LA must be strictly inhibited to facilitate the insertion of CL. To address this requirement beyond simple reactivity modulation, an enantiomorphic site and chain end synergistic control strategy was designed in this work (Scheme 1b). We hypothesized that the chiral catalyst could synergize with the propagating chain end to dynamically modulate the insertion of the highly reactive l-LA. Specifically, when the chain end is chiral (l-LA-terminated), synergistic steric repulsion from both the catalyst's enantiomorphic site and the chain end selectively precludes the insertion of another l-LA monomer, for the aim of effectively shutting down l-LA homopropagation. Polymerization then proceeds only *via* the insertion of the achiral CL monomer. Consequently, the achiral CL-terminated chain end intermediate with less steric and chiral barrier restores the high activity for the next rapid l-LA insertion. Thus, the chain end and enantiomorphic site may be able to effectively switch the intermediate activity to enforce a strictly alternating sequence.

To realize this strategy, in this work, some *R*,*R*-SalenAl catalysts were utilized and highly alternating copolymers of l-LA and CL (Scheme 1b) with a remarkable alternating probability (*P*_alt_) of up to 0.91 were achieved (1 : 4 l-LA : CL feed ratio, bulk, 70 °C). The universality of this method was further verified by the alternating copolymerization of l-ethylglycolide (l-EG) and CL, achieving a high *P*_alt_ of 0.94 (1 : 1 feed ratio, toluene, 70 °C). To our knowledge, this represents the first successful synthesis of highly alternating l-LA and CL copolymers. Meanwhile, it validates enantiomorphic site and chain end synergistic control as a general mechanism for the alternating-sequence-controlled ROP of chiral and achiral cyclic esters.

## Results and discussion

### Alternating copolymerization of l-lactide with CL

At the outset, the copolymerization of an equimolar mixture of l-LA and CL was conducted, employing a phenyl-substituted *R*,*R*-SalenAl catalyst of complex *R*,*R*-5 (Scheme 1b).^9^ After 133 hours in toluene at 70 °C ([l-LA]_0_ = [CL]_0_ = 2.5 M; [l-LA]_0_ : [CL]_0_ : [Cat.]_0_ = 50 : 50 : 1), 55% l-LA and 42% CL were converted into a polymer (Table S1, Entry 1). Analysis of the resulting copolymer's microstructure revealed a strong bias towards alternating sequence, with calculated cross-propagation probabilities of *P*_CL-LA_ = 0.95 and, more critically, *P*_LA-CL_ = 0.68, where *P*_CL-LA_ represents the probability of a CL-chain end attacking an LA monomer and *P*_LA-CL_ represents the probability of an LA-chain end attacking a CL monomer. The probability *P*_CL-LA_ was calculated from the relative integrals of the ε-methylene protons (–CH_2_–O–) of the CL unit in the ^1^H NMR spectrum (Fig. S1), which are sensitive to the subsequent monomer (4.08–4.19 ppm for a CL-LA dyad *vs.* 4.03–4.08 ppm for a CL–CL dyad). The crucial probability *P*_LA-CL_ was calculated from the ratio of the integrals of two key signals: the α-methylene protons of CL units in LA-CL dyads (2.33–2.47 ppm) and the methine protons of all l-LA units (5.06–5.23 ppm). Since the signal from the α-CH_2_ group of the LA-CL interunit linkage and the signal from the l-LA unit's two methine protons both represent two protons (Fig. S1), the probability can be directly given by the ratio *P*_LA-CL_ = *I*_LA-CL_/*I*_LA, all_.

Employing an *R*,*R*-SalenAl catalyst with less hindered benzyl substituents, complex *R*,*R*-6 (Table S1, Entry 2), led to a significant increase in activity (94% l-LA, 74% CL conversion in 54 h), while sequence selectivity remained high (*P*_LA-CL_ = 0.70, *P*_CL-LA_ = 0.93). Conversely, introducing bulkier 3,5-dimethylphenyl groups in the *R*,*R*-SalenAl catalyst (*R*,*R*-7) was detrimental to both activity and selectivity. The polymerization became exceptionally sluggish (279 h), and the crucial probability of cross-propagation from the l-LA-terminated chain end dropped sharply to *P*_LA-CL_ = 0.30, even though *P*_CL-LA_ remained high at 0.92 (Table S1, Entry 3). The initial screening indicated that the less hindered benzyl-substituted catalyst provided a superior balance of activity and selectivity. This prompted us to synthesize a new series of *R*,*R*-SalenAl complexes bearing smaller alkoxy substituents on the Salen ligand (Scheme 1b): methoxy (1), ethoxy (2), benzyloxy (3), and isopropoxy (4), all of which exhibit high heteroselectivity for *rac*-LA (*P*_r_ > 0.90, Table S2). When these catalysts were applied to the l-LA and CL copolymerization (Table 1, Entries 1–4), all four catalysts exhibited a remarkable improvement in catalytic activity, reaching high conversions within 25 hours. Furthermore, this enhanced activity was accompanied by a slight improvement in alternating selectivity. Among them, complexes *R*,*R*-2 and *R*,*R*-3 were the most effective, affording the highest alternating probabilities with *P*_LA-CL_/*P*_CL-LA_ values of 0.76/0.93 and 0.75/0.96, respectively. This demonstrated that tuning the Salen ligand with smaller, electron-donating alkoxyl groups is a highly effective strategy for achieving both high activity and excellent sequence control. However, further enhancing the alternating selectivity solely through ligand modification seems to be difficult. A critical analysis of our results revealed that across all catalysts, the conversion of l-LA was consistently higher than that of CL, indicating that the intrinsic reactivity disparity between the two monomers is the primary obstacle to achieving perfect alternating selectivity. To overcome this fundamental challenge, *i.e.*, to facilitate the insertion of CL after l-LA insertion, increasing the concentration of CL in the feed may be helpful to kinetically compensate for CL's lower reactivity.

**Table 1:** Results for the alternating copolymerization of chiral cyclic diesters with CL^a^

Entry	Monomer	Catalyst	[CL]_0_/[*M*]_0_	Time (h)	Conv.^b^ (%) M, CL	*M*_*n*,obsd_ c (kg mol^−1^)	*Đ* c	*P*_M-CL_ d	*P*_CL-M_ d
1	l-LA	*R*,*R*-1	1	18	95, 65	18.2	1.08	0.63	0.92
2	l-LA	*R*,*R*-2	1	13	86, 70	15.3	1.10	0.76	0.93
3	l-LA	*R*,*R*-3	1	14	90, 70	19.7	1.07	0.75	0.96
4	l-LA	*R*,*R*-4	1	25	84, 65	15.6	1.07	0.73	0.94
5^e^	l-LA	*R*,*R*-1	4	48	68, 17	28.8	1.04	0.81	0.90
6^e^	l-LA	*R*,*R*-2	4	41	87, 23	44.8	1.06	0.92	0.90
7^e^	l-LA	*R*,*R*-3	4	64	92, 25	46.0	1.06	0.92	0.89
8^e^	l-LA	*R*,*R*-4	4	94	68, 17	31.7	1.05	0.88	0.90
9	l-EG	*R*,*R*-1	1	49	81, 80	18.5	1.06	0.90	0.91
10	l-EG	*R*,*R*-2	1	28	78, 72	17.6	1.06	0.87	0.91
11	l-EG	*R*,*R*-3	1	24	80, 78	19.4	1.05	0.91	0.92
12	l-EG	*R*,*R*-4	1	48	88, 90	22.3	1.06	0.96	0.96
13	l-EG	*S*,*S*-4	1	114	81, 55	15.9	1.08	0.44	0.66

a General conditions for solution polymerization: [*M*]_0_ : [CL]_0_ : [Cat.]_0_ = 50 : 50 : 1; performed in toluene with [*M*]_0_ = 2.5 M and [CL]_0_ = 2.5 M, under a dry argon atmosphere at 70 °C.

b Monomer conversion was determined by integrating selected monomer and polymer signals in the ^1^H NMR spectrum.

c Experimental *M*_*n*_ and *Đ* were determined by gel permeation chromatography (GPC) in THF calibrated with standard polystyrene samples.

d The sequence probabilities *P*_M-CL_ and *P*_CL-M_ were determined by ^1^H NMR analysis for l-LA/CL copolymers and by ^13^C NMR analysis for l-EG/CL copolymers.

e Carried out *via* bulk polymerization with a molar ratio of [LA]_0_ : [CL]_0_ : [Cat.]_0_ = 100 : 400 : 1 at 70 °C.

Therefore, copolymerizations were conducted at 70 °C using a [l-LA]_0_ : [CL]_0_ : [Cat.]_0_ feed ratio of 100 : 400 : 1. With this high ratio [CL]_0_/[l-LA]_0_ of 4, the large excess of the liquid CL monomer enabled the polymerization to be performed under solvent-free, bulk conditions, representing a more industrially relevant process. Gratifyingly, employing a high excess of CL in bulk polymerization proved to be highly effective in boosting the alternating selectivity. The four alkoxyl group-substituted catalysts (*R*,*R*-1 to *R*,*R*-4) were evaluated under these conditions (Table 1, Entries 5–8). All polymerizations were well-controlled, affording high molecular weight copolymers (up to 46.0 kg mol^−1^) with narrow dispersities (*Đ* = 1.04–1.06), confirming the good controllability of the catalysis even under bulk polymerization conditions. *R*,*R*-2 emerged as the top-performing system. Most strikingly, it dramatically enhanced the selectivity of the most challenging step: the addition of CL to an l-LA-terminated chain end, affording a copolymer with an outstanding *P*_LA-CL_ value of 0.92. This represents a significant leap from the previous best of ∼0.76 achieved in solution, while *P*_CL-LA_ remained high at 0.90. This dramatic improvement can be attributed to the high concentration of CL, which kinetically favors the desired cross-propagation pathway (l-LA-end + CL) and effectively outcompetes the undesired l-LA homopropagation (l-LA-end + l-LA). This result, achieving an average alternating probability (*P*_alt_ = *f*_LA_*P*_LA-CL_ + *f*_CL_*P*_CL-LA_) of up to 0.91, where *f*_LA_ and *f*_CL_ are the molar fractions of LA and CL units in the copolymer, respectively, marks a breakthrough. It not only validates our strategy of overcoming the intrinsic monomer reactivity disparity through kinetic control but also represents, to our knowledge, the first successful synthesis of a highly alternating copolymer of l-LA and CL.

**Fig. 1:**
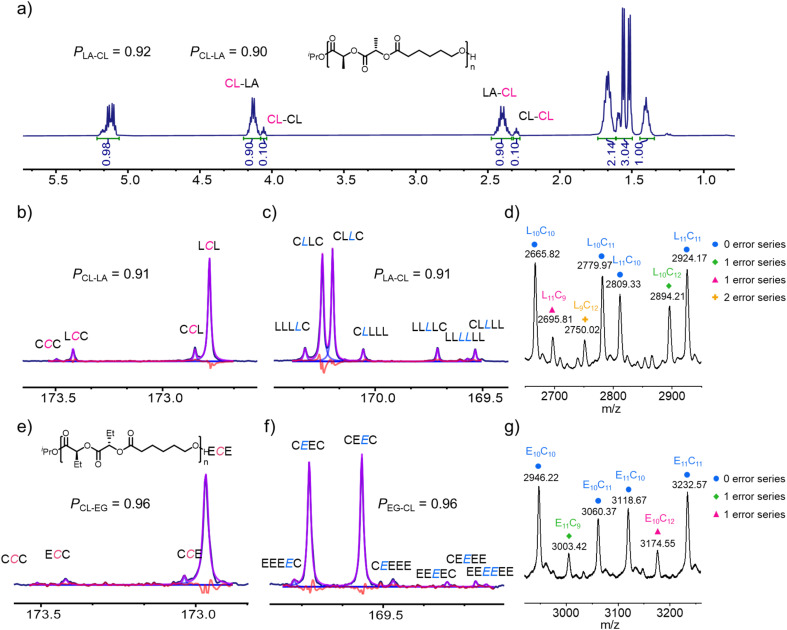
Structural characterization of the highly alternating copolymers. (a) The ^1^H NMR spectrum of poly(l-LA-*alt*-CL) (Table 1, Entry 6). (b) The CL carbonyl region of the ^13^C NMR spectrum of poly(l-LA-*alt*-CL) (Table 1, Entry 6). (c) The LA carbonyl region of the ^13^C NMR spectrum of poly(l-LA-*alt*-CL) (Table 1, Entry 6). (d) MALDI-TOF mass spectrum of a low molecular weight poly(l-LA-*alt*-CL) sample. (e) The CL carbonyl region of the ^13^C NMR spectrum of poly(l-EG-*alt*-CL) (Table 1, Entry 12). (f) The EG carbonyl region of the ^13^C NMR spectrum of poly(l-EG-*alt*-CL) (Table 1, Entry 12). (g) MALDI-TOF mass spectrum of a low molecular weight poly(l-EG-*alt*-CL) sample.

The ^1^H NMR spectrum (Fig. 1a) provided initial, strong evidence for a highly alternating sequence. In the ε-methylene region of the CL units (4.03–4.19 ppm), the signal corresponding to the alternating Cl-LA dyad accounted for over 90% of the total integral, indicating a high *P*_CL-LA_. Furthermore, the methyl region of the l-LA units (1.50–1.61 ppm) displayed two sharp, well-defined doublets, a stark contrast to the complex multiplets typically observed in random PLA.^36,37^ This highly regular pattern strongly suggests a uniform stereochemical and sequential environment consistent with a repeating LA-CL sequence. Translating these high cross-propagation probabilities into average sequence lengths yields values of approximately 1.09 for l-LA and 1.11 for CL (Fig. S2).^39^ These values are very close to the theoretical minimum sequence length of one and provide compelling support for the predominantly alternating structure. Quantitative analysis from the ^13^C NMR spectrum furnished definitive proof. In the CL carbonyl region (Fig. 1b), four distinct peaks were observed, which correspond to CCC, LCC, CCL, and LCL triads (where C = caprolactone and L = lactic acid) according to literature assignments.^40^ The very high relative intensity of the LCL triad suggested a very high alternating probability, and the analysis of the relative peak intensities yielded a *P*_CL-LA_ value of 0.91 (Fig. S5). Remarkably, an independent analysis of the more complex carbonyl carbon signal of the l-LA unit (Fig. 1c), which is sensitive to its neighboring monomer units, yielded a *P*_LA-CL_ value of 0.91 (Fig. S5), in agreement with the ^1^H NMR-derived sequence analysis.

The most direct evidence for the alternating sequence was obtained from MALDI-TOF-MS analysis of a low molecular weight sample (Fig. 1d). The spectrum was dominated by two primary series of peaks, reflecting the two possible initiation events from the non-chiral isopropoxide initiator. One major series corresponds to polymer chains initiated *via* the first ring-opening of a CL monomer. This series itself was composed of two sub-series: one with an even number of total monomer units, described by the general formula ^*i*^PrOH + *n*LA + *n*CL + Na^+^, and another with an odd number of monomer units terminated by one CL, with the formula ^*i*^PrOH + *n*LA + (*n*+1) CL + Na^+^. The second major series corresponds to chains initiated *via* the first ring-opening of an LA monomer. Similarly, this series also contains chains with an even number of units (^*i*^PrOH + *n*LA + *n*CL + Na^+^) and chains with an odd number of units terminated by LA, having the formula ^*i*^PrOH + (*n*+ 1) LA + *n*CL + Na^+^. The clear dominance of these two highly regular, alternating series confirms the fidelity of the main alternating propagation pathway. The minor peaks observed in the spectrum correspond to chains containing a single LA-LA homopropagation error (LA-LA) or a single CL-CL error (CL-CL). Although the polymer chain of L_9_C_12_ (9 l-LA units + 12 CL units, Fig. 1d) including one error CL-CL-CL sequence or two CL-CL error sequences exists, the low intensity of this peak suggests that the probability of CL-CL linkage is low. Therefore, the MALDI-TOF-MS analysis also confirmed that the dominant polymer chain structure is indeed highly alternating.

Additional control polymerizations were performed to further examine the stereochemical origin of the high alternating selectivity in the l-LA/CL system (Fig. S12 and Table S4). l-LA homopolymerization with *R*,*R*-2 was approximately 8.1 times slower than that with *S*,*S*-2 (*k*_app_ = 0.0152 h^−1^*vs.* 0.123 h^−1^). Under comparable conditions, *rac*-LA polymerization with *R*,*R*-2 proceeded more rapidly than l-LA polymerization with either *R*,*R*-2 or *S*,*S*-2. These results suggest that the slow l-LA homopropagation in the *R*,*R*-2 system arises from the synergistic effect of the catalyst enantiomorphic site and the l-LA-terminated chain end. Consistently, *rac*-LA/CL copolymerization with *R*,*R*-2 gave an LA-rich copolymer, in which the molar ratio of LA units to CL units reached 2.22 : 1 as determined by ^1^H NMR analysis (Fig. S13). This result supports that the high alternating selectivity in the l-LA/CL system is closely related to the combined effects of the catalyst enantiomorphic site and the propagating chain end.

### Alternating copolymerization of l-ethylglycolide with CL

As mentioned above, when the l-LA : CL ratio is 1 : 1, the consecutive insertion of l-LA into the polymer chain using this catalytic system is difficult to suppress due to the higher reactivity of l-LA than CL. Thus, to further confirm the universality of this enantiomorphic site and chain-end synergistic control strategy for the alternating copolymerization of the achiral cyclic ester CL with a chiral cyclic diester, the copolymerization of CL with l-EG was tested further, and l-EG is a less reactive analogue of l-LA. Employing the series of *R*,*R*-alkoxyl-substituted catalysts with an equimolar feed of l-EG and CL in toluene ([l-EG]_0_ = [CL]_0_ = 2.5 M; [l-EG]_0_ : [CL]_0_ : [Cat.]_0_ = 50 : 50 : 1), the copolymerizations proceeded efficiently (Table 1, Entries 9–12). Remarkably, *R*,*R*-4 proved to be exceptionally effective, yielding a highly alternating sequence with excellent probability values of *P*_EG-CL_ = 0.96 and *P*_CL-EG_ = 0.96. These probabilities were determined by ^13^C NMR analysis (Fig. 1e and f).

This highly alternating microstructure was further corroborated by MALDI-TOF-MS analysis of a low molecular weight poly(l-EG-*alt*-CL) sample (Fig. 1g). The spectrum provides compelling evidence that poly(l-EG-*alt*-CL) exhibits a more regular alternating sequence than poly(l-LA-*alt*-CL) when the feed ratio of CL to cyclic diester is 1 : 1. Similar to the l-LA/CL system, the spectrum is dominated by two primary series of peaks, arising from the two possible initiation events of the isopropoxide initiator. The first series comprises chains initiated *via* the first ring-opening of a CL monomer (leading to compositions of ^*i*^PrOH + *n*EG + *n*CL + Na^+^ and ^*i*^PrOH + *n*EG + (*n*+ 1) CL + Na^+^). The second major series comprises chains initiated *via* the first ring-opening of an l-EG monomer (leading to compositions of ^*i*^PrOH + *n*EG + *n*CL + Na^+^ and ^*i*^PrOH + (*n* + 1) EG + *n*CL + Na^+^). The dominance of these signals confirms the high fidelity of the alternating propagation. Signals corresponding to isolated, single homopropagation errors of an EG-EG linkage or a CL-CL linkage are much weaker. Significantly, consecutive error sequences like CL-CL-CL, which were faintly detectable for the l-LA/CL copolymer, were significantly attenuated in this case.

This represents a significant improvement even over the above optimized l-LA/CL system and corresponds to an overall alternating probability (*P*_alt_) of 0.96. We attribute this exceptional level of control to the fact that the lower intrinsic reactivity of l-EG relative to l-LA further narrows the reactivity disparity with CL. To validate the critical role of the enantiomorphic site in this control mechanism, a control experiment was performed using the enantiomeric *S*,*S*-4 catalyst to copolymerize l-EG and CL (Table 1, Entry 13). Under these conditions, the alternating selectivities decreased to *P*_EG-CL_ = 0.44 and *P*_CL-EG_ = 0.66. This dramatic drop in selectivity clearly demonstrates that the specific *R*,*R*-configuration of the catalyst is essential for suppressing l-EG homopropagation. Consistent with our previous findings, this result demonstrates that the enantiomorphic site plays a pivotal role in balancing the reactivity difference between the two monomers, which is an essential condition for achieving high alternating probability.

### Mechanism of enantiomorphic site and chain-end synergistic control

**Fig. 2:**
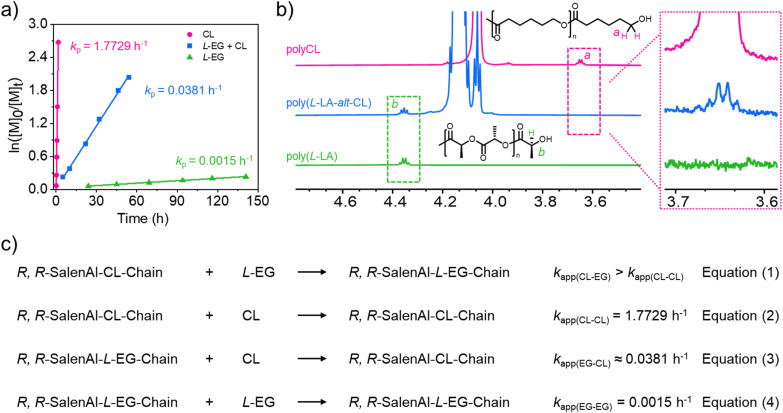
Kinetic and mechanistic studies of the alternating copolymerization. (a) First-order kinetic plots for the polymerizations catalyzed by *R*,*R*-4. The plots correspond to the homopolymerization of CL (●; *k*_app(CL-CL)_ = 1.7729 h^−1^, *R*^2^ = 0.999), the homopolymerization of l-EG (▲; *k*_app(EG-EG)_ = 0.0015 h^−1^, *R*^2^ = 0.998), and their equimolar copolymerization (■; *k*_app(EG/CL)_ = 0.0381 h^−1^, *R*^2^ = 0.998). Conditions: [*M*]_0_ = 1.0 M, [Cat.]_0_ = 0.02 M, toluene, 70 °C. (b) An expansion of the ^1^H NMR spectrum showing the chain-end region of a poly(l-LA-*alt*-CL) sample (at 78% conversion of l-LA, [l-LA]_0_ : [CL]_0_ : [Cat.]_0_ = 50 : 200 : 1). (c) Comparison of the rate constants (*k*_app_) for homo- and cross-propagation.

To elucidate the mechanism underlying this highly alternating copolymerization system, we conducted a series of kinetic studies using the optimal catalyst of *R*,*R*-4 (Fig. 2a). The homopolymerizations of CL and l-EG, as well as their equimolar copolymerization, were monitored over time in toluene ([*M*]_0_ = 1.0 M, [Cat.]_0_ = 0.02 M). All three polymerizations exhibited a linear relationship between ln([*M*]_0_/[*M*]_*t*_) and time, indicating a first-order dependence on monomer concentration. The homopolymerization of CL was extremely rapid, with an apparent rate constant (*k*_app(CL-CL)_) of 1.7729 h^−1^ (Equation (2) in Fig. 2c), while the homopolymerization of l-EG was exceptionally sluggish, with a *k*_app(EG-EG)_ of only 0.0015 h^−1^ (Equation (4) in Fig. 2c). Most strikingly, the apparent rate constant for their copolymerization (*k*_app(EG/CL)_ = 0.0381 h^−1^) was drastically slower (Equation (3) in Fig. 2c), by a factor of more than 46, than the homopolymerization of the fast monomer of CL. This dramatic rate suppression in alternating copolymerization indicates that the overall reaction is governed by a slow rate-determining step, where one cycle consists of two sequential cross-propagation steps: (i) the attack of l-EG by a CL-terminated chain end (*k*_app(CL-EG)_) and (ii) the attack of CL by an l-EG-terminated chain end (*k*_app(EG-CL)_) (Equations (1) and (3) in Fig. 2c). Because both homopolymerizations of CL and l-EG are side reactions (Equations (2) and (4) in Fig. 2c), the formation of a highly alternating copolymer obviously suggests that the attack of the CL-terminated chain end on an l-EG monomer is significantly faster than the attack on a CL monomer, *i.e. k*_app(CL-EG)_ > *k*_app(CL-CL)_. Thus, step (i) of *k*_app(CL-EG)_ is an exceptionally facile process, which means that the *R*,*R*-SalenAl-CL-chain intermediate is more active for l-EG than CL. Although CL is the more reactive monomer in homopolymerization than l-EG, the reactivity of l-EG is higher than CL here, which indicates that l-EG is a highly reactive monomer when steric hindrance of the substituted group and chirality of the catalyst are low enough. This phenomenon can also explain well the reversed reactivity order of l-LA and CL in their copolymerization relative to their homopolymerization in the literature.^34,36,37,41^ Given that the overall copolymerization is slow and step (i) must be fast, we logically conclude that the second cross-propagation event of step (ii), the reaction of attacking the CL monomer by the l-EG-terminated chain end (Equation (3) in Fig. 2c, *k*_app(EG-CL)_), is the rate-determining step for the entire polymerization. The reaction rates follow the order of *k*_app(CL-EG)_ > *k*_app(CL-CL)_ (1.7729 h^−1^) > *k*_app(EG-CL)_ (≈0.0381 h^−1^) > *k*_app(EG-EG)_ (0.0015 h^−1^), thus the polymerization kinetic constant should be determined by *k*_app(EG-CL)_, *i.e. k*_app(EG-CL)_ is equal to the apparent rate constant of the alternating copolymerization.

To further validate our kinetic conclusion, we designed an experiment to directly identify the predominant growing chain end of the copolymer. For this analysis, we chose the poly(l-LA-*alt*-CL) system because the methine proton signal of its l-LA-terminated chain end provides a more clearly resolved signal in the ^1^H NMR spectrum than the corresponding l-EG end group. A copolymerization of l-LA and CL was conducted using *R*,*R*-2 with a feed ratio of [l-LA]_0_ : [CL]_0_ : [Cat.]_0_ = 50 : 200 : 1. This specific ratio was chosen to generate lower molecular weight polymers, thereby increasing the relative concentration of chain ends to facilitate their detection by ^1^H NMR. The ^1^H NMR spectrum of the purified polymer (Fig. 2b) was dominated by a clear methine signal corresponding to the l-LA-terminated end group (4.35 ppm). Correspondingly, a much weaker methylene signal for the CL-terminated end group (–CH_2_OH) was also observed at 3.65 ppm. The integral ratio between the l-LA end-group signal and the CL end-group signal was 34 : 1 (Fig. S4). After normalization for the number of protons (one methine proton in the lactic acid-end unit *vs.* two methylene protons in the CL-end unit) the true molar ratio between the l-LA-terminated chain end and CL-terminated chain end should be 68 : 1. Considering the high CL : l-LA feed ratio of 4 : 1 and a ratio of more than 4 : 1 for CL : l-LA after partial conversion of monomers, the polymer end is still overwhelmingly capped with the l-LA unit in the presence of this large excess of CL, which provides unequivocal, direct evidence that the reaction of an l-LA-terminated chain end with a CL monomer is indeed the slow, rate-determining step during the entire alternating copolymerization. The results of this chain end group analysis agree well with the above kinetic conclusion.

**Fig. 3:**
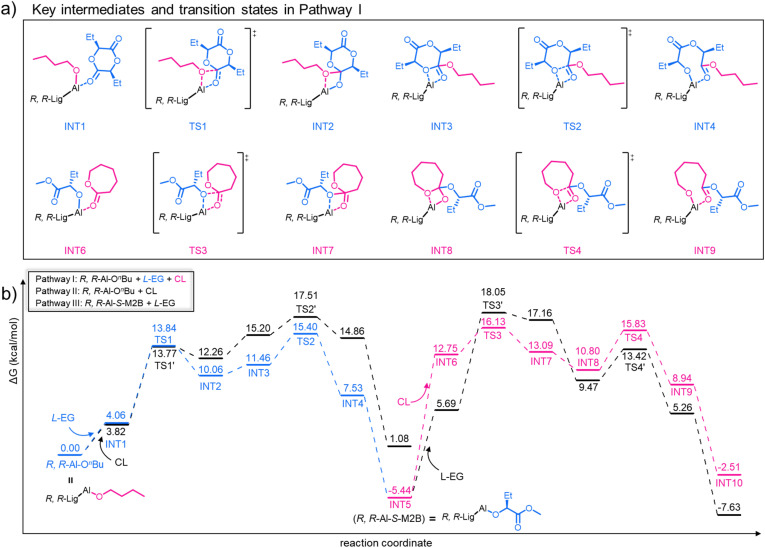
DFT-calculated mechanism for the key propagation pathways in the copolymerization of l-EG and CL catalyzed by *R*,*R*-1. (a) Structures of the key intermediates (INT) and transition states (TS) along the major reaction pathway (Pathway I). (b) The corresponding free energy profiles for all four key propagation pathways. All calculations were performed at the PWPB95-D4/def2-TZVPP/SMD(Toluene)//B3LYP-D3(BJ)/6-31G(d,p) level of theory.

To gain deeper insight into the origin of the high alternating selectivity and to corroborate the proposed mechanism, density functional theory (DFT) calculations were performed (Fig. 3). To balance high alternating selectivity with computational efficiency, the copolymerization of l-EG and CL with catalyst *R*,*R*-1 was selected as the model system. To further simplify the model while retaining the essential stereochemical features, the growing chain ends were simulated using proxy initiators: *n*-butanol (^*n*^BuOH) was used to mimic the CL-terminated chain end, and methyl (*S*)-2-hydroxybutanoate (*S*-M2B) was used to mimic the l-EG-terminated chain end. The polymerization is presumed to proceed *via* a coordination–insertion mechanism, widely accepted for SalenAl catalysts.^42^ The pathway for each monomer insertion involves two key transition states (TS): the initial nucleophilic attack of the alkoxide chain end on the coordinated monomer's carbonyl carbon and the subsequent ring-opening step where the acyl-oxygen bond is cleaved. To accurately determine the overall energy barrier for each propagation pathway, a comprehensive conformational search was performed for the two TS to locate their respective lowest-energy structures.^43^

The calculated energy barriers for the four key propagation pathways provide strong theoretical support for our experimental observations (Fig. 3). The CL homopropagation initiated by *n*-butanol has a calculated energy barrier (Δ*G*) of 17.51 kcal mol^−1^, while the cross-propagation from this same chain end is highly facile, with a significantly lower barrier of 15.40 kcal mol^−1^. Conversely, the reactions initiated by *S*-M2B face much higher barriers. The cross-propagation step has a barrier of 21.57 kcal mol^−1^, while the l-EG homopropagation step faces the most formidable barrier of all at 23.50 kcal mol^−1^. These calculated energy barriers align remarkably well with our experimental findings. The significant energy preference for cross-propagation over both types of homopropagation (ΔΔ*G* ≈ 2 kcal mol^−1^ in both cases) provides a powerful kinetic rationale for the high alternating selectivity observed experimentally. Moreover, the DFT results provide a comprehensive explanation for the observed polymerization kinetics. First, the calculated energy barriers provide direct theoretical support for our conclusion regarding the rate-determining step. The calculations show that step (i), the attack on l-EG by a CL-terminated chain end, has a low energy barrier of 15.40 kcal mol^−1^. In contrast, step (ii), the attack on CL by an l-EG-terminated chain end, faces a significantly higher barrier of 21.57 kcal mol^−1^. This confirms that step (ii) is indeed the slow, rate-determining step of the alternating cycle. Furthermore, the calculated energy barriers for the four key propagation pathways follow the trend: fast cross-propagation (CL-EG, 15.40 kcal mol^−1^) < CL homopropagation (17.51 kcal mol^−1^) < slow, rate-determining cross-propagation (EG-CL, 21.57 kcal mol^−1^) < l-EG homopropagation (23.50 kcal mol^−1^). This trend is fully consistent with the experimentally observed rate order of *k*_app(CL-CL)_ > *k*_app(EG-CL)_ > *k*_app(EG-EG)_, and supports the kinetic conclusion that *k*_app(CL-EG)_ > *k*_app(CL-CL)_. Then, a clear pattern emerges that the energy barriers for the ROP of CL and l-EG are significantly lower when initiated by *n*-butoxy than by *S*-M2B. This difference can be attributed to the different alkoxide types, where *n*-butoxy as a model of the CL-terminated chain end should be a highly effective nucleophile due to its small hindrance. Consequently, its initial nucleophilic attack (TS1, *i.e.*, TS1_path(CL-EG)_, TS1_path(CL-CL)_) is facile and has a lower energy barrier than the subsequent ring-opening step (TS2, *i.e.*, TS2_path(CL-EG)_, TS2_path(CL-CL)_). In contrast, the sterically hindered secondary alkoxy model, *S*-M2B, exhibits reduced nucleophilicity, which renders the initial nucleophilic attack (TS3, *i.e.*, TS3_path(EG-CL)_, TS3_path(EG-EG)_) the higher-energy and more challenging step than its subsequent ring-opening step (TS4, *i.e.*, TS4_path(EG-CL)_, TS4_path(EG-EG)_). These detailed calculation results suggest that the chain end's steric and electronic properties modulate not only the overall reaction barrier but also the fundamental profile of the insertion pathway itself.

**Fig. 4:**
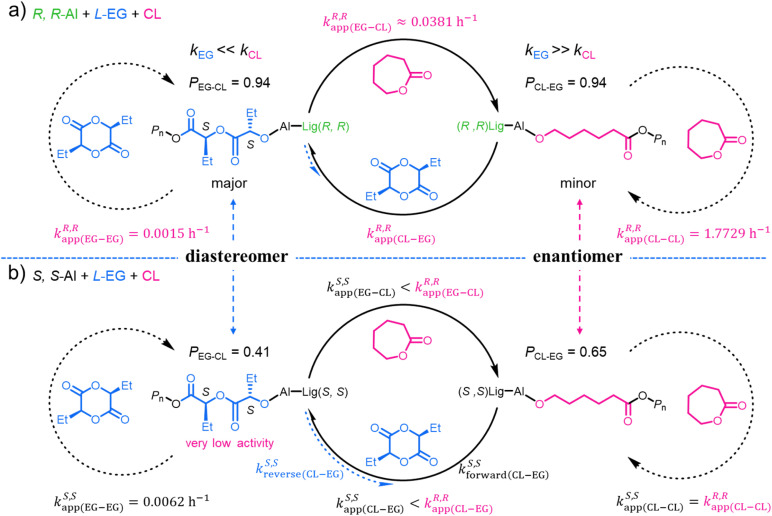
Kinetic rationale for the enantiomorphic site and chain-end synergistic control mechanism. (a) Kinetic profile for the *R*,*R*-Al catalyst. (b) Kinetic profile for the *S*,*S*-Al catalyst.

Switching the catalyst configuration from *R*,*R* to *S*,*S* significantly impaired the enantiomorphic site and chain-end synergistic control strategy, leading to a marked decrease in both catalytic activity and selectivity, as evidenced by a drop in alternating probabilities (*P*_EG-CL_ = 0.44 and *P*_CL-EG_ = 0.66, Table 1, Entry 13) and reduced catalytic performance (88% EG and 90% CL conversion in 48 h with *R*,*R vs.* 81% EG and 55% CL conversion in 114 h with *S*,*S*). Although the *S*,*S* configuration of the catalyst is beneficial for the insertion of l-EG, the significantly lower *P*_CL-EG_ value compared to the *R*,*R*-catalyst is clearly contrary to our intuition. This selectivity is kinetically governed by the competition between the cross-propagation of l-EG (*k*_app(CL-EG)_) and the homopropagation of CL (*k*_app(CL-CL)_) from a CL-terminated chain end. Given that the *S*,*S*-catalyst is beneficial for the insertion of l-EG, while the rate of CL homopolymerization should be identical for both enantiomeric catalysts, a higher *P*_CL-EG_ value is expected for the *S*,*S*-catalyst. But, why is *P*_CL-EG_ so low? To confirm that the insertion of l-EG is easier using the *S*,*S*-catalyst, the ring-opening of l-EG was conducted with isopropanol as an initiator (an achiral mimic of a CL-terminated chain end), using both *S*,*S*-4 and *R*,*R*-4 catalysts ([^*i*^PrOH]_0_ : [l-EG]_0_ : [Cat.]_0_ = 4 : 4:1); after 1 hour, ^1^H NMR analysis revealed 45% l-EG conversion for the *S*,*S*-4 catalyst, and only 19% for the *R*,*R*-4 catalyst (Fig. S14). This observation is consistent with the homopolymerization kinetics of l-EG, which also show a higher propagation rate for the *S*,*S*-catalyst (*k*^*S*,*S*^_app(EG-EG)_ = 0.0062 h^−1^*vs. k*^*R*,*R*^_app(EG-EG)_ = 0.0015 h^−1^ Fig. S15). These results confirm that the l-EG insertion rate is indeed faster with the *S*,*S*-catalyst. Anyway, after careful analyses, this apparent discrepancy between the high homopolymerization reactivity and the low apparent copolymerization performance can be rationalized by the reversibility of the l-EG insertion step. In the *S*,*S-*catalyst system, the low alternating probability (*P*_EG-CL_ = 0.41) indicates that the rate of CL insertion after the l-EG-chain end is slower than that of l-EG homopropagation, thus suggesting that the rate constant limit of *k*^*S*,*S*^_app(EG-CL)_ must be smaller than *k*^*S*,*S*^_app(EG-EG)_ = 0.0062 h^−1^. This rate constant upper limit is at least 6-fold smaller than the rate determined for the *R*,*R*-catalyst system (*k*^*R*,*R*^_app(EG-CL)_ ≈ 0.0381 h^−1^), which indicates well that *S*,*S*-SalenAl-l-EG-Chain is much less active than the diastereomer of *R*,*R*-SalenAl-l-EG-Chain; consequently this cross-propagation rate is very slow. As a result, this severe kinetic bottleneck of cross-propagation allows side reactions, such as back-biting and transesterification, to become kinetically significant (Fig. 4b, blue dashed arrow). For instance, because of the reversible depolymerization *via* back-biting, the *S*,*S*-SalenAl-l-EG-CL-Chain can revert to the *S*,*S*-SalenAl-CL-Chain (*k*^*S*,*S*^_reverse(CL-EG)_). This reversibility effectively reduces the net rate of successful cross-propagation events, causing the apparent rate constant (*k*^*S*,*S*^_app(CL-EG)_ = *k*^*S*,*S*^_forward(CL-EG)_ − *k*^*S*,*S*^_reverse(CL-EG)_) to be much lower than the intrinsic forward rate of insertion of l-EG after the CL-chain end and leading to a low *P*_CL-EG_ value.

Thus, as shown in Fig. 4, the high alternating fidelity is governed by an enantiomorphic site and chain-end synergistic control mechanism, where the intermediate activity is dynamically modulated by each last inserted unit. With the *R*,*R*-catalyst, the *R*,*R*-SalenAl-CL-Chain intermediate with an achiral CL-terminated chain end is highly active for the insertion of chiral l-EG. In the resulting chiral l-EG-terminated intermediate species (*R*,*R*-SalenAl-l*-*EG-Chain), the synergistic steric repulsion arising from both the *R*,*R*-catalyst's enantiomorphic site and the l-configuration of the last unit of the polymer chain end strongly disfavors l-EG homopropagation, rendering the slower insertion of an achiral CL monomer the only kinetically viable forward pathway. Thus, a highly alternating copolymerization cycle of l-EG and CL can be successfully established. Conversely, the *S*,*S*-catalyst fails to achieve highly alternating copolymerization of l-EG and CL *via* this mechanism due to both stereochemical and kinetic factors (Fig. 4b). Stereochemically, the *S*,*S*-catalyst's inherent preference for l-EG fails to suppress undesired l-EG homopropagation. Kinetically, an exceptionally slow subsequent cross-propagation step (*k*^*S*,*S*^_app(EG-CL)_) creates a bottleneck, which renders the l-EG insertion significantly reversible and thereby causes a decrease in the probability of *P*_CL-EG_. This comparative analysis unequivocally demonstrates that the successful enantiomorphic site and chain-end synergistic control mechanism is critically dependent on two synergistic factors: the effective shutting down of chiral cyclic ester homopropagation, and simultaneously, a sufficiently rapid forward propagation from this intermediate to kinetically suppress reversible side reactions.

### Thermal properties and hydrolytic degradation of copolymers

**Fig. 5:**
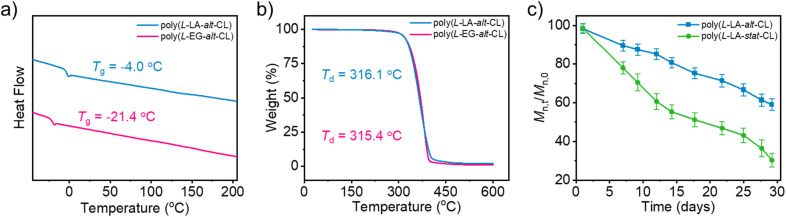
Thermal properties and hydrolytic degradation behavior of the sequence-controlled copolyesters. (a) DSC curves of poly(l-LA-*alt*-CL) and poly(l-EG-*alt*-CL) recorded from the second heating scan. (b) TGA curves of poly(l-LA-*alt*-CL) and poly(l-EG-*alt*-CL). *T*_d_ values refer to the decomposition temperatures at 5% weight loss. (c) Hydrolytic degradation behavior of poly(l-LA-*alt*-CL) and poly(l-LA-*stat*-CL), monitored by the change in number-average molecular weight over time. *M*_*n*,*t*_/*M*_*n*,0_ denotes the ratio of the number-average molecular weight at degradation time *t* to the initial number-average molecular weight. Error bars represent standard deviation from triplicate experiments.

The thermal properties of the resulting alternating copolymers, poly(l-LA-*alt*-CL) and poly(l-EG-*alt*-CL), were investigated by differential scanning calorimetry (DSC) and thermogravimetric analysis (TGA). The glass transition temperatures (*T*_g_) of the copolymers were determined from the second heating scan in the DSC analysis (Fig. 5a). Both polymers exhibited a single, distinct *T*_g_, indicating the formation of homogeneous amorphous materials. This amorphous characteristic is highly advantageous for applications such as drug delivery, as it can facilitate higher, more uniform drug loading and predictable release kinetics.^15,44,45^ As is characteristic for linear polymers, the *T*_g_ was found to be dependent on the number-average molecular weight (*M*_*n*_). To facilitate a direct comparison of the intrinsic segmental mobility of the polymer backbones, free from the plasticizing effect of chain ends, the theoretical *T*_g_ at infinite molecular weight (*T*_g_,_∞_) was determined by extrapolation using the Flory–Fox equation (Fig. S16).^46^ For poly(l-LA-*alt*-CL), the highest molecular weight sample (*M*_*n*_ = 44.8 kg mol^−1^) exhibited a *T*_g_ of −4.0 °C, while the extrapolated *T*_g,∞_ was found to be −2.9 °C. This intrinsic *T*_g_ value stands in sharp contrast to those of previously reported random poly(l-LA-*co*-CL), where the *T*_g_ exhibits a strong compositional dependence, varying widely from −19.5 °C to +26 °C as the LA content changes (Table S5). In stark contrast, the single, well-defined *T*_g_ of poly(l-LA-*alt*-CL) is an intrinsic property of the highly alternating copolymer, reflecting its uniform chemical environment. The poly(l-EG-*alt*-CL) copolymer with *M*_*n*_ = 34.2 kg mol^−1^ displayed a significantly lower *T*_g_ of −21.4 °C, which can be attributed to the internal plasticizing effect of the more flexible ethyl side group in the l-EG unit compared to the compact methyl group in the l-LA unit. The thermal stability of the copolymers was evaluated using TGA (Fig. 5b). Both polymers demonstrated excellent thermal stability, with the decomposition temperatures (*T*_d_, at 5% weight loss) of 316.1 °C for poly(l-LA-*alt*-CL) and 315.4 °C for poly(l-EG-*alt*-CL), respectively.

To further evaluate the sequence effect on the copolyesters, accelerated hydrolytic degradation experiments were performed in buffer solution at 50 °C. The degradation behavior was monitored by the decrease in number-average molecular weight over time (Fig. 5c). Under identical conditions, poly(l-LA-*alt*-CL) showed a more gradual decrease in *M*_*n*_ than the statistical poly(l-LA-*stat*-CL) analogue. After 29 days, poly(l-LA-*alt*-CL) retained approximately 59% of its initial *M*_*n*_, whereas poly(l-LA-*stat*-CL) retained only approximately 30%. Thus, these results suggest that the sequence regulation can influence the hydrolytic stability of LA/CL copolyesters, which may be potentially useful for medical applications.

## Conclusions

In conclusion, we have successfully realized the first highly alternating copolymerization of two common industrial monomers, l-lactide and ε-caprolactone, achieving an alternating probability (*P*_alt_) of up to 0.91 through an enantiomorphic site and chain-end synergistic control strategy. The universality of this approach was further demonstrated by the highly alternating copolymerization of l-ethylglycolide and ε-caprolactone (*P*_alt_ = 0.96). Specifically, the high fidelity of this alternating copolymerization process arises from a synergistic interplay between the enantiomorphic site of the catalyst and the polymer chain end. This interaction generates a significant steric barrier that selectively and effectively inhibits the competing homopropagation of the highly reactive chiral monomer (l-LA or l-EG). Consequently, this steric exclusion renders the slower insertion of the achiral CL monomer (the rate-determining step) the only kinetically viable pathway, thereby enforcing a highly alternating sequence. This mechanistic model is fully consistent with kinetic, spectroscopic, and computational evidence. Ultimately, beyond this specific system, our findings establish enantiomorphic site and chain-end synergistic control as a generalizable paradigm for sequence control in ring-opening polymerization.

## Author contributions

Jincai Wu and Ji Xian conceived the idea and designed the experiments. Ji Xian and Jincai Wu co-wrote the manuscript and all authors contributed to the data analysis and discussions and the revised manuscript.

## Conflicts of interest

There are no conflicts to declare.

## Supplementary Material

SC-OLF-D6SC04630D-s001

## Data Availability

The data supporting this article have been included as part of the supplementary information (SI). Supplementary information: Tables S1 and S7, NMR spectra and further experimental details. See DOI: https://doi.org/10.1039/d6sc04630d.
