# Supplementary material for: Alternating copolymerization of l-lactide and ε-caprolactone via enantiomorphic site and chain-end synergistic control
Source: Chem Sci. 2026 Jun 22. Online ahead of print. doi: 10.1039/d6sc04630d (PMC13284822; doi:10.1039/d6sc04630d)
Supplement: SC-OLF-D6SC04630D-s001 [file SC-OLF-D6SC04630D-s001.pdf]

Supplementary Information (SI) for Chemical Science.
This journal is © The Royal Society of Chemistry 2026

Supporting information

Alternating Copolymerization of *L*-Lactide and *ε*-Caprolactone via Enantiomorphic Site and Chain-End Synergistic Control

Ji Xian, Guojie Li, Linyun Wu, Hongjun Fu, Chunmei Wang, Shaoyu Lü, Xiaobo Pan, Jincai Wu*

State Key Laboratory of Natural Product Chemistry (Lanzhou University), Key Laboratory of Nonferrous Metal Chemistry and Resources Utilization of Gansu Province, College of Chemistry and Chemical Engineering, Lanzhou University, Lanzhou 730000, People's Republic of China.

*Correspondence to: wujc@lzu.edu.cn

This PDF file includes:

Experimental details

Figures S1 to S42

Table S1 to S7

EXPERIMENTAL SECTION

General methods:

Unless otherwise specified, all ROP catalysts experiments were conducted in dry nitrogen atmosphere. All syntheses and manipulations of air/moisture-sensitive materials were performed under a dry nitrogen atmosphere in a glovebox or using standard Schlenk techniques.

Materials:

Toluene and hexane were distilled from sodium benzophenone before use. *L*-lactide, *D*-lactide and *rac*-lactide were purchased from Energy Chemical Co., Ltd and used after recrystallization with toluene three times and sublimation. *ε*-Caprolactone (CL) was purchased from TCI, dried over CaH_2, and distilled before use. Chloroform-d was purchased from Adamas and carefully dried over activated molecular sieves. Ethylenediamine was purchased from Energy Chemical Co., Ltd. 1,1'-bi-2-naphthol was purchased from Bide Pharmatech Ltd. (Shanghai, China). $Al(O^iPr)_3$ was purchased from TCI. *L*-ethyl glycolide (*L*-EG) and the SalenAl complexes bearing phenyl, benzyl, and 3,5-dimethylphenyl substituents were synthesized according to our previously reported procedures.[1] All other chemicals were commercially available and used as received.

Measurements

^1H and ^{13}C NMR spectra were recorded on Bruker 400 MHz and 600 MHz spectrometers. Chemical shifts (δ) are reported in parts per million (ppm). ^1H NMR spectra were referenced to tetramethylsilane (TMS) as

an internal standard (δ = 0.00 ppm). ^{13}C NMR spectra were referenced to the solvent resonance ($CDCl_3$ at δ 77.0 ppm).

The molecular weights (M_n) and the molecular mass distributions (M_w/M_n) of the polymer samples were determined by gel permeation chromatography (GPC) using THF as the eluent (flow rate: 1.0 mL min^{-1} at 40 °C), and narrow polystyrene standards as reference samples. The measurements were performed using a Shodex GPC KF-800 system that was equipped with a Shodex RI-201H detector using Shodex-KF-803 and Shodex-KF-804 Styragel columns (0.4 - 400 kg/mol).

Matrix-Assisted Laser Desorption/Ionization Time of Flight (MALDI-TOF) Mass Spectrometry conditions were as follows. Instrument type: BIFLEX III MALDI-TOF mass spectrometer, adjust to reflection mode, Power 80, P. Ext at 5000.00. The MALDI-TOF mass spectroscopic data were obtained using trans-2-[3-(4-tert-butylphenyl)-2-methyl-2-propenylidene]alonitrile (DCTB) as the matrix (10 mg/mL in THF), sodium trifluoroacetate as the cationization agent (10 mg/mL in THF) and samples were dissolved in THF (10 mg/mL). The solutions of samples, matrix, and salt were mixed in a volume ratio of 1:1:1; then the mixed solution (1 μL) was hand-spotted on a stainless steel MALDI target, which allowed to be dried completely.

Differential scanning calorimetry (DSC) measurements, using 5-10 mg of material, DSC experiments were performed on a DZ-DSC300C instrument with a heat (-60-210 °C) /cool 210-60 °C)/heat (-60-210 °C) cycle at a heating rate of 10 °C/min and cooling rate of 10 °C/min under N_2 atmosphere. All T_g values were obtained from a second heating scan.

Thermal gravimetric analyzer (TGA): using 5-10 mg of material, TGA experiments were performed on a Linseis PT 1600 instrument, with a heat (25-600 °C) at a rate of 10 °C/min under N_2 atmosphere. Decomposition temperatures (T_d, defined by the temperature of 5 % weight loss).

High Resolution MS (ESI-HRMS) spectra were determined on a Bruker maxis EIS-Q-TOF and Thermo Scientific UHPLC-Exactive Orbitrap MS spectrometer.

General synthetic route for 2-hydroxy-2'-(alkoxy)-1,1'-binaphthyl-3-carbaldehydes

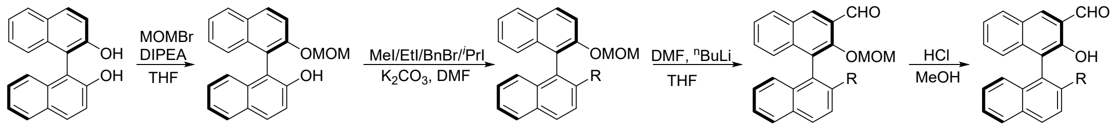

The target aldehydes were synthesized via a four-step sequence starting from BINOL. following a procedure from the literature.[2] The general route involves (1) mono-protection of (*R*)-BINOL, (2) alkylation of the free hydroxyl group, (3) directed ortho-formylation, and (4) acidic deprotection of the MOM group. The synthesis of the methoxy derivative (R = OMe) is described in detail as a representative example. All other derivatives (R = OEt, O^{i}Pr, OBn) were prepared following these procedures by using the corresponding alkylating agents.

Bromomethyl methyl ether (MOMBr, 1.55 mL, 20 mmol) was dissolved in THF (20 mL). The resulting solution was added dropwise over 40 min to a stirred solution of (*R*)-1,1'-bi-2-naphthol ((*R*)-BINOL, 5.72 g, 20 mmol) and *N,N*-diisopropylethylamine (DIPEA, 7.32 mL, 42 mmol) in THF (60 mL) at 0 °C. The reaction mixture was allowed to warm to room temperature and stirred for 12 h. The reaction was then quenched by the addition of acetic acid (1.15 mL, 20 mmol). The solvent was removed under reduced pressure, and the residue was dissolved in ethyl acetate (120 mL). The organic phase was washed sequentially with water (1 ×) and brine (2 ×), dried over anhydrous Na_2SO_4, filtered, and concentrated in vacuo. The crude product was purified by silica-gel column chromatography to afford (*R*)-2-methoxymethoxy-2’-hydroxy-1,1’-binaphthyl

as a white solid (5.58 g, 85% yield). ^{1}H NMR (400 MHz, Chloroform-*d*) δ 7.94 (dd, *J* = 9.0, 3.9 Hz, 2H), 7.86 (dd, *J* = 8.2, 5.5 Hz, 2H), 7.56 (d, *J* = 9.0 Hz, 1H), 7.42 (d, *J* = 9.0 Hz, 1H), 7.36 – 7.27 (m, 2H), 7.23 – 7.17 (m, 2H), 7.13 (d, *J* = 8.5 Hz, 2H), 5.07 (d, *J* = 6.8 Hz, 1H), 4.97 (d, *J* = 6.7 Hz, 1H), 3.16 (s, 3H).

To a solution of (*R*)-2-methoxymethoxy-2'-hydroxy-1,1'-binaphthyl (5.58 g, 17 mmol) in anhydrous DMF (90 mL) were added potassium carbonate (K_2CO_3, 4.7 g, 34 mmol) and iodomethane (1.58 mL, 25 mmol). The resulting mixture was stirred at room temperature for 20 h. The reaction was then diluted with 0.5 M aqueous NaOH solution (180 mL), and the mixture was extracted with diethyl ether (3 ×). The combined organic layers were washed with brine, dried over anhydrous Na_2SO_4, filtered, and concentrated in vacuo. The crude residue was purified by silica-gel column chromatography to afford (*R*)-2-methoxymethoxy-2'-methoxy-1,1'-binaphthyl as the product (5.17 g, 88% yield). ^{1}H NMR (400 MHz, Chloroform-*d*) δ 7.97 (dd, *J* = 14.8, 8.8 Hz, 2H), 7.87 (ddt, *J* = 8.2, 3.7, 1.0 Hz, 2H), 7.57 (d, *J* = 9.0 Hz, 1H), 7.46 (dd, *J* = 9.0, 1.3 Hz, 1H), 7.38 – 7.28 (m, 2H), 7.24 – 7.18 (m, 2H), 7.12 (dd, *J* = 8.4, 1.1 Hz, 2H), 5.07 (d, *J* = 6.8 Hz, 1H), 4.99 (d, *J* = 6.8 Hz, 1H), 3.77 (s, 3H), 3.16 (s, 3H).

To a solution of (*R*)-2-methoxymethoxy-2'-methoxy-1,1'-binaphthyl (5.17 g, 15 mmol) in anhydrous THF was added dropwise n-butyllithium (^{n}BuLi, 15 mL, 37.5 mmol, 2.5 M in hexanes) at -78 °C. The mixture was stirred at -78 °C for 3 h, after which anhydrous DMF (5.8 mL, 75 mmol) was added. The reaction mixture was then allowed to warm to room temperature and stirred for an additional 1 h. The reaction was quenched by the addition of saturated aqueous NH_4Cl solution (40 mL), and the aqueous layer was extracted with ethyl acetate (3 ×). The combined organic layers were washed sequentially with saturated aqueous $NaHCO_3$ solution and brine, dried over anhydrous Na_2SO_4, filtered, and concentrated in vacuo. The crude intermediate was dissolved in methanol (60 mL), and concentrated hydrochloric acid (3 mL) was added. The resulting solution was heated to 60 °C and stirred for 3 h. After cooling to room temperature, the solvent was removed under reduced pressure. The residue was purified by silica-gel column chromatography to afford (*R*)-2-hydroxy-2'-methoxy-1,1'-binaphthyl-3-carbaldehyde (3.6 g, 73% yield over two steps).^{1}H NMR (400 MHz, Chloroform-*d*) δ 10.44 (s, 1H), 10.19 (s, 1H), 8.31 (s, 1H), 8.02 (d, *J* = 9.1 Hz, 1H), 8.00 – 7.95 (m, 1H), 7.89 (d, *J* = 8.2 Hz, 1H), 7.48 (d, *J* = 9.0 Hz, 1H), 7.41 – 7.30 (m, 3H), 7.24 (dd, *J* = 6.8, 1.5 Hz, 1H), 7.19 – 7.10 (m, 2H), 3.80 (s, 3H).

General procedure for the synthesis of salen ligands

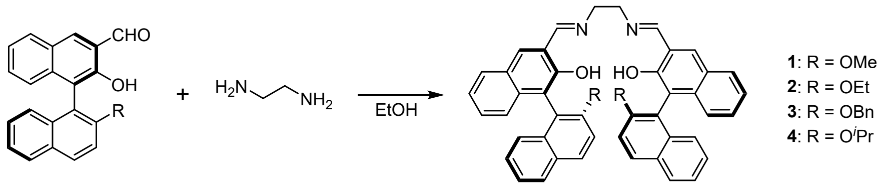

Take *R, R*-**1** ligand as an example, a mixture of (*R*)-2-hydroxy-2'-methoxy-1,1'-binaphthyl-3-carbaldehyde (0.656 g, 2.0 mmol) and ethylenediamine (0.060 g, 1.0 mmol) in ethanol (10 mL) was heated to 70 °C and stirred overnight. Upon cooling to room temperature, the resulting yellow precipitate was collected by filtration. The solid was washed with cold ethanol (2 × 5 mL) and dried in vacuo to afford the desired Schiff base ligand as a yellow solid (0.673 g, 95% yield). ^{1}H NMR (600 MHz, Chloroform-*d*) δ 12.93 (s, 2H), 8.52 (s, 2H), 7.97 (d, *J* = 9.0 Hz, 2H), 7.89 – 7.84 (m, 2H), 7.81 (s, 2H), 7.79 – 7.75 (m, 2H), 7.45 (d, *J* = 9.0 Hz, 2H), 7.31 – 7.21 (m, 6H), 7.18 – 7.12 (m, 4H), 7.11 – 7.05 (m, 2H), 3.95 – 3.85 (m, 4H), 3.71 (s, 6H). ^{13}C NMR (151 MHz, Chloroform-*d*) δ 166.79, 155.08, 154.36, 135.41, 133.77, 133.33, 129.65, 129.34, 128.75,

128.13, 128.02, 127.32, 126.46, 125.02, 124.79, 123.54, 123.17, 120.56, 118.80, 117.26, 114.25, 59.63, 56.88. HRMS (ESI): m/z calculated for $C_{46}H_{36}N_2O_4$ $[M + H]^+$ 681.2753, found 681.2759.

R, R-**2** ligand: ^{1}H NMR (600 MHz, Chloroform-*d*) δ 12.91 (s, 2H), 8.53 (s, 2H), 7.94 (d, *J* = 9.0 Hz, 2H), 7.85 (d, *J* = 8.2 Hz, 2H), 7.81 (s, 2H), 7.79 – 7.75 (m, 2H), 7.42 (d, *J* = 9.0 Hz, 2H), 7.30 (ddd, *J* = 8.1, 6.1, 1.8 Hz, 2H), 7.27 – 7.21 (m, 4H), 7.20 – 7.14 (m, 4H), 7.12 – 7.07 (m, 2H), 4.06 – 3.94 (m, 4H), 3.91 (s, 4H), 0.93 (s, 6H). ^{13}C NMR (151 MHz, Chloroform-*d*) δ 166.83, 154.45, 154.34, 135.44, 133.85, 133.22, 129.48, 128.66, 128.00, 127.99, 127.27, 126.36, 125.16, 124.93, 123.61, 123.08, 120.47, 119.85, 117.48, 116.17, 65.45, 59.63, 14.91. HRMS (ESI): m/z calculated for $C_{48}H_{40}N_2O_4$ $[M + H]^+$ 709.3066, found 709.3072.

R, R-**3** ligand: ^{1}H NMR (600 MHz, Chloroform-*d*) δ 12.97 (s, 2H), 8.42 (s, 2H), 7.88 (d, *J* = 9.0 Hz, 2H), 7.85 – 7.81 (m, 2H), 7.73 (dd, *J* = 8.0, 1.7 Hz, 2H), 7.69 (s, 2H), 7.38 (d, *J* = 9.0 Hz, 2H), 7.29 (ddd, *J* = 8.0, 6.6, 1.3 Hz, 2H), 7.25 – 7.20 (m, 6H), 7.19 – 7.09 (m, 4H), 7.04 (dd, *J* = 5.2, 1.9 Hz, 6H), 6.95 (dd, *J* = 6.9, 2.8 Hz, 4H), 5.10 – 4.94 (m, 4H), 3.81 (s, 4H). ^{13}C NMR (151 MHz, Chloroform-*d*) δ 166.73, 154.44, 154.11, 137.48, 135.44, 133.85, 133.35, 129.56, 129.47, 128.74, 128.12, 128.05, 128.01, 127.30, 127.26, 126.85, 126.45, 125.21, 124.97, 123.77, 123.14, 120.51, 120.01, 117.27, 116.09, 71.23, 59.63. HRMS (ESI): m/z calculated for $C_{58}H_{44}N_2O_4$ $[M + H]^+$ 833.3379, found 833.3360.

R, R-**4** ligand: ^{1}H NMR (600 MHz, Chloroform-*d*) δ 12.89 (s, 2H), 8.49 (s, 2H), 7.91 (d, *J* = 8.9 Hz, 2H), 7.84 (d, *J* = 8.2 Hz, 2H), 7.78 (s, 2H), 7.76 – 7.73 (m, 2H), 7.41 (d, *J* = 9.0 Hz, 2H), 7.29 (ddd, *J* = 8.1, 6.5, 1.4 Hz, 2H), 7.24 – 7.17 (m, 6H), 7.16 – 7.13 (m, 2H), 7.12 – 7.09 (m, 2H), 4.34 (p, *J* = 6.1 Hz, 2H), 3.89 (s, 4H), 0.90 (dd, *J* = 11.3, 6.1 Hz, 12H). ^{13}C NMR (151 MHz, Chloroform-*d*) δ 166.77, 154.33, 153.98, 135.41, 133.96, 133.17, 129.68, 129.27, 128.56, 127.96, 127.93, 127.16, 126.25, 125.27, 125.09, 123.74, 123.05, 121.42, 120.36, 118.54, 117.71, 72.66, 59.66, 22.38, 22.30. HRMS (ESI): m/z calculated for $C_{50}H_{44}N_2O_4$ $[M + H]^+$ 737.3379, found 737.3367.

General procedure for the synthesis of aluminum salen complexes

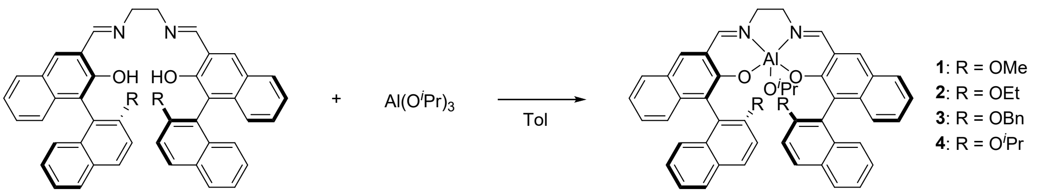

Take *R, R*-**1** as an example, in a glovebox, the Schiff base ligand (0.204 g, 0.30 mmol) and activated aluminum isopropoxide (Al(O^{i}Pr)$_3$, 0.062 g, 0.30 mmol) were dissolved in anhydrous toluene (6 mL). The reaction mixture was heated to 80 °C and stirred for 3 days. After cooling to room temperature, the solvent was removed in vacuo. The resulting solid residue was washed with n-hexane, and the product was collected by filtration and dried, affording the desired aluminum complex (0.205 g, 90%).^{1}H NMR (600 MHz, Chloroform-*d*) δ 8.42 (s, 1H), 8.31 (s, 1H), 8.08 – 8.01 (m, 2H), 7.95 – 7.88 (m, 2H), 7.75 (s, 1H), 7.69 (d, *J* = 9.0 Hz, 2H), 7.63 (dd, *J* = 7.4, 2.0 Hz, 1H), 7.21 (t, *J* = 6.8 Hz, 1H), 7.15 – 7.00 (m, 10H), 6.95 (d, *J* = 8.6 Hz, 1H), 6.74 (dd, *J* = 8.0, 1.7 Hz, 1H), 6.65 (dd, *J* = 8.2, 1.6 Hz, 1H), 4.25 (tdd, *J* = 11.8, 5.9, 1.7 Hz, 1H), 3.74 (ddd, *J* = 14.9, 5.7, 2.3 Hz, 1H), 3.65 – 3.58 (m, 1H), 3.51 – 3.40 (m, 2H), 2.97 (s, 3H), 2.74 (s, 3H), 0.62 (d, *J* = 6.0 Hz, 3H), 0.44 (d, *J* = 6.0 Hz, 3H). ^{13}C NMR (151 MHz, Chloroform-*d*) δ 171.33, 167.44, 158.84, 158.13, 155.83, 155.45, 138.34, 137.81, 136.15, 135.33, 133.98, 133.94, 129.51, 129.26, 129.04, 128.94, 128.80, 128.78, 128.57, 128.37, 127.71, 127.32, 125.98, 125.72, 125.65, 125.53, 125.25, 124.87,

123.32, 123.04, 122.16, 122.00, 121.79, 121.72, 121.47, 120.89, 120.70, 120.54, 117.82, 116.97, 77.00, 62.22, 57.43, 57.35, 56.09, 54.76, 27.35, 27.15. HRMS (ESI): m/z calculated for $C_{49}H_{41}AlN_2O_5$ $[M + H]^+$ 765.2909, found 765.2901.

R, R-**2**: ^{1}H NMR (600 MHz, Chloroform-*d*) δ 8.48 (s, 1H), 8.30 (s, 1H), 8.05 – 7.97 (m, 2H), 7.89 (d, *J* = 8.3 Hz, 2H), 7.80 – 7.72 (m, 2H), 7.71 – 7.64 (m, 2H), 7.25 – 7.18 (m, 3H), 7.15 (dd, *J* = 6.6, 2.0 Hz, 2H), 7.10 – 6.99 (m, 6H), 6.92 – 6.87 (m, 1H), 6.80 – 6.75 (m, 1H), 6.62 (d, *J* = 8.0 Hz, 1H), 4.33 (tdd, *J* = 12.1, 5.9, 1.8 Hz, 1H), 3.80 – 3.72 (m, 1H), 3.68 – 3.56 (m, 1H), 3.51 – 3.42 (m, 2H), 3.37 (dq, *J* = 10.4, 7.0 Hz, 1H), 3.12 (dq, *J* = 10.4, 7.1 Hz, 1H), 2.90 (dq, *J* = 9.6, 7.0 Hz, 1H), 2.80 (dq, *J* = 9.6, 6.9 Hz, 1H), 0.63 (d, *J* = 5.9 Hz, 3H), 0.52 (t, *J* = 7.0 Hz, 3H), 0.48 (d, *J* = 6.0 Hz, 3H), 0.20 (t, *J* = 7.0 Hz, 3H). ^{13}C NMR (151 MHz, Chloroform-*d*) δ 171.50, 167.21, 158.96, 158.08, 155.62, 155.19, 138.48, 137.90, 136.04, 135.29, 134.26, 133.89, 129.83, 129.68, 128.90, 128.76, 128.69, 128.66, 128.34, 128.27, 127.70, 127.45, 126.14, 125.94, 125.62, 125.51, 125.39, 125.07, 123.37, 123.18, 123.10, 122.43, 122.11, 121.95, 121.71, 121.26, 121.01, 120.84, 120.10, 119.66, 77.00, 66.93, 66.86, 62.20, 56.32, 54.69, 27.42, 27.30, 15.49, 14.96. HRMS (ESI): m/z calculated for $C_{51}H_{45}AlN_2O_5$ $[M + H]^+$ 793.3222, found 793.3227.

R, R-**3**: ^{1}H NMR (600 MHz, Chloroform-*d*) δ 8.31 (d, *J* = 1.6 Hz, 1H), 8.10 (s, 1H), 7.99 – 7.94 (m, 4H), 7.76 (s, 1H), 7.74 (dd, *J* = 8.1, 1.4 Hz, 1H), 7.68 – 7.64 (m, 1H), 7.52 (s, 1H), 7.38 (ddd, *J* = 8.0, 6.6, 1.2 Hz, 1H), 7.32 (ddd, *J* = 8.0, 6.4, 1.4 Hz, 1H), 7.23 (s, 1H), 7.13 – 6.99 (m, 12H), 6.91 – 6.86 (m, 1H), 6.83 (t, *J* = 7.5 Hz, 2H), 6.79 – 6.75 (m, 1H), 6.73 – 6.68 (m, 3H), 6.42 (d, *J* = 7.1 Hz, 2H), 4.64 (d, *J* = 13.9 Hz, 1H), 4.19 (d, *J* = 12.0 Hz, 1H), 4.17 – 4.10 (m, 1H), 4.07 (d, *J* = 14.0 Hz, 1H), 3.77 (d, *J* = 12.0 Hz, 1H), 3.63 (ddd, *J* = 13.6, 5.9, 2.9 Hz, 1H), 3.57 – 3.49 (m, 1H), 3.47 – 3.36 (m, 2H), 0.52 (d, *J* = 6.0 Hz, 3H), 0.30 (d, *J* = 6.0 Hz, 3H). ^{13}C NMR (151 MHz, Chloroform-*d*) δ 170.76, 167.66, 158.62, 158.24, 155.13, 155.02, 138.98, 138.80, 138.33, 137.93, 136.10, 135.34, 134.18, 134.00, 129.85, 129.37, 129.10, 128.91, 128.71, 128.68, 128.31, 127.93, 127.78, 127.62, 127.27, 126.65, 126.55, 126.44, 126.40, 126.24, 125.94, 125.86, 125.59, 125.56, 125.55, 125.43, 125.11, 123.70, 123.64, 123.27, 122.17, 121.97, 121.65, 121.49, 121.27, 120.90, 120.62, 119.86, 118.09, 77.00, 72.45, 71.52, 62.35, 55.95, 54.84, 27.25, 27.24. HRMS (ESI): m/z calculated for $C_{61}H_{49}AlN_2O_5$ $[M + H]^+$ 917.3535, found 917.3538.

R, R-**4**: ^{1}H NMR (600 MHz, Chloroform-*d*) δ 8.50 (s, 1H), 8.30 (s, 1H), 7.95 (d, *J* = 9.0 Hz, 1H), 7.89 (d, *J* = 8.9 Hz, 1H), 7.87 – 7.80 (m, 2H), 7.77 (s, 1H), 7.74 (s, 1H), 7.72 (d, *J* = 8.1 Hz, 1H), 7.70 – 7.65 (m, 1H), 7.24 – 7.22 (m, 1H), 7.13 (dd, *J* = 8.2, 1.3 Hz, 2H), 7.10 – 7.02 (m, 7H), 6.97 (ddd, *J* = 8.2, 6.6, 1.3 Hz, 1H), 6.88 (d, *J* = 8.6 Hz, 1H), 6.79 – 6.75 (m, 1H), 6.62 (d, *J* = 8.6 Hz, 1H), 4.35 (dddd, *J* = 11.9, 7.7, 6.0, 3.0 Hz, 1H), 3.87 (p, *J* = 6.1 Hz, 1H), 3.80 (ddd, *J* = 13.9, 5.8, 2.1 Hz, 1H), 3.69 – 3.60 (m, 1H), 3.52 (p, *J* = 6.0 Hz, 1H), 3.47 (ddd, *J* = 11.9, 5.8, 2.1 Hz, 1H), 3.26 (hept, *J* = 6.1 Hz, 1H), 0.70 (d, *J* = 6.1 Hz, 3H), 0.66 – 0.61 (m, 6H), 0.55 (d, *J* = 6.0 Hz, 3H), 0.33 (d, *J* = 6.1 Hz, 3H), 0.10 (d, *J* = 6.0 Hz, 3H). ^{13}C NMR (151 MHz, Chloroform-*d*) δ 171.37, 167.33, 158.96, 158.20, 154.45, 154.27, 138.66, 137.91, 135.79, 135.21, 134.52, 134.10, 129.46, 129.43, 128.77, 128.56, 128.46, 128.39, 128.37, 128.13, 127.63, 127.36, 126.12, 125.84, 125.72, 125.52, 125.45, 125.33, 125.27, 123.26, 123.00, 122.99, 122.65, 122.01, 121.79, 121.64, 121.53, 121.35, 119.75, 118.98, 77.00, 73.52, 72.97, 62.08, 56.38, 54.86, 27.49, 27.27, 22.82, 22.24, 22.18, 21.98. HRMS (ESI): m/z calculated for $C_{53}H_{49}AlN_2O_5$ $[M + H]^+$ 821.3535, found 821.3543.

General procedure for the solution copolymerization of cyclic diesters and *ε*-caprolactone

A typical copolymerization procedure is illustrated by the synthesis of poly(*L*-LA-*co*-CL) ($[Cat.]_0:[L\text{-}LA]_0:[CL]_0$ = 1:50:50, Table 1, entry 1). In a glovebox, a vial was charged with L-LA (36 mg, 0.25 mmol), ε-caprolactone (CL, 28 mg, 0.25 mmol), and the *R, R*-**1** (3.8 mg, 0.005 mmol). Anhydrous toluene (0.1 mL)

was added, and the vial was sealed with a Teflon-lined cap. The vial was then removed from the glovebox. The reaction mixture was stirred at 70 °C for 18 hours using a heating block. After the specified time, the vial was removed from the heat and allowed to cool to room temperature. An aliquot was taken directly from the reaction mixture and dissolved in $CDCl_3$ to determine monomer conversion by ^1H NMR spectroscopy. The remaining viscous solution was diluted with CH_2Cl_2 (0.5 mL) and subsequently added dropwise into vigorously stirred methanol (2 mL). The precipitated solid was purified by three cycles of dissolution in CH_2Cl_2 and re-precipitation into methanol. The final polymer was collected by filtration, washed with methanol, and dried in a vacuum oven at 50 °C for 8 h to a constant weight before GPC and NMR analyses.

General procedure for the bulk copolymerization of *L*-lactide and *ε*-caprolactone

A typical copolymerization procedure is illustrated by the synthesis of poly(*L*-LA-*alt*-CL) ($[Cat.]_0:[L\text{-}LA]_0:[CL]_0$ = 1:100:400, Table 1, entry 5). In a glovebox, a vial was charged with L-Lactide (L-LA, 72 mg, 0.5 mmol), ε-caprolactone (CL, 224 mg, 2.0 mmol), and the *R, R*-**1** (3.8 mg, 0.005 mmol). The vial was sealed with a Teflon-lined cap. The vial was then removed from the glovebox. The reaction mixture was stirred at 70 °C for 48 hours using a heating block. After the specified time, the vial was removed from the heat and allowed to cool to room temperature. An aliquot was taken directly from the highly viscous product and dissolved in $CDCl_3$ to determine monomer conversion by ^1H NMR spectroscopy. The remaining polymer was dissolved in a minimum amount of CH_2Cl_2 (1 mL) and subsequently added dropwise into vigorously stirred methanol (5 mL). The precipitated solid was purified by three cycles of dissolution in CH_2Cl_2 and re-precipitation into methanol. The final polymer was collected by filtration, washed with methanol, and dried in a vacuum oven at 50 °C for 8 h to a constant weight before GPC and NMR analyses.

Hydrolytic degradation experiments.

For each degradation time point, approximately 10 mg of polymer sample was placed at the bottom of a 2 mL glass vial and spread into a thin gel-like layer. The vial was heated under an argon atmosphere to obtain a more uniform sample layer. Then, 1.8 mL of preheated pH 7.4 PBS solution containing 0.04% v/v ProClin 300 was added to the vial. The sealed vial was placed in an oven at 50 °C for the desired time. At each predetermined time point, the corresponding vial was removed from the oven, and the supernatant buffer solution was carefully removed. The remaining polymer sample was washed with deionized water three times to remove residual buffer and then dried using a vacuum pump before GPC analysis. The dried sample was dissolved in THF and analyzed by GPC. The degradation behavior was evaluated by the ratio $M_{n,t}/M_{n,0}$, where $M_{n,t}$ is the number-average molecular weight after degradation time t, and $M_{n,0}$ is the initial number-average molecular weight before degradation. The same buffer solution was used for all degradation experiments, and the reported data are shown as mean ± SD from three independent hydrolytic degradation experiments.

Table S1. Results for the Alternating Copolymerization of *L*-LA with CL [a].

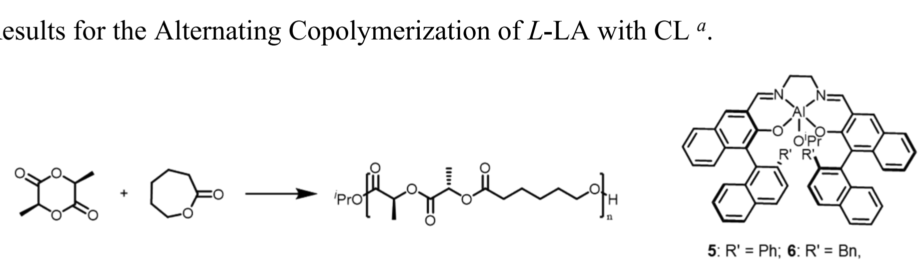

5: R' = Ph; **6**: R' = Bn,
7: R' = 3,5-*di*-CH_3-C_6H_3

Entry	Catalyst	Time (h)	Conv. [b] (%)	$P_{LA\text{-}CL}$ [c]	$P_{CL\text{-}LA}$ [c]

			LA, CL		
1	*R, R*-**5**	133	55, 42	0.68	0.95
2	*R, R*-**6**	54	94, 74	0.70	0.93
3	*R, R*-**7**	279	53, 18	0.30	0.92

[a] General conditions: [*L*-LA]$_0$:[CL]$_0$:[Cat.]$_0$ = 50:50:1; performed in toluene with [*L*-LA]$_0$ = 2.5 M and [CL]$_0$ = 2.5 M, under a dry argon atmosphere at 70°C. [b] Monomer conversion was determined by integrating selected monomer and polymer signals in the ^{1}H NMR spectrum. [c] The linkage probabilities $P_{LA\text{-}CL}$ and $P_{CL\text{-}LA}$ were determined by ^{1}H NMR analysis of the resulting copolymer.

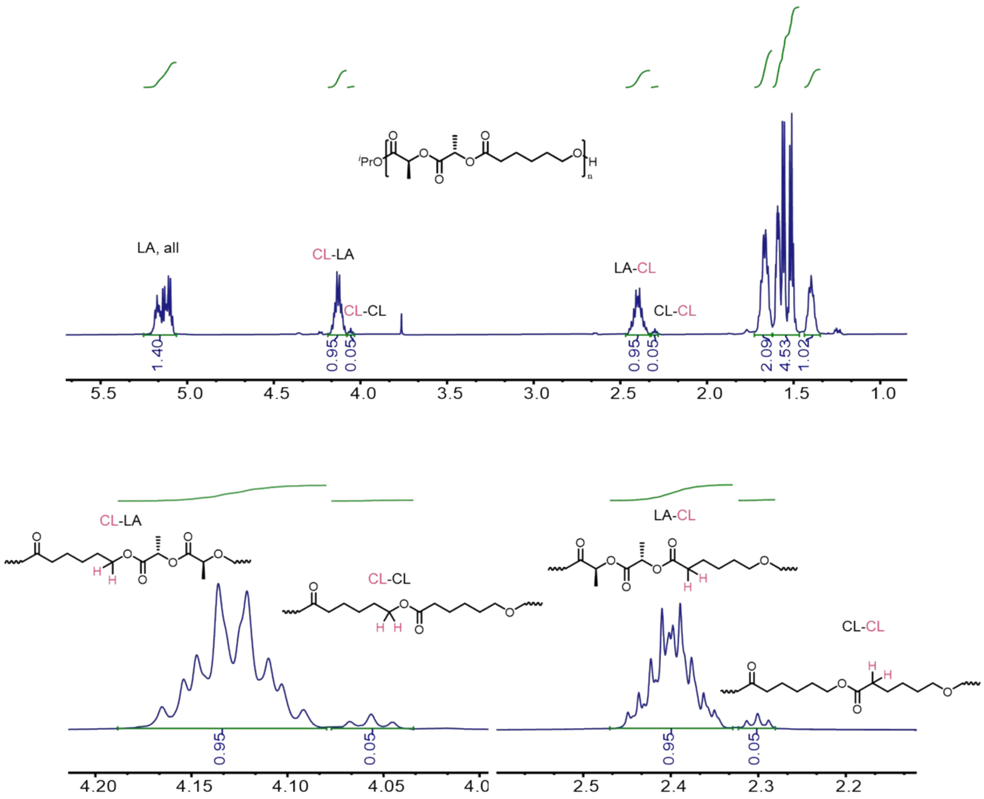

Figure S1. ^{1}H NMR spectrum of poly(*L*-LA-*alt*-CL) (Table S1, entry 1). Signal were assigned according to literature precedent.[3] The probabilities were determined using the equations $P_{CL\text{-}LA} = I_{CL\text{-}LA}/(I_{CL\text{-}LA}+I_{CL\text{-}CL})$ and $P_{LA\text{-}CL} = I_{LA\text{-}CL}/I_{LA,\ all}$ ($I_{LA,\ all} = I_{LA\text{-}LA} + I_{LA\text{-}CL}$, $I_{LA\text{-}CL} = I_{LA\text{-}CL}$).

Table S2. Results for the polymerization of *rac*-LA [a].

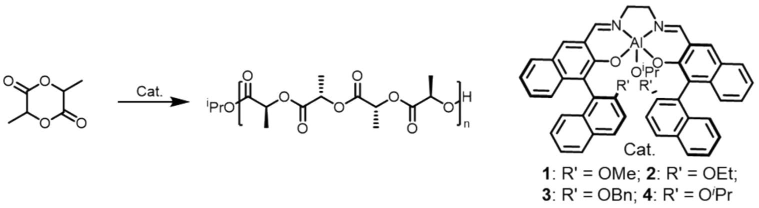

Entry	Catalyst	Time (h)	Conv. [b] (%)	$M_{n,obsd}$ [c]	$Đ$ [c]	P_r [d]

1	*R, R*-**1**	18	96	14.2	1.09	0.93
2	*R, R*-**2**	18	90	13.0	1.07	0.93
3	*R, R*-**3**	18	94	13.7	1.11	0.92
4	*R, R*-**4**	18	93	12.7	1.06	0.92

[a] General conditions: [*rac*-LA]$_0$:[Cat.]$_0$ = 100:1; performed in toluene with [*rac*-LA]$_0$ = 1.0 M, under a dry argon atmosphere at 70°C. [b] Monomer conversion was determined by integrating selected monomer and polymer signals in the ^{1}H NMR spectrum. [c] Experimental M_n and $Đ$ determined by gel permeation chromatography (GPC) in THF calibrated with standard polystyrene samples, and $M_{n,obsd}$ of PLA was corrected using a Mark–Houwink factor of 0.58. [d] PLA regularity was determined by analyzing all tetrad signals in the methine region of the homonuclear-decoupled ^{1}H NMR spectrum.

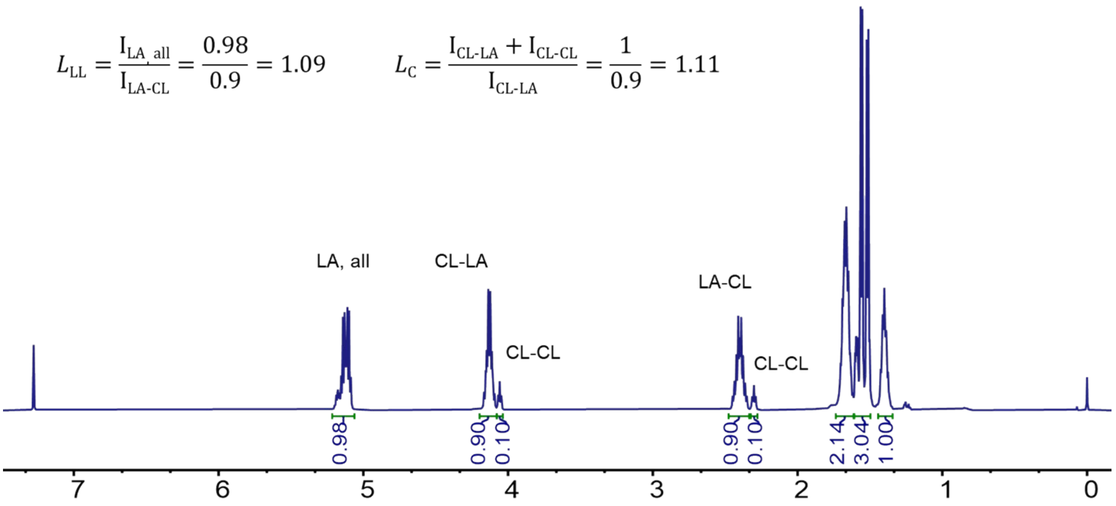

Figure S2. Calculation of number-average sequence lengths for poly(*L*-LA-*alt*-CL) (Table 1, entry 6) using ^{1}H NMR spectroscopy (600 MHz, $CDCl_3$).

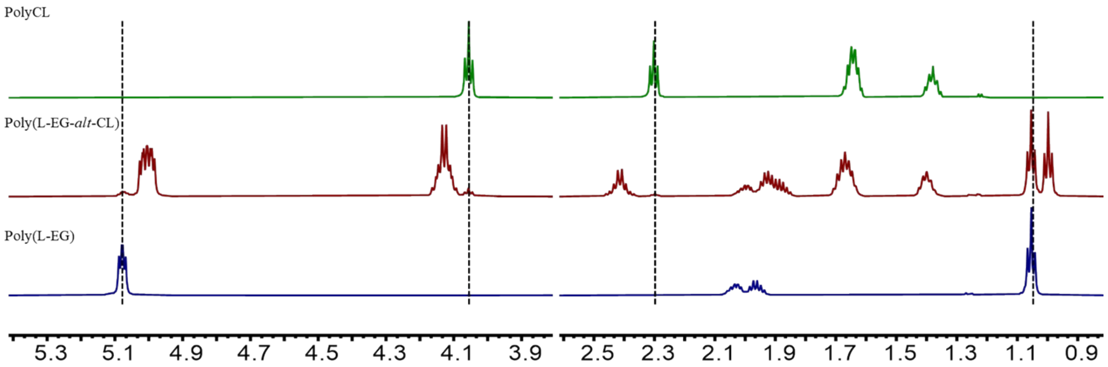

Figure S3. Stacked ^{1}H NMR spectra (600 MHz, $CDCl_3$) comparing the homopolymers polyCL and poly(*L*-EG) with the alternating copolymer poly(*L*-EG-*alt*-CL) (Table 1, entry 12).

Table S3. Raw kinetic data for CL homopolymerization, *L*-EG/CL copolymerization, and *L*-EG homopolymerization catalyzed by **4**.

Entry	*R, R*-**4** + CL		*R, R*-**4** + CL + *L*-EG		*R, R*-**4** + *L*-EG		*S, S*-**4** + *L*-EG	
	t (h)	Conv. (%)	*t* (h)	$Conv._{EG}$ (%)	*t* (h)	Conv. (%)	*t* (h)	Conv. (%)
1	0.17	6.62	5.15	20.12	24.00	5.84	10.00	7.37
2	0.33	22.80	10.15	31.38	45.00	8.85	22.00	13.92
3	0.50	44.50	22.15	56.18	69.00	11.78	34.00	19.31
4	0.67	58.95	32.15	72.15	94.00	15.28	49.00	27.55
5	1.00	77.76	46.15	83.44	116.00	18.22	59.50	31.60
6	1.67	93.12	54.15	86.97	141.00	20.61		

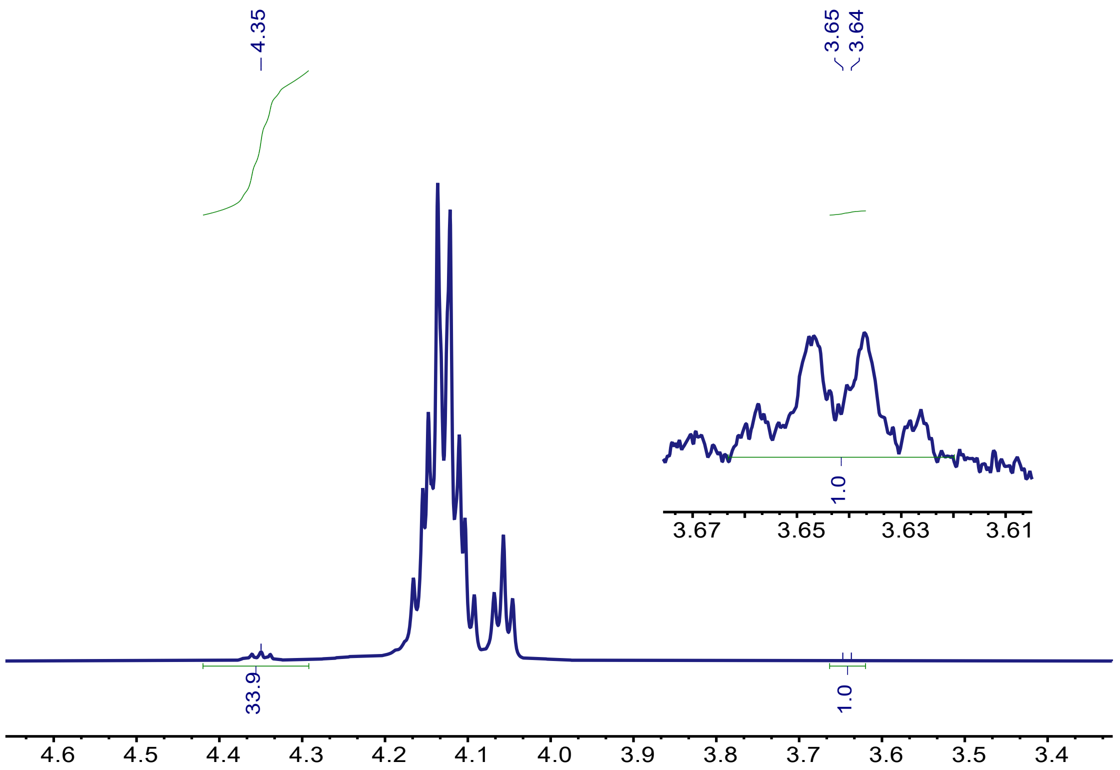

Figure S4. ^{1}H NMR spectrum (chain-end region) of poly(*L*-LA-*alt*-CL) prepared with *R, R*-**2** ($[LA]_0$:$[CL]_0$:$[Cat.]_0$ = 50:200:1).

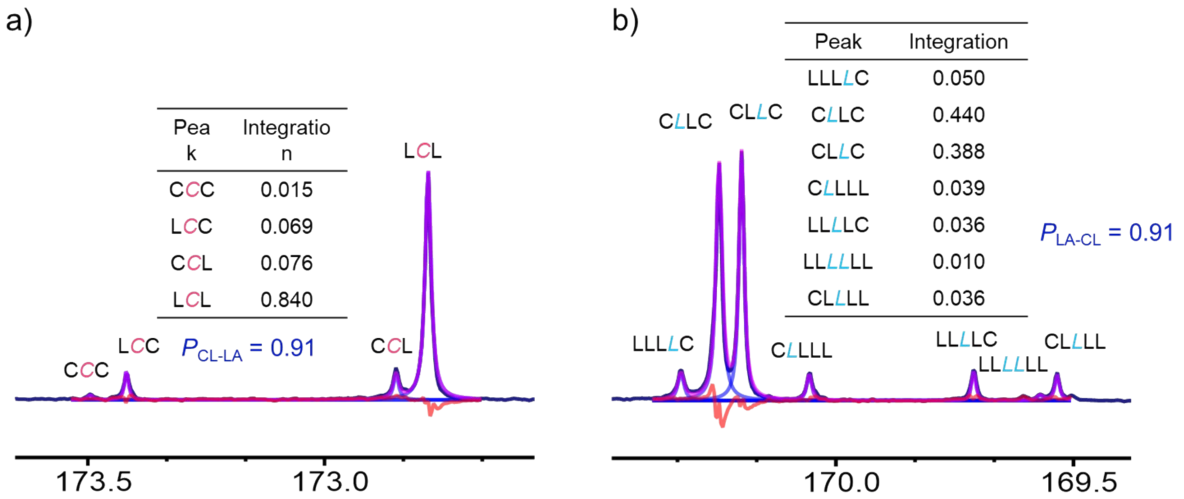

Figure S5. Simulation of the probability of alternating linkages between two monomers using signals of ^{13}C NMR spectra of poly(*L*-LA-*alt*-CL) catalyzed by *R, R*-**2** (P_{alt} = 0.91). a) from the carbonyl region of CL units, the probabilities were determined using the equations $P_{CL-LA} = I_{CCL} + I_{LCL}$. b) from the carbonyl region of LA units, the equations used were $P_{LA-CL} = I_{CLLC} + I_{CLLC} + I_{LLLLC} + I_{LLLLC}$.

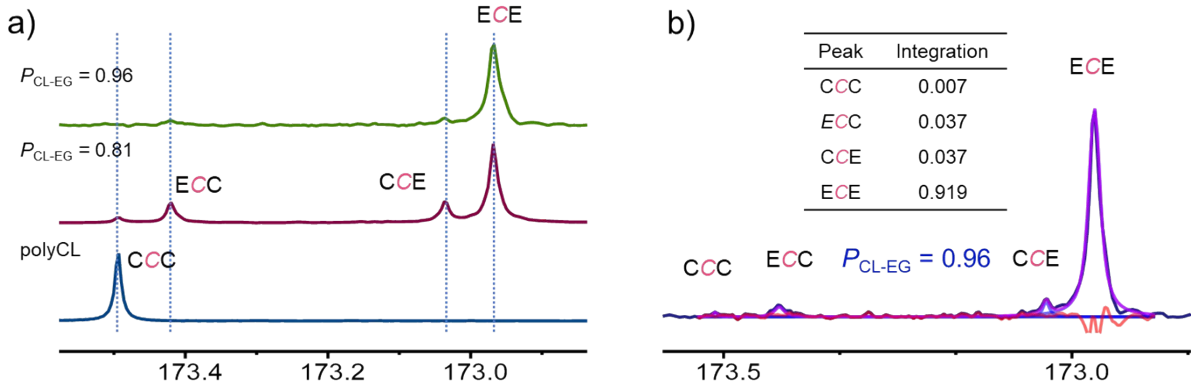

Figure S6. ^{13}C NMR analysis for determining P_{CL-EG} in poly(*L*-EG-*alt*-CL). a) Comparison of the relevant partial ^{13}C NMR spectra. b) Peak deconvolution and integration analysis of the CL carbonyl region of poly(*L*-EG-*alt*-CL) synthesized by *R, R*-**4**. Based on the integrations of the CL-centered sequence signals, P_{CL-EG} was calculated to be 0.96.

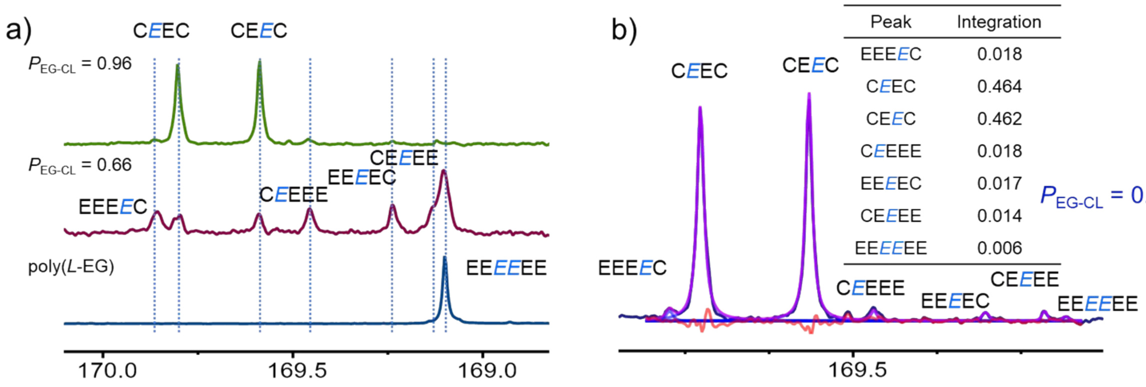

Figure S7. ^{13}C NMR analysis for determining $P_{EG–CL}$ in poly(*L*-EG-*alt*-CL). a) Comparison of the relevant partial ^{13}C NMR spectra. b) Peak deconvolution and integration analysis of the EG carbonyl region of

poly(*L*-EG-*alt*-CL) synthesized by *R, R*-**4**. Based on the integrations of the EG-centered sequence signals, $P_{\text{EG-CL}}$ was calculated to be 0.96.

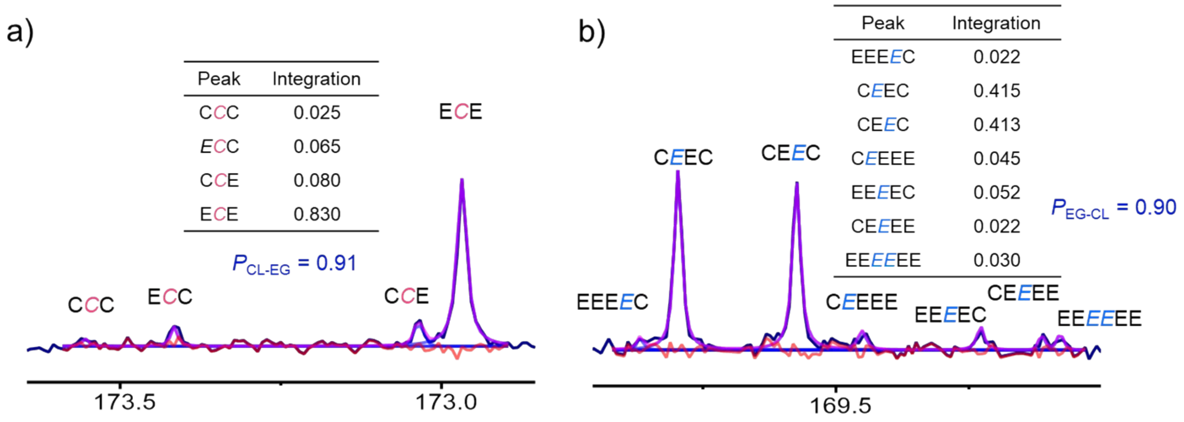

Figure S8. Simulation of the probability of alternating linkages between two monomers using signals of ^{13}C NMR spectra of poly(*L*-EG-*alt*-CL) catalyzed by *R, R*-**1**. a) from the carbonyl region of CL units, the probabilities were determined using the equations $P_{\text{CL-EG}} = I_{C\mathit{C}E} + I_{E\mathit{C}E}$. b) from the carbonyl region of EG units, the equations used were $P_{\text{EG-CL}} = I_{C\mathit{E}EC} + I_{CE\mathit{E}C} + I_{EEE\mathit{E}C} + I_{EE\mathit{E}EC}$.

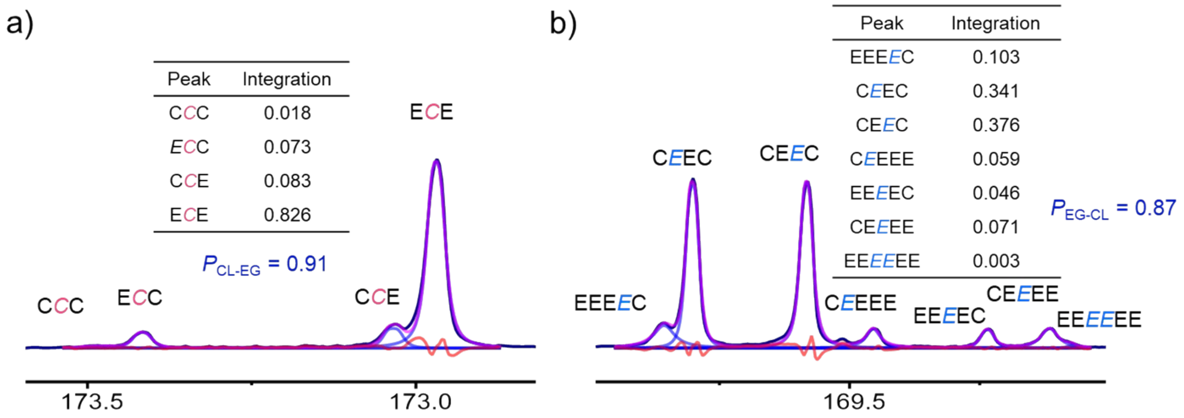

Figure S9. Simulation of the probability of alternating linkages between two monomers using signals of ^{13}C NMR spectra of poly(*L*-EG-*alt*-CL) catalyzed by *R, R*-**2**. a) from the carbonyl region of CL units, the probabilities were determined using the equations $P_{\text{CL-EG}} = I_{C\mathit{C}E} + I_{E\mathit{C}E}$. b) from the carbonyl region of EG units, the equations used were $P_{\text{EG-CL}} = I_{C\mathit{E}EC} + I_{CE\mathit{E}C} + I_{EEE\mathit{E}C} + I_{EE\mathit{E}EC}$.

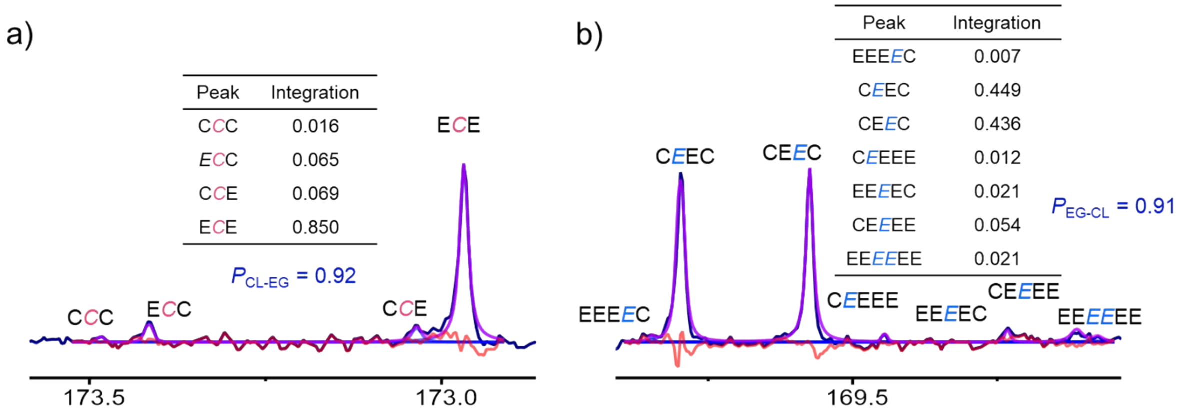

Figure S10. Simulation of the probability of alternating linkages between two monomers using signals of ^{13}C NMR spectra of poly(*L*-EG-*alt*-CL) catalyzed by *R, R*-**3**. a) from the carbonyl region of CL units, the

probabilities were determined using the equations $P_{\text{CL-EG}} = \text{I}_{\text{C}C\text{E}} + \text{I}_{\text{E}C\text{E}}$. b) from the carbonyl region of EG units, the equations used were $P_{\text{EG-CL}} = \text{I}_{\text{C}E\text{EC}} + \text{I}_{\text{CE}E\text{C}} + \text{I}_{\text{EEE}E\text{C}} + \text{I}_{\text{EE}E\text{EC}}$.

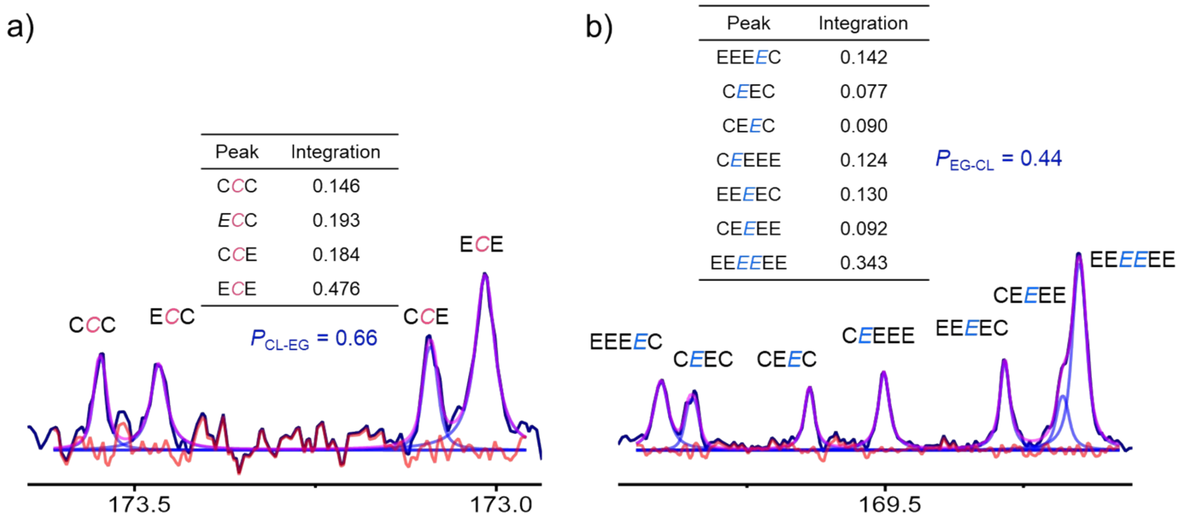

Figure S11. Simulation of the probability of alternating linkages between two monomers using signals of ^{13}C NMR spectra of poly(*L*-EG-*alt*-CL) catalyzed by *S, S*-**4**. a) from the carbonyl region of CL units, the probabilities were determined using the equations $P_{\text{CL-EG}} = \text{I}_{\text{C}C\text{E}} + \text{I}_{\text{E}C\text{E}}$. b) from the carbonyl region of EG units, the equations used were $P_{\text{EG-CL}} = \text{I}_{\text{C}E\text{EC}} + \text{I}_{\text{CE}E\text{C}} + \text{I}_{\text{EEE}E\text{C}} + \text{I}_{\text{EE}E\text{EC}}$.

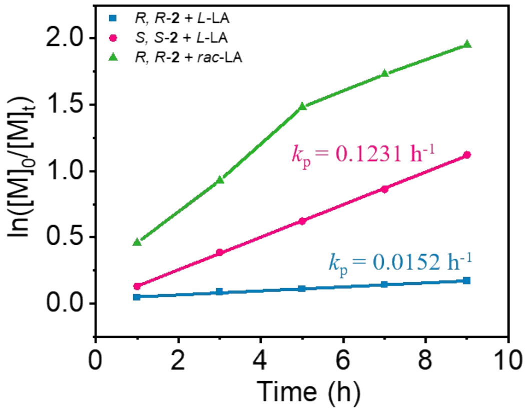

Figure S12. Control homopolymerization experiments for evaluating stereochemical effects in lactide polymerization. Conditions: $[\text{M}]_0 = 0.5$ M, $[\text{Cat.}]_0 = 0.005$ M. Apparent first-order kinetic plots for *L*-LA homopolymerization catalyzed by *R, R*-**2** and *S, S*-**2** are shown, together with the *rac*-LA homopolymerization profile catalyzed by *R, R*-**2** for comparison. The apparent propagation rate constants for *L*-LA homopolymerization were $k_p = 0.0152$ h^{-1} and 0.1231 h^{-1}, with $R^2 = 0.995$ and 0.999 for *R, R*-**2** and *S, S*-**2**, respectively. The *rac*-LA polymerization with *R, R*-**2** proceeded more rapidly than *L*-LA polymerization with either *R, R*-**2** or *S, S*-**2**.

Table S4. Raw kinetic data for lactide homopolymerizations catalyzed by **2**.

Entry	*t* (h)	*R, R*-**2** + *L*-LA	*S, S*-**2** + *L*-LA	*R, R*-**2** + *rac*-LA
		Conv. (%)	Conv. (%)	Conv. (%)
1	1	4.60	12.20	36.75

2	3	8.30	32.03	60.50
3	5	10.36	46.28	77.27
4	7	13.26	57.79	82.27
5	9	15.73	67.45	85.79

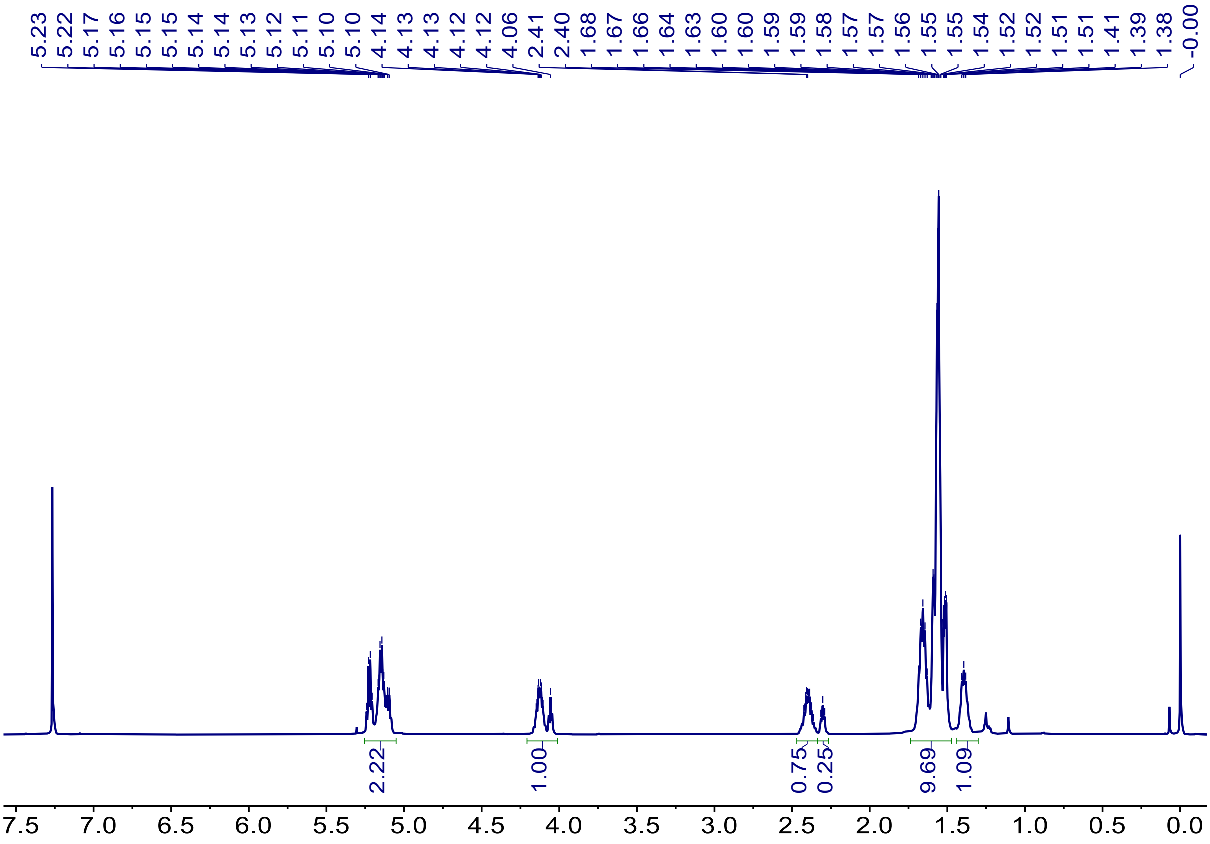

Figure S13. ^{1}H NMR spectrum of the copolymerization product from the *rac*-LA/CL control experiment catalyzed by *R, R*-**2**. Conditions: bulk polymerization, 70 °C, 12.5 h, [*rac*-LA]$_0$/[CL]$_0$/[Cat.]$_0$ = 100/400/1. The spectrum indicates preferential LA incorporation over CL incorporation, with the molar ratio of LA units to CL units in the resulting copolymer reaching 2.22:1.

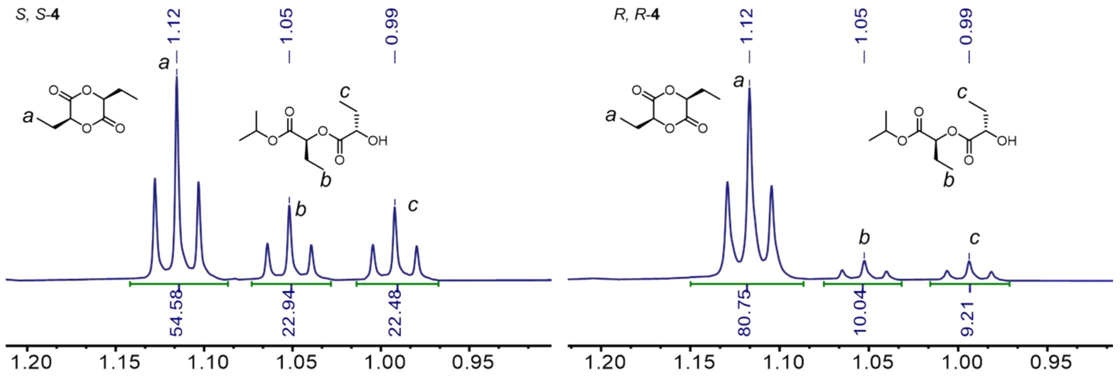

Figure S14. ^{1}H NMR spectra (methyl region) from the model reaction of isopropanol-initiated ring-opening of *L*-ethylglycolide (*L*-EG) ([^{i}PrOH]$_0$:[*L*-EG]$_0$:[Cat.]$_0$ = 4:4:1, [Cat.]$_0$ = 0.025 M).

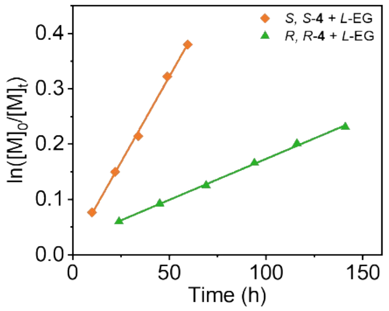

Figure S15. First-order kinetic plots for the polymerizations of *L*-EG. *S, S*-**4** (◆): $k_{app(EG-EG)}^{S,S} = 0.0062\ h^{-1}$, R^2 = 0.998. *R, R*-**4** (▲): $k_{app(EG-EG)}^{R,R} = 0.0015\ h^{-1}$, R^2 = 0.998.

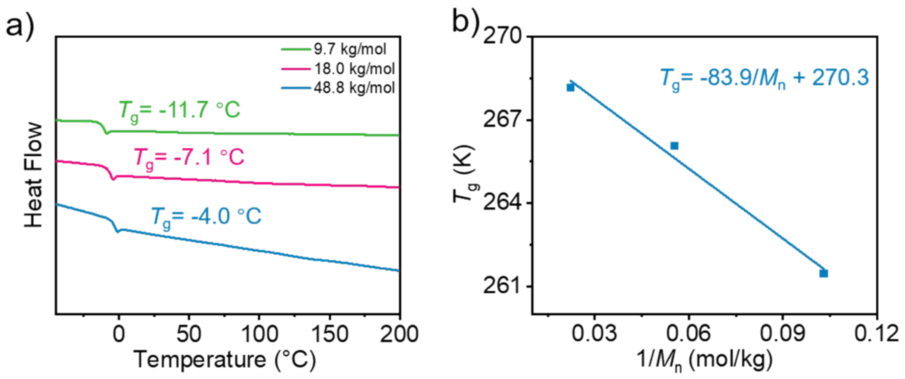

Figure S16. Determination of the glass transition temperature at infinite molecular weight ($T_{g,\infty}$) for poly(*L*-LA-*alt*-CL). a) Stacked DSC thermograms from the second heating scan for samples with M_n values of 9.7, 18.0, and 44.8 kg/mol. b) Linear regression of T_g versus 1/M_n based on the Flory-Fox equation ($T_g = T_{g,\infty} - k/M_n$). The y-intercept indicates a $T_{g,\infty}$ of -2.9 °C (270.3 K).

Table S5. Glass transition temperatures (T_g) of poly(*L*-LA-*random*-CL) copolymers reported in the literature.

Entry	[*L*-LA]$_0$:[CL]$_0$:[I]$_0$	LA/CL in copolymer	M_n (kg/mol)	T_g (°C)	Ref
1	100:100:1	50/50	-	-9.5	[4]
2	50:50:1	43/57	8.0	-17	[5]
3	150:150:4	52/48	5.9	-17.2	[6]

4	100:100:1	45/55	49	-5.5	7
5	100:200:1	81/19	54.9	26	8
6	50:50:1	52/48	3.5	8	9
7	50:50:1	50/50	12.8	-19.5	10
8	100:100:5	59/41	6.7	-6	11

Table S6. Molecular weights, compositions, and synthetic conditions of the copolymer samples used for hydrolytic degradation experiments.

Sample	$M_{n,0}$ (kg/mol)	*Đ*	LA/CL in copolymer
poly(*L*-LA-*alt*-CL)	31.5	1.06	48/52
poly(*L*-LA-*stat*-CL)	30.6	1.37	44/56

Note: poly(*L*-LA-*alt*-CL) was synthesized in toluene at 70 °C using the *R, R*-**2** catalyst; poly(*L*-LA-*stat*-CL) was synthesized according to a literature procedure (*Angew. Chem. Int. Ed.* **2008**, *47*, 9088–9091) in THF at 80 °C using the (*R*)-SalenAl-O^{i}Pr catalyst.

Table S7. Changes in molecular weight of poly(*L*-LA-*alt*-CL) and poly(*L*-LA-*stat*-CL) during hydrolytic degradation.

Time (d)	poly(*L*-LA-*alt*-CL)						poly(*L*-LA-*stat*-CL)					
	$M_{n,t}$	*Đ*	$M_{n,t}$	*Đ*	$M_{n,t}$	*Đ*	$M_{n,t}$	*Đ*	$M_{n,t}$	*Đ*	$M_{n,t}$	*Đ*
1	30.1	1.08	31.6	1.07	31.2	1.08	30.4	1.24	30.7	1.23	29.3	1.27
7	28.3	1.07	29.0	1.07	27.3	1.08	23.8	1.41	24.9	1.40	23.0	1.43
9.19	27.5	1.13	28.5	1.11	26.7	1.13	21.6	1.67	20.2	1.69	22.9	1.65
12	27.4	1.13	25.8	1.14	27.3	1.13	18.6	1.48	19.8	1.45	17.3	1.50
14.26	24.5	1.19	25.7	1.17	26.1	1.17	17.0	1.67	18.1	1.62	15.8	1.69
17.7	24.6	1.21	23.6	1.23	22.9	1.24	15.8	1.76	16.7	1.70	14.5	1.79
21.78	22.3	1.33	23.6	1.30	21.6	1.33	14.3	1.71	13.3	1.74	15.4	1.71
24.97	20.7	1.41	22.1	1.39	20.2	1.42	13.1	1.90	14.4	1.85	12.1	1.91
27.69	19.1	1.42	20.4	1.39	18.5	1.41	11.1	1.86	12.5	1.81	9.8	1.93
29.21	18.5	1.53	17.7	1.58	19.6	1.49	9.4	1.98	8.1	2.11	10.3	1.96

Note: $M_{n,t}$ is the number-average molecular weight of the sample after hydrolytic degradation for time t, expressed in kg/mol.

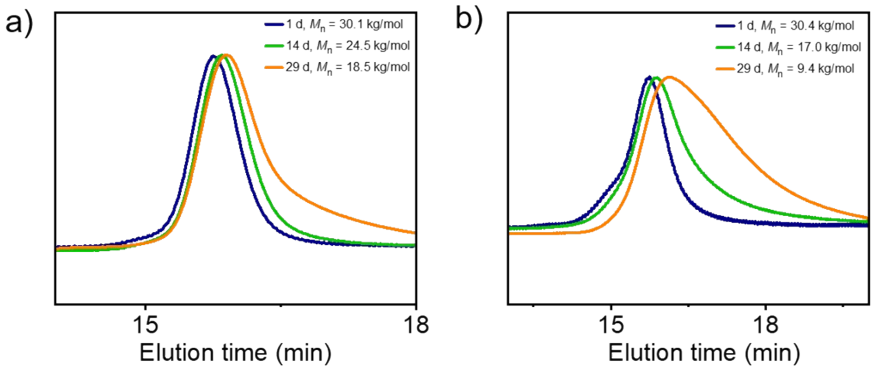

Figure S17. Representative GPC traces of poly(*L*-LA-*alt*-CL) and poly(*L*-LA-*stat*-CL) after hydrolytic degradation for different times. a) poly(*L*-LA-*alt*-CL). b) poly(*L*-LA-*stat*-CL).

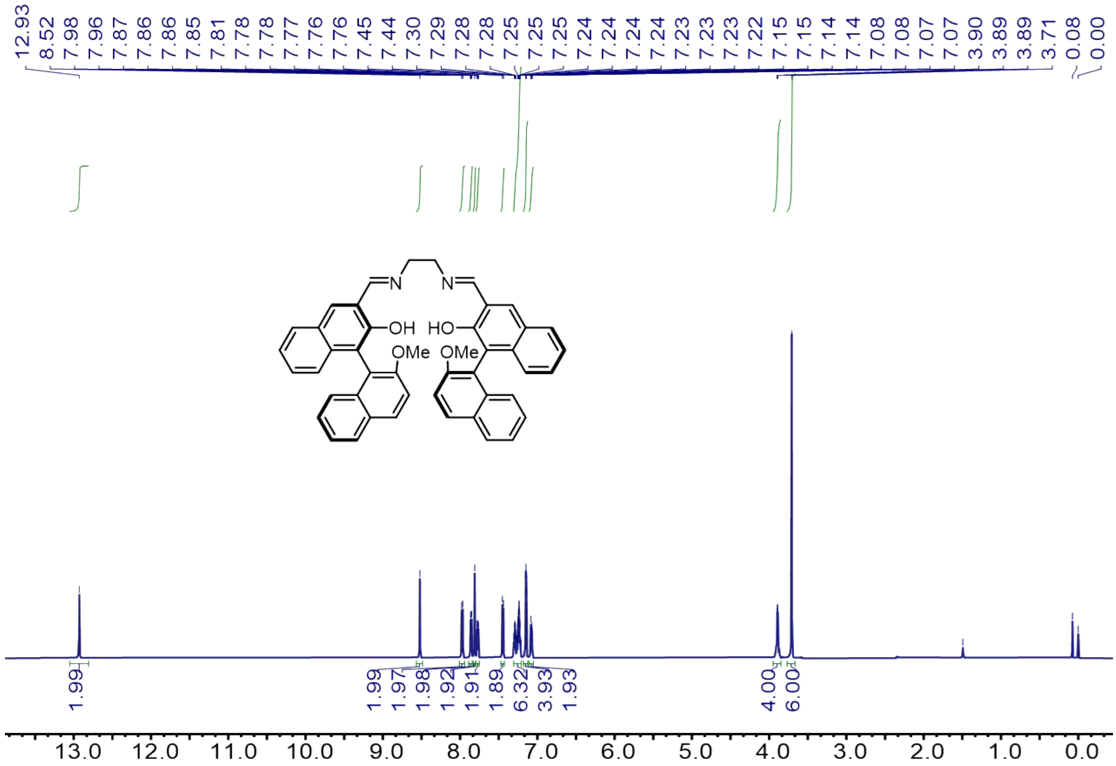

Figure S18. ^{1}H NMR spectra ($CDCl_3$, 600 MHz) of *R, R*-**1** ligand.

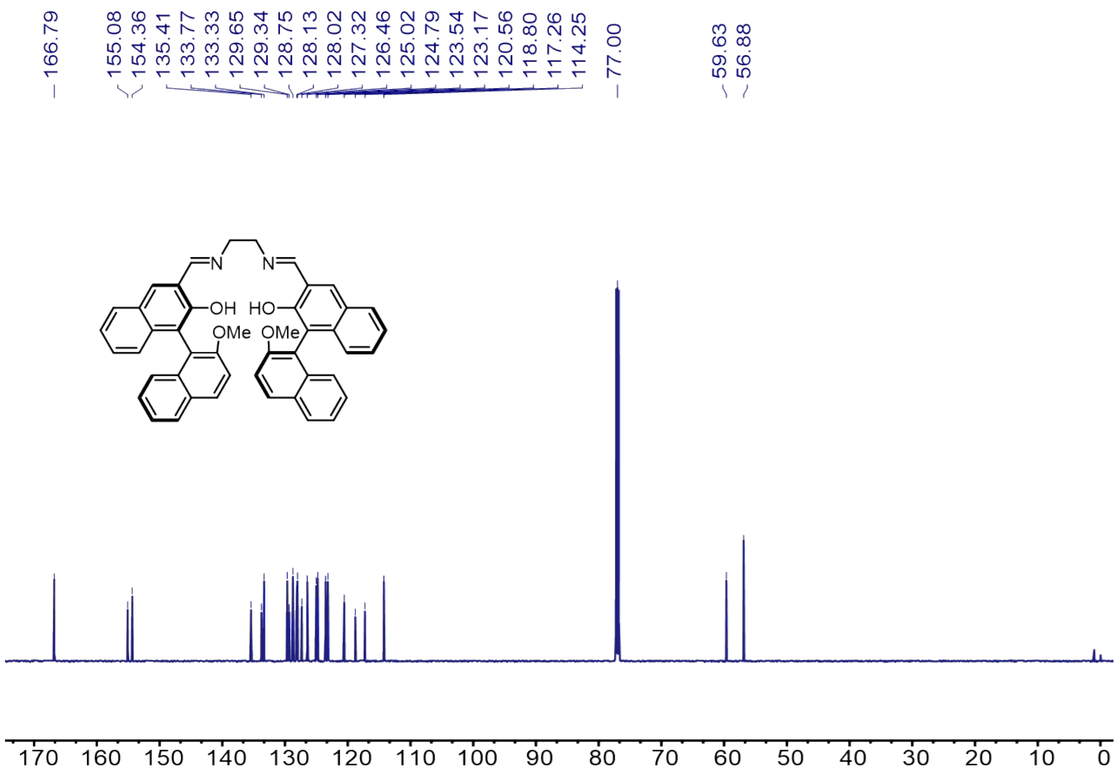

Figure S19. ^{13}C NMR spectra ($CDCl_3$, 151 MHz) of *R, R*-**1** ligand.

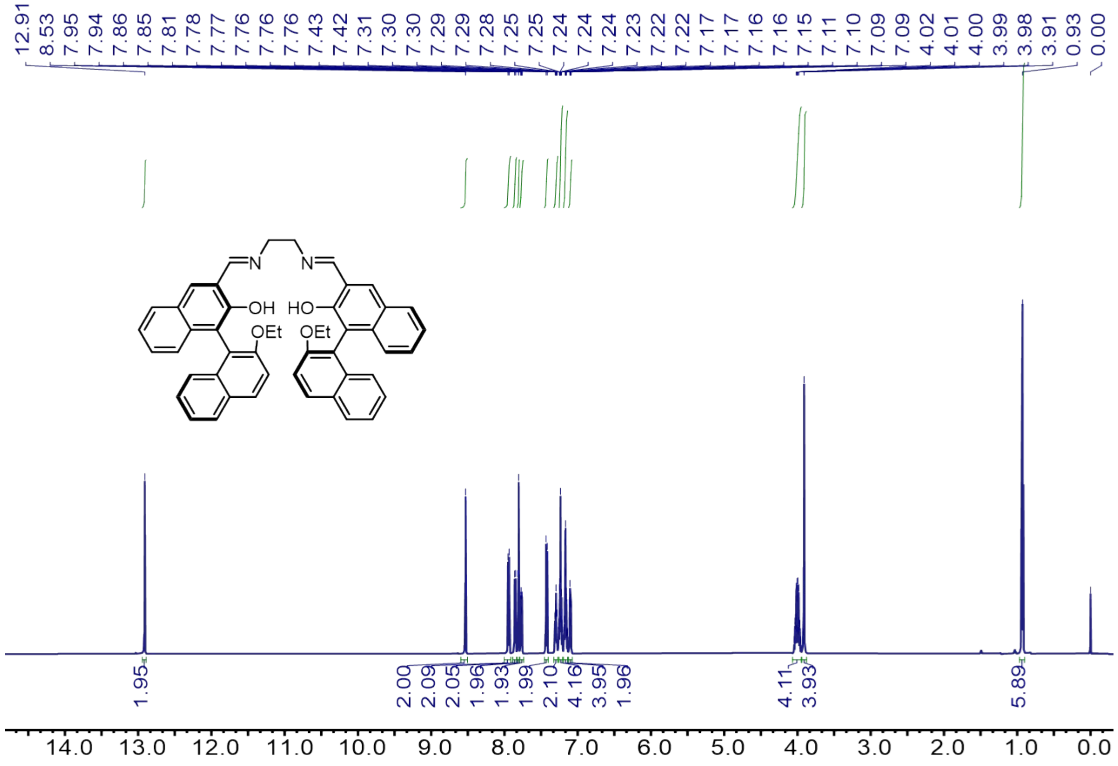

Figure S20. ^{1}H NMR spectra ($CDCl_3$, 600 MHz) of *R, R*-**2** ligand.

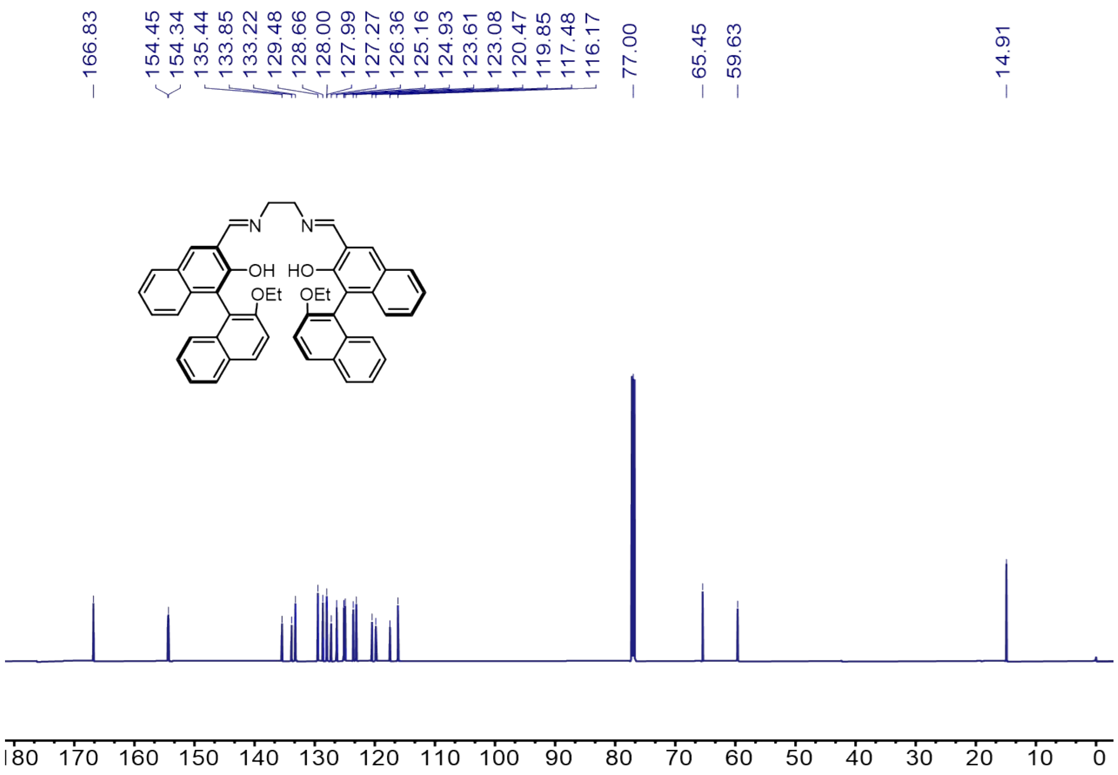

Figure S21. ^{13}C NMR spectra ($CDCl_3$, 151 MHz) of *R, R*-**2** ligand.

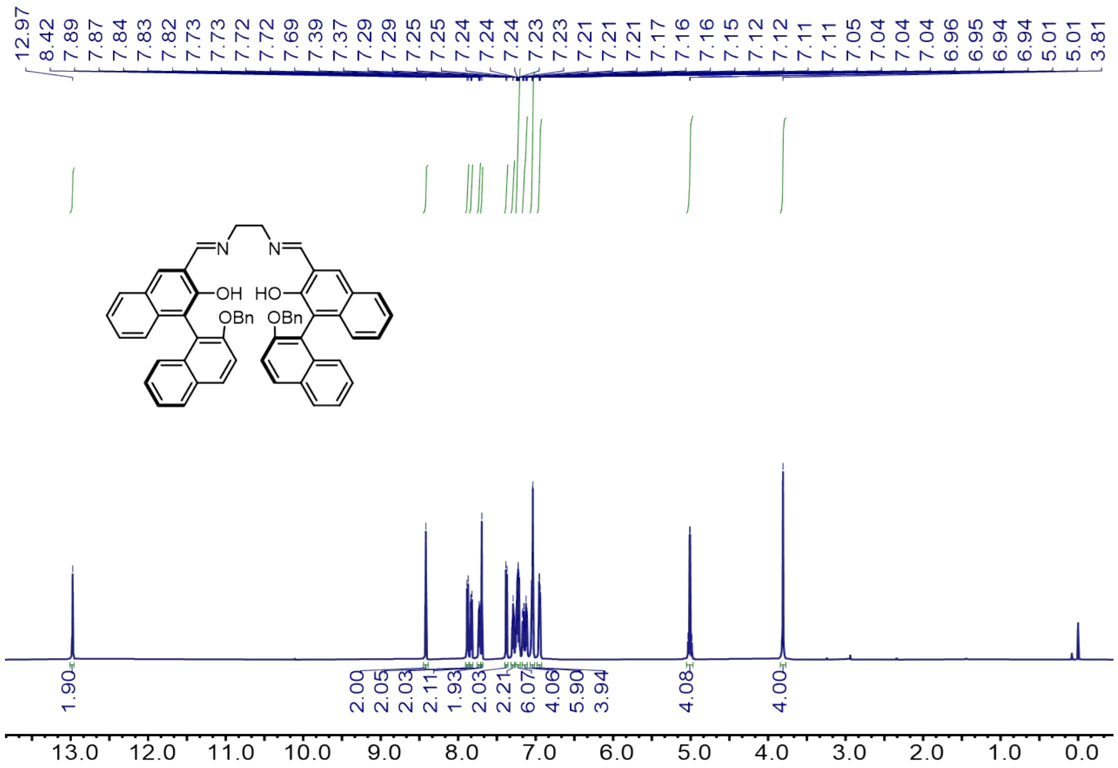

Figure S22. ^{1}H NMR spectra ($CDCl_3$, 600 MHz) of *R, R*-**3** ligand.

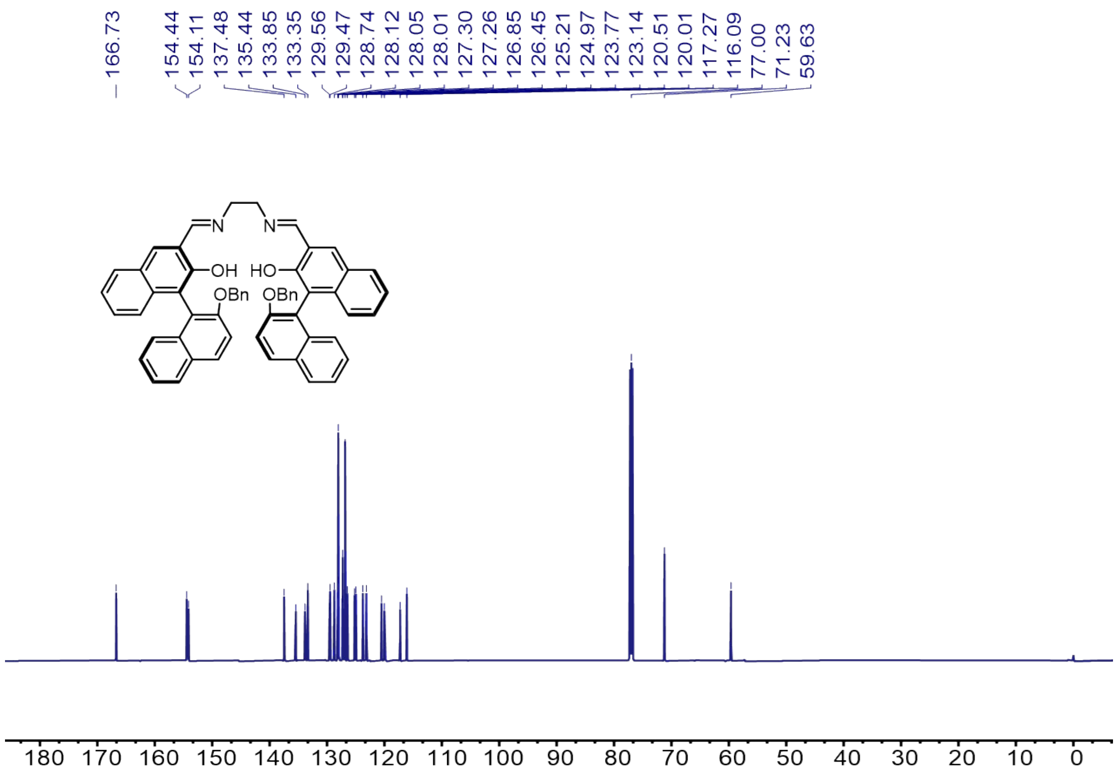

Figure S23. ^{13}C NMR spectra ($CDCl_3$, 151 MHz) of *R, R*-**3** ligand.

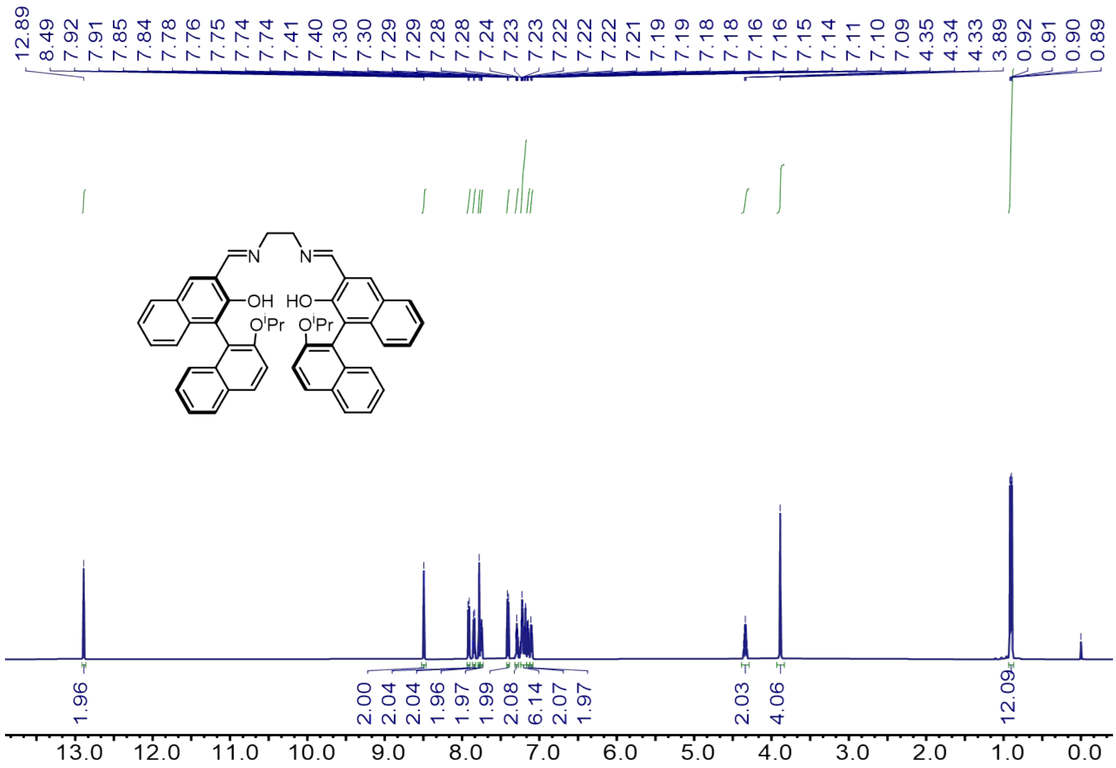

Figure S24. ^{1}H NMR spectra ($CDCl_3$, 600 MHz) of *R, R*-**4** ligand.

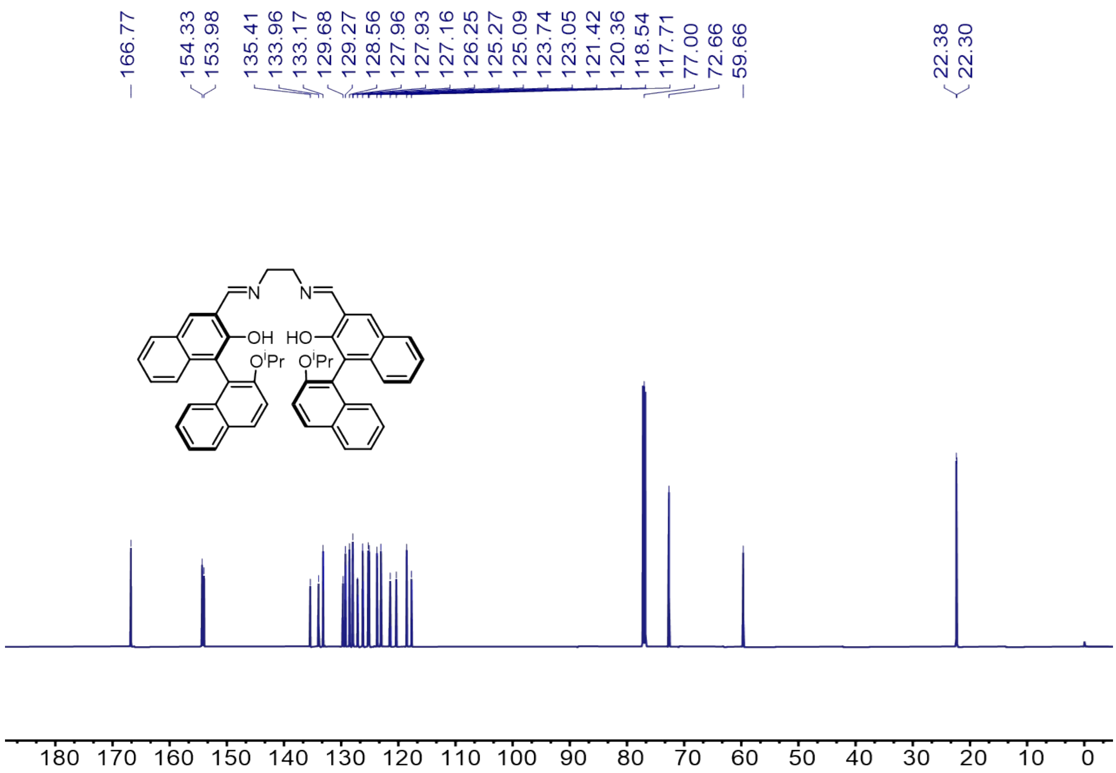

Figure S25. ^{13}C NMR spectra (CDCl$_3$, 151 MHz) of *R, R*-**4** ligand.

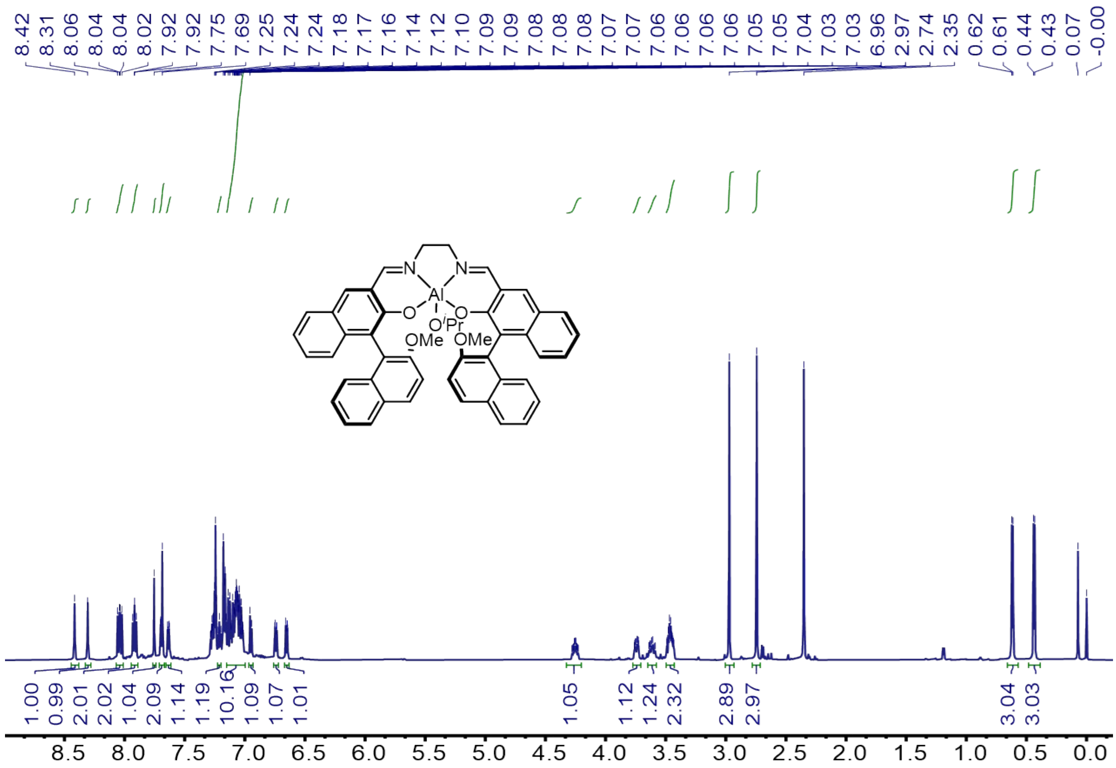

Figure S26. ^{1}H NMR spectra (CDCl$_3$, 600 MHz) of *R, R*-**1**

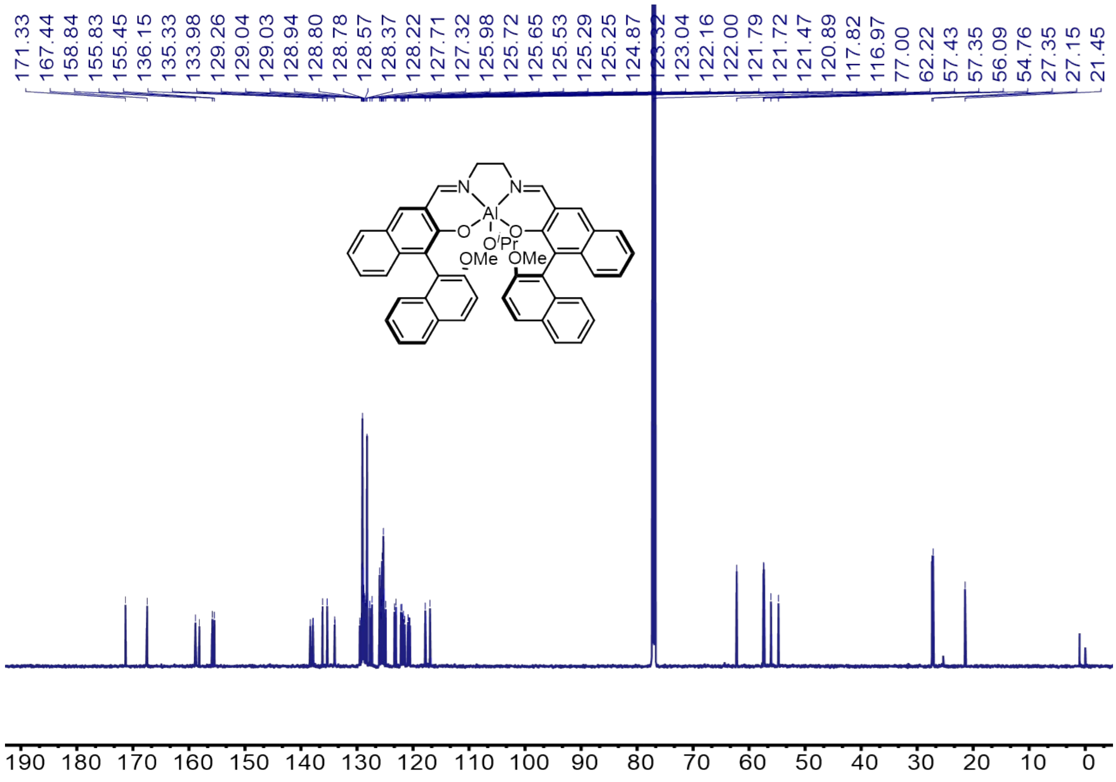

Figure S27. ^{13}C NMR spectra ($CDCl_3$, 151 MHz) of *R, R*-**1**.

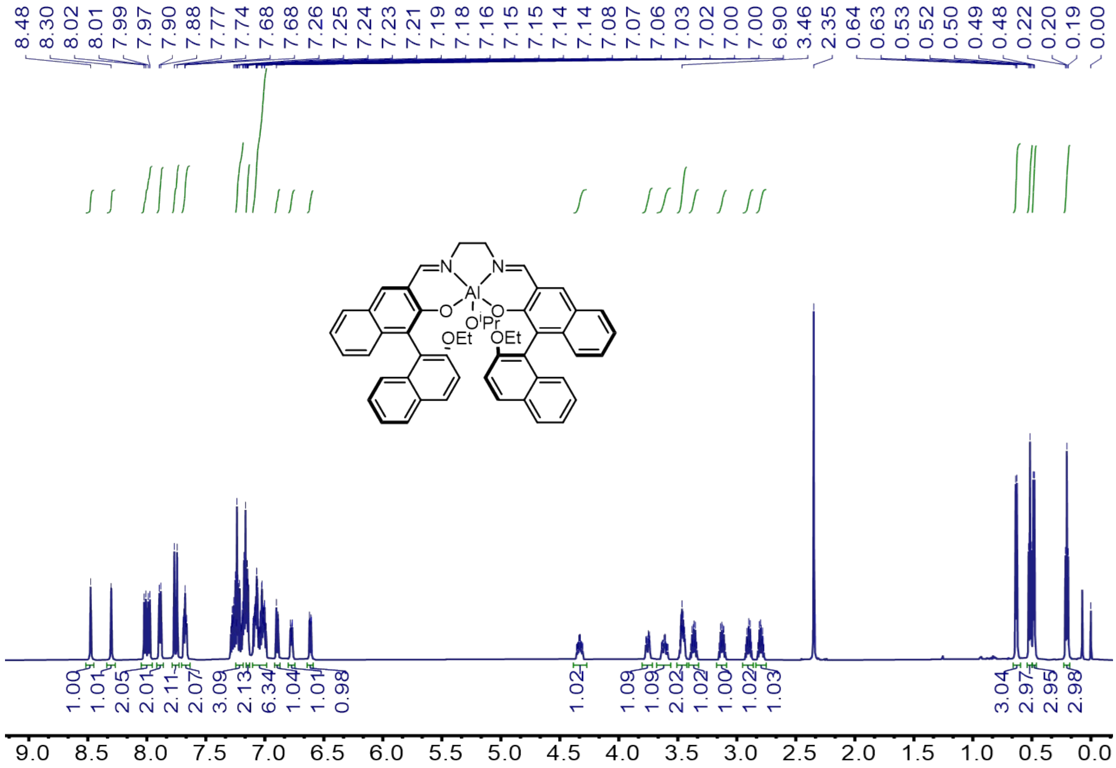

Figure S28. ^{1}H NMR spectra ($CDCl_3$, 600 MHz) of *R, R*-**2**.

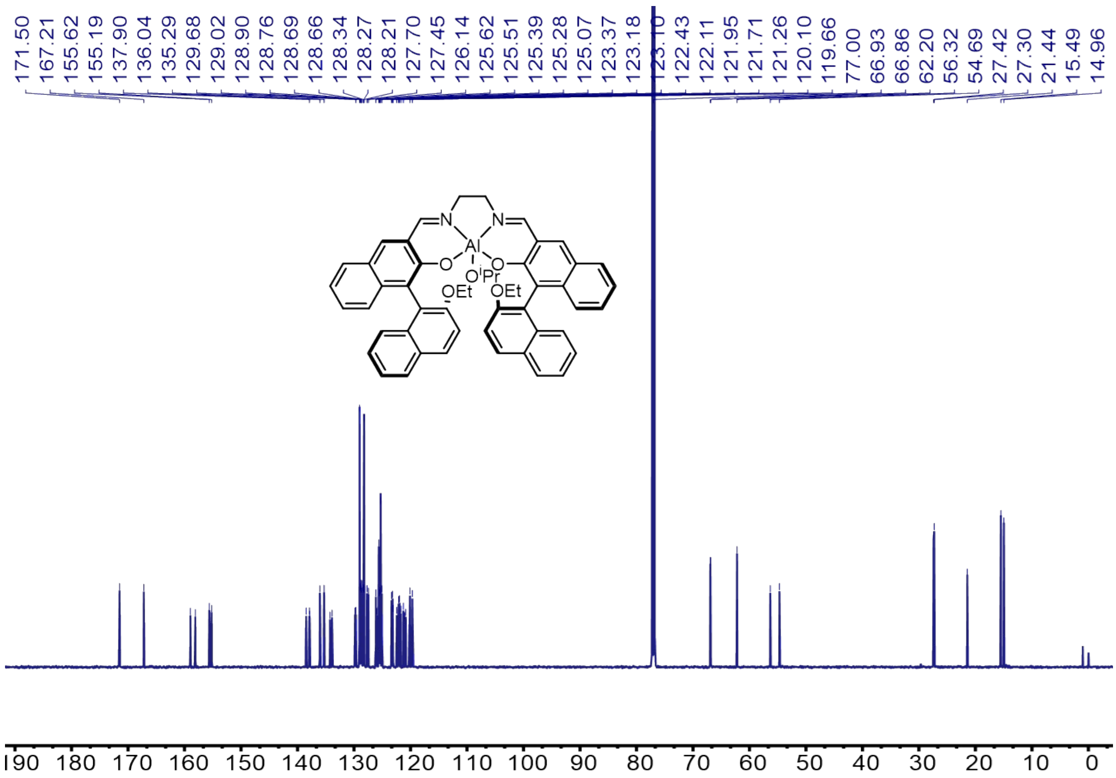

Figure S29. ^{13}C NMR spectra ($CDCl_3$, 151 MHz) of *R, R*-**2**.

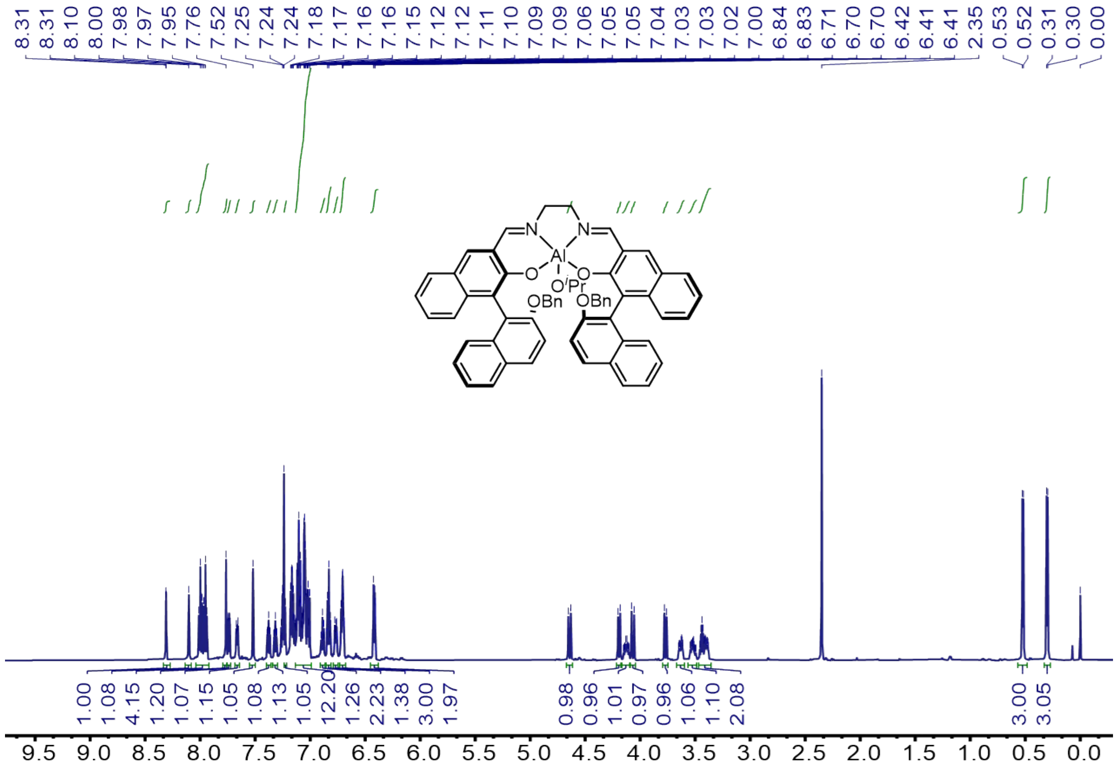

Figure S30. ^{1}H NMR spectra ($CDCl_3$, 600 MHz) of *R, R*-**3**.

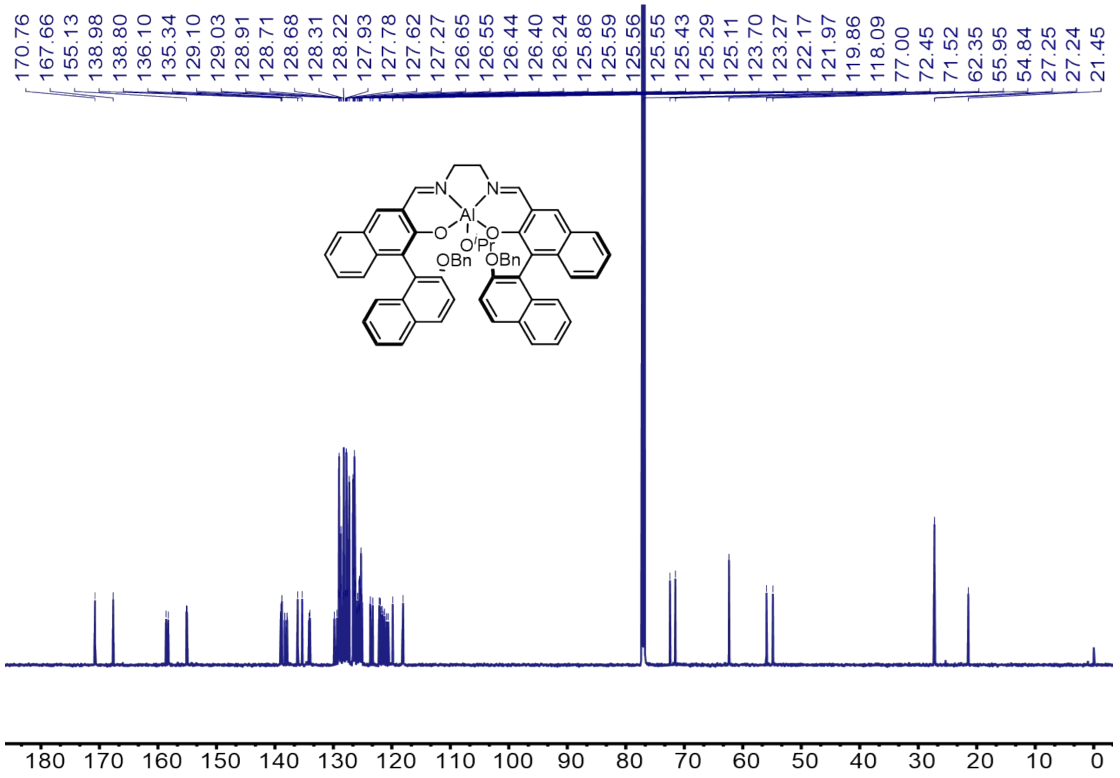

Figure S31. ^{13}C NMR spectra ($CDCl_3$, 151 MHz) of *R, R*-**3**.

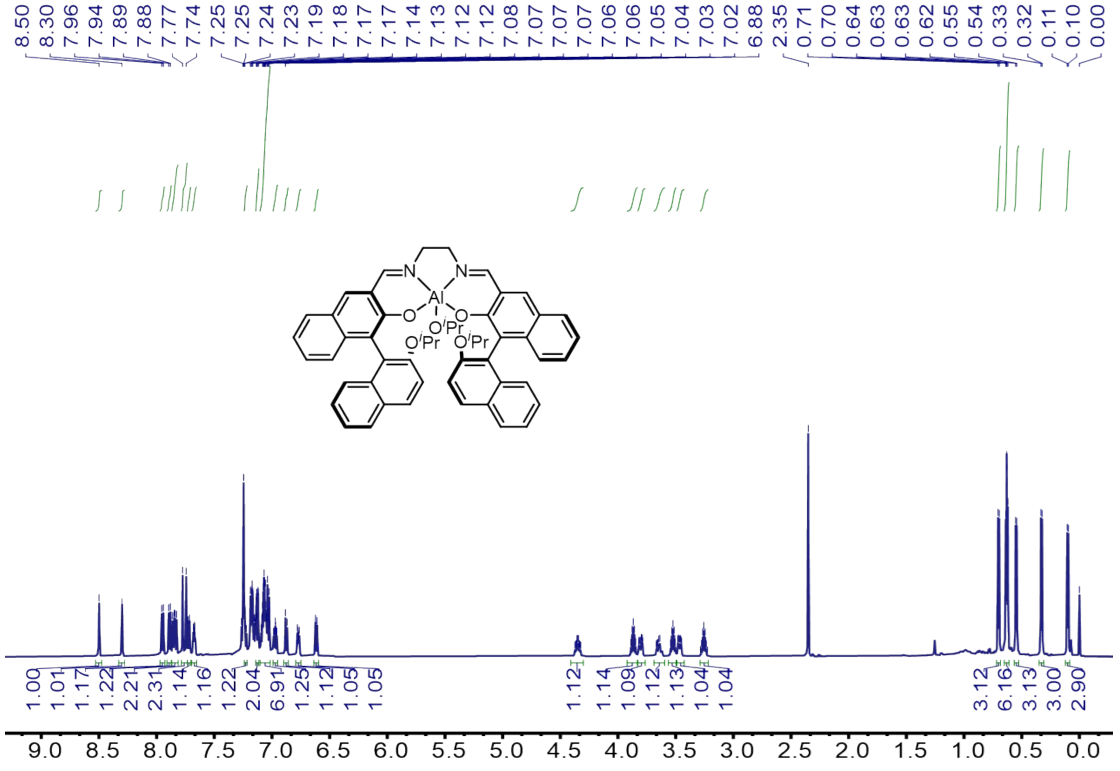

Figure S32. ^{1}H NMR spectra ($CDCl_3$, 600 MHz) of *R, R*-**4**.

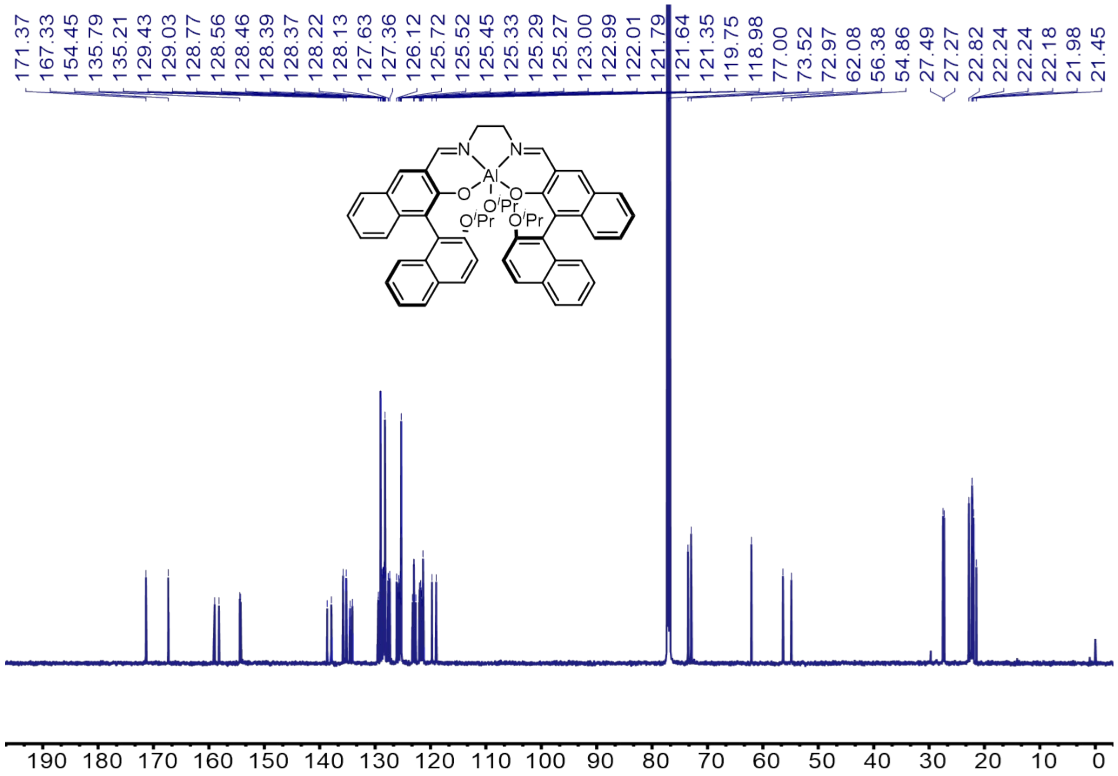

Figure S33. ^{13}C NMR spectra ($CDCl_3$, 151 MHz) of *R, R*-**4**.

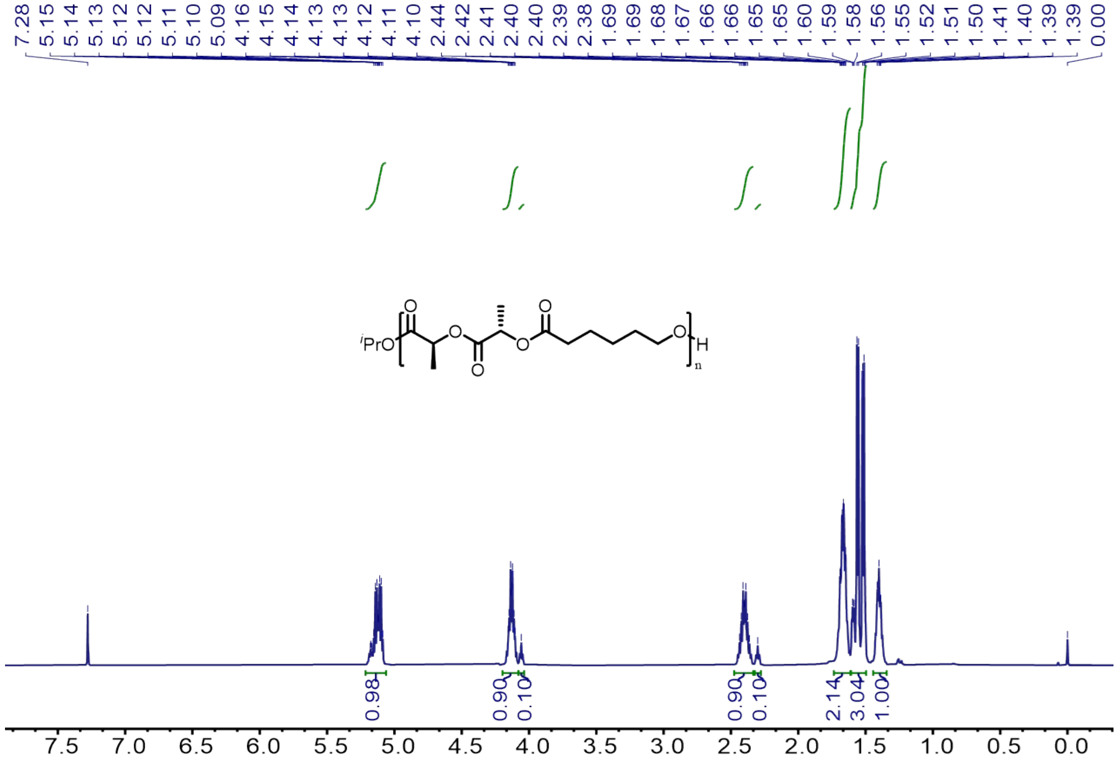

Figure S34. ^{1}H NMR spectra ($CDCl_3$, 600 MHz) of poly(*L*-LA-*alt*-CL) (P_{alt} = 0.91).

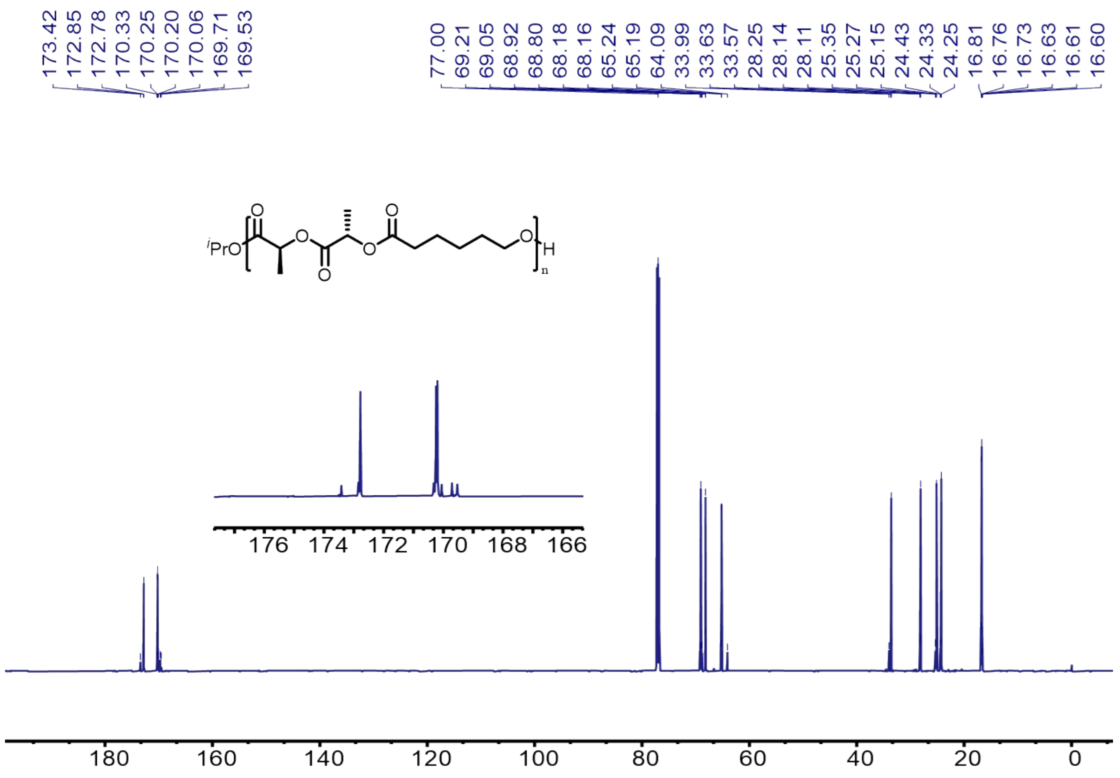

Figure S35. ^{13}C NMR spectra ($CDCl_3$, 151 MHz) of poly(*L*-LA-*alt*-CL) (P_{alt} = 0.91).

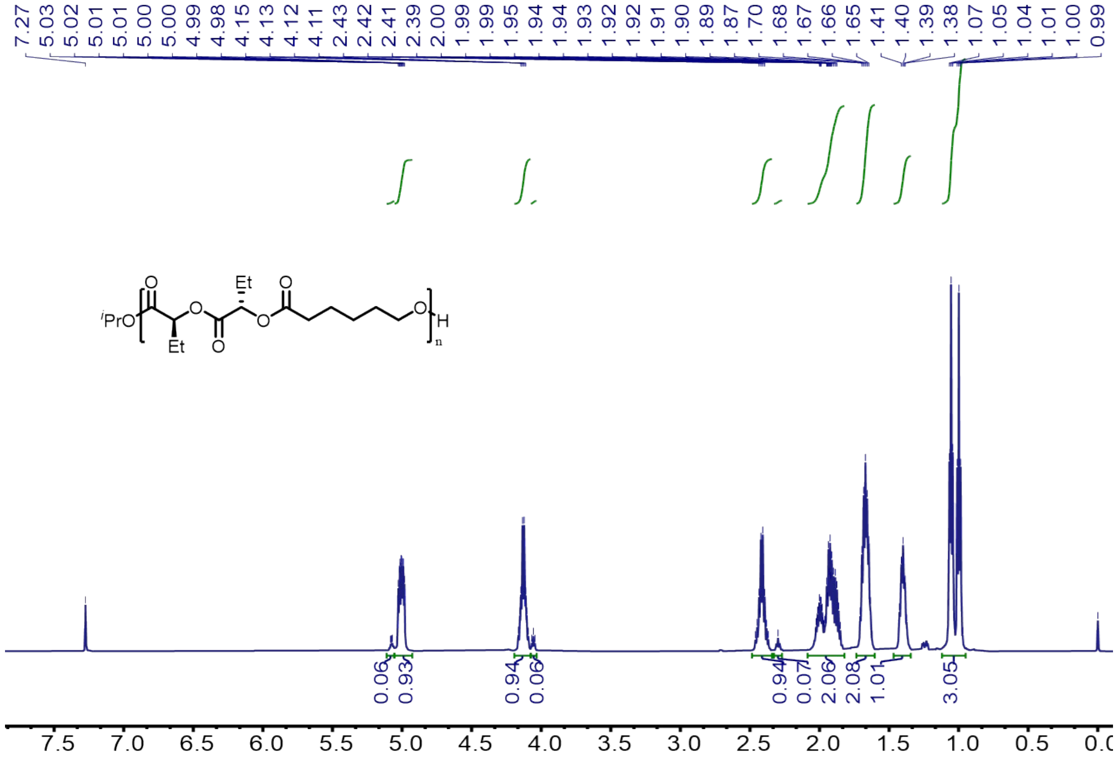

Figure S36. ^{1}H NMR spectra ($CDCl_3$, 600 MHz) of poly(*L*-EG-*alt*-CL) (P_{alt} = 0.94).

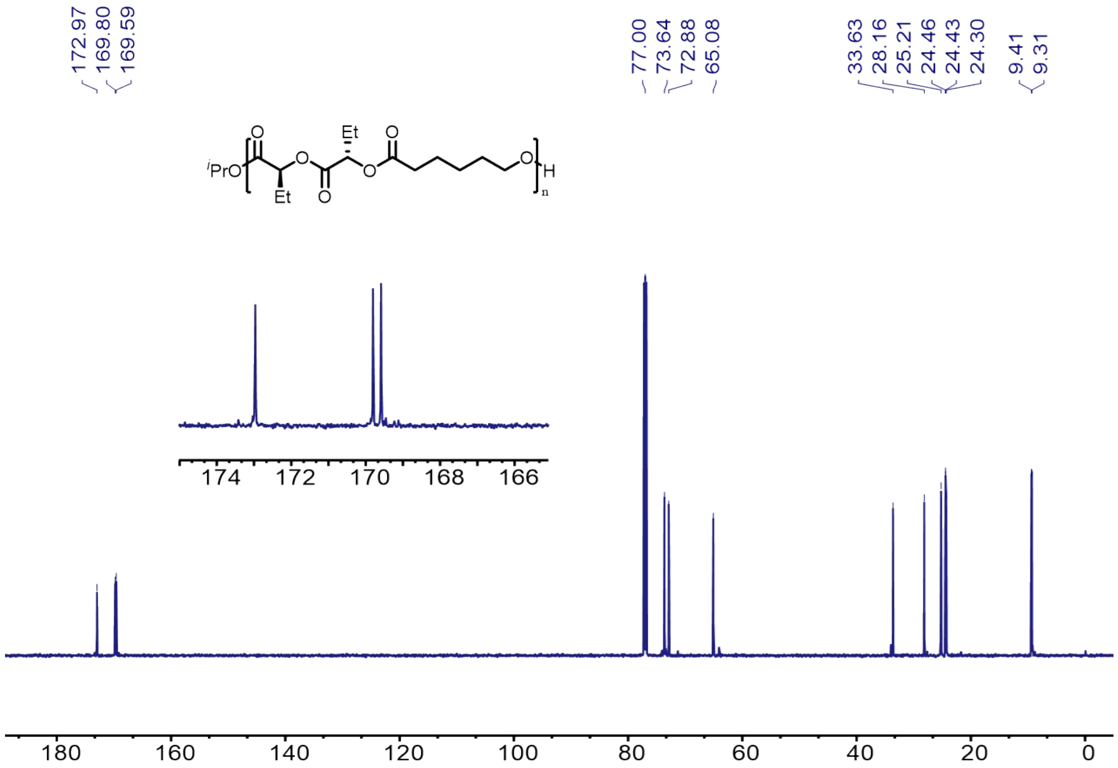

Figure S37. ^{13}C NMR spectra ($CDCl_3$, 151 MHz) of poly(*L*-EG-*alt*-CL) (P_{alt} = 0.94).

Computational Methodology for Transition State (TS) Conformer Search and Refinement: Exemplified by *si*-Face *L*-EG Insertion into an n-Butoxy-Aluminum Species

A hierarchical multi-step computational protocol, orchestrated using the *Molclus* program package (version 1.12),[12] was employed to locate and refine the lowest Gibbs free energy transition state (TS) conformer for the insertion of *L*-EG into an n-butoxy-aluminum bond of a chiral Salen-Aluminum complex. The n-butoxy group serves as a model for the growing polymer chain end after ε-caprolactone (CL) ring-opening. This EG insertion represents a key step in the alternating copolymerization of EG with CL. The conformational search was performed independently for nucleophilic attack of the n-butoxy group onto both the *re*-face and *si*-face of the EG carbonyl group. *Molclus* was utilized for initial conformer generation, batch job submission and management for quantum chemical calculations performed with *xtb* (version 6.7.1),[13] *Gaussian 16* (Revision C.02),[14] and *ORCA* (version 6.0.1),[15-22] as well as for subsequent conformer processing using its *isostat* module. The *isostat* module was used for deduplication/clustering and energy-window-based selection after single-point energy calculations. Two conformers were considered similar and grouped into the same cluster if their energy difference was simultaneously less than 0.2 kcal/mol and the maximum absolute difference between their sorted one-dimensional intramolecular distance arrays (derived from their respective distance matrices) was less than 0.2 Å. All DFT and composite method calculations performed with *ORCA* (r^2SCAN-3c,[23] B97-D3,[24, 25] PWPB95-D4[26, 27]) employed tightSCF convergence criteria. The overall workflow for identifying such transition states is depicted in Figure S38. The methodology is detailed below, using the *si*-face attack pathway as a specific example. An identical computational procedure was applied to investigate the *si*-face attack pathway.

1. Initial Conformer Generation for the *si*-Face EG Insertion TS1:

Drawing from literature precedents indicating that SalenAl complexes typically adopt a six-coordinate geometry in ROP transition states,[28] four distinct initial guess geometries for the transition state were manually constructed. These initial guesses specifically modeled the attack of the n-butoxy group onto the *si*-face of the coordinated EG carbonyl carbon and represented plausible coordination isomers around the aluminum center: *fac-mer* 1, *fac-mer* 2, *mer-fac* 1, and *mer-fac* 2 configurations (Figure S38, top row). In these initial structures, a bond was defined between the oxygen atom of the n-butoxy group and the carbonyl carbon atom of the EG with an initial length of approximately 1.99 Å. For each of these four isomeric starting structures, the *gentor* module of *molclus* was used to perform a systematic conformational search. This involved the systematic rotation of dihedral angles around the bond axes defined by atom pairs 2-3, 3-4, 4-5, and 5-6. After this automated generation and removal of sterically unreasonable conformers by *gentor*, the unique conformers from all four initial isomeric guesses were merged, resulting in a pool of 122 initial candidate conformers for **TS1**.

2. Low-Level Optimization, First Single-Point Energy (SPE) Calculation, Deduplication & Filtering:

The 122 initial candidate conformers for **TS1** were subjected to geometry optimization using the GFN1-xTB semi-empirical tight-binding method. These calculations were managed by *molclus* interfacing with the *xtb* program. During these optimizations, the O-C (5-6) bond length was constrained at its initial value of 1.99 Å. Single-point energy (SPE) calculations were then performed on all optimized structures using the

r^2SCAN-3c composite method with the SMD[29] implicit solvation model for toluene, executed via *molclus* calls to the *ORCA* program package. The *isostat* module of *molclus* was subsequently employed to process these conformers. Using the similarity criteria defined above, *isostat* performed deduplication and clustering, yielding 82 unique representative conformers. From these unique representatives, *isostat* further selected the 22 lowest-energy structures falling within a 6 kcal/mol window of the overall minimum energy conformer.

3. **Mid-Level Constrained Optimization, Second SPE Calculation, Deduplication & Filtering**:

The 22 selected conformers were further optimized using Density Functional Theory (DFT) at the B97-D3/def2-SV(P)[30] level of theory (employing tightSCF convergence), with the O-C bond length still constrained at 1.99 Å. These DFT optimizations were managed by *molclus* using the *ORCA* program package. Single-point energy calculations were then performed on these 22 B97-D3/def2-SV(P) optimized structures using r^2SCAN-3c/SMD(toluene), managed by *molclus* with *ORCA*. The *isostat* module of *molclus* was again used to process these 22 energy-evaluated conformers. It performed deduplication/clustering based on the defined similarity criteria and then selected the 6 unique representative conformers falling within a 3 kcal/mol energy window of the lowest-energy structure in this set.

4. **Initial Transition State Search and Gibbs Energy Ranking**:

The 6 candidate conformers were subjected to full transition state (TS) optimization (without bond constraints) and frequency analysis using the B97-D3 functional with the def2-SVP basis set and D3 dispersion correction. These calculations were managed by *molclus* using *ORCA*. Frequency calculations confirmed the nature of the stationary points as true transition states (one imaginary frequency corresponding to the nucleophilic attack of the n-butoxy oxygen on the EG carbonyl carbon and concomitant ring-opening of EG) and provided thermal corrections to the Gibbs free energy (at 298.15 K and 1 atm). To obtain more accurate energies, single-point energy calculations were performed on these B97-D3/def2-SVP optimized TS geometries using the PWPB95 functional with the D4 dispersion correction and the def2-TZVPP basis set (employing tightSCF convergence), including the SMD(toluene) implicit solvent model, managed by *molclus* with *ORCA*. The Gibbs free energy of each transition state, G_{solv}^{0}, was calculated as the sum of the PWPB95-D4/def2-TZVPP/SMD(toluene) electronic energy and the B97-D3/def2-SVP thermal correction. The *isostat* module of *molclus* was used to rank these TSs based on their G_{solv}^{0} values, and the conformer with the lowest G_{solv}^{0} was identified as the primary candidate for **TS1**.

5. **Final Transition State Refinement and Gibbs Free Energy Determination:**

The geometry of the primary **TS1** candidate was further refined. It was re-optimized as a transition state, and a subsequent frequency analysis was performed using the B3LYP[31, 32] functional with D3(BJ)[33] dispersion correction and the 6-31G(d,p)[34, 35] basis set. These calculations were performed using the Gaussian 16 program package. This frequency analysis confirmed the TS nature (one imaginary frequency) and provided thermal corrections to the Gibbs free energy (G_{corr} at 298.15 K and 1 atm). To further verify

that this transition state correctly connects the desired reactant and product intermediates, Intrinsic Reaction Coordinate (IRC)[36] calculations were performed in both forward and reverse directions from this TS geometry.

A final, high-level single-point energy calculation was then performed using PWPB95-D4/def2-TZVPP with the SMD(toluene) implicit solvent model in the ORCA program package. This provided the electronic energy in solution (E_{solv}).

The final Gibbs free energy of the transition state in toluene solution, G^0_{solv} was computed as:

$$G^0_{solv} = E_{solv} + G_{corr} + \Delta G_{solv}$$

where G_{corr} is the gas-phase Gibbs free energy correction obtained from frequency analysis, and ΔG_{solv} is an empirical correction of +1.89 kcal/mol representing the estimated free energy change for transferring the species from the gas phase to toluene solution.

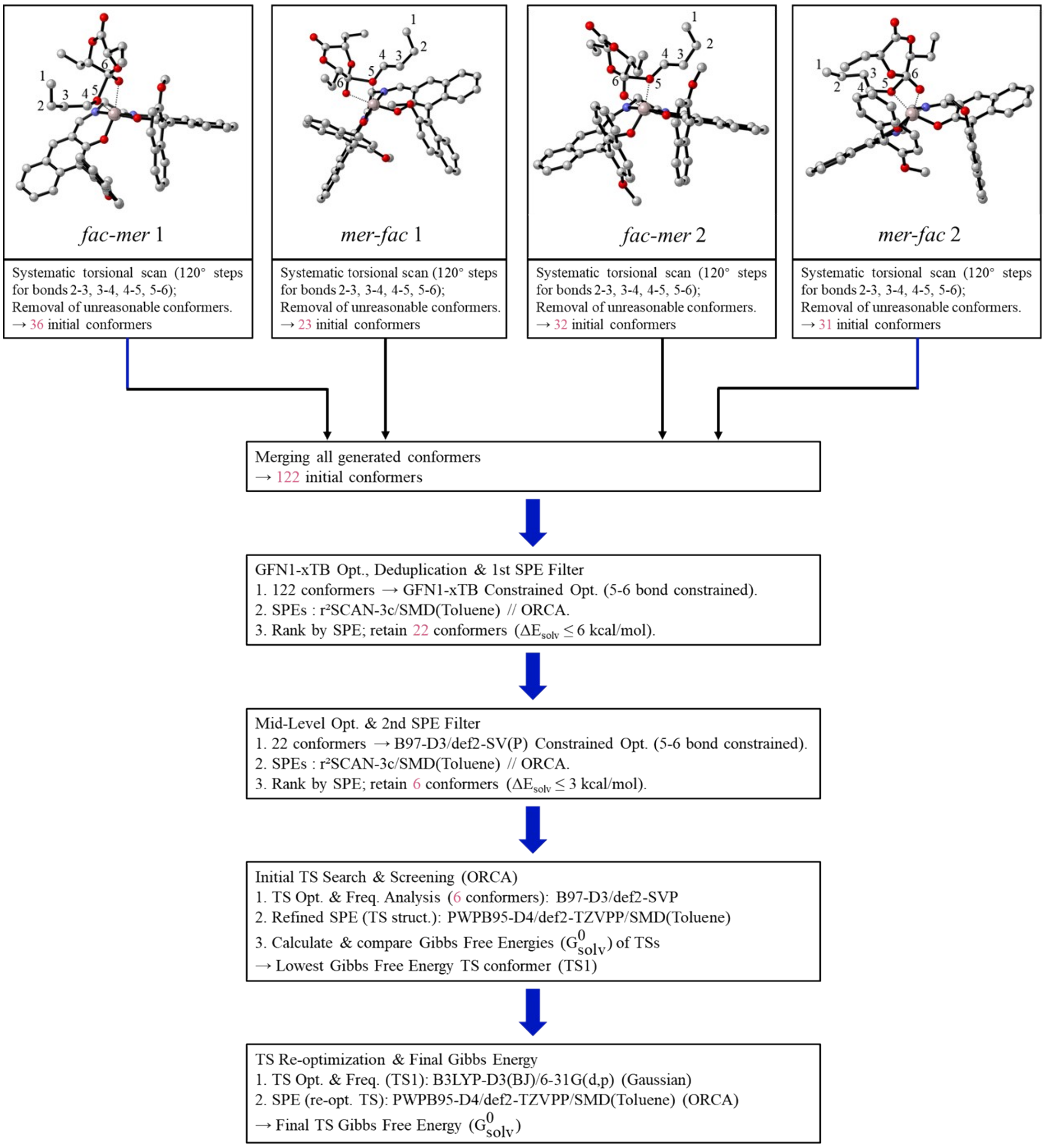

Figure S38. Schematic of the hierarchical multi-step protocol for transition state (TS) conformer search and refinement.

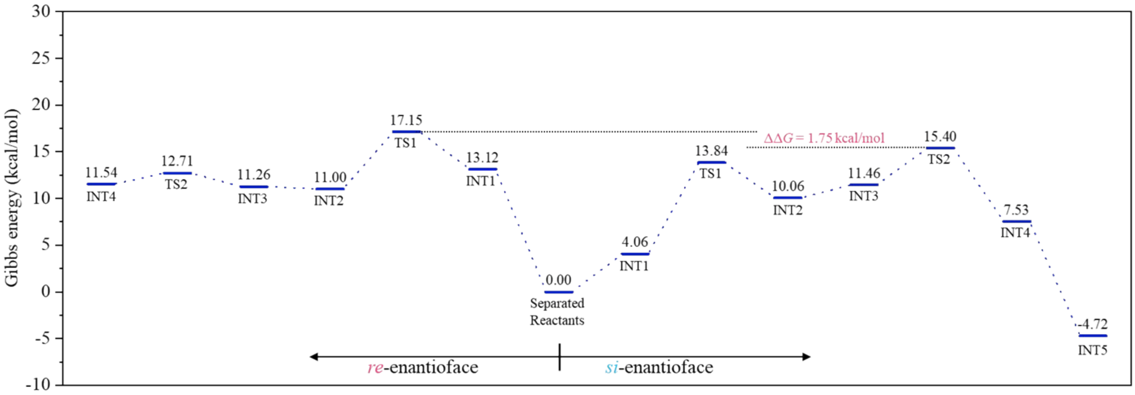

Figure S39. Calculated Gibbs free energy profile for the reaction of *R,R*-SalenAl-O^{n}Bu with L-EG.

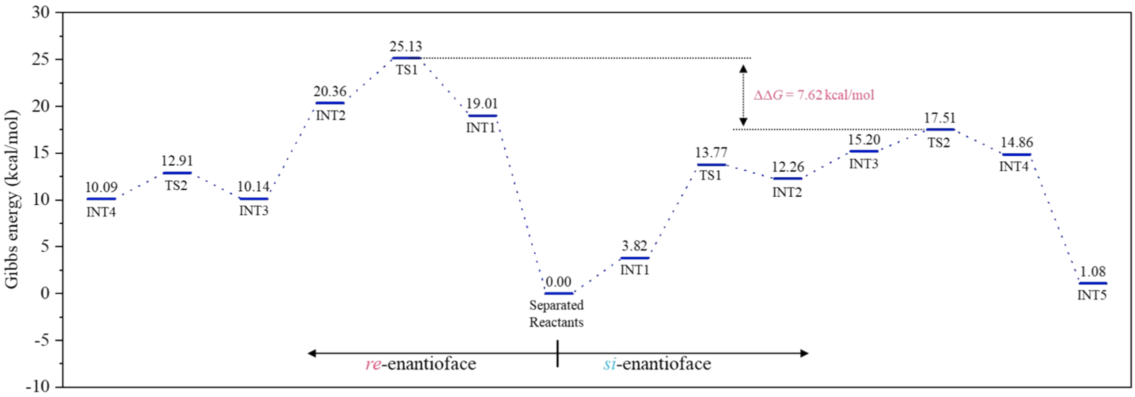

Figure S40. Calculated Gibbs free energy profile for the reaction of *R*,*R*-SalenAl-O^{n}Bu with CL.

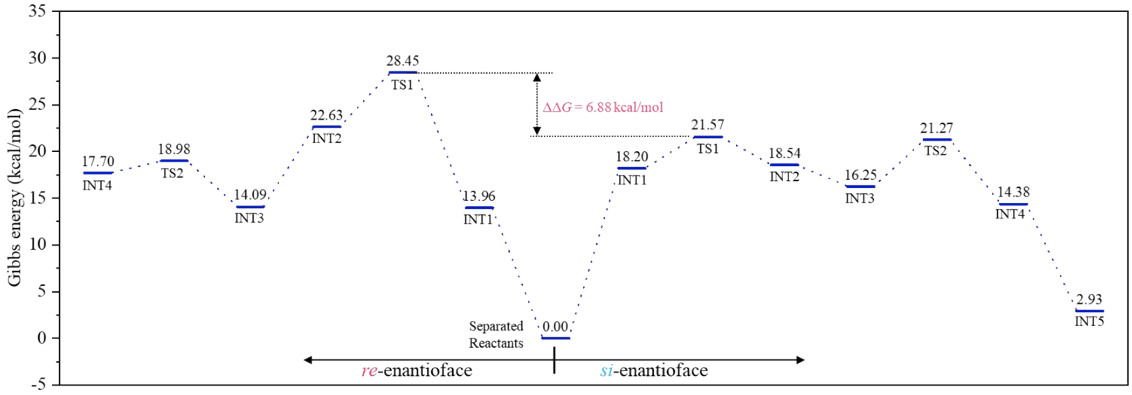

Figure S41. Calculated Gibbs free energy profile for the reaction of *R*,*R*-SalenAl-*S*-M2B with CL.

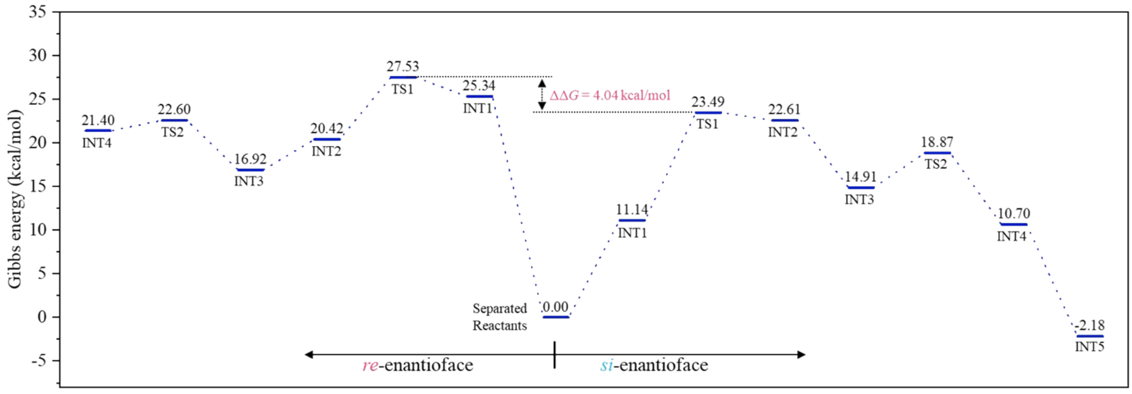

Figure S42. Calculated Gibbs free energy profile for the reaction of *R*,*R*-SalenAl-*S*-M2B with L-EG.

Coordinates (B3LYP-D3(BJ)/6-31G(d,p))

CL

C	-1.29186500	-0.01139900	0.04972600
C	-0.48596800	-1.15392400	0.64537600
C	0.62108500	1.47316400	0.41543100
C	0.71765100	-1.57732300	-0.22149500
C	1.70784500	0.79502400	-0.41572700
C	1.94676500	-0.67710900	-0.06596700
H	-1.19228400	-1.97957200	0.74084500
H	0.99342700	-2.60021500	0.05610200
H	0.70886200	1.20631400	1.47643000
H	2.64052400	1.35238600	-0.26629200
H	-0.14295700	-0.89361800	1.65481100
H	0.71023500	2.55863500	0.33927600
H	0.40626100	-1.61628700	-1.27263400
H	1.44180300	0.90525900	-1.47410100
H	2.29771100	-0.73841000	0.97372900
H	2.76022200	-1.06750600	-0.68803000
O	-0.71188800	1.21414400	-0.05569700
O	-2.42018000	-0.14948300	-0.35876100

L-EG

C	-1.33402300	0.16120700	-0.29354700
C	-0.48633600	1.33076700	0.20046900
O	-0.93195300	2.40353800	0.51844000
O	0.84249600	1.08368500	0.26059600
C	1.33393800	-0.16101200	-0.29379000
C	0.48630800	-1.33084700	0.19967200
O	0.93196000	-2.40380100	0.51697600
O	-0.84251700	-1.08380000	0.26008500
H	-1.23744200	0.11085100	-1.38934000
H	1.23722900	-0.11004100	-1.38954400
C	-2.79832600	0.29437100	0.09090100
H	-2.86148000	0.30906400	1.18386500
H	-3.13450000	1.27600900	-0.25396400
C	2.79828600	-0.29439000	0.09041200
H	2.86156700	-0.30969800	1.18336100
H	3.13442100	-1.27583400	-0.25504300
C	-3.66265000	-0.82454200	-0.48972200
H	-3.63165400	-0.82145400	-1.58509800
H	-3.32014700	-1.80318200	-0.14476900
H	-4.70530600	-0.70045400	-0.18557700
C	3.66254200	0.82484900	-0.48968200
H	4.70523400	0.70059100	-0.18572900
H	3.63141800	0.82237700	-1.58505600
H	3.32007800	1.80329500	-0.14413900

***R*,*R*-Al-O^{n}Bu**

C	7.58918100	0.54559300	-1.14086100
C	6.77533400	1.64606700	-1.20283000
C	5.39197800	1.55187400	-0.87429200
C	4.83113500	0.28302400	-0.48468900
C	5.71916900	-0.83184700	-0.41282500
C	7.04798500	-0.70290600	-0.73276200
H	4.97107100	3.64247600	-1.15503100
H	8.64211400	0.62276000	-1.39187800
H	7.17067100	2.61414400	-1.49896100
C	4.55520500	2.67281100	-0.88967400
C	3.44910500	0.17220400	-0.17701700
H	5.32559800	-1.79032100	-0.09907200
H	7.69919600	-1.56970900	-0.66993900
C	2.61809300	1.30416200	-0.21854700
C	3.20432900	2.57741700	-0.55915800
C	-3.63608400	-4.10068900	2.11237400
C	-2.60065000	-4.33911300	1.23971400
C	-2.21442700	-3.36818100	0.27893900
C	-2.92499200	-2.12579700	0.20560600
C	-3.97785100	-1.90542600	1.13749000
C	-4.32275700	-2.86454700	2.06172100
H	-0.58049100	-4.52052200	-0.54184500
H	-3.92109100	-4.84932900	2.84505000
H	-2.05278700	-5.27732800	1.27413800
C	-1.12674900	-3.58523900	-0.60018300
C	-2.54085600	-1.15159200	-0.76729000
H	-5.12849600	-2.66914900	2.76309900
C	-1.46518300	-1.41609300	-1.60439600
C	-0.74743100	-2.63334100	-1.51243400
H	0.09743500	-2.81713300	-2.16387900
C	2.87832400	-1.11942100	0.28820000
C	2.73482600	-2.24111100	-0.57613400
C	2.51605800	-1.23974200	1.62736900
C	3.05933700	-2.17788900	-1.96110400
C	2.25498300	-3.48595800	-0.05212000
C	2.00145500	-2.45786800	2.13533400
C	2.92559600	-3.27987400	-2.77324300
C	2.14648900	-4.60854500	-0.91409700
C	1.88414300	-3.55171700	1.31098900

H	1.70086800	-2.53179200	3.17210200
C	2.46926700	-4.51422200	-2.24729600
H	3.17488900	-3.20576900	-3.82758100
H	1.79399600	-5.54849300	-0.49727300
H	1.49417500	-4.48546000	1.70574800
H	2.37581700	-5.37815700	-2.89763600
C	-3.23958500	0.15598400	-0.86334400
C	-4.59702500	0.23293900	-1.27856600
C	-2.53212900	1.32289800	-0.53211700
C	-5.32659400	-0.91789600	-1.70033900
C	-5.28437400	1.49726600	-1.29593900
C	-3.23605300	2.58279000	-0.54706000
C	-6.64051500	-0.82307700	-2.08767800
H	-4.82096300	-1.87528700	-1.71527000
C	-6.64956400	1.55679900	-1.69741900
C	-4.57619700	2.64478400	-0.91750300
C	-7.31924600	0.42427000	-2.08194000
H	-7.16966900	-1.71575900	-2.40768900
H	-7.14696400	2.52316300	-1.69544200
H	-5.08457700	3.60667900	-0.91589700
H	-8.35920300	0.47568300	-2.38807300
C	2.42817200	3.77822000	-0.55817600
N	1.17662500	3.87590500	-0.23304000
N	-1.34717900	3.88269900	0.19094100
C	0.55128200	5.20178200	-0.32264600
C	-0.73250800	5.15866200	0.50703700
O	1.33723200	1.19783800	0.02366200
O	-1.25941600	1.25234800	-0.21378800
C	-2.57821400	3.80619600	-0.18459800
Al	0.00023400	2.34791000	0.46302100
H	2.95996600	4.68849200	-0.85682500
H	-3.17835800	4.72226400	-0.23761400
H	-1.38394900	6.01200700	0.28363200
H	0.29443700	5.40483200	-1.37021200
H	1.22977400	5.98891900	0.02432200
H	3.41180900	-1.23757900	-2.36895500
H	-4.50613600	-0.96013000	1.11594700
C	0.21880600	-0.27338100	-2.82959100
H	0.27117600	0.65017700	-3.41008100
C	2.42572100	-0.20101500	3.78468000
H	2.62581300	0.79810100	4.17119300
H	-0.48367500	5.16126000	1.57331300
O	-1.16418500	-0.47579600	-2.54644400
O	2.70749000	-0.13610300	2.39653000
H	3.07284400	-0.92956000	4.29042300
H	1.37887300	-0.44933400	3.97379200
H	0.64169400	-1.09013600	-3.42592800
H	0.78458800	-0.15711600	-1.90276100
C	-1.12101400	2.35411300	3.00766700
C	-1.15775000	1.01163900	3.74154400
C	-1.26962400	-1.53265300	3.54260500
H	-1.18809300	-2.37515500	2.85168900
H	-0.49725000	-1.65081700	4.31380100
H	-2.24099000	-1.60920600	4.04254300
O	0.02389900	2.49815200	2.20359000
H	-1.15044400	3.16762500	3.75120600
H	-2.04928400	2.44582000	2.41456500
H	-2.06693200	0.98272200	4.36022600
H	-0.30863900	0.96754400	4.43654800
C	-1.12989600	-0.19998800	2.80703100
H	-0.20281800	-0.18800400	2.22571300
H	-1.93753500	-0.11221000	2.07321700

***R*,*R*-Al-*S*-M2B**

C	7.86449500	1.04682700	0.66210600
C	7.02267700	2.00812100	0.16371800
C	5.64108400	1.73356800	-0.04113300
C	5.11972800	0.42866200	0.26601400
C	6.02375800	-0.53657300	0.79704500
C	7.35077900	-0.23586000	0.98630400
H	5.13955700	3.70228500	-0.75614700
H	8.91674600	1.26286300	0.81704600
H	7.39449600	2.99999600	-0.07948300
C	4.75779400	2.71000300	-0.52567300
C	3.74533700	0.13873300	0.04577100
H	5.64377400	-1.51807800	1.05247700
H	8.01798100	-0.98974700	1.39345700
C	2.87777100	1.13083300	-0.42260700
C	3.40466700	2.44435200	-0.70179700
C	-3.62853800	-0.49066300	4.34382000
C	-2.94789500	-1.68474700	4.26297200
C	-2.49752200	-2.18555400	3.01507900
C	-2.74913500	-1.43401300	1.82251200
C	-3.48335600	-0.22550100	1.93939100
C	-3.90151800	0.24085500	3.16593900
H	-1.65657300	-4.02151300	3.80129100
H	-3.96010400	-0.11341000	5.30625700
H	-2.74205900	-2.26516300	5.15847300

C	-1.81752500	-3.42728200	2.90604900
C	-2.26726400	-1.90490700	0.55636100
H	-4.43418400	1.18474500	3.21973300
C	-1.53484500	-3.08301600	0.51879400
C	-1.35343000	-3.86177200	1.69325800
H	-0.81595100	-4.79745500	1.59038300
C	3.17941600	-1.20427300	0.33528200
C	3.51699200	-2.34237100	-0.45712400
C	2.25501100	-1.34253400	1.36459600
C	4.39151600	-2.25741700	-1.57689800
C	2.93443600	-3.61681000	-0.15362000
C	1.68620900	-2.60348100	1.66602900
C	4.67999300	-3.36603400	-2.33847100
C	3.25740400	-4.74168300	-0.95702000
C	2.02627800	-3.70998300	0.92771400
H	0.97253600	-2.69033500	2.47388400
C	4.11286100	-4.62534700	-2.02733200
H	5.34723600	-3.27437400	-3.19046800
H	2.80542200	-5.69894900	-0.71106000
H	1.58295800	-4.67376200	1.15768900
H	4.35066400	-5.49182600	-2.63663400
C	-2.69371200	-1.20017100	-0.68697800
C	-3.81535100	-1.69981400	-1.40495900
C	-2.03904700	-0.03531900	-1.11150500
C	-4.51006600	-2.87819100	-1.00395200
C	-4.29559600	-1.01149300	-2.57438400
C	-2.53109200	0.64728900	-2.28546900
C	-5.59213500	-3.33899600	-1.71344800
H	-4.16842600	-3.40849700	-0.12389800
C	-5.42048600	-1.51863400	-3.28523000
C	-3.63565200	0.15634300	-2.97721200
C	-6.05814200	-2.65838900	-2.86895100
H	-6.10145700	-4.24022300	-1.38553300
H	-5.76092700	-0.98066800	-4.16600400
H	-3.99047400	0.68876900	-3.85706700
H	-6.91425400	-3.04201400	-3.41436200
C	2.55716500	3.49787800	-1.19968000
N	1.27758000	3.39706800	-1.27811300
N	-0.85895000	2.41434100	-2.30035200
C	0.43628900	4.41865100	-1.87089100
C	-0.41128800	3.67548700	-2.91065700
O	1.60584200	0.83352500	-0.62832600
O	-0.99886400	0.41861700	-0.45746800
C	-1.91924600	1.84832900	-2.77739200

Al	0.13220200	1.84573200	-0.64071000
H	3.04802300	4.42099200	-1.52902100
H	-2.41822000	2.31703800	-3.63155500
H	0.20758600	3.44353500	-3.78644400
H	1.01074800	5.23235900	-2.32920400
H	-0.22225700	4.81173900	-1.09127200
H	4.82393900	-1.29645300	-1.82893200
H	-3.70137500	0.34598100	1.04739800
C	-0.26071200	-2.75264700	-1.49604900
H	0.15349900	-1.89486900	-0.96069100
C	0.62364200	-0.12990700	2.61006100
H	-0.11609500	-0.39035600	1.84907300
H	-1.26610800	4.27943300	-3.22843900
O	-0.98252500	-3.61928200	-0.60668900
O	1.94885100	-0.22775800	2.08434000
H	0.49691700	0.91587400	2.89071100
H	0.48535700	-0.76274100	3.49465800
H	-0.90565800	-2.40423900	-2.30750400
H	0.55492800	-3.35187900	-1.90418200
C	-1.53987100	2.73541700	1.42746800
C	-2.67752500	3.23504300	0.53118300
O	-2.55952500	3.90117400	-0.47888400
O	-3.88452100	2.83856600	0.99857300
C	-5.01270500	3.21475800	0.19393400
H	-4.93649300	2.76041600	-0.79735600
H	-5.88875300	2.83581900	0.71881500
H	-5.06567300	4.30030500	0.08508500
O	-0.32234500	2.80726900	0.76450900
H	-1.80130500	1.70552300	1.70174100
C	-1.50176000	3.57487000	2.72008300
H	-2.46291100	3.47396400	3.23662300
H	-0.73913900	3.12361100	3.36430000
C	-1.16383700	5.04392900	2.46794100
H	-0.21487500	5.12000100	1.93157200
H	-1.93231900	5.53432500	1.86012800
H	-1.07744900	5.59631500	3.40855200

_si_-enantioface: _R,R_-Al-O^{n}Bu + L-EG

INT1

C	7.66270900	2.12924100	0.05512300
C	6.62603700	2.80988700	-0.53170700
C	5.36693100	2.18082700	-0.74214200
C	5.17995300	0.81413700	-0.33410100

C	6.28073000	0.14013600	0.26543400
C	7.48213300	0.77924800	0.45448200
H	4.43651500	3.87806300	-1.68845800
H	8.61961200	2.61584500	0.21427400
H	6.74780900	3.84326900	-0.84526300
C	4.29561000	2.85268600	-1.35352800
C	3.92701200	0.17860600	-0.53362000
H	6.14999900	-0.89202600	0.56928200
H	8.30802700	0.24553200	0.91531100
C	2.85697700	0.88154200	-1.09232000
C	3.05845400	2.24332800	-1.52839100
C	-2.01841800	0.18077300	4.47248600
C	-1.16209300	-0.89156900	4.58172500
C	-0.99257300	-1.80513200	3.50932000
C	-1.70106800	-1.58965300	2.28468200
C	-2.59731300	-0.48929800	2.22107100
C	-2.75758500	0.37270200	3.28269400
H	0.36069900	-3.13252400	4.55832000
H	-2.13762300	0.87407800	5.29890100
H	-0.60442200	-1.06125400	5.49922000
C	-0.14872600	-2.94232800	3.61727200
C	-1.50102000	-2.47523800	1.17311400
H	-3.44549900	1.20847400	3.20588000
C	-0.61448300	-3.53343600	1.31009800
C	0.03006800	-3.78296100	2.55058600
H	0.68148100	-4.64762900	2.60704200
C	3.70441700	-1.24859000	-0.17065900
C	3.71999200	-2.25279300	-1.18244900
C	3.42598700	-1.61031900	1.14089400
C	3.98824800	-1.94650800	-2.54553900
C	3.45617900	-3.61795400	-0.83777500
C	3.15413000	-2.95964800	1.47949900
C	3.99046000	-2.93010400	-3.50653600
C	3.47261700	-4.61066500	-1.85288700
C	3.16728300	-3.93292700	0.51020900
H	2.90774900	-3.22537300	2.49754300
C	3.72987800	-4.27887900	-3.16195500
H	4.19647400	-2.67206400	-4.54114400
H	3.27464800	-5.64169600	-1.57137300
H	2.93746500	-4.96106700	0.77338100
H	3.73791600	-5.04515900	-3.93090400
C	-2.25882900	-2.22679600	-0.08550600
C	-3.41407100	-2.97565400	-0.41846100
C	-1.81880200	-1.19301700	-0.92476000
C	-3.89444100	-4.03297100	0.40724300
C	-4.15026600	-2.67397600	-1.62226800
C	-2.59216200	-0.85695900	-2.09479800
C	-5.01968300	-4.74037000	0.06369000
H	-3.35133400	-4.26517500	1.31552900
C	-5.31292000	-3.43222900	-1.94433100
C	-3.71966300	-1.61005900	-2.42512700
C	-5.74125700	-4.44288200	-1.12397900
H	-5.36720300	-5.54094200	0.70966200
H	-5.85465100	-3.18670300	-2.85371800
H	-4.28770000	-1.35394100	-3.31664900
H	-6.62851900	-5.01550300	-1.37347700
C	2.00643500	2.97596400	-2.19148900
N	0.78586400	2.56991400	-2.24402100
N	-1.26424000	1.08614900	-2.71524200
C	-0.25608200	3.27361800	-2.96936800
C	-1.07494200	2.18858500	-3.67493100
O	1.69175600	0.27392600	-1.23039000
O	-0.71100800	-0.56558400	-0.64769800
C	-2.24891100	0.26321300	-2.92144100
Al	0.04942800	0.98802200	-1.19080900
H	2.28466400	3.91904600	-2.67548800
H	-2.90031200	0.44212800	-3.78029400
H	-0.51436300	1.80903900	-4.53772800
H	0.14014100	4.00685100	-3.68130500
H	-0.89598400	3.79278100	-2.24890700
H	4.19351300	-0.91695400	-2.81544600
H	-3.16122300	-0.33497400	1.31201200
C	-0.02615200	-3.93309700	-0.99169800
H	-0.93006600	-3.90582600	-1.60608000
C	3.15232400	-0.94928900	3.42045300
H	2.13356900	-1.33620800	3.53380200
H	-2.03964900	2.58061600	-4.00673000
O	-0.30874100	-4.42958500	0.32428200
O	3.42829900	-0.60706900	2.06895600
H	3.25579600	-0.02641800	3.98779700
H	3.86911600	-1.68678800	3.80255800
H	0.69039400	-4.62834200	-1.43067800
H	0.42207700	-2.93734100	-0.94557700
C	-0.05410900	1.81547300	1.50622100
C	1.34310900	2.32295700	1.85024300
C	3.07255100	2.71304200	3.66740300
H	3.28565100	3.73061300	3.32108900
H	3.28173900	2.67867400	4.74219200

H	3.77545000	2.04480300	3.15814700
O	-0.37371100	2.05218700	0.14596500
C	-4.22426800	1.86388900	-0.38100300
C	-3.81680300	2.90665900	-1.41519400
O	-4.03774500	2.78629400	-2.60182200
O	-3.17429100	3.98356500	-0.94022000
C	-2.65728000	3.91852100	0.42901200
C	-3.71871100	3.42327200	1.38592700
O	-3.83167500	3.79113900	2.53016000
O	-4.54721300	2.47395300	0.88940400
H	-3.35858700	1.20384600	-0.23104500
H	-1.84008600	3.17987200	0.40697100
C	-5.44499900	1.06480500	-0.81868000
H	-5.22617200	0.65222100	-1.80512100
C	-2.10504000	5.28446000	0.79340100
H	-1.82368100	5.22969200	1.84847300
H	-2.90268500	6.03202900	0.71911300
H	-6.27604000	1.76700600	-0.94651200
H	-0.79609000	2.33378200	2.13447800
H	-0.13791300	0.74945000	1.75203100
H	1.45455200	3.34036500	1.45139600
H	2.08765100	1.69911100	1.34395600
C	1.62665400	2.32209500	3.35293900
H	1.40686100	1.32724600	3.76047500
H	0.93599500	3.00946700	3.85941000
C	-0.90090700	5.67289300	-0.06604700
H	-1.19571900	5.82071500	-1.10907100
H	-0.45522900	6.60591100	0.29027900
H	-0.13830000	4.88820600	-0.03236700
C	-5.82083900	-0.04297200	0.16417800
H	-6.02674700	0.36541000	1.15625200
H	-6.71251300	-0.57209900	-0.18279900
H	-5.02132400	-0.78116900	0.25733900

TS1

C	6.25371700	-3.32007400	0.58076000
C	5.96958300	-2.24370300	-0.22006100
C	4.62680800	-1.92582600	-0.56789000
C	3.54631700	-2.73370000	-0.06852500
C	3.88727000	-3.85707500	0.73916600
C	5.19450100	-4.13644200	1.05512900
H	5.13739600	-0.29479200	-1.87894800
H	7.27957600	-3.55555100	0.84519800
H	6.76604600	-1.61285800	-0.60587000
C	4.32336600	-0.86282800	-1.43321100
C	2.19897700	-2.40677800	-0.38996000
H	3.09233000	-4.49894900	1.09765200
H	5.42323000	-4.99880900	1.67451700
C	1.90695300	-1.27403500	-1.16432700
C	3.01197300	-0.52583000	-1.73625000
C	-1.49984100	2.74844900	4.09673000
C	-1.45989800	1.40992500	4.41163400
C	-1.99945400	0.43637900	3.53245700
C	-2.56410300	0.85031000	2.28427100
C	-2.60424000	2.24029900	1.99619700
C	-2.09565100	3.16315400	2.88297600
H	-1.60562400	-1.25916300	4.81944600
H	-1.08473200	3.48603200	4.77593300
H	-1.01510700	1.07572600	5.34491000
C	-2.00977800	-0.94419400	3.86110400
C	-3.08365600	-0.12223300	1.37194700
H	-2.15577100	4.22187700	2.65340400
C	-3.03647200	-1.46443700	1.72466600
C	-2.52290000	-1.86534000	2.98584100
H	-2.53641400	-2.92478500	3.21142400
C	1.08049500	-3.27927400	0.06366200
C	0.35970800	-4.07147500	-0.88258100
C	0.75345100	-3.38086100	1.41121900
C	0.64142100	-4.03012300	-2.27646400
C	-0.67087900	-4.96026600	-0.43463600
C	-0.27070400	-4.25620300	1.85147600
C	-0.06517000	-4.80181900	-3.16848700
C	-1.37956100	-5.74738000	-1.38078600
C	-0.96374000	-5.02236500	0.94740200
H	-0.51621200	-4.31635900	2.90305900
C	-1.09166000	-5.66902700	-2.72223600
H	0.16801200	-4.74906100	-4.22794300
H	-2.15891900	-6.41311100	-1.01923800
H	-1.75528100	-5.68213000	1.29023400
H	-1.64130900	-6.27220000	-3.43821500
C	-3.61822500	0.29957900	0.04802200
C	-5.00481000	0.29868000	-0.23885800
C	-2.68334000	0.61451600	-0.94758000
C	-5.98377200	-0.01218000	0.74772000
C	-5.46209800	0.60686200	-1.56983100
C	-3.14200300	0.86340300	-2.29114100
C	-7.32236600	-0.00264700	0.44087100
H	-5.64790000	-0.25251500	1.74976000

C	-6.85834300	0.60819600	-1.84867300
C	-4.50741600	0.86814900	-2.56565400
C	-7.77157200	0.31210900	-0.86917600
H	-8.05102900	-0.23952300	1.21042000
H	-7.18449200	0.84596600	-2.85778500
H	-4.84518900	1.05950100	-3.58209500
H	-8.83426100	0.31409700	-1.08926800
C	2.75385700	0.45673800	-2.76285000
N	1.61306400	1.03674300	-2.89769300
N	-0.93345000	0.93817800	-3.30181400
C	1.22005400	1.75156200	-4.09684300
C	-0.11761900	1.10591600	-4.50738800
O	0.65705500	-0.94134900	-1.41528500
O	-1.41588500	0.62671800	-0.64267100
C	-2.21945100	1.03019400	-3.38448200
Al	0.12726100	0.81809600	-1.55007300
H	3.55058100	0.65226300	-3.48873000
H	-2.67185700	1.22773500	-4.36295400
H	0.08717400	0.11225400	-4.92490200
H	1.96122000	1.66430900	-4.89972000
H	1.06541600	2.80231300	-3.84419000
H	1.43090300	-3.37765200	-2.62789700
H	-3.05262700	2.56330300	1.06326700
C	-3.06362300	-2.56193600	-0.42254300
H	-3.74250500	-2.02102100	-1.08584500
C	1.39182800	-2.92657900	3.66594700
H	0.41420400	-2.65381700	4.07942600
H	-0.63479700	1.70286200	-5.26724200
O	-3.49792200	-2.48810100	0.94396800
O	1.47631900	-2.61507700	2.28297100
H	2.16376800	-2.33483000	4.15461300
H	1.58116600	-3.99161300	3.84738400
H	-3.07069300	-3.61980000	-0.68279000
H	-2.04737500	-2.17320700	-0.53221400
C	0.93511100	1.03909200	1.19623300
C	2.25301500	0.75939700	1.90699300
C	3.34362900	0.14113600	4.11713300
H	4.10278600	0.92501200	4.02016000
H	3.18503000	-0.03537700	5.18694000
H	3.76189000	-0.77230000	3.67865300
O	1.14040200	1.47578900	-0.14560800
C	0.40207800	4.10052900	0.33148000
C	0.78942300	3.33018400	-0.90393600
O	-0.08250200	2.96844300	-1.72352100
O	2.02950000	3.57932200	-1.37870100
C	3.11302500	3.52391000	-0.43127500
C	2.77336400	4.09471400	0.93971900
O	3.64139000	4.32833100	1.74763300
O	1.48036500	4.26551700	1.27654900
H	-0.37486800	3.54822400	0.86036300
H	3.31412100	2.45896200	-0.26613800
C	-0.10546600	5.48586300	-0.09814500
H	-0.95050300	5.32572100	-0.77490200
C	4.31919200	4.21044700	-1.05791400
H	5.10691400	4.22409100	-0.30089800
H	4.06088800	5.25377400	-1.27335600
H	0.68613800	5.97119900	-0.68232600
H	0.36891500	1.79911600	1.75000100
H	0.31727900	0.13493100	1.19600500
H	2.94385200	1.59815600	1.76416300
H	2.72143700	-0.12462400	1.46099600
C	2.05074600	0.54422700	3.40749400
H	1.28036500	-0.22021800	3.55665700
H	1.65263400	1.46445200	3.85289000
C	4.79024900	3.50129300	-2.32761900
H	3.99783300	3.47883300	-3.08072200
H	5.65773600	4.00821000	-2.75930900
H	5.07865100	2.46709100	-2.10875800
C	-0.50883700	6.36983200	1.08113900
H	0.32042700	6.48956000	1.78197500
H	-0.81000500	7.36077400	0.72959100
H	-1.35266300	5.93897900	1.62763700

INT2

C	-6.42086100	3.04912800	0.51272400
C	-6.07828700	2.02044000	-0.32732800
C	-4.71593500	1.75730500	-0.64293400
C	-3.67811400	2.57088600	-0.06728300
C	-4.07844000	3.64421600	0.77984600
C	-5.40341500	3.87140700	1.06220800
H	-5.13274400	0.18128500	-2.05262000
H	-7.46167000	3.24292500	0.75126500
H	-6.84260400	1.38746500	-0.77037400
C	-4.35207900	0.74787100	-1.54931400
C	-2.31240500	2.29719500	-0.35634000
H	-3.31457200	4.28903800	1.19636800
H	-5.67884200	4.69607300	1.71312200
C	-1.96304900	1.21126100	-1.17298100

C	-3.02161100	0.46733200	-1.82673000
C	1.70801600	-2.88724100	3.94117100
C	1.60910200	-1.56886800	4.32349500
C	2.08137800	-0.52690600	3.48515100
C	2.64748200	-0.84985600	2.21073500
C	2.74759400	-2.22079400	1.85196200
C	2.29840500	-3.21147600	2.69758300
H	1.61064800	1.08523400	4.85244700
H	1.34205900	-3.67681800	4.58935900
H	1.16613300	-1.30396900	5.27959300
C	2.02025400	0.83609300	3.87715800
C	3.10804800	0.19035500	1.34049500
H	2.39843200	-4.25303300	2.40860500
C	2.99800900	1.51003500	1.75799900
C	2.47346500	1.82203200	3.04108000
H	2.43319900	2.86902000	3.31631200
C	-1.23014200	3.18242500	0.15757900
C	-0.51433200	4.03024500	-0.74405200
C	-0.92468300	3.23667800	1.51210300
C	-0.77496500	4.04134400	-2.14260800
C	0.48875800	4.92237200	-0.24393700
C	0.07359400	4.11396700	2.00421200
C	-0.07478900	4.86665500	-2.99066100
C	1.19037300	5.76659700	-1.14509400
C	0.76246700	4.93167500	1.14335300
H	0.30417300	4.13384300	3.06070700
C	0.92334400	5.73875900	-2.49280000
H	-0.29187200	4.85336800	-4.05471500
H	1.94749300	6.43521000	-0.74380500
H	1.53545500	5.59216200	1.52468300
H	1.46750300	6.38535600	-3.17420700
C	3.66118100	-0.16362700	0.00340700
C	5.05355700	-0.18872000	-0.24912900
C	2.74096000	-0.43390700	-1.01862000
C	6.01478200	0.07909500	0.76730500
C	5.53603500	-0.48263400	-1.57422200
C	3.22790300	-0.69238700	-2.35311400
C	7.35983100	0.04739900	0.49151500
H	5.66007700	0.30609100	1.76604400
C	6.93814600	-0.50759000	-1.82041800
C	4.60006500	-0.71923200	-2.59312700
C	7.83362800	-0.24978200	-0.81404800
H	8.07477700	0.25276000	1.28267800
H	7.28338600	-0.73367600	-2.82583700
H	4.95889100	-0.91616600	-3.60122400
H	8.90097500	-0.26981800	-1.00905000
C	-2.69831300	-0.45727400	-2.88871300
N	-1.51412400	-0.92956400	-3.04959900
N	1.04839800	-0.79888000	-3.42512600
C	-1.08234100	-1.66713100	-4.22100000
C	0.24874100	-1.00755500	-4.63566200
O	-0.69512800	0.92501200	-1.37998900
O	1.46649400	-0.38818100	-0.74467800
C	2.33759000	-0.87130300	-3.47262100
Al	-0.01878500	-0.75493400	-1.70565800
H	-3.49137200	-0.71087200	-3.60016400
H	2.81935100	-1.07161700	-4.43633400
H	0.03275600	-0.02798000	-5.08002000
H	-1.81230000	-1.62934800	-5.03794300
H	-0.90702000	-2.70383900	-3.92205200
H	-1.54387200	3.38682000	-2.53364700
H	3.19011300	-2.47614900	0.89592700
C	3.04471100	2.69396700	-0.34853500
H	3.79271700	2.21752500	-0.98637600
C	-1.59400800	2.68601000	3.73598900
H	-0.61808000	2.42863200	4.16417700
H	0.78369600	-1.61447400	-5.37481700
O	3.40175200	2.59835000	1.03811800
O	-1.63928300	2.41626800	2.34241700
H	-2.35867400	2.05767800	4.18928900
H	-1.81743800	3.73897100	3.94601500
H	3.00383900	3.75939700	-0.57211700
H	2.06161100	2.25139800	-0.53008500
C	-0.83821000	-1.09795200	1.22293900
C	-2.12861100	-0.89518500	1.99846400
C	-3.14470400	-0.60730200	4.30530800
H	-3.81650000	-1.46262300	4.17658000
H	-2.94164200	-0.50082100	5.37621600
H	-3.68708200	0.28471900	3.97150600
O	-1.10064300	-1.50963200	-0.15328300
C	-0.38004500	-3.90382000	0.32920700
C	-0.81637100	-2.88281200	-0.71382000
O	0.08109300	-2.64419500	-1.65963700
O	-2.03230700	-3.32095900	-1.28602100
C	-3.11318900	-3.43795500	-0.36824600
C	-2.73211100	-4.05886500	0.97674200
O	-3.57685300	-4.38969900	1.77718300
O	-1.42599500	-4.14378200	1.30938200

H	0.45779200	-3.51261600	0.90548600
H	-3.47648500	-2.42832900	-0.12609200
C	0.01821700	-5.21932600	-0.33629400
H	0.81944100	-4.99041200	-1.04444900
C	-4.23118000	-4.22082800	-1.05055400
H	-5.03231000	-4.35333000	-0.31918200
H	-3.85532000	-5.21843700	-1.30574400
H	-0.83193600	-5.57833700	-0.92674800
H	-0.19833700	-1.83651500	1.70368000
H	-0.27148300	-0.16964500	1.14774500
H	-2.81252200	-1.72951200	1.82576000
H	-2.62517700	0.01490200	1.64757000
C	-1.85895400	-0.80666600	3.50236700
H	-1.15091700	0.00576400	3.69825600
H	-1.36125700	-1.72762500	3.82857600
C	-4.74037600	-3.50945700	-2.30393200
H	-3.93360700	-3.38345200	-3.03081700
H	-5.54580900	-4.07728800	-2.77854000
H	-5.12991300	-2.51558400	-2.05481300
C	0.46165800	-6.27979100	0.67133400
H	-0.32685800	-6.48385500	1.40008900
H	0.71038900	-7.21605400	0.16328800
H	1.34990600	-5.95466500	1.22323100

INT3

C	7.03301000	-3.18995300	-0.37670100
C	6.65584500	-2.00466000	-0.95429900
C	5.30330600	-1.56537400	-0.90002200
C	4.31787000	-2.35607800	-0.21777900
C	4.74683400	-3.58966900	0.34968500
C	6.05857900	-3.99122000	0.27192500
H	5.61976800	0.21742600	-2.06729800
H	8.06535500	-3.52179200	-0.42126300
H	7.38128700	-1.37981800	-1.46808800
C	4.89061300	-0.38149600	-1.52781200
C	2.96707700	-1.90596800	-0.12958200
H	4.01367700	-4.21879700	0.83913200
H	6.35487600	-4.93952200	0.71077400
C	2.57997000	-0.70672200	-0.74625500
C	3.56973500	0.04107600	-1.48558700
C	-7.09859200	1.46210000	1.63900100
C	-6.17859200	1.33953600	2.65290700
C	-4.93050300	0.69957800	2.43616200
C	-4.62428700	0.15532600	1.14530900
C	-5.59616100	0.31476200	0.11717200
C	-6.79414000	0.94823300	0.35715900
H	-4.19623700	1.01358800	4.44588600
H	-8.04851000	1.95717600	1.81455300
H	-6.38781000	1.74025300	3.64125100
C	-3.96116600	0.60909600	3.46529200
C	-3.35401400	-0.47215100	0.91783400
H	-7.51163600	1.05977300	-0.45029500
C	-2.42807400	-0.50193300	1.95287500
C	-2.74032300	0.03052900	3.23136400
H	-2.00366200	-0.03211200	4.02346400
C	1.97254700	-2.69062200	0.65159300
C	0.83870100	-3.29461000	0.03064200
C	2.13700800	-2.82331400	2.02804400
C	0.61513200	-3.22586000	-1.37133200
C	-0.11526300	-4.00471800	0.82881000
C	1.21670500	-3.55972200	2.81112200
C	-0.49383600	-3.80560000	-1.93975900
C	-1.25678800	-4.57891800	0.21143200
C	0.11065400	-4.12184700	2.21876200
H	1.36197100	-3.65677000	3.87954300
C	-1.44894600	-4.48320700	-1.14429800
H	-0.64837700	-3.73492200	-3.01166700
H	-1.98268500	-5.09171200	0.83617700
H	-0.60993100	-4.66468700	2.82365100
H	-2.33232100	-4.91066100	-1.60536400
C	-3.02244300	-0.98446900	-0.43925600
C	-3.77074200	-2.04046300	-1.02234000
C	-2.01694400	-0.35072200	-1.18994700
C	-4.66688500	-2.84006100	-0.25335500
C	-3.63760600	-2.35075700	-2.42253100
C	-1.94940000	-0.60967600	-2.61215000
C	-5.36983200	-3.87041300	-0.82578500
H	-4.76649100	-2.62971000	0.80467200
C	-4.39886400	-3.41455800	-2.98708400
C	-2.75925200	-1.58309300	-3.19425000
C	-5.24439500	-4.16395200	-2.21095500
H	-6.03201000	-4.47379300	-0.21203000
H	-4.28365200	-3.62601000	-4.04692600
H	-2.69901500	-1.75767600	-4.26630800
H	-5.81491300	-4.97879300	-2.64483900
C	3.20365600	1.17768100	-2.28730600
N	2.02342300	1.69145100	-2.30351400
N	-0.27843400	1.08662400	-3.08593800

C	1.60414300	2.60682000	-3.34839900
C	0.46176400	1.87294200	-4.08445400
O	1.32834900	-0.29001200	-0.65466800
O	-1.22124300	0.52795000	-0.64119300
C	-1.13457200	0.19358900	-3.47700900
Al	0.39582500	1.15536000	-1.20284400
H	3.97147300	1.57623300	-2.95751500
H	-1.27081400	0.03256500	-4.55223900
H	0.87644400	1.20695600	-4.85130500
H	2.41649900	2.86644800	-4.03594500
H	1.22602700	3.51488600	-2.87656600
H	1.34041100	-2.70979900	-1.98894100
H	-5.37679600	-0.05753000	-0.87476200
C	-0.09393400	-0.47921400	2.35497800
H	-0.21350600	0.60182900	2.44256100
C	3.47813200	-2.33480600	3.95476300
H	2.66863300	-1.94153100	4.58291200
H	-0.20578500	2.58903100	-4.57546300
O	-1.22218400	-1.07242200	1.71064900
O	3.20575700	-2.16393500	2.57621300
H	4.38833800	-1.76708700	4.15086900
H	3.64839000	-3.38859500	4.20843500
H	0.75406800	-0.68356200	1.71135100
H	0.07420600	-0.92262600	3.34411400
C	-1.46042900	3.01252200	1.62907600
C	-2.72485100	3.84988200	1.48310000
C	-3.40738500	3.21000000	-0.90984700
H	-4.31464500	2.70361000	-0.56432600
H	-3.60747500	3.61015400	-1.91058300
H	-2.61798300	2.46197200	-0.99886800
O	-0.30749800	3.83692800	1.36256900
C	1.59202300	4.53472000	0.24852900
C	0.63763900	3.34265000	0.46575800
O	0.11519800	2.84916300	-0.67769100
O	1.37568700	2.23615500	1.06551400
C	2.69544800	2.51826200	1.54031600
C	3.48555000	3.08962500	0.35903600
O	4.57061900	2.71006900	-0.01826600
O	2.83351300	4.05863300	-0.32510500
H	1.80860900	4.97731600	1.22701400
H	2.65090500	3.28018300	2.33547300
C	1.02307200	5.60140300	-0.66823400
H	0.08275500	5.93179900	-0.21734800
C	3.28663300	1.23568100	2.10912100
H	3.44309400	0.51974300	1.30219200
H	2.52942300	0.80408800	2.77042900
H	0.76405500	5.13912600	-1.62464300
H	-1.47854000	2.15762500	0.95429000
H	-1.37239100	2.64104300	2.65599100
H	-2.64305100	4.71567600	2.15265000
H	-3.56848200	3.24698600	1.84279700
C	-3.00035900	4.32960100	0.05155200
H	-2.10249100	4.82932600	-0.32900800
H	-3.79208100	5.08895300	0.08400000
C	4.59368800	1.45688600	2.87055300
H	4.45928500	2.14691600	3.71191700
H	4.95794600	0.50650700	3.27088900
H	5.36232700	1.86168900	2.20949600
C	1.97403000	6.78141400	-0.87110200
H	2.91782000	6.45091500	-1.31263200
H	1.52873900	7.53011800	-1.53286700
H	2.20397400	7.27263900	0.08090200

TS2

C	7.68210800	-1.79202600	-0.83506600
C	7.04421000	-0.67568500	-1.31129700
C	5.64277600	-0.50290100	-1.13045300
C	4.88021400	-1.49968800	-0.43042600
C	5.57964900	-2.64993100	0.03664600
C	6.93157700	-2.79037000	-0.16071500
H	5.53164400	1.38215300	-2.16512400
H	8.75052000	-1.92007800	-0.97634400
H	7.59591700	0.09826700	-1.83822400
C	4.96958300	0.61960800	-1.63097900
C	3.48108700	-1.32978200	-0.23004400
H	5.01927800	-3.42652800	0.54296200
H	7.43564000	-3.68197900	0.20088000
C	2.81655900	-0.20869000	-0.75871600
C	3.59983500	0.78150800	-1.46435600
C	-7.08565900	0.49483300	1.49769600
C	-6.22263200	0.43245300	2.56687400
C	-4.91834700	-0.10875700	2.42482900
C	-4.49545900	-0.60939800	1.14893300
C	-5.41368100	-0.52445200	0.06506100
C	-6.66975500	0.01245700	0.23454500
H	-4.33132300	0.23398700	4.47881100
H	-8.07946700	0.91463600	1.61781800
H	-6.52380500	0.80543400	3.54225900

C	-4.00727500	-0.13951900	3.51124200	Al	0.30392100	1.28748900	-0.84866000
C	-3.16679100	-1.12253900	0.99259100	H	3.58050000	2.43371600	-2.84914900
H	-7.34782800	0.06878900	-0.61170600	H	-1.00381900	-0.09685600	-4.34031400
C	-2.31334700	-1.11650700	2.08369900	H	0.73576000	1.61659100	-4.57120100
C	-2.73513900	-0.62691800	3.34685100	H	1.85164700	3.51510500	-3.66769300
H	-2.03988600	-0.64946600	4.17852500	H	0.53705200	3.85610700	-2.51061300
C	2.72431200	-2.29690300	0.60964000	H	1.91293800	-2.45149600	-1.97224500
C	1.67478600	-3.10164400	0.07451700	H	-5.10656300	-0.88359900	-0.90869400
C	3.02347900	-2.39537800	1.96761600	C	-0.00188600	-0.80870500	2.43841100
C	1.33946000	-3.08515500	-1.30642000	H	-0.24535000	0.25269200	2.35933200
C	0.92717700	-3.96529200	0.93835400	C	4.43339800	-1.70317200	3.77789300
C	2.31590600	-3.28361600	2.81234800	H	3.63580600	-1.45128600	4.48868400
C	0.31235000	-3.85732900	-1.79222000	H	-0.66663000	2.63723100	-4.18582900
C	-0.14524400	-4.72935100	0.40755700	O	-1.06098600	-1.60823100	1.90710000
C	1.28190800	-4.03503300	2.30443600	O	4.00682600	-1.56355700	2.43568300
H	2.56154700	-3.35017300	3.86462800	H	5.25706400	-0.99931900	3.90319000
C	-0.45346000	-4.67790900	-0.92879900	H	4.79224100	-2.71871400	3.98731300
H	0.07021700	-3.82768300	-2.84914100	H	0.86274300	-1.01097400	1.81468100
H	-0.72198100	-5.35333400	1.08447200	H	0.21885100	-1.07947100	3.47856200
H	0.71837600	-4.69281600	2.95976600	C	-2.74471300	2.48002300	1.13011300
H	-1.28462200	-5.25087000	-1.32543200	C	-4.01150400	3.12877900	0.59525700
C	-2.68415900	-1.54252300	-0.35018700	C	-3.86613700	2.29848100	-1.83011500
C	-3.22486200	-2.68665900	-0.99097500	H	-4.68863800	1.59567000	-1.66496600
C	-1.74382600	-0.73595200	-1.01684500	H	-3.89694200	2.61549600	-2.87855000
C	-4.04417000	-3.62263900	-0.29461500	H	-2.92625500	1.76927400	-1.67003000
C	-2.94773500	-2.94651000	-2.37816300	O	-1.65664600	3.45639600	1.21343900
C	-1.56404000	-0.94276800	-2.43757200	C	0.17169800	4.69153200	0.51327100
C	-4.53465900	-4.74119300	-0.92225600	C	-0.68476200	3.44274000	0.30781300
H	-4.25393700	-3.44566900	0.75376400	O	-0.82448600	2.90954400	-0.84566100
C	-3.48560100	-4.10737100	-3.00120500	O	0.57719000	2.11387600	0.87175400
C	-2.16229000	-2.02236400	-3.07889700	C	1.71073200	2.64411000	1.52719500
C	-4.25819800	-4.99253700	-2.29279000	C	2.36180600	3.67066100	0.59093000
H	-5.14109700	-5.44764200	-0.36320300	O	3.53366500	3.72451700	0.30659600
H	-3.26182000	-4.28198100	-4.05042000	O	1.49766700	4.54016100	-0.01183600
H	-2.01669100	-2.15195700	-4.14925600	H	0.22237400	4.89502900	1.58758100
H	-4.65829500	-5.88072400	-2.77119900	H	1.34714800	3.19233900	2.41317500
C	2.96973200	1.89325700	-2.11827000	C	-0.46188100	5.88320400	-0.20326300
N	1.73677200	2.24463000	-1.96729600	H	-1.49174400	5.97220700	0.15748400
N	-0.29548900	1.08325000	-2.80686000	C	2.70762900	1.59339100	2.01580800
C	1.11388900	3.06927000	-2.99140500	H	3.27162100	1.19592400	1.17414900
C	0.18362500	2.09844500	-3.75408100	H	2.13240300	0.76549500	2.43294200
O	1.51974300	-0.07060300	-0.61184700	H	-0.51686300	5.64866900	-1.27171900
O	-1.13439900	0.23685600	-0.38819700	H	-2.41239900	1.63363400	0.53610600
C	-0.91183700	0.04297800	-3.25645700	H	-2.88420200	2.14949600	2.16011000

H	-4.22889500	4.01891300	1.19926500
H	-4.82927000	2.42074200	0.77624900
C	-3.97984500	3.50370100	-0.89341100
H	-3.14436000	4.18757000	-1.08108200
H	-4.89572800	4.06248800	-1.12358900
C	3.67826900	2.14358600	3.06175700
H	3.14866400	2.51987100	3.94537000
H	4.36033100	1.35463600	3.39021500
H	4.27919000	2.95634600	2.64733300
C	0.30705400	7.18425000	0.02509600
H	1.33765100	7.09049500	-0.32493000
H	-0.16614100	8.01231600	-0.51032800
H	0.33338500	7.44449100	1.08887100

INT4

C	8.03609000	-0.97423500	-0.75291400
C	7.27029100	0.04215800	-1.26463400
C	5.85955600	0.05767800	-1.07687000
C	5.22548700	-0.99701800	-0.33523600
C	6.05420200	-2.03817900	0.17050500
C	7.41287100	-2.02639500	-0.03318000
H	5.52536200	1.88276900	-2.17096700
H	9.11138000	-0.98166600	-0.89974100
H	7.72652500	0.85403200	-1.82472300
C	5.05696800	1.07954000	-1.60672400
C	3.81639800	-0.98583600	-0.13521300
H	5.58898000	-2.85216300	0.71339800
H	8.02091300	-2.83619500	0.35933900
C	3.03184000	0.03555400	-0.68466400
C	3.67889100	1.09118600	-1.42846400
C	-6.95865300	-0.98425500	1.63092200
C	-6.07628200	-0.79707000	2.66891100
C	-4.68349200	-1.00639700	2.49443500
C	-4.18327500	-1.43079100	1.21897100
C	-5.12542100	-1.59779000	0.16423600
C	-6.47032900	-1.38391300	0.36525800
H	-4.14456400	-0.44985800	4.51388400
H	-8.02159900	-0.81910900	1.77602200
H	-6.43150500	-0.47753100	3.64505400
C	-3.76462000	-0.77240100	3.54839500
C	-2.77256000	-1.61700700	1.03532600
H	-7.16270800	-1.51723100	-0.46050700
C	-1.91720000	-1.37098700	2.10051900
C	-2.41694200	-0.94462200	3.35822800
H	-1.72121800	-0.76472500	4.16944600
C	3.15495300	-2.02244400	0.70297400
C	2.22564300	-2.95201000	0.14966900
C	3.39885700	-2.04324700	2.07356500
C	1.93606800	-2.99511300	-1.24131100
C	1.54751800	-3.87567700	1.00808600
C	2.76027300	-2.98425000	2.91707200
C	1.01626500	-3.88347900	-1.74397200
C	0.59353500	-4.77107300	0.45727600
C	1.84661200	-3.86649100	2.38955600
H	2.96357600	-2.99299700	3.98040600
C	0.32720000	-4.77760400	-0.88940300
H	0.80180700	-3.89362700	-2.80762200
H	0.06947800	-5.44745600	1.12679800
H	1.33524800	-4.56897000	3.04132900
H	-0.41647600	-5.45108000	-1.30061900
C	-2.24043400	-1.91889300	-0.31887700
C	-2.57384500	-3.11946000	-0.99833200
C	-1.47353900	-0.94011400	-0.97359200
C	-3.18466800	-4.21753900	-0.32505600
C	-2.29057700	-3.27074000	-2.40159400
C	-1.29746300	-1.05158800	-2.40286800
C	-3.48337900	-5.38101800	-0.98957800
H	-3.39031000	-4.12211800	0.73456600
C	-2.62681400	-4.48684200	-3.06156300
C	-1.70679700	-2.19468000	-3.08274600
C	-3.20714600	-5.52344100	-2.37611500
H	-3.93370400	-6.20788200	-0.44855300
H	-2.40468700	-4.57803900	-4.12153100
H	-1.57120700	-2.25436700	-4.16035500
H	-3.45424900	-6.45002200	-2.88419400
C	2.92224500	2.13995100	-2.06568300
N	1.66067700	2.33206700	-1.89762100
N	-0.37687400	1.17298000	-2.64933200
C	0.91238500	3.22492300	-2.77115600
C	-0.07423100	2.31013600	-3.52585800
O	1.72215600	0.00877200	-0.52270000
O	-1.01942000	0.10609700	-0.32742300
C	-0.85064600	0.08266200	-3.16232600
Al	0.38814400	1.18013500	-0.77623500
H	3.46915000	2.78714400	-2.75933200
H	-0.96302800	0.01611700	-4.25015200
H	0.36235500	1.95389300	-4.46722600
H	1.56314900	3.77390400	-3.45957600

H	0.35334500	3.93577300	-2.16165300
H	2.45566000	-2.31105400	-1.90180000
H	-4.77183000	-1.89255600	-0.81458200
C	0.28963500	-0.60113700	2.47400800
H	-0.12037200	0.40406400	2.34669100
C	4.55279900	-1.06362400	3.93155200
H	3.65723000	-0.89018600	4.54202500
H	-0.99889700	2.85134200	-3.74484600
O	-0.58983100	-1.57432100	1.90637500
O	4.25127900	-1.08253400	2.54890000
H	5.24758400	-0.23480300	4.07314300
H	5.03262300	-1.99505500	4.25821600
H	1.21432800	-0.67917100	1.91008400
H	0.48767900	-0.80957600	3.53304300
C	-3.43295700	1.97059500	0.75325600
C	-4.83164400	2.17242000	0.19224300
C	-4.31367600	1.39337500	-2.19238800
H	-4.76316800	0.41932000	-1.97168800
H	-4.48061000	1.60981700	-3.25319000
H	-3.23666700	1.31646800	-2.03417000
O	-2.72164500	3.22901400	0.96310900
C	-1.16431100	4.90664700	0.56051800
C	-1.92141900	3.69997200	0.00951300
O	-1.86810000	3.32681100	-1.14747400
O	0.17641400	2.29204300	0.58497900
C	1.04742500	3.18685800	1.21578000
C	1.22834500	4.41235400	0.30345400
O	2.28153900	4.77516400	-0.16752900
O	0.10250200	5.08480300	-0.08581800
H	-1.02584300	4.76655200	1.63483000
H	0.56054400	3.53161900	2.14425700
C	-1.97073100	6.18195100	0.30208300
H	-2.97013000	6.03178600	0.72513500
C	2.39795300	2.58377100	1.61300100
H	2.99888900	2.41616800	0.71791500
H	2.19445900	1.60234000	2.05126100
H	-2.08995700	6.28643900	-0.78213400
H	-2.80732300	1.32802400	0.13885000
H	-3.48208200	1.54228900	1.75486600
H	-5.32930800	2.95900000	0.77326900
H	-5.38205700	1.24532300	0.38946000
C	-4.91251700	2.49057600	-1.30800800
H	-4.40432400	3.43774200	-1.51698000
H	-5.96882200	2.63645700	-1.56735000
C	3.18248600	3.45207000	2.59712000
H	2.60887900	3.63537700	3.51364800
H	4.11698800	2.96022900	2.88324100
H	3.43353100	4.41770800	2.15109300
C	-1.31216400	7.42668300	0.89533700
H	-0.31202000	7.56807400	0.47879600
H	-1.90478500	8.32033600	0.67950700
H	-1.21824200	7.34125100	1.98363500

INT5

C	-6.53698300	-4.41248300	0.96126200
C	-5.36613300	-4.73543300	0.32473000
C	-4.40989400	-3.73158300	0.00077500
C	-4.67625200	-2.35758300	0.33625400
C	-5.89970700	-2.06574000	1.00765700
C	-6.79789000	-3.06031900	1.30775800
H	-3.00071100	-5.07037200	-0.92538500
H	-7.26139200	-5.18267000	1.20627300
H	-5.14443900	-5.76570100	0.05866800
C	-3.20459800	-4.03878800	-0.64596000
C	-3.73830800	-1.34498600	0.00488000
H	-6.10821400	-1.03823800	1.28022600
H	-7.72224000	-2.81087200	1.82037200
C	-2.52096000	-1.67187500	-0.60703700
C	-2.26258100	-3.05692600	-0.93661100
C	3.17920300	1.73602400	4.05624600
C	2.00988400	2.39745800	4.35714500
C	1.19078900	2.93552100	3.33243200
C	1.57504500	2.76950000	1.96330700
C	2.80155600	2.10657400	1.69066300
C	3.58142500	1.60232100	2.70794900
H	-0.28192000	3.80689300	4.66136800
H	3.79591400	1.32455300	4.84913300
H	1.69514500	2.52040600	5.39001700
C	-0.00080800	3.65160100	3.62353900
C	0.73364400	3.26549500	0.91413500
H	4.50002000	1.07704900	2.46910100
C	-0.45375000	3.90246500	1.25089700
C	-0.79366900	4.12614600	2.61310500
H	-1.71271500	4.66579200	2.81411300
C	-3.99055200	0.08021600	0.34796200
C	-4.93325200	0.87249200	-0.36870000
C	-3.25245900	0.65974900	1.37294100
C	-5.69057900	0.35234400	-1.45538900

C	-5.12068900	2.24954000	-0.01182500
C	-3.43798500	2.01952700	1.72495600
C	-6.58399900	1.14304400	-2.13986500
C	-6.05251500	3.03612800	-0.73875400
C	-4.35339200	2.78885800	1.04913700
H	-2.84931500	2.45144300	2.52281700
C	-6.77288200	2.49949100	-1.77997100
H	-7.15021500	0.72430700	-2.96673900
H	-6.18359300	4.07704100	-0.45427000
H	-4.48267900	3.83428200	1.31458500
H	-7.48291900	3.11095900	-2.32830300
C	1.18272600	3.14727800	-0.49953400
C	1.73952100	4.27471900	-1.15969200
C	1.04174100	1.92071100	-1.16435300
C	1.89854800	5.53349700	-0.51115300
C	2.16105500	4.17251500	-2.53140400
C	1.45792100	1.83491100	-2.54262400
C	2.43756700	6.60835600	-1.17549200
H	1.58753700	5.62776700	0.52215200
C	2.71599900	5.30718600	-3.18772000
C	2.00476900	2.94192100	-3.18471300
C	2.85281100	6.50208200	-2.52881000
H	2.54863000	7.55648000	-0.65786600
H	3.02729200	5.20487300	-4.22391100
H	2.31268200	2.85306700	-4.22439200
H	3.27519700	7.36465100	-3.03397500
C	-1.10074900	-3.42236700	-1.70449500
N	-0.07702400	-2.65498500	-1.86230200
N	0.78648900	-0.47424000	-2.88360900
C	0.89138900	-2.88753100	-2.91553200
C	0.78396400	-1.62866000	-3.78780700
O	-1.66614600	-0.71819700	-0.92177900
O	0.51800400	0.89553700	-0.54133600
C	1.32013900	0.62381900	-3.30178500
Al	0.13657300	-0.84589500	-0.94635600
H	-1.12997100	-4.38704100	-2.22445400
H	1.71563300	0.65947600	-4.32296700
H	-0.17146600	-1.65492000	-4.32627600
H	0.67941100	-3.79415800	-3.49459000
H	1.89274200	-2.95337100	-2.48348700
H	-5.54670400	-0.68381900	-1.73923100
H	3.11165100	1.99351700	0.65822700
C	-1.69386400	3.64199500	-0.81067100
H	-1.61254000	2.56883600	-0.62220000
C	-1.22807600	0.45603000	2.61244500
H	-0.70573500	1.07508600	1.87994200
H	1.59811600	-1.56966000	-4.51861200
O	-1.36071000	4.39983500	0.36255100
O	-2.38101100	-0.14826800	2.03104600
H	-0.58653900	-0.36985800	2.90395800
H	-1.47993600	1.05339300	3.49601100
H	-1.05849100	3.92414200	-1.65363900
H	-2.73333200	3.88446000	-1.03527200
C	1.74015800	-1.23381500	1.28474300
C	2.84671900	-1.12690200	0.24558700
O	2.61121700	-1.16909300	-0.95630900
O	0.57317000	-1.58084900	0.62808900
H	1.66899000	-0.24163700	1.76048900
C	2.13401300	-2.25798300	2.36843000
H	2.36714000	-3.20938200	1.88216600
H	3.05441900	-1.91420800	2.85125700
C	1.02872000	-2.45737000	3.40106700
H	0.87496300	-1.54772800	3.99148600
H	0.08756500	-2.70867200	2.90606500
H	1.28436300	-3.26303700	4.09696400
C	4.28002500	-4.79126500	-0.68553200
C	3.17005400	-5.48089800	0.08979000
C	0.68938600	-5.51341600	0.68886200
H	0.89669200	-5.24344000	1.72947100
H	-0.29422700	-5.10471300	0.44206300
H	0.63261300	-6.60661700	0.63153200
O	4.38707500	-3.44110800	-0.17070200
C	5.14572700	-1.17794600	-0.24991300
C	5.11915900	-2.57185100	-0.88025100
O	5.73964200	-2.83940700	-1.88526100
O	4.09312700	-1.01959600	0.72130000
H	4.99737800	-0.46653700	-1.06536200
C	6.47077600	-0.91763200	0.46591100
H	6.46011600	0.12046900	0.81543200
H	7.26153700	-0.99692100	-0.28702700
H	4.07246900	-4.74142400	-1.75878700
H	5.24784700	-5.28426200	-0.55246900
H	3.37457500	-5.36507800	1.16143400
H	3.22528500	-6.55653800	-0.12113500
C	1.76794400	-4.95493900	-0.23907700
H	1.53348300	-5.21272500	-1.28091100
H	1.74895500	-3.86393800	-0.17829700
C	6.73623100	-1.87107700	1.63272800

H	6.80800100	-2.90865800	1.29223900
H	7.67696200	-1.61626100	2.12813800
H	5.93280900	-1.81698200	2.37134000

***re*-enantioface: *R,R*-Al-O^{n}Bu + L-EG**

INT1

C	7.75122800	-1.20327600	-0.82193800
C	6.92516200	-1.85067000	0.06158700
C	5.62004200	-1.35515600	0.33413500
C	5.15340800	-0.16398000	-0.31710300
C	6.04246800	0.48000200	-1.22535300
C	7.29742900	-0.02517000	-1.46831800
H	5.10634000	-2.87829900	1.76573600
H	8.74557500	-1.58577900	-1.02877100
H	7.25153300	-2.75536800	0.56765400
C	4.76022700	-1.99024800	1.24136700
C	3.84512700	0.33687900	-0.05468500
H	5.71158300	1.38060500	-1.72760500
H	7.95350900	0.48543700	-2.16705800
C	2.97771700	-0.33890200	0.80898000
C	3.47537000	-1.52251200	1.47957500
C	-6.29682800	0.71648700	-2.57186000
C	-5.18229200	0.83709800	-3.37052000
C	-3.92959500	1.21539000	-2.82647200
C	-3.81068800	1.48680400	-1.42555800
C	-4.97442700	1.30941600	-0.62612200
C	-6.18069100	0.94432900	-1.18281400
H	-2.85757800	1.13445900	-4.70772200
H	-7.25443400	0.43803900	-3.00071400
H	-5.24429200	0.64649000	-4.43861000
C	-2.77703000	1.34317100	-3.64476400
C	-2.55412000	1.92202400	-0.86718000
H	-7.05089100	0.82703200	-0.54370800
C	-1.46283100	2.04936200	-1.71985800
C	-1.58948300	1.74734800	-3.10630100
H	-0.70812300	1.89129200	-3.72041400
C	3.42538000	1.58386900	-0.75344900
C	3.77424300	2.86078100	-0.22945000
C	2.81917800	1.50097600	-1.99963100
C	4.38683600	3.00929700	1.04557600
C	3.52377700	4.04137300	-1.00361800
C	2.55967000	2.66630500	-2.76245300
C	4.72137600	4.25246300	1.52907300
C	3.88232200	5.30904500	-0.47456300
C	2.91564200	3.90107500	-2.27535200
H	2.07603800	2.58856600	-3.72733800
C	4.46787300	5.41760400	0.76475900
H	5.18409300	4.34416700	2.50730000
H	3.68231700	6.19527600	-1.07113900
H	2.71757900	4.79205100	-2.86446700
H	4.73704900	6.39226700	1.15987400
C	-2.49741000	2.16157300	0.60118300
C	-3.22509100	3.23023000	1.19112300
C	-1.75788600	1.28211400	1.40931100
C	-3.88661400	4.21886400	0.40386200
C	-3.30492600	3.35979700	2.62280100
C	-1.88605600	1.39307600	2.84211800
C	-4.58312800	5.24596000	0.99198600
H	-3.82487400	4.14718300	-0.67519800
C	-4.04558400	4.43208700	3.19743300
C	-2.65117500	2.40670400	3.41351900
C	-4.67447200	5.35694400	2.40482300
H	-5.07183200	5.98754800	0.36709400
H	-4.09529900	4.50292500	4.28074800
H	-2.73532700	2.46525800	4.49649400
H	-5.23430100	6.17397500	2.84823600
C	2.69101700	-2.21532600	2.46870800
N	1.43305900	-2.02424100	2.64627400
N	-0.47587500	-0.51997400	3.36071700
C	0.72153200	-2.58819500	3.77544700
C	0.02820500	-1.39346500	4.43429800
O	1.75463800	0.12054000	1.03090100
O	-0.97815800	0.38359800	0.87568000
C	-1.27134400	0.43988900	3.71390000
Al	0.26401800	-0.81042200	1.48074000
H	3.22367200	-2.92237200	3.11535300
H	-1.51457200	0.54579600	4.77659200
H	0.75304600	-0.83359500	5.03840500
H	1.38468800	-3.10684500	4.47792000
H	-0.04157100	-3.27478100	3.40646800
H	4.58118000	2.11860200	1.63245600
H	-4.90733600	1.46487800	0.44230700
C	0.10149800	3.20986200	-0.23823000
H	0.29360700	2.54746200	0.60533700
C	1.96567000	0.07203300	-3.72851600
H	0.95830700	0.49809800	-3.77321700
H	-0.79068000	-1.72354200	5.08038500

O	-0.20824600	2.45920200	-1.41227400
O	2.54326500	0.24369100	-2.44531000
H	1.90238800	-1.00511300	-3.87981000
H	2.59015900	0.51261200	-4.51565900
H	-0.69481200	3.91885900	-0.00026700
H	1.01365400	3.75169100	-0.47982200
C	-0.13765300	-1.83626100	-1.01875100
C	0.63367200	-2.87399900	-1.83345900
C	2.89216900	-3.98526000	-2.26873600
H	2.88652800	-3.71348100	-3.33127600
H	3.93835700	-4.04071200	-1.94864100
H	2.46101400	-4.99042500	-2.18912300
O	-0.07811900	-2.13381600	0.36927300
C	-1.90296900	-4.71450700	0.84718200
C	-2.35037000	-3.36852000	1.41058100
O	-2.08091800	-3.00246500	2.53402100
O	-3.16469300	-2.64030200	0.63041700
C	-3.75240100	-3.28153900	-0.53055100
C	-2.68589700	-4.01975700	-1.32922200
O	-2.61862500	-4.03686200	-2.53321600
O	-1.79320600	-4.70747700	-0.58861900
H	-2.68171400	-5.44553700	1.12002400
H	-4.46967200	-4.03421000	-0.16626300
C	-0.55475200	-5.16075200	1.39779100
H	-0.65215500	-5.20987200	2.48624100
C	-4.48916200	-2.21950900	-1.32893700
H	-3.76036400	-1.58873300	-1.84444300
H	-5.00932200	-1.57927500	-0.61264400
H	0.16001300	-4.37018600	1.15383800
H	-1.18551100	-1.79920600	-1.35211700
H	0.28174000	-0.84094100	-1.22753000
H	0.54638500	-2.60820200	-2.89670500
H	0.15558700	-3.85256800	-1.72463300
C	2.10655700	-2.97501000	-1.43231400
H	2.15689500	-3.25572600	-0.37543600
H	2.57608100	-1.98891100	-1.50845300
C	-5.48249200	-2.82010200	-2.32535200
H	-6.22643100	-3.44465400	-1.81588200
H	-6.01426200	-2.01836600	-2.84063000
H	-4.96833100	-3.43133200	-3.06904700
C	-0.10421400	-6.51005500	0.83939600
H	0.00975300	-6.46414700	-0.24583100
H	0.85937700	-6.79670200	1.27039300
H	-0.82367000	-7.30391000	1.07129300

TS1

C	7.37357700	-0.90655700	-1.29737500
C	6.66310900	-1.48060000	-0.27447300
C	5.40173300	-0.95491800	0.12200600
C	4.85221900	0.18724700	-0.55533700
C	5.63251100	0.76531800	-1.59876400
C	6.84746700	0.23372900	-1.95750400
H	5.11122700	-2.32184400	1.75876300
H	8.33323500	-1.31336700	-1.59954700
H	7.04933400	-2.34908700	0.25245200
C	4.67889200	-1.50041400	1.19144800
C	3.57500300	0.69913400	-0.18662000
H	5.25112300	1.63954000	-2.11139000
H	7.41672300	0.69442500	-2.75955500
C	2.81268000	0.07711100	0.81216500
C	3.42366300	-1.02248200	1.54001500
C	-6.43936900	-0.92955600	-2.26627700
C	-5.37193000	-0.81042100	-3.12722900
C	-4.18619300	-0.14107100	-2.73492600
C	-4.09590700	0.44973000	-1.43424200
C	-5.20548700	0.27644400	-0.56026500
C	-6.34177500	-0.39068600	-0.96407800
H	-3.11020600	-0.52792000	-4.57576900
H	-7.34327900	-1.44388000	-2.57738100
H	-5.41651200	-1.23809200	-4.12543800
C	-3.06098200	-0.06186000	-3.59575700
C	-2.91620200	1.17490400	-1.03628500
H	-7.17082600	-0.50312100	-0.27166800
C	-1.85224000	1.24368300	-1.93199100
C	-1.93284100	0.59670800	-3.19828700
H	-1.06819300	0.67623400	-3.84656900
C	3.06847500	1.91035200	-0.89109900
C	3.16914700	3.19599200	-0.28652100
C	2.61324100	1.81154200	-2.20071600
C	3.60101400	3.36453300	1.05842800
C	2.84473300	4.36916300	-1.04467000
C	2.30005500	2.96833800	-2.95595400
C	3.68913000	4.61536400	1.62190400
C	2.94823600	5.64610400	-0.43220400
C	2.42746900	4.21500100	-2.38871000
H	1.95568100	2.87579900	-3.97772200
C	3.35816900	5.77174400	0.87353600
H	4.01644000	4.72073100	2.65215000

H	2.69435900	6.52472300	-1.01941800
H	2.18846500	5.10080300	-2.97048400
H	3.43180300	6.75259400	1.33297700
C	-2.88961500	1.76811100	0.33009400
C	-3.75145300	2.84512400	0.67419900
C	-2.01028000	1.22942100	1.28330700
C	-4.58909400	3.48475200	-0.28651600
C	-3.78748500	3.34539800	2.02261600
C	-2.05522700	1.74152500	2.63256400
C	-5.41508500	4.52305000	0.07013700
H	-4.56118400	3.13674800	-1.31178500
C	-4.66240100	4.41667600	2.35816100
C	-2.93932600	2.75941900	2.97323700
C	-5.46426000	4.99523100	1.40764800
H	-6.03990800	4.99255400	-0.68388700
H	-4.67491800	4.77078400	3.38564100
H	-2.96131700	3.12432300	3.99798400
H	-6.12715900	5.81373200	1.66897700
C	2.81583500	-1.51324800	2.74858200
N	1.56613600	-1.36682100	3.02266600
N	-0.26312300	0.35258600	3.50026400
C	1.07374000	-1.46512600	4.38407000
C	0.51572900	-0.06222800	4.67100000
O	1.61045700	0.51938700	1.12280500
O	-1.16304900	0.30117700	0.93548400
C	-1.19875500	1.22582200	3.66456000
Al	0.23271800	-0.57099600	1.70466500
H	3.47978100	-1.96392800	3.49573400
H	-1.37277200	1.62948500	4.66880300
H	1.35782600	0.62991300	4.79733200
H	1.86200500	-1.73105500	5.09814200
H	0.26745700	-2.19931700	4.42550500
H	3.85858600	2.48257400	1.63287800
H	-5.14814700	0.67454600	0.44431200
C	-0.46791600	2.92077800	-0.80889200
H	-0.15191800	2.50042400	0.14531800
C	2.09726600	0.37539200	-4.04689800
H	1.08782900	0.77537300	-4.19082200
H	-0.08872100	-0.04642000	5.58508000
O	-0.66698100	1.88637500	-1.77567200
O	2.53558700	0.55051500	-2.71197400
H	2.08624200	-0.70149400	-4.21442100
H	2.78148100	0.84841600	-4.76296100
H	-1.36722200	3.52740400	-0.68754000
H	0.33385800	3.53494500	-1.21009400
C	0.24367700	-1.59302600	-0.92180700
C	1.01866800	-2.57360200	-1.80115800
C	3.28982300	-3.56484000	-2.41731700
H	3.19023200	-3.27107600	-3.46960100
H	4.35847300	-3.56619900	-2.17628500
H	2.92125000	-4.59385700	-2.33136500
O	0.36392000	-1.90128000	0.45887200
C	-0.70726300	-4.30390300	1.50882800
C	-1.28265600	-2.89550600	1.56591000
O	-1.08035500	-2.12853100	2.52104300
O	-2.30195700	-2.65320100	0.74200000
C	-2.80460500	-3.73645900	-0.08690300
C	-1.65528800	-4.53788500	-0.68579700
O	-1.62891400	-4.94834600	-1.81926900
O	-0.63346000	-4.79208600	0.15682200
H	-1.42240100	-4.93846800	2.06096200
H	-3.37036200	-4.41165900	0.57395400
C	0.67790700	-4.44850500	2.11515700
H	0.63220700	-4.05849900	3.13519600
C	-3.73187800	-3.13080200	-1.12533300
H	-3.12756000	-2.62831800	-1.88376600
H	-4.32073400	-2.36011800	-0.62274300
H	1.34103300	-3.79944300	1.54170700
H	-0.82057500	-1.59447500	-1.20418900
H	0.60648400	-0.57500300	-1.11183700
H	0.85553300	-2.27495700	-2.84719400
H	0.60176300	-3.58098300	-1.70562400
C	2.51700500	-2.61544400	-1.50172200
H	2.65654800	-2.91652200	-0.45878100
H	2.92889600	-1.60701400	-1.58941100
C	-4.65439600	-4.16728100	-1.76859600
H	-5.27660800	-4.66491300	-1.01491500
H	-5.32402300	-3.67163400	-2.47426900
H	-4.08169700	-4.92733800	-2.30293400
C	1.16804000	-5.89620000	2.11289900
H	1.21711100	-6.28803500	1.09440700
H	2.16857200	-5.96101700	2.55012400
H	0.50645400	-6.54784000	2.69543100

INT2

C	7.31517100	-0.74514400	-1.19865900
C	6.63888400	-1.25272500	-0.11870300
C	5.36560100	-0.73780200	0.25256500

C	4.76809600	0.32301000	-0.51199300
C	5.51561500	0.83837900	-1.61058900
C	6.74281100	0.31793500	-1.94343400
H	5.15087600	-1.95922200	2.01351300
H	8.28392400	-1.14462000	-1.48077100
H	7.06275300	-2.05906600	0.47389500
C	4.67823700	-1.21229900	1.37928100
C	3.47452200	0.81368200	-0.17410000
H	5.09873200	1.65549800	-2.18609800
H	7.28626000	0.72824300	-2.78951300
C	2.74496100	0.24050400	0.87734700
C	3.41043600	-0.75108400	1.70277100
C	-6.07843800	-1.51128400	-2.42466100
C	-4.96561000	-1.32419800	-3.21220200
C	-3.89622800	-0.49848800	-2.78406700
C	-3.97157300	0.17217100	-1.52131500
C	-5.11920500	-0.07770100	-0.71788600
C	-6.14265700	-0.88834800	-1.15841400
H	-2.65514900	-0.85439500	-4.52432700
H	-6.89403300	-2.14197000	-2.76420400
H	-4.88398600	-1.81413200	-4.17883300
C	-2.73017200	-0.33071800	-3.57548500
C	-2.91314600	1.05201600	-1.09309200
H	-7.00571400	-1.05422400	-0.52054500
C	-1.80824300	1.20318700	-1.92587400
C	-1.72170300	0.48786900	-3.15357300
H	-0.82742000	0.64315700	-3.74593900
C	2.91983300	1.96298800	-0.94412800
C	2.96807700	3.28038600	-0.39936300
C	2.47109200	1.78944500	-2.24642300
C	3.39270600	3.53059900	0.93469800
C	2.60267300	4.40257400	-1.21318300
C	2.10723000	2.89444800	-3.05329200
C	3.43436400	4.81006700	1.43682500
C	2.65894500	5.71070900	-0.66436400
C	2.18693400	4.16959500	-2.54689800
H	1.75798800	2.73779600	-4.06575000
C	3.06203000	5.91526200	0.63373500
H	3.75665600	4.97694700	2.46044500
H	2.37362300	6.54953400	-1.29377200
H	1.91079600	5.01716900	-3.16779900
H	3.09875900	6.91943400	1.04457500
C	-3.02289700	1.68398300	0.25204600
C	-4.01978100	2.65442600	0.53582100
C	-2.12683700	1.27287600	1.25372700
C	-4.88290600	3.17391100	-0.47384800
C	-4.17603100	3.16747500	1.87171900
C	-2.29957500	1.78572100	2.59476400
C	-5.83999900	4.11288400	-0.17547100
H	-4.76778700	2.81315700	-1.48875800
C	-5.18557400	4.13330200	2.14544100
C	-3.31380000	2.69788400	2.87236200
C	-6.00480500	4.59789700	1.14867600
H	-6.48124200	4.49278900	-0.96532100
H	-5.28765000	4.49921800	3.16374200
H	-3.43190700	3.06909700	3.88821800
H	-6.77054500	5.33627600	1.36323100
C	2.82314400	-1.15415300	2.95532700
N	1.57833900	-0.97639400	3.22100500
N	-0.42800200	0.59550200	3.59077200
C	1.02451000	-1.08999000	4.55637200
C	0.33284000	0.26307100	4.79964100
O	1.51590900	0.63705400	1.13540900
O	-1.14602600	0.47225200	0.95217900
C	-1.44964600	1.38165500	3.68160300
Al	0.15862100	-0.36657600	1.89389600
H	3.49059700	-1.57531100	3.71631800
H	-1.71227300	1.79002700	4.66415300
H	1.10262500	1.02942100	4.95528500
H	1.78805100	-1.28917600	5.31760100
H	0.27843400	-1.88933700	4.55370600
H	3.68338700	2.68926300	1.55236600
H	-5.18382600	0.37799100	0.26121100
C	-0.70879400	3.09489300	-0.83293600
H	-0.42551500	2.77750000	0.17066500
C	2.27202400	0.26789500	-4.09069500
H	1.27773300	0.56347500	-4.44553600
H	-0.31068600	0.23108900	5.68592600
O	-0.71613000	1.98598100	-1.73074000
O	2.42994400	0.50179800	-2.70235000
H	2.39163000	-0.80755000	-4.22465600
H	3.03502000	0.79542700	-4.67608600
H	-1.67724500	3.59973600	-0.81702000
H	0.05095700	3.77078700	-1.21978700
C	0.30167500	-1.55212200	-0.94310400
C	1.12339300	-2.54630100	-1.74836400
C	3.41101500	-3.56771600	-2.22420900
H	3.31591300	-3.38661700	-3.30182000

H	4.47648600	-3.52518600	-1.97532000
H	3.05677600	-4.58695000	-2.03252600
O	0.36476700	-1.80839900	0.49251700
C	-0.20419400	-4.05291800	1.43776700
C	-0.68024300	-2.60866000	1.21566300
O	-0.78611200	-1.92731500	2.35925800
O	-1.84469400	-2.58113400	0.43241300
C	-2.45167800	-3.82928200	0.06994200
C	-1.39231100	-4.76147600	-0.50951700
O	-1.49751500	-5.40041000	-1.52860100
O	-0.24204700	-4.80703400	0.20542400
H	-0.92094000	-4.49719500	2.14127300
H	-2.86936300	-4.30749500	0.97173700
C	1.19883500	-4.18023400	2.00254500
H	1.22625000	-3.59437600	2.92375500
C	-3.57372200	-3.51637900	-0.90619800
H	-3.13192600	-3.21204000	-1.85818300
H	-4.10373400	-2.64799000	-0.50932200
H	1.89748500	-3.71406500	1.30361900
H	-0.74745600	-1.55229000	-1.23399000
H	0.68854500	-0.53938200	-1.05391400
H	0.97868000	-2.27874100	-2.80453400
H	0.72005300	-3.55636600	-1.63348700
C	2.61721200	-2.54039400	-1.41780000
H	2.75027800	-2.73637400	-0.34908500
H	3.01734300	-1.53991400	-1.59443000
C	-4.54129800	-4.68044900	-1.11516600
H	-5.00269600	-4.98792300	-0.16905600
H	-5.34462700	-4.37626100	-1.79133300
H	-4.03152900	-5.54367200	-1.54795500
C	1.59594000	-5.63173300	2.27305200
H	1.56555800	-6.22032500	1.35315400
H	2.61007700	-5.68646700	2.68014200
H	0.91933400	-6.10161500	2.99588100

INT3

C	7.44182000	1.40873300	1.42461100
C	6.54537700	2.24267100	0.80801900
C	5.26743500	1.76725500	0.39808800
C	4.89522400	0.39957200	0.65120500
C	5.87167800	-0.44181900	1.25957400
C	7.09752800	0.04826200	1.63763000
H	4.71548600	3.55763900	-0.66235400
H	8.41393700	1.77668600	1.73634300
H	6.79637100	3.28181900	0.61291600
C	4.38748300	2.57160400	-0.34083100
C	3.60703000	-0.06957800	0.27529200
H	5.63319200	-1.48564600	1.41839300
H	7.81908600	-0.61642700	2.10356700
C	2.66885200	0.80715300	-0.29816700
C	3.13036200	2.11622500	-0.72203400
C	-7.03115300	-1.68322800	0.24425800
C	-6.50393200	-1.17624400	1.40963000
C	-5.10569000	-1.17711200	1.64112100
C	-4.22216000	-1.73034100	0.65941800
C	-4.80140800	-2.22921700	-0.53968400
C	-6.16417900	-2.20689800	-0.74009200
H	-5.21551300	-0.14206100	3.54142000
H	-8.10340800	-1.67282300	0.07536400
H	-7.15181000	-0.75060800	2.17107400
C	-4.55187700	-0.59514100	2.81103900
C	-2.79761400	-1.74224500	0.88446800
H	-6.57525100	-2.59393600	-1.66778400
C	-2.32291200	-1.18876600	2.06574500
C	-3.20128600	-0.59395400	3.01269500
H	-2.75352200	-0.13752600	3.88820400
C	3.24396100	-1.50853700	0.38652500
C	3.01796900	-2.27846500	-0.79782200
C	3.13652600	-2.13472300	1.62196700
C	3.17006100	-1.73065100	-2.10268600
C	2.65132400	-3.65924200	-0.69279900
C	2.80345800	-3.50854600	1.72587500
C	2.97181500	-2.49975100	-3.22568100
C	2.43736000	-4.42183800	-1.87085800
C	2.54636700	-4.24192300	0.59268200
H	2.72052400	-3.97618200	2.69884400
C	2.59799400	-3.86075100	-3.11492100
H	3.11508400	-2.06138100	-4.20950600
H	2.14428700	-5.46234400	-1.76576600
H	2.26626400	-5.28788700	0.67487300
H	2.44500800	-4.45625400	-4.00994400
C	-1.93033200	-2.24217700	-0.22336300
C	-1.86357500	-3.62305100	-0.54750900
C	-1.24630300	-1.30285300	-1.00914700
C	-2.44526800	-4.62262400	0.28476200
C	-1.17560000	-4.05772900	-1.73299000
C	-0.64787300	-1.73534200	-2.25403000
C	-2.33257800	-5.95680400	-0.02586000

H	-2.97383700	-4.30788500	1.17728400
C	-1.08528200	-5.44634900	-2.02710800
C	-0.62727600	-3.08530600	-2.58328400
C	-1.64472600	-6.38135200	-1.19257000
H	-2.77701500	-6.69926100	0.63036100
H	-0.55692100	-5.74944000	-2.92699700
H	-0.16157600	-3.39794800	-3.51256500
H	-1.56798200	-7.43973200	-1.42005500
C	2.43766100	2.78974700	-1.79302500
N	1.24483600	2.47698100	-2.16502200
N	-0.04252500	0.47990700	-3.03962300
C	0.73518000	2.74560300	-3.49866700
C	0.47690400	1.34580400	-4.10697300
O	1.44573200	0.39791100	-0.52818100
O	-1.18997200	-0.05125100	-0.63166300
C	-0.13765500	-0.79696200	-3.22182000
Al	-0.05073700	1.26477400	-1.18859300
H	3.01769500	3.50770200	-2.38455400
H	0.17316500	-1.22417800	-4.18088500
H	1.41437700	0.93152400	-4.49888800
H	1.43765700	3.31806600	-4.11500300
H	-0.20718100	3.28925600	-3.40890500
H	3.47169500	-0.69526600	-2.20048000
H	-4.15378500	-2.62803900	-1.30980900
C	-0.03153300	-2.01646300	1.98610200
H	0.38615900	-1.69398800	1.03189000
C	3.42922900	-1.94307300	3.99869700
H	2.48769100	-2.41305000	4.30792600
H	-0.24101000	1.41536600	-4.93128000
O	-1.02091900	-1.11014300	2.46491600
O	3.32468000	-1.34054500	2.71819400
H	3.66146500	-1.13087200	4.68745800
H	4.23237000	-2.68941000	4.02679400
H	-0.42764500	-3.03134200	1.89827900
H	0.75184600	-1.99167800	2.74141400
C	-0.42696200	5.07855200	-0.45929200
C	-0.92364800	6.48947300	-0.74119100
C	-2.02471400	5.78012700	-2.95595800
H	-1.94073700	4.70390900	-2.77909400
H	-2.89407300	5.94490000	-3.60046000
H	-1.13625400	6.11175500	-3.50855600
O	-1.50020200	4.33031500	0.12098900
C	-2.73361200	2.37120100	0.55657600
C	-1.51591800	2.97307900	-0.13944000
O	-1.36266700	2.59088100	-1.41811700
O	-0.28851900	2.34483700	0.47365000
C	-0.26744300	1.92594400	1.85932700
C	-1.50274600	2.37523400	2.64219000
O	-1.46594700	2.48931700	3.84774600
O	-2.65258200	2.59981700	1.98706400
H	-2.71111800	1.29460400	0.37499700
H	-0.29924600	0.83226200	1.85800200
C	-4.03668100	2.98260500	0.06947300
H	-4.00774900	2.96145500	-1.02503600
C	1.03506500	2.41603200	2.49817500
H	0.90257200	3.44769200	2.84042800
H	1.78775600	2.42970500	1.70540400
H	-4.05119100	4.03328000	0.37480500
H	0.42135300	5.08847900	0.23636500
H	-0.10426000	4.58926400	-1.37966400
H	-0.10076200	7.04120800	-1.21655700
H	-1.13560500	6.99490000	0.20823400
C	-2.17338700	6.53657300	-1.63248600
H	-2.42064500	7.58700500	-1.82830900
H	-3.01575400	6.11450900	-1.07520800
C	1.51263700	1.52082900	3.64244300
H	1.73404800	0.51556600	3.27265800
H	2.43243400	1.92170900	4.08019600
H	0.75317200	1.45773700	4.42358600
C	-5.26953700	2.24281800	0.58712100
H	-5.26619600	2.20053800	1.67954400
H	-6.18732600	2.74562200	0.26683100
H	-5.29941400	1.21536200	0.21512800

TS2

C	7.06095600	2.87264500	1.41416900
C	6.01709800	3.50354500	0.78868400
C	4.86446300	2.77599900	0.37660200
C	4.77903800	1.36199600	0.63314300
C	5.90011600	0.74059800	1.25709600
C	6.99814500	1.47188000	1.63757300
H	3.94559100	4.42072900	-0.66279200
H	7.93630500	3.43223300	1.72765600
H	6.05171300	4.57138500	0.58992600
C	3.83435200	3.38339100	-0.35477600
C	3.61947000	0.63720500	0.24919800
H	5.87554400	-0.32889800	1.42412900
H	7.83597200	0.97118900	2.11400700

C	2.52491200	1.29497800	-0.33951000
C	2.69705100	2.68032600	-0.73699700
C	-6.46108900	-3.28585500	0.08539700
C	-6.09352100	-2.76110900	1.30323000
C	-4.74348300	-2.43016200	1.57917300
C	-3.73622800	-2.65999900	0.58806500
C	-4.15564500	-3.18917900	-0.66337400
C	-5.47745600	-3.49216300	-0.90668400
H	-5.13030200	-1.62479100	3.55356500
H	-7.49883300	-3.53170000	-0.11711800
H	-6.83718700	-2.57733000	2.07399800
C	-4.36648900	-1.83033100	2.80936300
C	-2.35475500	-2.34266400	0.85822800
H	-5.76538300	-3.89269300	-1.87419800
C	-2.04656500	-1.78631700	2.09206200
C	-3.06262600	-1.50608900	3.04954700
H	-2.74952300	-1.04362300	3.97897900
C	3.54674500	-0.83992300	0.40600200
C	3.51376700	-1.68352000	-0.74656700
C	3.52016300	-1.42233900	1.66719600
C	3.58224100	-1.16450700	-2.06999200
C	3.43160000	-3.10479800	-0.58847600
C	3.46876300	-2.83026800	1.82474300
C	3.56899400	-1.99925600	-3.16282600
C	3.40653900	-3.93865500	-1.73704500
C	3.40778700	-3.64498500	0.71934600
H	3.44707600	-3.26661800	2.81543900
C	3.47764600	-3.40317400	-3.00062000
H	3.63946900	-1.57866100	-4.16219500
H	3.32747600	-5.01233800	-1.59395600
H	3.34438600	-4.72214500	0.84231500
H	3.46847300	-4.05049500	-3.87246700
C	-1.37284400	-2.60188800	-0.23694300
C	-0.98987700	-3.93308200	-0.55945300
C	-0.90386000	-1.52836400	-1.00649000
C	-1.34740200	-5.04141300	0.26112600
C	-0.19788900	-4.19716400	-1.72841400
C	-0.19673600	-1.81338700	-2.23636500
C	-0.93126400	-6.31505700	-0.04698800
H	-1.94913700	-4.85910800	1.14390900
C	0.20796300	-5.52769600	-2.02187800
C	0.13497300	-3.12067000	-2.56535500
C	-0.14362500	-6.56896000	-1.19937600
H	-1.20970000	-7.14093500	0.60095400
H	0.81021600	-5.69869800	-2.90984000
H	0.68087000	-3.31435600	-3.48336900
H	0.17355600	-7.58193000	-1.42563700
C	1.86701900	3.21436100	-1.78521600
N	0.73228800	2.70591200	-2.13159300
N	-0.07591700	0.48093600	-3.00899500
C	0.21183200	2.85837400	-3.48032800
C	0.25610900	1.43195100	-4.07727900
O	1.42685600	0.63184100	-0.59897000
O	-1.13062000	-0.29272500	-0.62651100
C	0.11190100	-0.78223800	-3.19565700
Al	-0.24702300	1.23881600	-1.11894200
H	2.30100200	4.01484800	-2.39635100
H	0.52277300	-1.12916700	-4.15013000
H	1.26218400	1.22282900	-4.46247300
H	0.80601600	3.55291600	-4.08543100
H	-0.82114100	3.20181700	-3.42573100
H	3.66662000	-0.09304200	-2.20374600
H	-3.41743400	-3.34946700	-1.43868000
C	0.39520200	-1.89654300	1.97270100
H	0.64647400	-1.35342600	1.06106800
C	3.66864300	-1.07668800	4.03779600
H	2.82282700	-1.70714200	4.33824700
H	-0.45436900	1.34362500	-4.90652700
O	-0.81848500	-1.42986700	2.55653600
O	3.50400200	-0.56004600	2.72697700
H	3.70963600	-0.20774200	4.69429700
H	4.59862800	-1.65099900	4.13019100
H	0.36019400	-2.97037300	1.77701100
H	1.15260600	-1.68407300	2.72319600
C	-1.81445000	4.87021600	-1.16497100
C	-2.12060500	6.26900000	-0.65760300
C	-4.32591900	6.47837600	-1.96632800
H	-4.34263700	5.44027500	-2.31360600
H	-5.36511400	6.81365400	-1.89694900
H	-3.83260200	7.08203000	-2.73741600
O	-2.54310200	3.94456300	-0.33217800
C	-3.21264600	1.80343400	0.38787300
C	-2.35472100	2.64648500	-0.53509900
O	-1.92210200	2.19851100	-1.64507700
O	-0.64534100	2.25247400	0.39815400
C	-0.71950000	1.76392400	1.73074500
C	-1.96208100	2.35599100	2.40895500
O	-1.96958800	2.84030200	3.51639500

O	-3.14280400	2.30752400	1.73028200
H	-2.85023200	0.77463000	0.33522000
H	-0.86391500	0.67635900	1.72369400
C	-4.67692200	1.88828200	-0.04642500
H	-4.71674700	1.66565600	-1.11905200
C	0.56758800	2.08451500	2.49510300
H	0.65455000	3.17220200	2.59132300
H	1.39119100	1.74724500	1.86272700
H	-5.00390600	2.92517100	0.08560000
H	-0.75067700	4.63539000	-1.08830900
H	-2.13182800	4.73214400	-2.20231500
H	-1.59260700	6.97577100	-1.31185800
H	-1.69213400	6.38021900	0.34449300
C	-3.61745500	6.60871800	-0.61542900
H	-3.72649500	7.63337500	-0.24144700
H	-4.10416500	5.95711600	0.11760700
C	0.68464900	1.41158100	3.86563300
H	0.49736100	0.33698800	3.79018700
H	1.69385000	1.55222400	4.26621000
H	-0.03461200	1.83078900	4.57008500
C	-5.57660200	0.92761600	0.72714000
H	-5.47184200	1.08376400	1.80380300
H	-6.62624600	1.07869100	0.45731800
H	-5.31843500	-0.11034600	0.50939100

INT4

C	-6.70473100	-3.56966300	1.50100500
C	-5.59290200	-4.09513000	0.89564400
C	-4.53254500	-3.25228900	0.45596000
C	-4.61451600	-1.82986400	0.66294600
C	-5.80164400	-1.32289000	1.26791000
C	-6.80701500	-2.16420100	1.67584800
H	-3.42300100	-4.81534500	-0.52047900
H	-7.50899600	-4.21682300	1.83567700
H	-5.50150800	-5.16596900	0.73458100
C	-3.43604600	-3.76084700	-0.25379500
C	-3.54810300	-0.98823900	0.25014900
H	-5.90256600	-0.25266200	1.39772800
H	-7.69868100	-1.74909600	2.13659100
C	-2.38462100	-1.53315400	-0.32168200
C	-2.38748300	-2.94409400	-0.66395000
C	6.02856100	4.01637900	0.03711100
C	5.71868000	3.52257200	1.28365200
C	4.41819100	3.04215600	1.57686800
C	3.40010300	3.08016700	0.57137100
C	3.76009100	3.58690500	-0.70756000
C	5.03524200	4.03993300	-0.96629200
H	4.87677100	2.42952300	3.60477700
H	7.02894600	4.37878000	-0.17805400
H	6.47196700	3.48166200	2.06589500
C	4.10368800	2.48326400	2.84379500
C	2.06665600	2.60683600	0.85600100
H	5.27856700	4.41790200	-1.95492600
C	1.81805300	2.08434800	2.11796900
C	2.85061400	2.00630100	3.09689800
H	2.58759300	1.57015100	4.05413600
C	-3.64092700	0.49198700	0.36467500
C	-3.69575600	1.30306200	-0.81020300
C	-3.67858700	1.10876700	1.60936800
C	-3.70623500	0.74389900	-2.11887700
C	-3.76436800	2.72875900	-0.69094200
C	-3.76882300	2.51834500	1.72861200
C	-3.77772200	1.54510200	-3.23427500
C	-3.82722900	3.52877700	-1.86196500
C	-3.79449500	3.30450500	0.60138600
H	-3.78900300	2.98128200	2.70717400
C	-3.83712800	2.95478100	-3.11050400
H	-3.80024000	1.09235100	-4.22175100
H	-3.86433000	4.60812400	-1.74784600
H	-3.84048100	4.38550100	0.69469100
H	-3.89515700	3.57543000	-3.99972700
C	1.06725600	2.71943900	-0.24848200
C	0.52708600	3.99115000	-0.58904300
C	0.74357300	1.59017400	-1.01065900
C	0.73915600	5.14295400	0.22187000
C	-0.27866700	4.14502500	-1.76778100
C	0.01529400	1.77388100	-2.24672300
C	0.17533300	6.35359500	-0.10494100
H	1.34883500	5.04423300	1.11235300
C	-0.83873300	5.41352800	-2.08123300
C	-0.47081100	3.02675600	-2.59406600
C	-0.62432300	6.49827600	-1.26776700
H	0.34482400	7.21380000	0.53595700
H	-1.44731400	5.50086000	-2.97689700
H	-1.03093100	3.14144100	-3.51674100
H	-1.05865600	7.46315300	-1.50928600
C	-1.49694100	-3.41772100	-1.69157900
N	-0.41804700	-2.80572600	-2.05349400

N	0.15433300	-0.53160800	-2.97777800
C	0.10627700	-2.93360900	-3.40378700
C	-0.07859200	-1.53085700	-4.02752200
O	-1.37831100	-0.75194700	-0.61991900
O	1.11655200	0.39247800	-0.61586400
C	-0.16891100	0.69932900	-3.18989800
Al	0.36066500	-1.22129300	-1.05401000
H	-1.84220100	-4.27577700	-2.28078500
H	-0.61161800	0.98167700	-4.15148200
H	-1.09979800	-1.43036700	-4.41636900
H	-0.42296300	-3.69393500	-3.98974000
H	1.16755700	-3.17519500	-3.35556100
H	-3.67731100	-0.33376500	-2.22280800
H	3.01397600	3.60858200	-1.49160500
C	-0.60724500	1.77038100	1.94696900
H	-0.69603500	1.15232500	1.05358700
C	-3.80406100	0.81488700	3.98881400
H	-3.02602700	1.53328400	4.27472000
H	0.62086900	-1.38797000	-4.85860100
O	0.64660600	1.59471200	2.60296000
O	-3.58026900	0.28150400	2.69329400
H	-3.76471700	-0.03604100	4.66875400
H	-4.78644800	1.29747000	4.06099800
H	-0.78776800	2.81891000	1.70117800
H	-1.33818000	1.42863800	2.67523100
C	2.48645700	-4.68037200	-1.28133400
C	2.88804700	-6.04381100	-0.74720900
C	5.15611700	-6.09827300	-1.96127100
H	5.10161700	-5.07136500	-2.33728900
H	6.21580900	-6.34228700	-1.84097800
H	4.74820500	-6.75981500	-2.73464400
O	3.08288300	-3.68841300	-0.41210600
C	3.40906900	-1.50925200	0.42301800
C	2.77536500	-2.42262700	-0.60880300
O	2.22437700	-2.01264000	-1.65351500
O	0.79434400	-2.17342300	0.43792100
C	0.89210400	-1.66559500	1.75153900
C	2.15424400	-2.26176800	2.38929800
O	2.18788300	-2.85598100	3.44037800
O	3.34158800	-2.10400400	1.72279500
H	2.88883000	-0.54996500	0.38660000
H	1.03541900	-0.57585500	1.73972100
C	4.89502100	-1.32793300	0.09215600
H	4.96581500	-0.98701000	-0.94756700
C	-0.36848100	-1.97800400	2.56296200
H	-0.47893900	-3.06616700	2.62142500
H	-1.20970300	-1.59993300	1.97695500
H	5.37449100	-2.31126900	0.15002800
H	1.40431800	-4.53670200	-1.25987300
H	2.84760400	-4.51080900	-2.29951300
H	2.44976000	-6.79610200	-1.41666100
H	2.42550000	-6.18237400	0.23595000
C	4.40458300	-6.25668400	-0.63689300
H	4.58049100	-7.26074400	-0.23411000
H	4.80501100	-5.55278100	0.10008500
C	-0.41073300	-1.35776700	3.96250100
H	-0.18947600	-0.28721900	3.92428800
H	-1.40750800	-1.48273900	4.39888200
H	0.31870800	-1.83029900	4.62192800
C	5.58085800	-0.33255400	1.02418300
H	5.46020300	-0.63710500	2.06658300
H	6.65065200	-0.27137100	0.80288300
H	5.15528000	0.66551400	0.90877500

***si*-enantioface: *R,R*-Al-O^{n}Bu + CL**

INT1

C	7.69757800	1.78971100	0.24192900
C	6.71063600	2.58411300	-0.28448000
C	5.38654600	2.08939500	-0.44973000
C	5.07836300	0.73914900	-0.06108300
C	6.12974300	-0.05326800	0.48206200
C	7.39678400	0.45750400	0.62831000
H	4.58952600	3.89323000	-1.31691300
H	8.70510700	2.17358600	0.36581300
H	6.92239000	3.60709000	-0.58417900
C	4.36252500	2.87844800	-0.99727800
C	3.76138200	0.23397100	-0.22257400
H	5.90804300	-1.07102200	0.78109700
H	8.18154700	-0.16560100	1.04685400
C	2.74408800	1.05048700	-0.72383100
C	3.06620900	2.39604800	-1.13468500
C	-3.05801800	-0.54553500	4.49052700
C	-2.23024600	-1.64409000	4.52645900
C	-1.83418700	-2.29923600	3.33252000
C	-2.28615700	-1.79832600	2.06897900
C	-3.15906200	-0.67743400	2.07109700
C	-3.53303700	-0.06790900	3.24776500

H	-0.69477200	-3.86217100	4.30954900
H	-3.35452800	-0.05138600	5.41042600
H	-1.86922900	-2.03305500	5.47495900
C	-1.01263900	-3.45760900	3.35251200
C	-1.87352000	-2.43552900	0.85177200
H	-4.19679800	0.79048800	3.22314800
C	-1.00573300	-3.51617000	0.92482500
C	-0.61136300	-4.04606800	2.18296300
H	0.02991600	-4.92037300	2.17476800
C	3.43053200	-1.17742600	0.11558400
C	3.76657600	-2.23161200	-0.78301400
C	2.75160300	-1.47712900	1.29012800
C	4.42693200	-1.98859700	-2.02032700
C	3.42236700	-3.58449700	-0.45654400
C	2.41487300	-2.81386900	1.61418800
C	4.72647100	-3.02057000	-2.87852400
C	3.74788200	-4.62649900	-1.36454100
C	2.74439100	-3.83685400	0.75831100
H	1.87433400	-3.03215300	2.52376300
C	4.38575600	-4.35593600	-2.55205200
H	5.22770000	-2.81046600	-3.81876400
H	3.47700800	-5.64547700	-1.10088600
H	2.46593700	-4.85737800	1.00306200
H	4.62831900	-5.15982600	-3.24010500
C	-2.47511700	-1.98447200	-0.43518600
C	-3.58167200	-2.68459900	-0.98334200
C	-1.97673800	-0.83608100	-1.06514000
C	-4.11530900	-3.85821900	-0.37579400
C	-4.20989800	-2.21295900	-2.19124600
C	-2.62687200	-0.35848500	-2.26197600
C	-5.18619500	-4.51653100	-0.92896000
H	-3.65756700	-4.22430100	0.53509000
C	-5.31959500	-2.92183200	-2.73403700
C	-3.71418900	-1.04902100	-2.79363000
C	-5.80011600	-4.05015800	-2.12160800
H	-5.57211600	-5.40970700	-0.44668000
H	-5.77698400	-2.54579900	-3.64518500
H	-4.19511700	-0.66950600	-3.69206400
H	-6.64536500	-4.58687500	-2.53993700
C	2.07370800	3.24623900	-1.74354500
N	0.82060000	2.95697900	-1.79639000
N	-1.23218600	1.61528300	-2.54813300
C	-0.14197500	3.78108400	-2.50496800
C	-0.96232500	2.81292200	-3.36412500
O	1.52492200	0.56146100	-0.84188900
O	-0.92996100	-0.22851900	-0.57800900
C	-2.20475100	0.84100900	-2.92383000
Al	-0.08539700	1.34813400	-0.92199100
H	2.42955400	4.18124500	-2.19151700
H	-2.77761200	1.12320300	-3.80887900
H	-0.37499000	2.51683600	-4.24183400
H	0.33239800	4.55679400	-3.11739800
H	-0.80159200	4.25941500	-1.77493700
H	4.68611800	-0.96878500	-2.27994000
H	-3.52579500	-0.30400100	1.12315700
C	-0.01034100	-3.42497700	-1.26593800
H	0.36522200	-2.45168600	-0.94000200
C	1.65963100	-0.67769000	3.26405000
H	0.68846000	-1.11146300	3.00627200
H	-1.90014700	3.27234200	-3.68746900
O	-0.50097200	-4.18572600	-0.15066800
O	2.44637800	-0.42845500	2.10665600
H	1.50762100	0.29462200	3.73202000
H	2.17918400	-1.33843700	3.96925800
H	-0.79109700	-3.28735200	-2.01852300
H	0.80970500	-4.00704000	-1.68876100
C	-0.59245100	1.87120100	1.76561300
C	0.26550200	2.79722900	2.62732300
O	-0.64899600	2.33026800	0.42776100
H	-1.60766300	1.81701600	2.18515200
C	-3.74235900	3.29241500	-1.33891100
C	-3.93453500	2.24622600	-0.26214800
C	-2.71307900	4.77424500	0.32504100
C	-4.92760900	2.64344400	0.84685500
C	-3.82049300	4.90500000	1.36436800
C	-4.33775800	3.57141000	1.91112200
H	-2.95342300	2.00991800	0.16691900
H	-5.81717900	3.10164900	0.39622800
H	-2.20985500	5.73502900	0.19545600
H	-4.64054700	5.49029400	0.92902000
H	-4.29454100	1.35989500	-0.78512200
H	-1.96326800	4.02260300	0.59187000
H	-5.27161700	1.72237600	1.32948700
H	-3.41950300	5.49805500	2.19552700
H	-5.09474500	3.76608700	2.68011600
H	-3.51000300	3.05232200	2.41113500
O	-3.20322100	4.48838900	-1.01267300
O	-4.04477700	3.10620700	-2.50283900

H	-0.19442700	0.84867000	1.81226500
H	-0.21383000	3.78535600	2.68263000
H	0.27773200	2.40360100	3.65460000
C	1.69396300	2.95342300	2.10112500
H	1.64313400	3.41011500	1.10835900
H	2.14207700	1.96411300	1.95870500
C	2.59078200	3.79659300	3.00742000
H	2.69435500	3.34211600	3.99975000
H	3.59573300	3.89835500	2.58451000
H	2.18255400	4.80438900	3.15015100

TS1

C	7.25315500	-0.69080200	0.48753700
C	6.61250600	0.17521900	-0.36216300
C	5.23031000	0.01831000	-0.65913300
C	4.48580900	-1.05588500	-0.06090000
C	5.19249100	-1.94274100	0.80206400
C	6.52848000	-1.76350900	1.06776500
H	5.12706200	1.66160300	-2.04769600
H	8.30735900	-0.56434900	0.71258800
H	7.14996400	0.99883600	-0.82502600
C	4.56376400	0.87556300	-1.54894100
C	3.09665100	-1.20386500	-0.33087100
H	4.65526600	-2.77091400	1.24791100
H	7.04008900	-2.45492800	1.73103700
C	2.42449000	-0.29237700	-1.15978400
C	3.20613300	0.75010800	-1.79973000
C	-3.00610300	2.36009500	4.01724700
C	-2.41358700	1.18210900	4.41291600
C	-2.35956500	0.06322500	3.54374300
C	-2.90359700	0.17008600	2.22387900
C	-3.52707400	1.39220900	1.85583600
C	-3.58129400	2.45589900	2.72940400
H	-1.41120500	-1.27575400	4.95843800
H	-3.04516800	3.20858700	4.69338100
H	-1.98296300	1.08781400	5.40647100
C	-1.79952500	-1.17703800	3.94805400
C	-2.83385700	-0.93815400	1.31978900
H	-4.06850800	3.37799200	2.42559900
C	-2.24820900	-2.12062900	1.75294600
C	-1.75586800	-2.23701700	3.08131300
H	-1.33321100	-3.19233200	3.36795200
C	2.34287200	-2.33309600	0.28217400
C	1.97077200	-3.46804900	-0.49637800
C	2.04649500	-2.32397400	1.64039200
C	2.23020600	-3.54101600	-1.89300200
C	1.32142300	-4.58250100	0.12797400
C	1.39153200	-3.41799600	2.25597800
C	1.86231100	-4.64459700	-2.62571000
C	0.96286100	-5.71153500	-0.65570600
C	1.04044200	-4.51858600	1.51211300
H	1.16181500	-3.38901200	3.31258500
C	1.22120800	-5.74499900	-2.00529300
H	2.06657600	-4.67605400	-3.69191700
H	0.47152100	-6.54738700	-0.16466200
H	0.53067900	-5.35241200	1.98544000
H	0.93891600	-6.61062500	-2.59658700
C	-3.38985300	-0.79573200	-0.05453100
C	-4.68644000	-1.26464800	-0.37609100
C	-2.57000600	-0.21158900	-1.03205700
C	-5.54411100	-1.85454500	0.59696200
C	-5.17276500	-1.16031300	-1.72714600
C	-3.04833600	-0.15119500	-2.39268600
C	-6.79843500	-2.29832100	0.25608300
H	-5.18491700	-1.94300500	1.61578300
C	-6.47862200	-1.63071900	-2.04189600
C	-4.32537100	-0.61033000	-2.70197800
C	-7.27818200	-2.18707300	-1.07586900
H	-7.43516800	-2.74245800	1.01554300
H	-6.82786400	-1.54124400	-3.06728600
H	-4.67373400	-0.55570200	-3.73141700
H	-8.27269500	-2.54428500	-1.32321500
C	2.60424900	1.57856600	-2.81672100
N	1.33332600	1.73666000	-2.92146200
N	-0.98984700	0.69892000	-3.36366300
C	0.70628300	2.27089300	-4.11476600
C	-0.27418200	1.16585800	-4.55258700
O	1.13357100	-0.41488300	-1.38359700
O	-1.38383000	0.21076300	-0.69716000
C	-2.21884900	0.32332800	-3.47146700
Al	-0.01607900	1.03452200	-1.56891000
H	3.27110000	2.03116100	-3.56016000
H	-2.69325000	0.34310900	-4.45977900
H	0.30552000	0.32760200	-4.95919200
H	1.42998700	2.49028000	-4.90895000
H	0.15586500	3.17427900	-3.84501900
H	2.72572400	-2.70470900	-2.37131100
H	-3.96552900	1.47096000	0.86736500

C	-1.70299100	-3.18931700	-0.35176900
H	-0.95128900	-2.40672300	-0.48358200
C	2.14767200	-1.14288100	3.72479000
H	1.06612800	-1.14676600	3.90247700
H	-0.96266700	1.52174900	-5.32802900
O	-2.13215400	-3.26331800	1.01669700
O	2.44385900	-1.21876300	2.34023000
H	2.56729200	-0.19546800	4.06326600
H	2.61599900	-1.96217400	4.28477500
H	-2.54592500	-3.01683400	-1.02430800
H	-1.25640800	-4.15792200	-0.57150600
C	0.62964200	1.42867300	1.20868400
C	1.62043900	2.12662500	2.13539000
O	0.66941200	1.97826800	-0.10277400
H	-0.38644800	1.48469700	1.61826200
C	-0.39598400	3.46989900	-0.75787400
C	-1.23372800	3.79522000	0.45639600
C	1.17314100	5.20986500	-0.17944000
C	-1.86865000	5.19511300	0.37467300
C	0.34853800	6.47962000	0.01409200
C	-0.92397100	6.30581300	0.85313600
H	-0.64630700	3.72296200	1.37348700
H	-2.21356400	5.39312500	-0.64751300
H	2.14826100	5.45105000	-0.60834000
H	0.09567200	6.86366600	-0.98189100
H	-1.99165200	3.01690600	0.50789300
H	1.34517900	4.67220500	0.75748300
H	-2.76095700	5.19835600	1.01014100
H	0.99083900	7.23253600	0.48784400
H	-1.46277700	7.26028700	0.87755500
H	-0.63702200	6.08740500	1.89101000
O	0.56924200	4.33011600	-1.14307800
O	-0.90470800	2.80454200	-1.72082200
H	0.88158300	0.36580400	1.14296400
H	1.33401200	3.18146600	2.26252600
H	1.52840500	1.67585100	3.13265000
C	3.06661800	2.04064100	1.64551000
H	3.11814800	2.46196500	0.63665700
H	3.34072600	0.98634100	1.54973800
C	4.06163900	2.75215800	2.56176900
H	4.04247900	2.33239700	3.57495500
H	5.08535200	2.65583500	2.18532000
H	3.83538000	3.82218500	2.64931300

INT2

C	7.02776700	-1.66550300	0.35792900
C	6.51019200	-0.68669900	-0.45278600
C	5.11795100	-0.63644200	-0.73951100
C	4.23519600	-1.61847600	-0.17088100
C	4.81458300	-2.62859100	0.64968900
C	6.16413100	-2.64789500	0.90698300
H	5.23825800	1.05165900	-2.07348400
H	8.09064700	-1.69618400	0.57539700
H	7.15577600	0.06966800	-0.89153200
C	4.57271200	0.33860700	-1.59140800
C	2.83742400	-1.55454800	-0.42409100
H	4.16755800	-3.38859500	1.07089700
H	6.57828100	-3.42808300	1.53900500
C	2.29665200	-0.51756600	-1.19781400
C	3.20875200	0.41727400	-1.82654000
C	-2.22359000	2.27290400	4.26739100
C	-1.94637000	0.95254100	4.54093800
C	-2.19700700	-0.06055700	3.58077900
C	-2.71751300	0.29944900	2.29630200
C	-3.01556600	1.66721400	2.05650700
C	-2.77906800	2.62653800	3.01634200
H	-1.61343500	-1.71817200	4.84823500
H	-2.03009000	3.03976400	5.01099700
H	-1.53759600	0.66284500	5.50562100
C	-1.96858600	-1.43345600	3.86117100
C	-2.94110900	-0.70422700	1.29959600
H	-3.01418700	3.66605100	2.80806400
C	-2.64615400	-2.02662800	1.60540500
C	-2.19368300	-2.38611200	2.90314700
H	-2.01930300	-3.43855800	3.09193600
C	1.92542900	-2.59176600	0.13392300
C	1.38833500	-3.61076900	-0.70730300
C	1.61561800	-2.60711200	1.48801000
C	1.64948800	-3.64969000	-2.10500900
C	0.56338900	-4.63954700	-0.14699000
C	0.79276300	-3.61700800	2.04199900
C	1.12053400	-4.64052000	-2.89793700
C	0.04044000	-5.65434800	-0.99190700
C	0.28147400	-4.60695900	1.23799500
H	0.55214000	-3.60410800	3.09606700
C	0.30642900	-5.65685800	-2.34043600
H	1.33035400	-4.64732300	-3.96347200
H	-0.58184900	-6.42706900	-0.54824800

H	-0.35902400	-5.37387300	1.66311000
H	-0.10163200	-6.43447600	-2.97870600
C	-3.50707400	-0.31866200	-0.02327000
C	-4.87743300	-0.52900300	-0.31638700
C	-2.63803300	0.21993600	-0.98434400
C	-5.78692900	-1.06599000	0.64007000
C	-5.39057500	-0.20420400	-1.62155600
C	-3.15241700	0.50201400	-2.30526100
C	-7.11054300	-1.25723300	0.32677000
H	-5.41241600	-1.31804000	1.62521200
C	-6.76847000	-0.41525200	-1.90895600
C	-4.50052700	0.29640200	-2.58432000
C	-7.61447800	-0.92964400	-0.95960800
H	-7.78453600	-1.66575900	1.07394600
H	-7.13513400	-0.16123700	-2.90009300
H	-4.87462500	0.51557300	-3.58229600
H	-8.66397200	-1.08880400	-1.18542000
C	2.71506100	1.36222400	-2.80137200
N	1.47004700	1.65685000	-2.90100100
N	-1.02249000	1.09083700	-3.31749200
C	0.90598800	2.40509200	-4.00693400
C	-0.27711700	1.54593200	-4.49391100
O	0.99518700	-0.43515000	-1.37406900
O	-1.38316300	0.39782200	-0.67896000
C	-2.30186200	0.93757600	-3.38462000
Al	0.02597000	1.12697400	-1.57002200
H	3.43918100	1.79295100	-3.50257300
H	-2.80790900	1.13286900	-4.33716400
H	0.11928700	0.66447900	-5.01352500
H	1.62758200	2.57544500	-4.81483900
H	0.54045200	3.35771500	-3.61646000
H	2.27712700	-2.87892200	-2.53607400
H	-3.43236100	1.94485600	1.09515100
C	-2.34886500	-2.95721700	-0.60407100
H	-1.47256800	-2.30671700	-0.66778200
C	1.90689900	-1.59233300	3.64179200
H	0.84114500	-1.45037400	3.85475200
H	-0.91752700	2.10067400	-5.18923100
O	-2.79667300	-3.08301500	0.75449800
O	2.15910100	-1.60546900	2.24615500
H	2.47155700	-0.74670000	4.03497300
H	2.25841900	-2.51259000	4.12475600
H	-3.14393300	-2.57628000	-1.24910600
H	-2.07299500	-3.96299600	-0.92029800

C	0.78150900	1.37640500	1.36225000
C	1.91266000	1.82131700	2.27761200
O	0.90763200	1.93860400	0.03449800
H	-0.20058500	1.61448100	1.77400100
C	0.34083600	3.30112500	-0.39858500
C	-0.43076900	3.97526200	0.73328100
C	2.22404500	4.80425600	0.02231400
C	-0.73950800	5.44909900	0.43037800
C	1.70400500	6.23879900	0.09586800
C	0.38566000	6.40128400	0.86219600
H	0.09835700	3.90692600	1.68763900
H	-0.95827500	5.56204100	-0.63729000
H	3.23950800	4.79403300	-0.38622300
H	1.58218200	6.59064100	-0.93588500
H	-1.34920500	3.40081400	0.83998900
H	2.27234100	4.33887500	1.01333800
H	-1.65223200	5.72963900	0.96858600
H	2.47323100	6.87050500	0.55909100
H	0.04477400	7.43879600	0.76144600
H	0.57443100	6.24464800	1.93383600
O	1.44195800	4.02412300	-0.88022300
O	-0.41994000	2.94223600	-1.43616600
H	0.82456400	0.29818600	1.20656300
H	1.83235000	2.89644400	2.48678600
H	1.76018200	1.32268400	3.24376300
C	3.30242900	1.49764900	1.72502700
H	3.40794100	1.96416800	0.74057100
H	3.37155100	0.41950500	1.56096800
C	4.43620800	1.95550300	2.64151100
H	4.36389100	1.48777300	3.63085200
H	5.41148100	1.68907800	2.22184900
H	4.41964800	3.04187100	2.79197400

INT3

C	7.46960900	-1.69941100	-1.48138000
C	6.61308400	-2.44424000	-0.71156000
C	5.33994000	-1.93400600	-0.33215500
C	4.93585000	-0.62064600	-0.76605900
C	5.86726000	0.13063500	-1.54082900
C	7.08715100	-0.39588900	-1.89075800
H	4.84801500	-3.56757800	0.98177800
H	8.43766700	-2.09564800	-1.77038600
H	6.89254700	-3.43830400	-0.37268100
C	4.49193100	-2.64205500	0.53436900

C	3.65043800	-0.12335100	-0.41756200
H	5.59660900	1.12849500	-1.86017900
H	7.77271600	0.19710300	-2.48876600
C	2.74113500	-0.92959100	0.29163600
C	3.23367300	-2.16241200	0.87712700
C	-6.90599600	0.70512100	-0.29931400
C	-6.34478700	0.18068400	-1.44115100
C	-4.97251600	0.37660900	-1.73739900
C	-4.15503600	1.14370000	-0.84601000
C	-4.76701300	1.66285700	0.32798200
C	-6.10174600	1.44936400	0.59296800
H	-4.98821800	-0.78683000	-3.56423000
H	-7.95665400	0.54395700	-0.07944700
H	-6.94422100	-0.40415600	-2.13344900
C	-4.37402100	-0.19936400	-2.88765700
C	-2.75602200	1.33819900	-1.12259100
H	-6.53977600	1.85498600	1.50001500
C	-2.22757400	0.75120500	-2.26485600
C	-3.04243700	-0.01820300	-3.13964400
H	-2.56352400	-0.44448200	-4.01441100
C	3.25966800	1.28374800	-0.70893600
C	3.06935300	2.18919000	0.39599300
C	3.12713700	1.78395500	-1.99525800
C	3.22072500	1.77936200	1.74854000
C	2.74146300	3.56096100	0.15330100
C	2.87556600	3.16247100	-2.22994900
C	3.03508900	2.66095800	2.79039400
C	2.53691000	4.44100300	1.24618000
C	2.66701600	4.02300500	-1.18762200
H	2.81160700	3.48927400	-3.26207800
C	2.67989100	4.00617300	2.54349400
H	3.17389600	2.32027600	3.81286200
H	2.26957100	5.47247200	1.03453100
H	2.43719800	5.06808900	-1.37311000
H	2.53310100	4.69201600	3.37252100
C	-1.93341700	2.02895500	-0.08474500
C	-1.95517400	3.43658600	0.08267700
C	-1.19069500	1.22391600	0.79147800
C	-2.64194400	4.29666700	-0.82213500
C	-1.24805800	4.04446100	1.17845700
C	-0.58189000	1.83031100	1.95543800
C	-2.60680500	5.66198600	-0.66644200
H	-3.18946600	3.84982400	-1.64450400
C	-1.23445200	5.46053600	1.30806300
C	-0.61278700	3.21049600	2.11270000
C	-1.89322400	6.25815300	0.40574700
H	-3.13278700	6.29610400	-1.37407300
H	-0.68672300	5.89690200	2.13914400
H	-0.13044500	3.65723500	2.97675800
H	-1.87553800	7.33833100	0.50894300
C	2.57297000	-2.68852000	2.04897100
N	1.38286900	-2.34337800	2.39590300
N	0.08737200	-0.25297500	3.00519400
C	0.89982400	-2.42857400	3.76216900
C	0.63676100	-0.95893900	4.17113800
O	1.52093100	-0.50694700	0.48502400
O	-1.09707700	-0.05781600	0.56785000
C	-0.02317200	1.03500400	3.02066600
Al	0.03624000	-1.29752600	1.27491800
H	3.17920100	-3.30265600	2.72572000
H	0.30202400	1.58914700	3.90730400
H	1.57689300	-0.48589100	4.48247900
H	1.61911500	-2.90095300	4.44140900
H	-0.03975600	-2.98418600	3.75752100
H	3.50662600	0.75625800	1.95583700
H	-4.16253900	2.22830600	1.02589300
C	-0.11493400	1.93259800	-2.36928400
H	-0.69758200	2.85720800	-2.40220500
C	2.61412200	-0.25514600	-3.17974400
H	3.31778400	-1.03698900	-2.88438000
H	-0.06441400	-0.91755600	5.01173900
O	-0.92727900	0.79806800	-2.67161800
O	3.22356100	1.03986300	-3.14081400
H	2.31056000	-0.41145400	-4.21747300
H	1.73315900	-0.29088700	-2.53418100
H	0.64781400	1.95696300	-3.14689800
H	0.36456700	1.83528700	-1.39518200
C	-3.24835000	-2.26440400	-0.13360000
C	-4.30361400	-2.22224200	0.96283900
O	-2.41805800	-3.44847900	-0.03153000
H	-2.62985400	-1.37060200	-0.10385600
C	-1.25897800	-3.36040200	0.70189000
C	-0.72392700	-4.76920000	0.93539600
C	-0.11236800	-2.56978100	-1.55612600
C	-0.62406200	-5.65767600	-0.30751300
C	-0.37946200	-3.92431100	-2.20718800
C	0.24368200	-5.11533400	-1.45722700
H	-1.38761500	-5.23510800	1.67225000

H	-0.22152900	-6.62878000	0.00636800
H	0.90290000	-2.23335600	-1.78359700
H	0.02162700	-3.87113700	-3.22686900
H	0.25736200	-4.65378800	1.40471700
H	-1.63335200	-5.84762700	-0.68919200
H	-1.45709500	-4.08523600	-2.29672200
H	1.23857200	-4.84057200	-1.08109800
H	0.39819800	-5.93154400	-2.17248500
O	-0.14646000	-2.63022900	-0.12475100
O	-1.25916000	-2.55841000	1.77468900
H	-4.87352800	-1.29315500	0.83824900
H	-3.70277800	-2.33546800	-1.12383500
H	-3.78388000	-2.15739300	1.92286400
C	-5.25769700	-3.42320800	0.96065000
H	-5.88202400	-3.38096400	1.86196200
H	-4.66660800	-4.34386400	1.03502600
C	-6.16343000	-3.48893600	-0.27281000
H	-6.74931200	-2.56896000	-0.37468200
H	-5.58294900	-3.61153000	-1.19311300
H	-6.86069900	-4.33122300	-0.21161900
H	-0.80890000	-1.80462900	-1.91015100

TS2

C	7.53167600	-1.71417700	-1.40312900
C	6.65911500	-2.46946600	-0.66217900
C	5.37894400	-1.96333400	-0.30053000
C	4.98721700	-0.64168500	-0.72063500
C	5.93325500	0.11913400	-1.46841400
C	7.15878600	-0.40416200	-1.80214200
H	4.84811100	-3.62554600	0.96014100
H	8.50481700	-2.10751100	-1.67875000
H	6.93031400	-3.46949300	-0.33420200
C	4.50781100	-2.68628800	0.52929900
C	3.69863500	-0.14611500	-0.38934100
H	5.66777400	1.12159800	-1.77880100
H	7.85657200	0.19554700	-2.37897700
C	2.77180100	-0.95695000	0.29233300
C	3.24247400	-2.21002800	0.85315000
C	-6.91114500	0.93478000	-0.24160400
C	-6.39380200	0.38703500	-1.39350300
C	-5.01884700	0.51731300	-1.71175300
C	-4.14991900	1.24240700	-0.83410200
C	-4.71793700	1.78672900	0.35092600
C	-6.05707500	1.63694300	0.63802100
H	-5.12001800	-0.64235200	-3.53898700
H	-7.96486200	0.82467900	-0.00540100
H	-7.03191300	-0.16543200	-2.07793500
C	-4.46747000	-0.08589900	-2.87207900
C	-2.74653400	1.37129300	-1.13316700
H	-6.45947800	2.06142800	1.55294400
C	-2.26597000	0.75937100	-2.28421300
C	-3.13342600	0.02967600	-3.14416500
H	-2.68911300	-0.42117400	-4.02475300
C	3.30883100	1.26102700	-0.68137700
C	3.11711500	2.16448900	0.42410800
C	3.16151600	1.75791600	-1.96753600
C	3.28219800	1.75417700	1.77484800
C	2.77303400	3.53254000	0.18499000
C	2.87779000	3.13131400	-2.19923000
C	3.10224800	2.63446400	2.81885300
C	2.57816700	4.41207100	1.27978000
C	2.66972000	3.99005600	-1.15536500
H	2.79424100	3.45578200	-3.23066200
C	2.74002200	3.97855900	2.57543700
H	3.25101700	2.29401700	3.83996900
H	2.30024400	5.44128800	1.07105600
H	2.41884100	5.03054400	-1.33940000
H	2.59983100	4.66395800	3.40602500
C	-1.88371000	2.02393400	-0.10300400
C	-1.85835500	3.43009500	0.07903500
C	-1.15699300	1.18782900	0.75672900
C	-2.51380500	4.32188300	-0.81805200
C	-1.13377700	4.00206100	1.18239600
C	-0.53185100	1.75976000	1.92828700
C	-2.43419600	5.68367300	-0.64738300
H	-3.07184300	3.90213100	-1.64761200
C	-1.07544400	5.41507600	1.32864100
C	-0.52576500	3.13695300	2.10716500
C	-1.70543500	6.24398400	0.43366000
H	-2.93584100	6.34248900	-1.35012200
H	-0.51559600	5.82379900	2.16559200
H	-0.03316200	3.55719200	2.97845100
H	-1.65281000	7.32180200	0.54925700
C	2.54821600	-2.76973300	1.98873400
N	1.34060700	-2.45505400	2.30760800
N	0.07660500	-0.35950900	2.93896700
C	0.84588500	-2.56090600	3.66866500
C	0.59165000	-1.09650100	4.10056000

O	1.55619700	-0.52355000	0.48101800
O	-1.08962900	-0.09154900	0.50860900
C	0.00030800	0.92954500	2.98076400
Al	0.04331700	-1.36774200	1.16138400
H	3.14274800	-3.38602900	2.67409900
H	0.33372500	1.45806100	3.88036400
H	1.53067200	-0.64316700	4.44325100
H	1.55824900	-3.04888100	4.34434500
H	-0.09665000	-3.11017100	3.65451000
H	3.57447500	0.73176800	1.97769700
H	-4.07666900	2.32094700	1.04024400
C	-0.05530200	1.76558200	-2.35694200
H	-0.53731700	2.74609900	-2.32894800
C	2.73245300	-0.30516600	-3.15639500
H	3.49310100	-1.04877500	-2.90778900
H	-0.12721400	-1.06315700	4.92661200
O	-0.97904700	0.74198000	-2.72633700
O	3.26836800	1.02213800	-3.11678700
H	2.39360100	-0.45996700	-4.18347400
H	1.88587300	-0.40734100	-2.47455700
H	0.70467400	1.75891300	-3.13739100
H	0.41166300	1.55577100	-1.39431400
C	-3.36511200	-2.16337000	-0.10082100
C	-4.40215600	-2.06285100	1.00672800
O	-2.59018600	-3.39507000	-0.00131700
H	-2.68726100	-1.31400200	-0.08542300
C	-1.49131700	-3.40134800	0.76052700
C	-0.91823200	-4.79769700	0.88639900
C	0.07198400	-2.58294400	-1.59939400
C	-0.89660800	-5.63642100	-0.39524700
C	-0.44311700	-3.86415300	-2.25131900
C	0.01889100	-5.14615100	-1.53152700
H	-1.53718300	-5.30449500	1.63967600
H	-0.58223000	-6.64959300	-0.11529600
H	1.15389800	-2.50016800	-1.78132500
H	-0.08602100	-3.87014900	-3.28869200
H	0.08407700	-4.69252900	1.30679800
H	-1.92029000	-5.72524000	-0.77527200
H	-1.53664800	-3.84569100	-2.29437500
H	1.03889500	-5.00373700	-1.14921900
H	0.07814200	-5.95716600	-2.26728100
O	-0.12316200	-2.61616700	-0.20139300
O	-1.36424600	-2.56400100	1.74130300
H	-4.92728200	-1.10991300	0.87090200
H	-3.82804400	-2.22703300	-1.08586500
H	-3.87344100	-2.00337000	1.96243800
C	-5.41441400	-3.21543900	1.03352300
H	-6.03595500	-3.11485200	1.93185400
H	-4.87406900	-4.16446700	1.13663800
C	-6.32062300	-3.26927500	-0.19993500
H	-6.85382800	-2.32174600	-0.33156400
H	-5.74755200	-3.45375300	-1.11442800
H	-7.06337000	-4.06908800	-0.11309400
H	-0.40828900	-1.69911900	-2.03817500

INT4

C	7.59994000	-1.86682400	-1.28638900
C	6.69268900	-2.61856900	-0.58462900
C	5.41299300	-2.09387500	-0.24789500
C	5.06112500	-0.75568000	-0.65028500
C	6.04025000	-0.00085800	-1.36083900
C	7.26367500	-0.54241100	-1.67155100
H	4.80221000	-3.78254300	0.93789000
H	8.57237200	-2.27442700	-1.54320200
H	6.93470800	-3.63054200	-0.27068000
C	4.49949500	-2.81962900	0.53190900
C	3.77689900	-0.23892300	-0.34160100
H	5.80073400	1.01204600	-1.65933000
H	7.98835400	0.05286800	-2.21911800
C	2.81661400	-1.03740400	0.30706100
C	3.23479500	-2.32440100	0.83157500
C	-6.85797000	1.29740700	-0.27689100
C	-6.37527800	0.70951300	-1.42438300
C	-4.99358400	0.74189300	-1.73801700
C	-4.07819800	1.40705400	-0.85995200
C	-4.61141100	1.99134700	0.32274700
C	-5.95940600	1.93929600	0.60427800
H	-5.16785500	-0.41896300	-3.55980500
H	-7.91814300	1.26491600	-0.04613800
H	-7.04827100	0.20170700	-2.11011400
C	-4.48117800	0.09696300	-2.89452100
C	-2.66849800	1.44201000	-1.15820600
H	-6.33388600	2.39545600	1.51584100
C	-2.22862000	0.80426200	-2.31216400
C	-3.14264400	0.12459300	-3.16642200
H	-2.72823900	-0.35866700	-4.04431300
C	3.41371500	1.17422800	-0.63917000
C	3.23115900	2.08353700	0.46193000

C	3.26866200	1.66526000	-1.92826500
C	3.39028400	1.67419700	1.81364900
C	2.90158900	3.45417000	0.21858200
C	2.98736300	3.03896100	-2.16400700
C	3.22324300	2.55987400	2.85509000
C	2.72340400	4.34044500	1.31061800
C	2.79212200	3.90481100	-1.12341600
H	2.89997400	3.35899600	-3.19650500
C	2.88204800	3.90895500	2.60738700
H	3.36551500	2.22017600	3.87735600
H	2.45815000	5.37232000	1.09897300
H	2.54656800	4.94584700	-1.31166100
H	2.75355200	4.59914500	3.43596800
C	-1.76698600	2.02640000	-0.12116500
C	-1.66907100	3.42594900	0.09124900
C	-1.07989300	1.13625000	0.71572600
C	-2.26860000	4.36910300	-0.79187600
C	-0.92578800	3.93512700	1.21188000
C	-0.44012600	1.64908600	1.90575100
C	-2.12024000	5.72113700	-0.59096500
H	-2.83822200	3.99656800	-1.63597700
C	-0.79756400	5.33941000	1.39103600
C	-0.37026900	3.01857300	2.12064900
C	-1.37540900	6.21912800	0.50915300
H	-2.57893900	6.41999900	-1.28429600
H	-0.22485800	5.70002300	2.24106900
H	0.13348700	3.39229300	3.00651200
H	-1.26873700	7.29002600	0.64946800
C	2.48405000	-2.92248600	1.90966500
N	1.26174100	-2.62221800	2.19022200
N	0.05056700	-0.52236900	2.86007900
C	0.73392500	-2.76904900	3.53496200
C	0.49326600	-1.31642800	4.01254600
O	1.61527100	-0.56676500	0.49520100
O	-1.06327000	-0.13983100	0.42981400
C	0.03248900	0.76546200	2.94365800
Al	0.06882000	-1.45562900	1.01386300
H	3.04563400	-3.56670400	2.59722400
H	0.36747400	1.25139500	3.86696800
H	1.42506600	-0.90145400	4.41791000
H	1.42488800	-3.29092800	4.20750100
H	-0.21480600	-3.30463600	3.48800300
H	3.66661800	0.64745500	2.01790800
H	-3.93768900	2.48129800	1.01375000
C	0.05702200	1.64014300	-2.35578900
H	-0.33345300	2.65841800	-2.28763900
C	2.87130600	-0.40992000	-3.11811200
H	3.65203500	-1.13646000	-2.88254300
H	-0.26338300	-1.29683600	4.80471500
O	-0.95289600	0.72052300	-2.77076300
O	3.37697300	0.92980200	-3.07681500
H	2.52575400	-0.56606900	-4.14274000
H	2.03512900	-0.53856500	-2.42909800
H	0.81703400	1.59650100	-3.13495200
H	0.49813500	1.34771800	-1.40240900
C	-3.62780700	-1.91610600	0.06237000
C	-4.62891500	-1.76844800	1.19444100
O	-2.97787300	-3.23251600	0.09010700
H	-2.85413700	-1.15357100	0.10036700
C	-1.91859700	-3.40774600	0.84690100
C	-1.35587200	-4.80371000	0.76336600
C	0.11712500	-2.57778600	-1.70033000
C	-1.45767100	-5.50650800	-0.59993500
C	-0.71739600	-3.68264900	-2.35209900
C	-0.48266700	-5.06805600	-1.71041000
H	-1.92517900	-5.38735300	1.50346800
H	-1.28983700	-6.57478400	-0.41432500
H	1.18036800	-2.74297100	-1.95725200
H	-0.47068100	-3.72312500	-3.42100200
H	-0.32420900	-4.75129700	1.11257700
H	-2.48686000	-5.41914600	-0.96418800
H	-1.77930300	-3.41930700	-2.28443300
H	0.54516900	-5.11778200	-1.32778900
H	-0.55303100	-5.83298900	-2.49368600
O	-0.06127100	-2.63521800	-0.31954800
O	-1.54814200	-2.56445000	1.69750800
H	-5.07780100	-0.77402400	1.08576400
H	-4.10559300	-1.89571100	-0.91573300
H	-4.08205200	-1.76707400	2.14299400
C	-5.72923800	-2.83794400	1.22039100
H	-6.35049900	-2.67376000	2.10922700
H	-5.27058000	-3.82677400	1.34506200
C	-6.61983100	-2.83904900	-0.02521500
H	-7.05951900	-1.85003400	-0.19005700
H	-6.05463700	-3.10238500	-0.92501000
H	-7.43469700	-3.56351800	0.07225900
H	-0.17010800	-1.60035300	-2.11554100

INT5

C	5.32548200	-5.57944400	1.27508300
C	4.11493100	-6.00223700	0.79039100
C	3.08553700	-5.06727800	0.48435400
C	3.30713000	-3.66093900	0.68670000
C	4.58289800	-3.26354000	1.18572700
C	5.55474200	-4.19181600	1.46934000
H	1.65760700	-6.53247500	-0.17812700
H	6.10754900	-6.29500500	1.50725700
H	3.91851500	-7.05882900	0.62884700
C	1.84472400	-5.47258200	-0.01946600
C	2.28410200	-2.71740400	0.39360700
H	4.77597000	-2.20905700	1.33826800
H	6.51678500	-3.85959500	1.84808100
C	1.04828900	-3.13575400	-0.12740800
C	0.84362900	-4.55386000	-0.32378400
C	-1.29888000	5.22824800	0.50473000
C	-0.59176100	4.46465500	1.40335800
C	-0.96309100	3.12214300	1.67627800
C	-2.10173400	2.55560100	1.01541400
C	-2.79637400	3.36760300	0.07382700
C	-2.40795300	4.66543100	-0.17056300
H	0.66213100	2.72845700	3.04571900
H	-0.99722100	6.24835100	0.29135000
H	0.28036400	4.87045200	1.90781500
C	-0.21457900	2.31100400	2.56211100
C	-2.48000000	1.20636100	1.29671900
H	-2.94845900	5.26019900	-0.90029200
C	-1.72422900	0.46322900	2.19401900
C	-0.57342400	1.00969500	2.81127100
H	0.01898300	0.40721500	3.48700200
C	2.55823700	-1.27185600	0.62144800
C	2.53683800	-0.71878200	1.93476400
C	2.95926100	-0.47358900	-0.44205100
C	2.22467400	-1.50333700	3.08031200
C	2.88633900	0.65657300	2.13384200
C	3.25950000	0.89810900	-0.25276200
C	2.25802500	-0.96009500	4.34383100
C	2.90815700	1.18649800	3.45040100
C	3.21365800	1.44575600	1.00693000
H	3.50995300	1.52766500	-1.09668700
C	2.60165000	0.40098100	4.53660900
H	2.02097000	-1.57994700	5.20337800
H	3.17987800	2.23043600	3.58238900

H	3.41556700	2.50341300	1.13575600
H	2.62425900	0.81652400	5.53900900
C	-3.65049500	0.57493700	0.63654600
C	-4.96585300	1.06586400	0.87259800
C	-3.44320200	-0.52891700	-0.20794000
C	-5.22935000	2.11889700	1.79838000
C	-6.09254000	0.49399000	0.18621900
C	-4.58944300	-1.10835100	-0.86636600
C	-6.50462000	2.58762500	1.99823600
H	-4.40174700	2.54476500	2.35099400
C	-7.40040600	1.01072600	0.41041800
C	-5.86474600	-0.58735200	-0.67338600
C	-7.60751900	2.03965900	1.29207700
H	-6.67370400	3.38846200	2.71190900
H	-8.23152200	0.56491000	-0.12966500
H	-6.70758100	-1.04277200	-1.18886500
H	-8.60627200	2.42933400	1.46083100
C	-0.38819100	-5.07825200	-0.83282200
N	-1.42290300	-4.38537500	-1.18247200
N	-3.35805500	-2.88481300	-1.92738900
C	-2.59278900	-5.10366200	-1.69976100
C	-3.32840200	-4.14674500	-2.64233600
O	0.09742700	-2.27269800	-0.40163700
O	-2.23145600	-1.02014500	-0.37622900
C	-4.46921700	-2.28253700	-1.68306800
Al	-1.47420400	-2.33430600	-1.35179800
H	-0.43290800	-6.16858300	-0.93183000
H	-5.39946700	-2.69404000	-2.09147900
H	-4.32993900	-4.51920900	-2.88892400
H	-3.25408300	-5.35514700	-0.86073100
H	-2.30440900	-6.03272800	-2.20400800
H	1.96540800	-2.54623100	2.94002400
H	-3.63757200	2.94525900	-0.46166900
C	-1.18990000	-1.79334400	2.72380000
H	-1.72488400	-2.74549800	2.70843600
C	3.75382100	-0.42843100	-2.69788000
H	3.81117300	-1.14496700	-3.51787700
H	-2.74926800	-4.00409000	-3.55945000
O	-2.17357900	-0.79125200	2.48869100
O	3.04796200	-1.08815800	-1.65821400
H	4.76667500	-0.15365100	-2.37860600
H	3.22866100	0.46598200	-3.04675200
H	-0.70602200	-1.67546400	3.70029500
H	-0.43676500	-1.78338200	1.93317400

O	-1.06753000	-2.04950700	-3.02257200
C	2.91448500	5.46497400	-0.41198100
C	4.07349700	5.42507400	-1.39862000
O	2.16630300	4.23384300	-0.43684900
H	2.24189600	6.29658600	-0.63712300
C	1.20095000	4.14196100	-1.39586200
C	0.50894400	2.80838100	-1.36056000
C	0.17575400	-1.71431500	-3.58611200
C	0.68628000	2.03870600	-2.67642700
C	0.29719600	-0.21068700	-3.83794300
C	0.15559000	0.61089700	-2.55345700
H	-0.55450900	2.99533600	-1.19833600
H	0.18291400	2.58109800	-3.48477300
H	0.29274400	-2.25071500	-4.54143800
H	-0.46018500	0.10021000	-4.56885400
H	0.87679800	2.22225900	-0.51840500
H	1.75090800	2.01293500	-2.94780700
H	1.27581600	-0.01936700	-4.30138400
H	0.68624000	0.10357300	-1.74083700
H	-0.89482300	0.63752100	-2.24445300
O	0.97329700	5.03354100	-2.18704200
H	3.66458400	5.29838700	-2.40677900
H	3.27327200	5.55678000	0.61722500
H	4.69213500	4.54360500	-1.18488100
C	4.93171900	6.69195300	-1.33996900
H	5.31715200	6.82692400	-0.32024000
H	4.30049300	7.56577700	-1.54705200
C	6.09925600	6.65851700	-2.32819300
H	6.76384200	5.81165900	-2.12414400
H	5.73872900	6.55534600	-3.35732300
H	6.69724700	7.57340100	-2.27313100
H	1.00850200	-2.02341300	-2.93635200

***re*-enantioface: *R,R*-Al-O^{n}Bu + CL**

INT1

C	-7.46196600	-1.78200000	1.33801800
C	-6.61249700	-2.50434800	0.53956800
C	-5.34052700	-1.98593800	0.16671600
C	-4.93144300	-0.68667500	0.63734200
C	-5.85503400	0.04192400	1.44380000
C	-7.07327300	-0.49247400	1.78609200
H	-4.85500000	-3.58663000	-1.18761100
H	-8.42876500	-2.18517000	1.62180200
H	-6.89626800	-3.48748900	0.17325500
C	-4.49727700	-2.67068800	-0.72216400
C	-3.64957900	-0.18164200	0.29723000
H	-5.57742600	1.02826500	1.79284300
H	-7.75308200	0.08251100	2.40793200
C	-2.74641900	-0.96122700	-0.45231800
C	-3.23975000	-2.18415000	-1.05980000
C	6.96259900	0.60285600	0.07824500
C	6.47822500	0.10178600	1.26611700
C	5.11530200	0.25623800	1.62384100
C	4.22257100	0.95148800	0.74568800
C	4.75772500	1.45308100	-0.47332500
C	6.08630600	1.28289400	-0.79772700
H	5.26403000	-0.81922900	3.50030500
H	8.00784800	0.47672300	-0.18651800
H	7.13523700	-0.42796500	1.95124800
C	4.59577800	-0.28991400	2.82661700
C	2.83124200	1.10990300	1.08029000
H	6.46269800	1.67375400	-1.73838000
C	2.38094000	0.55068700	2.26979400
C	3.27213700	-0.15207400	3.13166800
H	2.85298000	-0.55763700	4.04512400
C	-3.23816400	1.20390500	0.65462300
C	-3.04772300	2.15861000	-0.40789600
C	-3.06428600	1.63570600	1.96172700
C	-3.24770500	1.82014000	-1.77384300
C	-2.67199800	3.50636300	-0.10960500
C	-2.74249000	2.99004000	2.25260600
C	-3.07123600	2.74777200	-2.77669200
C	-2.48178200	4.43587100	-1.16307100
C	-2.53240500	3.89446100	1.24893800
H	-2.63439900	3.26170100	3.29693400
C	-2.67730500	4.07109300	-2.47521100
H	-3.24818300	2.46146700	-3.80993900
H	-2.18083100	5.44828000	-0.90863300
H	-2.25337100	4.91845300	1.47924400
H	-2.54036600	4.79439400	-3.27362300
C	1.97481800	1.83310400	0.09230100
C	1.97897000	3.25135700	0.03167100
C	1.23326600	1.07996000	-0.83122800
C	2.66407700	4.05088300	0.99200200
C	1.25250000	3.93194500	-1.00521000
C	0.58965600	1.77355300	-1.92775300
C	2.61173000	5.42401500	0.93916400

H	3.22291100	3.55294800	1.77628000
C	1.22143800	5.35243700	-1.03037400
C	0.60764700	3.15957100	-1.98533300
C	1.88136500	6.08994500	-0.07826400
H	3.13679200	6.00927300	1.68848800
H	0.65798500	5.84138400	-1.82069600
H	0.09893800	3.66220600	-2.80210100
H	1.84982800	7.17457900	-0.10056300
C	-2.59411400	-2.66906200	-2.25675500
N	-1.39183700	-2.36177600	-2.60693500
N	-0.15423400	-0.21743600	-3.09001100
C	-1.01590000	-2.31710700	-4.00901300
C	-0.76390600	-0.81478100	-4.28470500
O	-1.53757200	-0.52533400	-0.66261200
O	1.15805600	-0.21564400	-0.70253400
C	-0.01768100	1.06270800	-3.02641900
Al	-0.05486300	-1.40383600	-1.38791100
H	-3.23501800	-3.20098100	-2.97045200
H	-0.36378200	1.68461400	-3.85925500
H	-1.72012100	-0.31910700	-4.49647000
H	-1.80212100	-2.70334200	-4.66864200
H	-0.09927100	-2.88762900	-4.15754500
H	-3.56473000	0.81514900	-2.02110200
H	4.09591900	1.96761300	-1.15832400
C	0.12379900	1.48778700	2.31568300
H	-0.30274700	1.17241400	1.36253200
C	-2.71534100	-0.49747100	3.06553000
H	-3.52491400	-1.18988900	2.82485100
H	-0.11302600	-0.68868200	-5.15728400
O	1.12003300	0.57444300	2.77513800
O	-3.17819600	0.85814100	3.08189900
H	-2.34846300	-0.70115600	4.07416700
H	-1.90272100	-0.62891100	2.35045200
H	0.51843400	2.50230600	2.23064500
H	-0.64607500	1.46382600	3.08463000
C	-0.21574300	-2.53403900	1.14580800
C	0.26778400	-3.69014700	2.02292200
O	0.15945600	-2.72182200	-0.19399600
H	0.17847600	-1.58459200	1.53934600
C	2.47904800	-2.80866800	-2.06777600
C	2.85356800	-4.26618300	-2.01684900
C	4.47251700	-2.22927600	-0.83249400
C	2.68435900	-4.81166500	-0.57969500
C	4.07223000	-2.89791500	0.47540200

C	3.81924900	-4.40744200	0.36727000
H	3.87909700	-4.41837200	-2.37281500
H	1.72108800	-4.45326200	-0.20677800
H	4.94079000	-1.26469800	-0.65859100
H	3.19430400	-2.37090800	0.86378000
H	2.16931400	-4.77902800	-2.69377100
H	5.15470900	-2.84962000	-1.42582000
H	2.63822800	-5.90417900	-0.63912500
H	4.88229100	-2.71950600	1.18964800
H	3.59773900	-4.80194500	1.36528000
H	4.75014300	-4.89474800	0.04257900
O	3.31291300	-1.88508800	-1.64045900
O	1.37847600	-2.45315100	-2.50172200
H	-1.31477200	-2.48573800	1.24740800
H	-0.08192000	-4.62405700	1.56463200
H	1.36557100	-3.72274800	2.00762100
C	-0.22043400	-3.60821000	3.47539600
H	-0.04750400	-4.57259200	3.96956400
H	-1.30976500	-3.46036200	3.47865800
C	0.45101600	-2.50084100	4.29449100
H	0.29451200	-1.51125900	3.85879400
H	0.07538900	-2.48347300	5.32350700
H	1.53447300	-2.66383000	4.34002300

TS1

C	-6.44873900	-3.51165900	1.75505700
C	-5.47714500	-3.98979200	0.91383300
C	-4.42425300	-3.14707000	0.45824500
C	-4.36856000	-1.77271000	0.89046800
C	-5.41498600	-1.30877300	1.74129000
C	-6.41329200	-2.15269900	2.16325400
H	-3.60532400	-4.57311600	-0.93069500
H	-7.24650400	-4.16051900	2.10182200
H	-5.49474100	-5.02228100	0.57493900
C	-3.48216100	-3.58928800	-0.48296600
C	-3.29922000	-0.93968800	0.46724500
H	-5.40810300	-0.27507300	2.06158600
H	-7.19180500	-1.77310000	2.81833700
C	-2.25112500	-1.46338000	-0.31434700
C	-2.43147400	-2.77945800	-0.90013400
C	6.24980900	0.46807300	-0.48218800
C	5.95294300	0.46116100	0.86171400
C	4.71842100	0.97208100	1.33879100
C	3.76389600	1.50205300	0.41238000

C	4.11058100	1.50086600	-0.96473400
C	5.31337100	0.99513700	-1.40044600
H	5.13767400	0.59051200	3.43023400
H	7.19984600	0.07948300	-0.83720400
H	6.66512300	0.07152300	1.58492100
C	4.41288400	0.98700600	2.72451200
C	2.51047500	2.02820900	0.88357900
H	5.54524700	1.00094300	-2.46102100
C	2.25719700	1.99167900	2.24867400
C	3.21643400	1.48444300	3.16469300
H	2.95186000	1.49288100	4.21635600
C	-3.29546600	0.52295800	0.74828400
C	-3.44813200	1.43397900	-0.35785100
C	-3.21879200	1.05406000	2.02741000
C	-3.57047000	0.98465800	-1.70083000
C	-3.51129500	2.84459000	-0.12546700
C	-3.35623700	2.44974100	2.25653100
C	-3.71262200	1.87197500	-2.74478200
C	-3.63869900	3.73537300	-1.22131700
C	-3.48129200	3.32233700	1.21116800
H	-3.31449700	2.79299200	3.28424400
C	-3.73582300	3.26540700	-2.51069900
H	-3.81931600	1.49522800	-3.75853200
H	-3.66448300	4.80227200	-1.01766500
H	-3.54860700	4.39112400	1.39138300
H	-3.84418000	3.95615800	-3.34152100
C	1.53325500	2.57406300	-0.10223900
C	1.18295500	3.94754200	-0.15097300
C	0.94292700	1.66437700	-0.99435300
C	1.70063400	4.90296600	0.77056100
C	0.24808100	4.41361800	-1.14414300
C	0.08887600	2.15464800	-2.05062900
C	1.30035600	6.21672700	0.72794100
H	2.41317600	4.57120000	1.51582200
C	-0.13973500	5.78229900	-1.15779400
C	-0.24521500	3.50294400	-2.09291100
C	0.36636800	6.66856600	-0.24072700
H	1.70512000	6.92289400	1.44702900
H	-0.85041300	6.10839100	-1.91281000
H	-0.91531000	3.86084700	-2.86901600
H	0.06206600	7.71031200	-0.25414000
C	-1.77026700	-3.06871100	-2.14907700
N	-0.75163000	-2.40736900	-2.58322500
N	-0.19446400	0.01135100	-3.15486600
C	-0.47726200	-2.26206200	-4.00377600
C	-0.71670700	-0.75954500	-4.28999400
O	-1.21261900	-0.72269900	-0.57641600
O	1.20228900	0.39528300	-0.86665600
C	-0.38461300	1.28919400	-3.10297500
Al	0.35892400	-1.07259900	-1.52096400
H	-2.26726700	-3.78624300	-2.81326000
H	-0.93131300	1.78234700	-3.91307500
H	-1.79394100	-0.57569000	-4.39565500
H	-1.12975800	-2.88502500	-4.62701800
H	0.56903600	-2.51015300	-4.18743100
H	-3.56878100	-0.07942400	-1.89843500
H	3.40695700	1.90653300	-1.67999900
C	-0.15567800	2.15036200	2.28020900
H	-0.11535000	1.26377900	1.64260700
C	-2.14428200	-0.79293200	3.15766400
H	-2.68660900	-1.72560400	2.98572700
H	-0.22446100	-0.46552100	-5.22372100
O	1.11978200	2.45132500	2.85065300
O	-3.03061700	0.32930800	3.17321600
H	-1.67444100	-0.82100700	4.14321200
H	-1.37694300	-0.67614100	2.38945700
H	-0.53793000	2.98799300	1.69444400
H	-0.82592500	1.96472700	3.12077600
C	1.39067500	-1.77174100	1.05732800
C	0.88099000	-2.67957000	2.17058500
O	1.07024800	-2.27482600	-0.23597100
H	2.47457000	-1.62390300	1.11287400
C	2.43099900	-2.55376200	-1.49757500
C	2.30412500	-4.05479100	-1.71863600
C	4.64257700	-2.84695400	-0.47939000
C	2.35273100	-4.99282500	-0.51084300
C	4.36174100	-3.85993100	0.62462700
C	3.72910100	-5.16374200	0.13390000
H	3.08086100	-4.32278600	-2.45047300
H	1.62911200	-4.62969900	0.22196100
H	5.34978000	-2.09306400	-0.13379000
H	3.74472300	-3.38912100	1.39562100
H	1.34549600	-4.17437600	-2.22579100
H	5.07872600	-3.34602700	-1.35530000
H	1.99949000	-5.97702800	-0.84092000
H	5.32562800	-4.08744300	1.09745400
H	3.64605900	-5.86449100	0.97303600
H	4.41133700	-5.63158500	-0.59050500

O	3.52870300	-2.04439300	-0.91205200
O	1.89948400	-1.79135200	-2.39613600
H	0.95403600	-0.77565100	1.17232100
H	-0.20126000	-2.80165000	2.04227500
H	1.31743500	-3.68218300	2.07600200
C	1.19264000	-2.11947600	3.56692000
H	0.61149500	-2.67415800	4.31381500
H	0.85190000	-1.07748300	3.62347300
C	2.67793600	-2.18045000	3.93794000
H	3.28612700	-1.56841800	3.26684400
H	2.84632700	-1.81103500	4.95477400
H	3.04994100	-3.21089000	3.89266500

INT2

C	-7.28121900	-2.33017700	1.18325700
C	-6.39018000	-2.90892000	0.31609400
C	-5.15897900	-2.27000600	-0.00203500
C	-4.83264500	-1.00156800	0.60008700
C	-5.80074800	-0.42074100	1.47055900
C	-6.97842400	-1.06824400	1.75638700
H	-4.59626200	-3.66972600	-1.53905000
H	-8.21676500	-2.82442900	1.42445200
H	-6.60997200	-3.86597900	-0.14958000
C	-4.28698000	-2.79655800	-0.96857600
C	-3.58631900	-0.38001400	0.31188100
H	-5.59217100	0.54265200	1.91633900
H	-7.69219000	-0.60437700	2.43066200
C	-2.64018900	-1.03037100	-0.50218300
C	-3.06884800	-2.19308100	-1.25552100
C	6.73549300	0.26990700	0.02197300
C	6.16381400	-0.30504800	1.13459500
C	4.83687900	0.01023900	1.51968500
C	4.07737800	0.95065900	0.75231900
C	4.69758600	1.51938800	-0.39297400
C	5.98732900	1.18865800	-0.74801600
H	4.79710200	-1.31341500	3.23556600
H	7.75126100	0.01805500	-0.26672000
H	6.71979900	-1.02051400	1.73477200
C	4.22657600	-0.60189300	2.64501500
C	2.72170400	1.27103200	1.11475400
H	6.43259200	1.63804500	-1.63069900
C	2.17759500	0.63428900	2.22128400
C	2.94024300	-0.29183000	2.98564400
H	2.45392000	-0.74090100	3.84453900
C	-3.28366100	1.00491500	0.76929100
C	-3.13793400	2.04326200	-0.22018900
C	-3.19867100	1.36129200	2.10705600
C	-3.25160700	1.78851900	-1.61389200
C	-2.89590100	3.39448100	0.18600300
C	-3.05157600	2.71711600	2.50391000
C	-3.09516800	2.79340900	-2.54255800
C	-2.71529000	4.40409100	-0.79358400
C	-2.88192800	3.70386500	1.57214700
H	-3.03065200	2.92565900	3.56806800
C	-2.80795900	4.11560000	-2.13525800
H	-3.20408600	2.56799200	-3.60006800
H	-2.50986200	5.41696800	-0.45860200
H	-2.72780000	4.73359900	1.88106200
H	-2.67912000	4.89874600	-2.87630100
C	1.94288000	2.13061500	0.17061100
C	2.01400000	3.54280700	0.17106300
C	1.19035500	1.45903500	-0.80653400
C	2.74221100	4.26585900	1.16039100
C	1.31700500	4.30006200	-0.83806000
C	0.57924600	2.21784500	-1.87589900
C	2.75783000	5.63982100	1.16495700
H	3.28153600	3.70379800	1.91505000
C	1.35602800	5.72166800	-0.79847600
C	0.64091200	3.60714200	-1.85556800
C	2.05488700	6.38308100	0.18020100
H	3.31664700	6.16775200	1.93220100
H	0.81793800	6.27381500	-1.56472000
H	0.15526800	4.17271400	-2.64614300
H	2.07754700	7.46783300	0.20369100
C	-2.41261600	-2.48263800	-2.50939900
N	-1.27098800	-1.98440300	-2.83536200
N	-0.15351100	0.28916400	-3.15824300
C	-0.85473800	-1.79818500	-4.21454300
C	-0.75296400	-0.26197200	-4.38117300
O	-1.44818100	-0.51847500	-0.64026200
O	1.09277200	0.16779900	-0.74618700
C	-0.02218500	1.56837200	-3.01415700
Al	0.04135000	-1.01259400	-1.63074400
H	-2.99493900	-3.03607600	-3.25573300
H	-0.36296700	2.23246200	-3.81503600
H	-1.75798100	0.15773400	-4.51906000
H	-1.56424700	-2.22212600	-4.93493400
H	0.12993900	-2.25286300	-4.33965600

H	-3.48318900	0.78462000	-1.94500200
H	4.13420700	2.22002700	-0.99666900
C	0.11063400	1.90519800	2.43802800
H	-0.35921800	1.86716500	1.45442100
C	-2.52745900	-0.74655700	3.05487000
H	-3.15285500	-1.55299800	2.66474900
H	-0.15049400	-0.01039100	-5.26095200
O	0.90141500	0.74572400	2.69268700
O	-3.25694300	0.48122200	3.15376400
H	-2.21065200	-0.99165100	4.07078400
H	-1.64849800	-0.62560900	2.41714900
H	0.70354000	2.81925200	2.52876900
H	-0.66252300	1.89440900	3.20499500
C	0.67602200	-2.29613200	1.06546500
C	-0.18228300	-3.31363400	1.79797500
O	0.54433100	-2.46477600	-0.36581600
H	1.73204200	-2.37971600	1.31022900
C	1.73344800	-2.75390700	-1.25035500
C	1.85792900	-4.26843200	-1.45927700
C	4.10778000	-2.41106400	-1.10626700
C	2.67208500	-5.02637400	-0.40074100
C	4.80653300	-3.54185300	-0.36066500
C	4.19474900	-4.92776100	-0.60322400
H	2.30898400	-4.41625600	-2.44690300
H	2.40337100	-4.67896200	0.60368900
H	4.65119100	-1.47315600	-0.97849900
H	4.76596100	-3.29480400	0.70736400
H	0.83691700	-4.65489600	-1.52392000
H	4.03167600	-2.60844900	-2.18253900
H	2.39151900	-6.08553200	-0.44470200
H	5.86775600	-3.55480200	-0.64130200
H	4.69024600	-5.65205300	0.05490600
H	4.42221300	-5.24479100	-1.63112500
O	2.81082200	-2.19675900	-0.54902900
O	1.38122200	-2.08837200	-2.36218500
H	0.36607100	-1.27633600	1.29508500
H	-1.23568400	-3.13214100	1.55345700
H	0.05544800	-4.32207300	1.43601800
C	0.03538600	-3.24174400	3.31739400
H	-0.75727600	-3.80735000	3.82126400
H	-0.06974000	-2.20046300	3.64716100
C	1.39998500	-3.77853500	3.76340100
H	2.22293800	-3.20179700	3.33099800
H	1.50500400	-3.73646200	4.85235000
H	1.52878100	-4.82264400	3.45620300

INT3

C	6.91940800	-2.55924900	0.96871900
C	6.58675800	-1.49353400	0.17176200
C	5.23405200	-1.25272400	-0.19740800
C	4.19523000	-2.12848900	0.27149800
C	4.58497000	-3.23113500	1.08574200
C	5.90100100	-3.43626800	1.42292900
H	5.65888400	0.44814000	-1.44853000
H	7.95281900	-2.73810800	1.24787700
H	7.35137400	-0.81350100	-0.19454600
C	4.87764200	-0.19247300	-1.04456600
C	2.83757800	-1.88998600	-0.08518300
H	3.82141400	-3.91545300	1.43439100
H	6.16795600	-4.28550800	2.04511100
C	2.48662900	-0.78622400	-0.87743300
C	3.55521300	0.05477900	-1.38401100
C	-3.69273900	2.17913500	4.13276500
C	-3.28612000	0.88836100	4.38529200
C	-3.08478900	-0.03435400	3.32780600
C	-3.29358000	0.38441000	1.97443100
C	-3.72032500	1.72192300	1.75167200
C	-3.91501700	2.59364300	2.80002700
H	-2.56509000	-1.71382000	4.59416700
H	-3.84492800	2.87654000	4.95046400
H	-3.11723600	0.55227000	5.40501500
C	-2.71116600	-1.38228400	3.56941700
C	-3.11626800	-0.53872600	0.89024400
H	-4.24102200	3.60980900	2.60016800
C	-2.75026200	-1.84838100	1.18002600
C	-2.55729200	-2.25849400	2.52914700
H	-2.28707600	-3.29421800	2.69129200
C	1.78877000	-2.85035000	0.36060300
C	1.26642100	-3.81773900	-0.54825600
C	1.36995600	-2.87848000	1.68531100
C	1.63376700	-3.83704000	-1.92213900
C	0.34360500	-4.81071400	-0.08416800
C	0.45058900	-3.85383800	2.14278000
C	1.11385200	-4.77561800	-2.78212100
C	-0.16638000	-5.77307400	-0.99525900
C	-0.04572300	-4.79514800	1.27434100
H	0.13425700	-3.85973900	3.17719300
C	0.20447300	-5.75789900	-2.31889500

H	1.40507600	-4.76721400	-3.82832800
H	-0.86626000	-6.51686200	-0.62421500
H	-0.75527800	-5.53779500	1.62675000
H	-0.19507500	-6.49420200	-3.00932600
C	-3.41727200	-0.08161800	-0.49687600
C	-4.74073900	-0.16192200	-1.00025600
C	-2.37595400	0.42464800	-1.28880700
C	-5.82236200	-0.65663800	-0.21485700
C	-5.02834200	0.25942800	-2.34598600
C	-2.66548800	0.79556900	-2.65462100
C	-7.09627500	-0.71722900	-0.72494800
H	-5.61942700	-0.98318400	0.79815700
C	-6.36097200	0.18190900	-2.84018300
C	-3.96786700	0.71839500	-3.14058600
C	-7.37685300	-0.29343300	-2.05080400
H	-7.90385000	-1.09596900	-0.10547800
H	-6.55487100	0.50704000	-3.85897100
H	-4.16763500	1.00746100	-4.17032900
H	-8.39101300	-0.34983200	-2.43291000
C	3.27143300	1.07589400	-2.36222100
N	2.08541400	1.52420700	-2.56810900
N	-0.36695000	1.22226900	-3.31960500
C	1.73987500	2.35437600	-3.70573300
C	0.56633900	1.62025300	-4.37551100
O	1.22720600	-0.55759300	-1.18937300
O	-1.16742000	0.49516400	-0.80000100
C	-1.63150200	1.18369200	-3.57669700
Al	0.43497400	1.10049900	-1.43660200
H	4.10731500	1.43873900	-2.97214500
H	-1.97109700	1.44215300	-4.58627400
H	0.94873700	0.71481200	-4.86336400
H	2.57604600	2.48941800	-4.40215600
H	1.39825800	3.32382200	-3.33302700
H	2.33413700	-3.09306200	-2.28217000
H	-3.89527200	2.04791200	0.73325400
C	-2.04854800	-2.65557000	-1.01109000
H	-1.18414800	-1.99066200	-0.95397100
C	1.41700300	-1.84371300	3.84417400
H	0.34096500	-1.63216300	3.84900700
H	0.08005400	2.24526200	-5.13328900
O	-2.61510700	-2.86996700	0.28933400
O	1.91926500	-1.94388700	2.52030500
H	1.95308600	-1.01203700	4.30195600
H	1.61384900	-2.75453200	4.42339100
H	-2.78944000	-2.25629300	-1.70707300
H	-1.72826500	-3.63958100	-1.34984700
C	2.67121500	3.96855300	-0.32716300
O	1.45299200	4.02015200	0.41129600
H	2.45677800	4.04136600	-1.39932300
C	0.47524600	3.08206900	0.08272600
C	-0.75799200	3.38097100	0.93008700
C	0.89775100	0.98983000	1.60372700
C	-0.51709300	3.78448600	2.38681200
C	1.14491300	1.85536300	2.83441800
C	-0.08383000	2.64053600	3.30765100
H	-1.41716400	2.51415700	0.85813700
H	0.22953700	4.58432100	2.40917100
H	1.62910400	0.18540800	1.54540800
H	1.99187000	2.52329400	2.64111700
H	-1.25561900	4.19945700	0.40057400
H	-0.10119800	0.54049800	1.62851400
H	-1.44909700	4.20444400	2.78142000
H	1.45536200	1.17868400	3.63955600
H	0.11321200	3.04904900	4.30686600
H	-0.91708200	1.93825300	3.42300400
O	1.05055800	1.70096600	0.35580600
O	0.21336600	2.94729600	-1.23180300
H	3.17305900	3.01163900	-0.14215200
C	3.53476000	5.13395500	0.12901800
H	3.72106100	5.03621600	1.20626600
H	4.51177700	5.05421400	-0.36712900
C	2.90976000	6.50049500	-0.16476300
H	1.92058100	6.53965800	0.30273300
H	2.74091900	6.59134500	-1.24659700
C	3.77079500	7.66604000	0.32362800
H	3.92593300	7.61332200	1.40714800
H	4.75926200	7.65692300	-0.15038800
H	3.30414500	8.63102800	0.10149800

TS2

C	7.04226400	-2.33453600	1.01739200
C	6.67331300	-1.28271800	0.21834100
C	5.31328100	-1.09055700	-0.15339500
C	4.30533400	-2.00335900	0.31302000
C	4.73259900	-3.08835400	1.13305800
C	6.05421900	-3.24561500	1.47300500
H	5.67584500	0.63059800	-1.39470500
H	8.08089900	-2.47630200	1.29858600

H	7.41372500	-0.57589000	-0.14716800
C	4.91870600	-0.04194500	-0.99658600
C	2.94190100	-1.81826500	-0.04944200
H	3.99259900	-3.79712000	1.48380800
H	6.34987300	-4.08253400	2.09893800
C	2.54930200	-0.73081700	-0.84548500
C	3.58763900	0.15831400	-1.33570200
C	-3.86119400	2.08834100	4.07281800
C	-3.38144900	0.83147700	4.36397800
C	-3.11504600	-0.10563700	3.33402200
C	-3.32669900	0.26671800	1.96768100
C	-3.83473400	1.56785300	1.70464200
C	-4.09677900	2.45204800	2.72730300
H	-2.52195500	-1.72138800	4.64977900
H	-4.06467100	2.79648500	4.86993400
H	-3.20474100	0.53305200	5.39402300
C	-2.67041800	-1.42391800	3.61502200
C	-3.07229200	-0.66786700	0.91058100
H	-4.48708700	3.43907300	2.49775600
C	-2.63852600	-1.94763600	1.23766800
C	-2.44967000	-2.31504700	2.59948500
H	-2.12503800	-3.32982600	2.79165600
C	1.93107700	-2.80931200	0.41541200
C	1.47074800	-3.83593000	-0.45964700
C	1.49402700	-2.80234100	1.73476100
C	1.85699500	-3.88802800	-1.82742900
C	0.58912400	-4.85329500	0.03149400
C	0.61774700	-3.80442900	2.21929500
C	1.39215400	-4.88053700	-2.65790200
C	0.13587500	-5.87059300	-0.84929400
C	0.18064900	-4.80320000	1.38378400
H	0.29017500	-3.78548100	3.25017500
C	0.52269200	-5.88617400	-2.16850900
H	1.69547400	-4.89671000	-3.70062500
H	-0.53418600	-6.63206200	-0.45939000
H	-0.49637200	-5.56571100	1.75733400
H	0.16588000	-6.66466300	-2.83573900
C	-3.36559500	-0.26533800	-0.49417400
C	-4.66556800	-0.45403500	-1.02934500
C	-2.33761600	0.28421900	-1.27505600
C	-5.73414600	-0.99140900	-0.25415000
C	-4.94105200	-0.10493700	-2.39770900
C	-2.60976800	0.57273700	-2.66334800
C	-6.98607400	-1.15645200	-0.79479000

H	-5.53938300	-1.26527300	0.77592400
C	-6.25048000	-0.29037300	-2.92371100
C	-3.88816800	0.38866400	-3.18192200
C	-7.25541800	-0.80253600	-2.14318600
H	-7.78440300	-1.56517200	-0.18241100
H	-6.43552000	-0.01844900	-3.95962700
H	-4.07325500	0.61715300	-4.22953100
H	-8.25206900	-0.94142900	-2.54959500
C	3.27027700	1.16587500	-2.31515100
N	2.07791200	1.61190000	-2.50253900
N	-0.32163000	1.10038400	-3.28497200
C	1.71289000	2.34859500	-3.69718500
C	0.61077800	1.49255300	-4.34244300
O	1.28797500	-0.55607100	-1.17810100
O	-1.15492000	0.47110300	-0.75355000
C	-1.57228000	0.97612100	-3.57464000
Al	0.45530700	1.09964800	-1.35543600
H	4.08560200	1.51489900	-2.96053200
H	-1.89590400	1.16238900	-4.60546500
H	1.07018900	0.58619700	-4.75660100
H	2.55774900	2.48996500	-4.38207400
H	1.30006600	3.31615400	-3.40402100
H	2.52557400	-3.12383700	-2.20577800
H	-4.01907900	1.85409900	0.67590900
C	-1.84428700	-2.76334500	-0.91947600
H	-1.01674500	-2.05303500	-0.85962700
C	1.41587400	-1.64103400	3.82526100
H	0.33247000	-1.48492200	3.76451100
H	0.10491600	2.02893000	-5.15392200
O	-2.43008000	-2.97858700	0.37276000
O	1.98710300	-1.81323400	2.53744100
H	1.88737500	-0.75031700	4.24095600
H	1.62747900	-2.49453400	4.48212000
H	-2.58823100	-2.42066500	-1.64168900
H	-1.46516500	-3.73650800	-1.22696300
C	2.13631700	4.53092800	-0.69597900
O	0.98190100	4.36904100	0.14661800
H	1.81035000	4.84193300	-1.69453600
C	0.10820100	3.40451400	-0.17565700
C	-1.07600800	3.37430600	0.75683500
C	1.11276800	1.08894200	1.49721800
C	-0.81438300	3.73442500	2.22148200
C	1.11012300	2.03321700	2.69803000
C	-0.26400200	2.59680400	3.08844300

H	-1.53384000	2.39060400	0.65973000
H	-0.14425100	4.59922900	2.26801900
H	2.02717400	0.48781600	1.51268900
H	1.82540200	2.84498600	2.51387500
H	-1.78186000	4.09331300	0.31807500
H	0.26668600	0.39108500	1.57229200
H	-1.76744600	4.04936800	2.65842900
H	1.49858900	1.46623200	3.55371900
H	-0.21172100	2.96607200	4.12073800
H	-0.98990500	1.77551400	3.09853800
O	1.06430700	1.78680700	0.26326600
O	-0.01656200	3.02923300	-1.39644300
H	2.63915800	3.56531700	-0.77103100
C	3.02451500	5.58015100	-0.05157400
H	3.31730400	5.22860800	0.94558000
H	3.94725000	5.65062600	-0.64310700
C	2.36786100	6.95958800	0.05481900
H	1.43399800	6.86377300	0.61911100
H	2.08643800	7.30180000	-0.95044400
C	3.27515800	7.99637400	0.71862100
H	3.54386900	7.68978300	1.73558500
H	4.20716400	8.12654900	0.15672700
H	2.78625300	8.97331500	0.78389200

INT4

C	7.07821500	-2.26322700	1.03359200
C	6.69350300	-1.21051100	0.24324100
C	5.33187100	-1.04023200	-0.13341100
C	4.33943000	-1.97708400	0.31766300
C	4.78249500	-3.06095200	1.13070800
C	6.10511000	-3.19675900	1.47593500
H	5.66573400	0.70668700	-1.34566100
H	8.11794500	-2.38827900	1.31871800
H	7.42215600	-0.48586200	-0.11087100
C	4.92047200	0.01195300	-0.96361500
C	2.97493200	-1.81553200	-0.05145400
H	4.05370400	-3.78596700	1.47170300
H	6.41324000	-4.03355900	2.09599300
C	2.56574300	-0.73089300	-0.84154700
C	3.58740000	0.19020100	-1.30814500
C	-3.90770900	2.06381400	4.05987700
C	-3.40806400	0.81820600	4.36539500
C	-3.12438200	-0.12536200	3.34621500
C	-3.33683900	0.23047700	1.97573800
C	-3.86792700	1.51902900	1.69752200
C	-4.14802200	2.40894800	2.71025100
H	-2.50947300	-1.71831500	4.67939600
H	-4.12443500	2.77692900	4.84904300
H	-3.22871100	0.53385600	5.39893600
C	-2.65907600	-1.43336300	3.64133800
C	-3.05801300	-0.70808400	0.92945900
H	-4.55639300	3.38616700	2.47004500
C	-2.60408700	-1.97719700	1.26933500
C	-2.41758500	-2.33000700	2.63516400
H	-2.07609500	-3.33720300	2.83821400
C	1.97915900	-2.81952500	0.41753000
C	1.53891500	-3.86462700	-0.44464100
C	1.53552300	-2.79889200	1.73481900
C	1.93338500	-3.92986800	-1.80944800
C	0.66740400	-4.88624700	0.05617400
C	0.66972800	-3.80550300	2.22896100
C	1.48530300	-4.93900800	-2.62900300
C	0.23116800	-5.92028000	-0.81364400
C	0.25041700	-4.82188100	1.40550800
H	0.33741700	-3.77570600	3.25819000
C	0.62522000	-5.94838300	-2.13059000
H	1.79424800	-4.96547800	-3.66988300
H	-0.43171200	-6.68481200	-0.41741400
H	-0.41890300	-5.58769600	1.78619800
H	0.28128300	-6.73986800	-2.78926800
C	-3.34747100	-0.32438200	-0.48063100
C	-4.63441500	-0.56001100	-1.02933400
C	-2.32868800	0.24945700	-1.25504200
C	-5.69428900	-1.12304100	-0.26065000
C	-4.90424500	-0.23487200	-2.40455300
C	-2.59251700	0.51183800	-2.64880900
C	-6.93377900	-1.33390600	-0.81393100
H	-5.50309000	-1.37927700	0.77460300
C	-6.20061700	-0.46784500	-2.94383100
C	-3.85748900	0.28239300	-3.18169600
C	-7.19792100	-1.00344000	-2.16925800
H	-7.72598100	-1.76132800	-0.20641200
H	-6.38170800	-0.21354900	-3.98489800
H	-4.03545000	0.49205600	-4.23445400
H	-8.18462400	-1.17878200	-2.58574000
C	3.25957800	1.20889800	-2.27208500
N	2.06499700	1.65318700	-2.45725900
N	-0.31097100	1.08994200	-3.24504800

C	1.70118400	2.37226000	-3.66302100
C	0.61873100	1.48935100	-4.30291800
O	1.30403700	-0.58312200	-1.19146200
O	-1.15989200	0.48953800	-0.72077300
C	-1.55426600	0.93359100	-3.54946700
Al	0.47196100	1.07279700	-1.30267700
H	4.07137900	1.56750200	-2.91668500
H	-1.86882400	1.10325500	-4.58592500
H	1.09722200	0.58738600	-4.70473000
H	2.54988300	2.51438600	-4.34294300
H	1.27284500	3.33819700	-3.38923700
H	2.59402500	-3.16157300	-2.19376400
H	-4.05583100	1.79024900	0.66533100
C	-1.77216000	-2.79178200	-0.87322900
H	-0.95515900	-2.06980700	-0.80611900
C	1.41372100	-1.58685900	3.79385000
H	0.33009100	-1.44725300	3.70656300
H	0.10526200	2.00628800	-5.12217200
O	-2.37280500	-3.00953900	0.41194700
O	2.01288900	-1.79333500	2.52394400
H	1.86474500	-0.67600200	4.18798300
H	1.62325300	-2.41554000	4.48267800
H	-2.51064300	-2.46347900	-1.60758800
H	-1.37482800	-3.76067800	-1.17069800
C	1.92224100	4.75198700	-0.81859200
O	0.74726200	4.59332100	0.00822500
H	1.61857600	5.16823700	-1.78565900
C	-0.12014500	3.65210800	-0.33571300
C	-1.23656500	3.46766300	0.65058000
C	1.21269800	1.12605500	1.45924400
C	-0.92338400	3.74888700	2.12616600
C	1.06086200	2.09056400	2.63580800
C	-0.37194300	2.56486400	2.93398500
H	-1.61266900	2.45541100	0.50727100
H	-0.24403400	4.60437800	2.20130400
H	2.19502600	0.63882400	1.52707600
H	1.72424800	2.95086700	2.47377900
H	-2.02813900	4.14802300	0.30248200
H	0.46181600	0.32603100	1.55378200
H	-1.86140100	4.05207900	2.60171800
H	1.43729900	1.57579400	3.53027300
H	-0.42373600	2.85822100	3.99026800
H	-1.05582700	1.71529300	2.82844300
O	1.08769800	1.79617700	0.23018900
O	-0.12807500	3.14665700	-1.47785200
H	2.35289800	3.76161900	-0.96964600
C	2.87045900	5.67887500	-0.08158500
H	3.12918400	5.21893100	0.87992300
H	3.80062400	5.73617200	-0.66263000
C	2.30984800	7.08596000	0.14444200
H	1.36777600	7.00729900	0.69794900
H	2.06061200	7.53446900	-0.82696500
C	3.28249400	7.99374500	0.89859700
H	3.52124300	7.58032100	1.88462200
H	4.22528300	8.10693500	0.35138700
H	2.86239000	8.99290900	1.04918000

***si*-enantioface: *R,R*-Al-*S*-M2B + CL**

INT1

C	-7.48251100	-1.80356800	0.10153600
C	-6.90662200	-0.72602100	0.72392600
C	-5.49191000	-0.56940100	0.75457200
C	-4.64634500	-1.54179600	0.11570600
C	-5.28597100	-2.65631700	-0.50182000
C	-6.65346900	-2.78100200	-0.50870400
H	-5.51268100	1.22581600	1.94236800
H	-8.56160000	-1.91869400	0.08187500
H	-7.51999300	0.02742600	1.21178400
C	-4.88552900	0.50408900	1.42250600
C	-3.23245500	-1.38807600	0.13315800
H	-4.66866200	-3.41801000	-0.96167700
H	-7.10988600	-3.64422200	-0.98449100
C	-2.64022200	-0.29415200	0.79292700
C	-3.50642700	0.66177400	1.44904100
C	5.35769900	2.86562000	-1.25992800
C	4.97438900	2.17673200	-2.38617700
C	4.28233700	0.94063100	-2.28612000
C	3.95666200	0.41475300	-0.99360400
C	4.35728000	1.16264800	0.14925700
C	5.04299500	2.34761700	0.02027400
H	4.16581100	0.59126400	-4.41699400
H	5.90542400	3.79913700	-1.34698700
H	5.21271200	2.55788500	-3.37573800
C	3.93268400	0.19092900	-3.43431600
C	3.29017400	-0.84406700	-0.88031200
H	5.34495600	2.89358800	0.90901200
C	3.00923200	-1.55757900	-2.03889900

C	3.32326500	-1.03440800	-3.31825800	H	-2.00391500	3.01886700	4.19055400
H	3.06390300	-1.59269200	-4.20909800	H	-0.60115000	3.55786900	3.21921400
C	-2.35583400	-2.35333400	-0.58409600	H	-1.92520600	-2.47698100	2.09254300
C	-1.40829800	-3.16421200	0.10991400	H	4.12428600	0.77379600	1.13241200
C	-2.43988200	-2.45554600	-1.97082300	C	2.48666900	-3.69188400	-2.95832400
C	-1.27244000	-3.12821800	1.52499100	H	1.78721100	-3.43267800	-3.76257700
C	-0.56013000	-4.05651400	-0.62205700	C	-3.36284700	-1.57785900	-4.00037300
C	-1.63214300	-3.36767000	-2.69074500	H	-4.09209600	-0.80818800	-4.25608900
C	-0.33024800	-3.89589100	2.16548600	H	0.44536800	2.08163400	4.80855000
C	0.40962700	-4.82853800	0.07054000	O	2.41473400	-2.77664500	-1.88076300
C	-0.71855600	-4.14767200	-2.02327300	O	-3.34465100	-1.63554900	-2.58719800
H	-1.72894700	-3.45187000	-3.76587800	H	-3.67725100	-2.53063300	-4.44621400
C	0.53360000	-4.74753200	1.43483100	H	-2.38349500	-1.29524600	-4.40556400
H	-0.23740300	-3.84242700	3.24590500	H	2.20868200	-4.66189700	-2.54681500
H	1.06017300	-5.48305700	-0.50274800	H	3.50284100	-3.75305400	-3.36735300
H	-0.10633000	-4.85176300	-2.57692300	C	-1.03816900	2.22948500	-1.59632700
H	1.29419800	-5.32283500	1.95136300	C	-2.33364100	3.04241300	-1.51795700
C	2.92799500	-1.37346300	0.46233700	O	-2.40510300	4.24900100	-1.67013500
C	3.57902500	-2.50997500	1.00849200	O	-3.39746700	2.28463500	-1.19209000
C	1.95468800	-0.67904400	1.20547800	C	-4.62982500	2.98587200	-0.99208900
C	4.54534800	-3.26395200	0.28022600	H	-4.54280100	3.67867400	-0.15044800
C	3.28413000	-2.93653300	2.35036200	H	-4.89917700	3.55333600	-1.88613200
C	1.75202500	-1.05546200	2.58670000	H	-5.37078400	2.21704700	-0.77890100
C	5.13079500	-4.38260900	0.82017700	O	-0.49202100	2.22854500	-0.31825900
H	4.81518100	-2.93633700	-0.71599500	H	-0.38002000	2.82591800	-2.25460800
C	3.91169800	-4.09860400	2.87995000	C	-1.23509000	0.86280800	-2.25649100
C	2.40114900	-2.16357000	3.11642200	H	-1.94563000	0.27835700	-1.67518300
C	4.80728000	-4.81945800	2.13186300	H	-1.70698000	1.04053100	-3.23389100
H	5.85810300	-4.94096600	0.23768000	C	0.86336800	3.81452700	0.75978800
H	3.66399700	-4.40185800	3.89409700	C	1.77771500	3.88064800	-0.43260800
H	2.22570600	-2.43547600	4.15528600	C	-0.24604700	5.86421200	0.06570800
H	5.27880500	-5.70812800	2.53918300	C	2.73061400	5.09166700	-0.40244700
C	-2.95805700	1.68874300	2.29316500	C	0.89502500	6.86722200	-0.03870500
N	-1.71907100	2.04477100	2.32154800	C	2.08504700	6.39599400	-0.88056500
N	0.29327000	0.79866500	3.13220900	H	1.17233100	3.91266700	-1.34304500
C	-1.20298200	2.70018600	3.51228200	H	3.14628600	5.21530300	0.60583500
C	-0.32581000	1.61966000	4.18273500	H	-1.14147900	6.33774900	0.47020400
O	-1.33885700	-0.18276500	0.84487700	H	1.22055900	7.12450500	0.97732200
O	1.31353700	0.32397600	0.66879100	H	2.32953800	2.94232500	-0.43832600
C	0.98080400	-0.23436400	3.48131500	H	-0.52148100	5.40842800	-0.88786600
Al	-0.20557800	1.23083300	1.15865800	H	3.57587600	4.85205100	-1.05243800
H	-3.64648500	2.12883900	3.02550800	H	0.48504300	7.78517200	-0.47740300
H	1.03435000	-0.50676100	4.54285700	H	2.84178400	7.18886500	-0.90462300
H	-0.95348600	0.98621800	4.82291600	H	1.74870300	6.25743900	-1.91710500

O	0.02913500	4.81233000	1.03073100
O	0.95592200	2.92912700	1.62793400
C	0.07492900	0.10037400	-2.43282400
H	0.53461200	-0.11167500	-1.46864900
H	-0.09463400	-0.85649000	-2.93592700
H	0.79568200	0.67324100	-3.02769200

TS1

C	-7.29570900	-2.39107500	0.16560600
C	-6.80778800	-1.29264500	0.82594600
C	-5.41606000	-0.99538900	0.82050600
C	-4.50143900	-1.84474900	0.10711700
C	-5.04843400	-2.98587300	-0.54932800
C	-6.39642900	-3.24756100	-0.52119800
H	-5.56916400	0.72645000	2.10481000
H	-8.35799300	-2.61362800	0.17329200
H	-7.47530400	-0.62972900	1.37071000
C	-4.89404000	0.09982800	1.52476100
C	-3.10896200	-1.55049100	0.09168000
H	-4.37580100	-3.65800200	-1.06743600
H	-6.78149300	-4.12742600	-1.02852300
C	-2.60467800	-0.43450400	0.78420200
C	-3.53739400	0.39281900	1.51695000
C	5.19405300	3.18581600	-1.10596000
C	4.86002900	2.50477300	-2.25214700
C	4.23952100	1.22894100	-2.18876400
C	3.93148900	0.65386400	-0.91307300
C	4.28003100	1.39460700	0.25124000
C	4.89940100	2.61859500	0.15785300
H	4.16561900	0.92600600	-4.32860400
H	5.68674400	4.15163800	-1.16500200
H	5.08385300	2.92348300	-3.22981100
C	3.94535100	0.48914600	-3.35858900
C	3.33389500	-0.64270500	-0.83686900
H	5.16140900	3.15873500	1.06251000
C	3.10866800	-1.34267700	-2.01587000
C	3.40689100	-0.77156600	-3.27852800
H	3.19078100	-1.32271500	-4.18520300
C	-2.17049800	-2.41280500	-0.67660700
C	-1.17400700	-3.19714200	-0.02275100
C	-2.26759500	-2.46806800	-2.06425900
C	-1.01921600	-3.19977100	1.39087400
C	-0.29871600	-4.02704000	-0.79513500
C	-1.42520100	-3.31199200	-2.82649100
C	-0.03779500	-3.94853000	1.99372000
C	0.70793200	-4.78386000	-0.13948700
C	-0.46996700	-4.07430300	-2.19711100
H	-1.52988100	-3.36146900	-3.90300000
C	0.84771500	-4.74202200	1.22489900
H	0.06962300	-3.92457700	3.07356200
H	1.37370400	-5.39629100	-0.74084900
H	0.16627900	-4.72935000	-2.78311700
H	1.63725800	-5.30268200	1.71334900
C	2.98521200	-1.21837700	0.49078900
C	3.68487200	-2.33230400	1.02213400
C	1.97624200	-0.58229600	1.24059300
C	4.68825200	-3.02973200	0.28727800
C	3.40446700	-2.79342600	2.35614200
C	1.78872300	-0.98675100	2.61710300
C	5.31972700	-4.13051500	0.81135500
H	4.94872600	-2.67320600	-0.70134700
C	4.08060200	-3.93560600	2.86950300
C	2.48713800	-2.07234000	3.13183000
C	5.00966600	-4.60397300	2.11380200
H	6.07403900	-4.64592000	0.22364700
H	3.84249800	-4.26626000	3.87734800
H	2.32345800	-2.36695300	4.16646400
H	5.51815900	-5.47784400	2.50843300
C	-3.06386400	1.43714100	2.38437100
N	-1.85977100	1.89361100	2.39699600
N	0.24698800	0.79155000	3.18953900
C	-1.37640400	2.58417600	3.58186800
C	-0.41410800	1.57218100	4.24529800
O	-1.31889400	-0.19163600	0.79414500
O	1.29038200	0.39650700	0.71765600
C	0.98038400	-0.21544300	3.52350800
Al	-0.25757300	1.24334600	1.24009800
H	-3.77356700	1.79898500	3.13907300
H	1.04379200	-0.50236600	4.58062100
H	-0.98268000	0.90009800	4.90125800
H	-2.18850400	2.84832800	4.27020500
H	-0.83836500	3.47843500	3.27295100
H	-1.68958300	-2.59417200	1.98772800
H	4.06194300	0.96968700	1.22270900
C	2.74355900	-3.48337800	-2.99156100
H	2.05338200	-3.25027600	-3.81161700
C	-3.30158600	-1.60997100	-4.04821700
H	-4.08413000	-0.88326900	-4.27004100

H	0.33250400	2.09355000	4.85400400
O	2.58363400	-2.59821500	-1.89819500
O	-3.22296800	-1.67283800	-2.63727300
H	-3.57737400	-2.57763700	-4.48716300
H	-2.35752800	-1.26941300	-4.49156500
H	2.51466300	-4.47770900	-2.60908300
H	3.77298000	-3.47227600	-3.37065600
C	-1.11063000	2.28563400	-1.60257200
C	-2.45831200	3.00280400	-1.55231700
O	-2.70016400	4.06460700	-2.09450300
O	-3.36373300	2.31924900	-0.83057100
C	-4.65855100	2.92324400	-0.72224400
H	-4.59680900	3.85835700	-0.15876400
H	-5.06686000	3.13567000	-1.71268400
H	-5.27455600	2.19558300	-0.19680200
O	-0.53708200	2.25786400	-0.32477600
H	-0.48036400	2.91333200	-2.24898900
C	-1.23984500	0.90659400	-2.25833200
H	-1.91390600	0.29135500	-1.66503300
H	-1.72615200	1.05909500	-3.23234800
C	0.39040900	3.66888400	0.53341400
C	1.50759700	3.88810100	-0.45978400
C	-0.69613200	5.70708300	-0.23898900
C	2.34526700	5.13292900	-0.11198900
C	0.36043800	6.78911800	-0.04686400
C	1.73778500	6.44415200	-0.62433000
H	1.11405600	3.98740300	-1.47555200
H	2.50241800	5.17599400	0.97294500
H	-1.69309000	6.09575300	-0.02520200
H	0.43686700	6.99776000	1.02756600
H	2.11420900	2.98433900	-0.42605200
H	-0.71912100	5.31387900	-1.25774700
H	3.33251100	5.00215700	-0.56193600
H	-0.00866500	7.70716100	-0.52097600
H	2.42795000	7.27052800	-0.41653000
H	1.65393000	6.38156600	-1.71829300
O	-0.53599500	4.63255800	0.71459700
O	0.60006800	2.95405100	1.57504000
C	0.10958200	0.21679800	-2.43614600
H	0.59006800	0.05195300	-1.47263600
H	-0.01386000	-0.75790700	-2.91665500
H	0.78805800	0.81691100	-3.05291500

INT2

C	-6.85319800	-3.42735900	0.17745800
C	-6.53173100	-2.27964800	0.85612800
C	-5.20234800	-1.77249000	0.84454700
C	-4.17819200	-2.45823600	0.10494100
C	-4.55023700	-3.65753200	-0.56888400
C	-5.84209600	-4.12317100	-0.53439300
H	-5.59767200	-0.12942200	2.18016900
H	-7.86880000	-3.80996500	0.18982800
H	-7.28658900	-1.73778100	1.42019000
C	-4.84399200	-0.62757700	1.57296600
C	-2.84816900	-1.95129800	0.08217900
H	-3.78841300	-4.20828300	-1.10634800
H	-6.09423900	-5.04175900	-1.05626500
C	-2.51675300	-0.78090600	0.78509900
C	-3.54826700	-0.13061300	1.55985100
C	4.50339700	3.91775600	-1.22897900
C	4.27515600	3.16599000	-2.35631100
C	3.90477600	1.79840600	-2.25899800
C	3.73277600	1.20103000	-0.96821500
C	3.96935300	2.01346100	0.17583300
C	4.35323400	3.32740100	0.04917400
H	3.85197500	1.44567000	-4.39171300
H	4.80077400	4.95844700	-1.31380800
H	4.39312500	3.60110500	-3.34520200
C	3.73267500	0.99447800	-3.41064300
C	3.38141900	-0.18148800	-0.85905900
H	4.53364800	3.92217700	0.93917100
C	3.27036100	-0.93345100	-2.02249400
C	3.43935500	-0.34230700	-3.29963800
H	3.31620600	-0.94215100	-4.19272300
C	-1.78393900	-2.65849700	-0.67975600
C	-0.68582200	-3.27787000	-0.01061300
C	-1.85461000	-2.72855100	-2.06739700
C	-0.54771500	-3.25209400	1.40471800
C	0.31701700	-3.96183600	-0.76987600
C	-0.88268000	-3.43355500	-2.81711200
C	0.53387800	-3.83312600	2.02114600
C	1.42297200	-4.54874900	-0.10037900
C	0.17212400	-4.03636500	-2.17384400
H	-0.96428600	-3.49920900	-3.89475200
C	1.54111600	-4.47988000	1.26494900
H	0.62468400	-3.78870100	3.10193200
H	2.18355800	-5.04961000	-0.69185900
H	0.91064500	-4.58350700	-2.75012800

H	2.40401200	-4.90794400	1.76343100
C	3.16472600	-0.78294100	0.48542500
C	4.05231100	-1.75351700	1.01653600
C	2.08142600	-0.30994500	1.25339500
C	5.14727800	-2.28120600	0.27045800
C	3.87895700	-2.23543600	2.36215700
C	1.99691000	-0.71193200	2.64127500
C	5.96400100	-3.25262500	0.79361400
H	5.32856700	-1.89739200	-0.72548400
C	4.74589200	-3.24248000	2.87333000
C	2.87400000	-1.66301700	3.15173700
C	5.75983600	-3.75557500	2.10624800
H	6.78530400	-3.64013500	0.19756800
H	4.58529200	-3.59408500	3.88932400
H	2.78421200	-1.96396200	4.19352900
H	6.41465100	-4.52667400	2.49927800
C	-3.21992400	0.94834900	2.45384000
N	-2.07900700	1.53996600	2.47857000
N	0.20550300	0.81057600	3.23552000
C	-1.67719500	2.32036400	3.63561500
C	-0.56146200	1.48022900	4.29706400
O	-1.28360600	-0.33570100	0.76370900
O	1.22736200	0.52878300	0.73684600
C	1.09693800	-0.06722100	3.55813900
Al	-0.39515400	1.16015700	1.32402000
H	-3.97775800	1.21465400	3.20137900
H	1.22304900	-0.33114400	4.61514000
H	-1.00767800	0.72878400	4.96128000
H	-2.50238400	2.48778900	4.33837800
H	-1.27794300	3.27048000	3.28111300
H	-1.31273300	-2.76115300	1.99305400
H	3.85753400	1.57451600	1.15893600
C	3.32531600	-3.12473400	-2.95047400
H	2.61738400	-3.03962300	-3.78400900
C	-3.00368800	-2.06854800	-4.06452500
H	-3.89101000	-1.47885400	-4.29793900
H	0.09785500	2.11844600	4.89512200
O	2.98823800	-2.26336200	-1.87849200
O	-2.91164800	-2.08443700	-2.65268100
H	-3.12531200	-3.07795700	-4.47815900
H	-2.12608400	-1.59540200	-4.52271500
H	3.27845500	-4.13747500	-2.54987700
H	4.34039900	-2.93047300	-3.31832800
C	-1.40823700	2.17463400	-1.69755100

C	-2.84099500	2.68042100	-1.58270300
O	-3.31499000	3.53119100	-2.30789800
O	-3.53583900	2.01980700	-0.64298600
C	-4.89632100	2.43957000	-0.47853400
H	-4.92828300	3.44677100	-0.05450600
H	-5.41721000	2.43962700	-1.43793800
H	-5.34285100	1.71870200	0.20355400
O	-0.74806200	2.15314300	-0.43698700
H	-0.88817400	2.88549200	-2.34660400
C	-1.36425800	0.78168000	-2.32643700
H	-1.93521200	0.09439600	-1.70307000
H	-1.88891700	0.85226600	-3.28793600
C	-0.33558400	3.43662200	0.22745900
C	0.82943700	4.04239300	-0.55104000
C	-1.64739900	5.45194200	-0.38860400
C	1.40455500	5.29366500	0.12488300
C	-0.82284700	6.59187800	0.20599000
C	0.65186500	6.58593700	-0.20720500
H	0.54960400	4.26834700	-1.58601800
H	1.42338700	5.12307800	1.20714600
H	-2.71416600	5.67525500	-0.30897000
H	-0.91686000	6.53786400	1.29765100
H	1.58127400	3.25292600	-0.58142400
H	-1.43651400	5.31941800	-1.45632800
H	2.44481800	5.40413500	-0.19354000
H	-1.27831900	7.54061900	-0.10652200
H	1.16123700	7.43525100	0.26465000
H	0.71129800	6.75763500	-1.29162600
O	-1.50404600	4.22096600	0.32776900
O	0.02016500	2.96172100	1.42817600
C	0.06344000	0.27601000	-2.51985900
H	0.57706700	0.19074200	-1.56237800
H	0.06053100	-0.71368600	-2.98417400
H	0.64476700	0.95034900	-3.15782300

INT3

C	-7.74522900	-0.52645300	0.60215800
C	-6.97370000	-1.30919000	-0.21823300
C	-5.62458000	-0.95933300	-0.50317600
C	-5.04560400	0.21971800	0.07809800
C	-5.88843800	1.01436800	0.90912300
C	-7.18962300	0.65219800	1.16176500
H	-5.26820600	-2.60365600	-1.84721100
H	-8.77410200	-0.79689700	0.81680400

H	-7.37927000	-2.21099300	-0.66931100
C	-4.83406400	-1.72702500	-1.37093400
C	-3.68662000	0.56600200	-0.18879200
H	-5.49025300	1.92601300	1.33617600
H	-7.80563500	1.28207800	1.79697100
C	-2.89465200	-0.23060500	-1.03144600
C	-3.51455800	-1.39556400	-1.63747500
C	4.99055200	-0.32764200	4.02585400
C	3.73796600	0.12575300	4.37055800
C	2.87355700	0.69660100	3.40283600
C	3.30260800	0.80950500	2.04070500
C	4.58718800	0.29183900	1.71444300
C	5.40981500	-0.24719800	2.67897700
H	1.23966200	1.05668200	4.77794300
H	5.64652200	-0.75777800	4.77609200
H	3.38816900	0.05582100	5.39724500
C	1.57581900	1.15645800	3.74924900
C	2.44512600	1.41907100	1.05624800
H	6.38671800	-0.62788700	2.39611300
C	1.20561500	1.90380400	1.46665500
C	0.77191900	1.74203500	2.81188600
H	-0.20832600	2.13181100	3.05686800
C	-3.13479000	1.79599800	0.44742700
C	-2.90977500	2.98224400	-0.30983200
C	-2.96200900	1.84073800	1.82693800
C	-3.04100200	3.00943200	-1.72567200
C	-2.56066400	4.20172500	0.35802400
C	-2.61554500	3.04312600	2.48866600
C	-2.82963300	4.16783800	-2.43541200
C	-2.36321500	5.38460700	-0.40354800
C	-2.42373500	4.19496900	1.76447100
H	-2.50385100	3.06245800	3.56544900
C	-2.48858600	5.37228800	-1.77233500
H	-2.92752400	4.16207800	-3.51695900
H	-2.10214100	6.30022500	0.12050300
H	-2.15987500	5.11764000	2.27322000
H	-2.32979900	6.28000800	-2.34625900
C	2.84988600	1.39840500	-0.37492200
C	3.97434300	2.11863400	-0.85308300
C	2.07508700	0.62153700	-1.25486800
C	4.72488600	2.99842400	-0.01726000
C	4.38307100	2.00055600	-2.22848400
C	2.47791900	0.53297100	-2.63815700
C	5.81589400	3.67839000	-0.50010700
H	4.41649900	3.12010100	1.01405000
C	5.52628300	2.71273900	-2.69113000
C	3.61687100	1.19728800	-3.08440400
C	6.23435000	3.53124000	-1.84928800
H	6.36768100	4.34190300	0.15913600
H	5.81932300	2.59915000	-3.73159900
H	3.90947000	1.10797300	-4.12852600
H	7.10291800	4.07350100	-2.20863600
C	-2.80288300	-2.18125100	-2.61419300
N	-1.52829900	-2.12086600	-2.75524700
N	0.61130700	-0.83371000	-3.38136000
C	-0.83217400	-2.71584200	-3.87970400
C	-0.02912500	-1.55515400	-4.48558700
O	-1.64412000	0.09416800	-1.29469600
O	1.00206800	0.02281500	-0.81805900
C	1.72572000	-0.22000500	-3.60320200
Al	-0.24026300	-1.09765300	-1.53155600
H	-3.39649500	-2.82345100	-3.27560100
H	2.15130500	-0.25306700	-4.61275900
H	-0.72239600	-0.87295800	-4.99378900
H	-1.51479100	-3.15556300	-4.61657300
H	-0.14606000	-3.47539200	-3.49651000
H	-3.30577700	2.09380700	-2.23930400
H	4.91499300	0.31328800	0.68530200
C	0.63829800	3.27463000	-0.46822200
H	0.60015700	2.63465200	-1.35066000
C	-3.10445600	0.67095700	3.91598600
H	-2.09236900	0.90697700	4.26822300
H	0.70639400	-1.91144100	-5.21558600
O	0.28620400	2.55173900	0.70826400
O	-3.17979600	0.66924500	2.50191400
H	-3.36280200	-0.34123500	4.22772300
H	-3.81679600	1.37710400	4.36009000
H	1.63123000	3.72284800	-0.37658500
H	-0.11244900	4.05709200	-0.56598600
C	2.11500500	-2.57248200	0.93013400
C	3.10887500	-2.94310900	-0.16483900
O	3.37024200	-4.05987300	-0.54870900
O	3.74730900	-1.83135800	-0.60088900
C	4.75030100	-2.04751400	-1.59815600
H	4.30691100	-2.49419800	-2.49176400
H	5.15528300	-1.06197100	-1.82592200
H	5.53175100	-2.71373800	-1.22371100
O	0.97367700	-3.42657600	0.86099200

H	1.82902600	-1.52818400	0.78991400
C	2.73032800	-2.76390100	2.32013700
H	3.62708300	-2.14358800	2.37473800
H	2.02483400	-2.35002800	3.04892200
C	0.10413700	-3.20297500	-0.20330500
C	-0.77561500	-4.45583500	-0.33475900
C	-1.07460100	-1.61945300	1.47819600
C	-2.09882800	-4.45720100	0.44796800
C	-2.22147400	-2.43875100	2.04394700
C	-2.03769800	-3.95846100	1.90605700
H	-0.14959300	-5.29837600	-0.02851200
H	-2.83804100	-3.85272500	-0.08961900
H	-1.32235800	-0.56014300	1.43209400
H	-3.15312900	-2.13416700	1.55381600
H	-0.99016900	-4.58402600	-1.39851000
H	-0.16759300	-1.74462400	2.07345200
H	-2.47853600	-5.48580300	0.43553500
H	-2.32379800	-2.17482100	3.10400900
H	-2.83840800	-4.44620200	2.47409400
H	-1.09229200	-4.26623900	2.36871500
O	-0.78675200	-2.00979100	0.12448800
O	0.66827700	-2.74477400	-1.33115000
C	3.05827700	-4.21651600	2.66800000
H	3.49617500	-4.27546600	3.66942300
H	2.15759700	-4.83472600	2.64725500
H	3.76771400	-4.64487200	1.95535900

TS2

C	-7.80048500	-0.72277500	0.62921500
C	-6.99713200	-1.52266900	-0.14250800
C	-5.65856300	-1.14308400	-0.43920700
C	-5.12374000	0.08558600	0.07721800
C	-5.99763000	0.89342900	0.86240200
C	-7.28775400	0.50207500	1.12757400
H	-5.22839000	-2.85771000	-1.66765200
H	-8.82134500	-1.01552700	0.85288300
H	-7.36847100	-2.46165300	-0.54471500
C	-4.83297600	-1.93374400	-1.25067300
C	-3.77797900	0.46803000	-0.20700600
H	-5.63221100	1.83870200	1.24327800
H	-7.92834200	1.14395600	1.72542000
C	-2.95724300	-0.33401900	-1.01623900
C	-3.52527300	-1.56858000	-1.53266700
C	4.98499200	0.03844800	4.05210300
C	3.70709100	0.43188600	4.37849100
C	2.81732800	0.92615800	3.39201100
C	3.24382500	1.01684800	2.02725000
C	4.56000200	0.56986200	1.72289600
C	5.40778300	0.10791500	2.70586000
H	1.16523200	1.25140300	4.75432100
H	5.65980900	-0.33226900	4.81721700
H	3.35692100	0.37403100	5.40580600
C	1.49732800	1.33161500	3.72268100
C	2.35314600	1.53411100	1.01996600
H	6.40801000	-0.21974100	2.43825400
C	1.08916800	1.96825200	1.41319300
C	0.66610100	1.84098400	2.76554900
H	-0.33314500	2.18862400	2.99608800
C	-3.26567400	1.72762500	0.40253900
C	-3.06990700	2.90737300	-0.37014700
C	-3.09534600	1.79392100	1.78241800
C	-3.20296200	2.91044300	-1.78613100
C	-2.74480200	4.14323400	0.28013700
C	-2.77174400	3.01211800	2.42653200
C	-3.01693500	4.06249100	-2.51290800
C	-2.57246800	5.31847700	-0.49922500
C	-2.60406800	4.15789900	1.68616000
H	-2.66095400	3.04831100	3.50310600
C	-2.69997000	5.28340400	-1.86752500
H	-3.11562600	4.03923400	-3.59418400
H	-2.32861400	6.24683200	0.01058900
H	-2.35816600	5.09265200	2.18183500
H	-2.56061900	6.18576200	-2.45488300
C	2.74819900	1.46497400	-0.41226500
C	3.82809800	2.21876500	-0.94027400
C	2.00419100	0.61099600	-1.24541100
C	4.54410900	3.17196800	-0.15666400
C	4.22284900	2.05923100	-2.31545100
C	2.38069500	0.49329600	-2.63167600
C	5.59587300	3.87973400	-0.68501400
H	4.24185100	3.32661500	0.87208300
C	5.32539100	2.80198900	-2.82578200
C	3.47925500	1.18891800	-3.12590300
C	6.00484600	3.68978700	-2.03145700
H	6.12263500	4.59876200	-0.06469400
H	5.60974200	2.65636300	-3.86468700
H	3.75482700	1.07481700	-4.17225100
H	6.84228000	4.25498700	-2.42752800

C	-2.78189400	-2.38944300	-2.45284100
N	-1.50260000	-2.33228800	-2.57042200
N	0.53659000	-0.94709900	-3.28253700
C	-0.81528000	-2.91130400	-3.70918100
C	-0.10100100	-1.71569200	-4.35501300
O	-1.73320500	0.03824100	-1.33860600
O	0.98381300	-0.04324600	-0.76097900
C	1.63011600	-0.31702700	-3.55091100
Al	-0.31284700	-1.15867300	-1.38801000
H	-3.35500000	-3.05637400	-3.10785600
H	2.03530400	-0.37588300	-4.56787300
H	-0.85178300	-1.07929200	-4.84025400
H	-1.50217700	-3.39178200	-4.41597400
H	-0.07616900	-3.63133700	-3.35227800
H	-3.44649500	1.98045800	-2.28448200
H	4.89336000	0.58386400	0.69570400
C	0.43447700	3.20377800	-0.58734900
H	0.40357700	2.51600500	-1.43336400
C	-3.12754900	0.62516900	3.87678800
H	-2.09915100	0.88115200	4.16086900
H	0.62534200	-2.03666600	-5.11051800
O	0.13423900	2.53003000	0.63235500
O	-3.29719000	0.62990800	2.47147100
H	-3.34096900	-0.39508600	4.19573300
H	-3.82372300	1.31213500	4.37423200
H	1.40992300	3.69462000	-0.54043100
H	-0.34831200	3.95013300	-0.70943100
C	2.35869400	-2.50897400	0.89223700
C	3.41150700	-2.79021100	-0.17186500
O	3.73021600	-3.88046700	-0.58826200
O	3.99804900	-1.63508000	-0.54551100
C	5.04229300	-1.75301600	-1.52015000
H	4.65662900	-2.21196000	-2.43338600
H	5.37477400	-0.73462200	-1.71633000
H	5.86127800	-2.36520600	-1.13452200
O	1.32066800	-3.50520900	0.81082100
H	1.92694000	-1.52481000	0.70959900
C	2.93740500	-2.59387400	2.30472700
H	3.71979200	-1.83739200	2.38246300
H	2.14367500	-2.29418000	2.99677200
C	0.54505600	-3.49413400	-0.27815300
C	-0.42676900	-4.65896900	-0.26272400
C	-0.96807900	-1.54455900	1.48424800
C	-1.71705900	-4.52144600	0.57403600
C	-1.94476300	-2.48943500	2.17609300
C	-1.57751700	-3.97701000	2.01681100
H	0.15292700	-5.51701500	0.09670000
H	-2.41833100	-3.88809300	0.02687700
H	-1.35205300	-0.51925600	1.49513700
H	-2.94878100	-2.31752400	1.76854700
H	-0.69650500	-4.86082100	-1.30229700
H	-0.01028900	-1.53555800	2.02936600
H	-2.15880200	-5.52526900	0.60767900
H	-1.98825200	-2.23872500	3.24440500
H	-2.24133500	-4.55883500	2.66700500
H	-0.56016100	-4.14719900	2.38715200
O	-0.76938200	-1.98053900	0.16706400
O	0.89795900	-2.87764900	-1.32459200
C	3.48195000	-3.97243100	2.68095600
H	3.87825300	-3.95442600	3.70056400
H	2.69792900	-4.73223600	2.63046900
H	4.28568400	-4.28279500	2.00755900

INT4

C	-8.17161100	-1.21784800	-0.09649100
C	-7.24276900	-1.99642000	-0.73946800
C	-5.87016600	-1.62379700	-0.76110800
C	-5.44310900	-0.41958000	-0.10578800
C	-6.43751500	0.35849500	0.55459700
C	-7.75620000	-0.02910500	0.55630700
H	-5.20207800	-3.33161900	-1.88876400
H	-9.21791600	-1.50575600	-0.08293400
H	-7.53844300	-2.91311600	-1.24287600
C	-4.89900800	-2.40766900	-1.40080700
C	-4.07172000	-0.03388700	-0.12997200
H	-6.13708000	1.26954100	1.05724900
H	-8.49346100	0.58436900	1.06571400
C	-3.11822500	-0.81603900	-0.78827200
C	-3.55912900	-2.04270400	-1.41652500
C	5.10966300	1.53634700	3.48801000
C	3.84176800	1.80247700	3.95386400
C	2.76725200	2.04067100	3.06093700
C	2.99163800	1.99817200	1.64767100
C	4.31330900	1.71310400	1.20379900
C	5.34048900	1.49430800	2.09555800
H	1.27719000	2.33787100	4.60467400
H	5.92456400	1.35440300	4.18141700
H	3.64137100	1.83404300	5.02163400

C	1.45451300	2.30471500	3.53303800
C	1.90081000	2.21021800	0.72936100
H	6.33600200	1.27462600	1.72134700
C	0.64220600	2.49502400	1.24782000
C	0.43269100	2.53392900	2.65629900
H	-0.57082600	2.76113000	2.99194600
C	-3.68090300	1.21073900	0.58961500
C	-3.81224200	2.48432400	-0.03038900
C	-3.30852900	1.14238100	1.92553500
C	-4.15497800	2.61674600	-1.40433900
C	-3.59421800	3.67753500	0.73301500
C	-3.11969100	2.32089400	2.68907600
C	-4.26077100	3.85630300	-1.99085800
C	-3.71672800	4.94121800	0.09837800
C	-3.26303900	3.55506200	2.10185100
H	-2.86846600	2.25307800	3.74011400
C	-4.03964000	5.03436100	-1.23541000
H	-4.51479100	3.93530600	-3.04373000
H	-3.54285100	5.83687600	0.68875800
H	-3.11357700	4.45596100	2.69009900
H	-4.12602300	6.00597700	-1.71181300
C	2.15507100	1.97493200	-0.71771200
C	2.97659500	2.84381400	-1.48119300
C	1.61227300	0.81985100	-1.30308000
C	3.47213300	4.07116800	-0.94976100
C	3.32725100	2.51589900	-2.83884200
C	2.00286900	0.48093000	-2.64821000
C	4.26780200	4.90005800	-1.70186000
H	3.20566100	4.33751500	0.06621000
C	4.16144500	3.39639100	-3.58594500
C	2.84098100	1.31802500	-3.37967900
C	4.62617000	4.56233700	-3.03421500
H	4.62936500	5.82979100	-1.27288500
H	4.41725900	3.12419100	-4.60656400
H	3.12602300	1.03589700	-4.39091600
H	5.25961900	5.22986900	-3.60926100
C	-2.63801500	-2.93318300	-2.07417600
N	-1.36234300	-2.78255900	-2.05959100
N	0.77510300	-1.61653200	-2.73104000
C	-0.46983900	-3.70163700	-2.73827500
C	0.52220600	-2.83667800	-3.51804100
O	-1.85724100	-0.41922600	-0.84925200
O	0.77950100	0.06345400	-0.63822000
C	1.59410400	-0.75424200	-3.24406900
Al	-0.31524300	-1.34469600	-1.03681700
H	-3.07264400	-3.79084600	-2.60082900
H	2.03045300	-0.97576200	-4.22397300
H	0.10038900	-2.55666300	-4.49096100
H	-1.00119800	-4.40090900	-3.39452600
H	0.08514000	-4.26205700	-1.98200700
H	-4.32070700	1.71603800	-1.98462100
H	4.50757300	1.64740200	0.14364400
C	-0.50146800	3.15692500	-0.80683300
H	-0.48109000	2.28707200	-1.46515600
C	-2.62724000	-0.23611200	3.75711300
H	-1.63369300	0.22550900	3.80812600
H	1.45702400	-3.38126100	-3.67088400
O	-0.49600500	2.76265900	0.56159900
O	-3.18998300	-0.10880700	2.46051100
H	-2.53536900	-1.30759500	3.93591200
H	-3.27537500	0.20112900	4.52720600
H	0.33723400	3.82046200	-1.03344700
H	-1.43980400	3.69086400	-0.95010000
C	3.10328800	-2.19467300	0.83105500
C	4.27854400	-2.07961800	-0.12513900
O	5.16929600	-2.88633100	-0.26209000
O	4.23166300	-0.89074100	-0.76487700
C	5.35188400	-0.60561000	-1.61314700
H	5.42894500	-1.35265400	-2.40686300
H	5.16037300	0.38142100	-2.03092800
H	6.27966200	-0.61064600	-1.03571000
O	2.76162100	-3.57808900	1.03217600
H	2.23699400	-1.68678900	0.40823400
C	3.43170800	-1.61496900	2.20836500
H	3.54006300	-0.53712600	2.09181300
H	2.54930000	-1.77794100	2.83521800
C	2.25850200	-4.23223400	-0.02851100
C	1.64786100	-5.55289300	0.37793500
C	-0.42998100	-2.22366200	1.61176900
C	0.19178900	-5.38752600	0.87279300
C	-0.96015900	-3.51224600	2.24329100
C	0.04858200	-4.67300100	2.23976800
H	2.26024600	-6.00662800	1.16337900
H	-0.36937700	-4.83370400	0.11812800
H	-1.21684600	-1.45798000	1.65399000
H	-1.88061200	-3.81452600	1.72561500
H	1.67278100	-6.20282400	-0.50075000
H	0.40880300	-1.85058400	2.22292500

H	-0.24770400	-6.39004100	0.92822600
H	-1.24567900	-3.28524800	3.27995800
H	-0.24964400	-5.40969200	2.99484900
H	1.02150500	-4.28649300	2.55906900
O	0.00414400	-2.44291400	0.28933400
O	2.28357800	-3.79669400	-1.16557300
C	4.67290100	-2.20704800	2.87457400
H	4.80667900	-1.77180300	3.86891000
H	4.58692100	-3.29228400	2.97682700
H	5.57594200	-1.99942900	2.29379300

INT5

C	-8.58791700	-2.10179100	-0.42904900
C	-7.54451500	-2.95809200	-0.67153500
C	-6.19744800	-2.50280800	-0.61004000
C	-5.91887800	-1.12562100	-0.30541200
C	-7.03097200	-0.27388700	-0.04071800
C	-8.31923200	-0.74743500	-0.10249900
H	-5.31263400	-4.41766800	-1.04051900
H	-9.61322900	-2.45450500	-0.47576700
H	-7.72656400	-4.00290900	-0.90906800
C	-5.11621800	-3.37053600	-0.82033200
C	-4.57457200	-0.65627400	-0.26621400
H	-6.84466200	0.76137200	0.21524800
H	-9.14633400	-0.07517800	0.10553600
C	-3.50728700	-1.54177400	-0.46191700
C	-3.80123000	-2.93071400	-0.73028000
C	2.48352300	0.83058400	4.81364700
C	1.53258600	1.82544100	4.78541800
C	1.00723900	2.30191000	3.55676900
C	1.45776100	1.72340600	2.32834500
C	2.45363700	0.71204300	2.39374800
C	2.95429200	0.27758200	3.60050400
H	-0.26898700	3.81875200	4.43152900
H	2.87756000	0.47540400	5.76060100
H	1.17127600	2.26892900	5.70945400
C	0.05974300	3.35908000	3.50361700
C	0.92069700	2.17819500	1.07868100
H	3.71131700	-0.50075300	3.61687700
C	-0.03558800	3.18356100	1.08281900
C	-0.43824000	3.79153100	2.30305800
H	-1.16485100	4.59393900	2.24318900
C	-4.28781500	0.77293800	0.02786300
C	-4.64998800	1.80773100	-0.88719100
C	-3.66217900	1.11459700	1.22179900
C	-5.21644200	1.52887000	-2.16271700
C	-4.41803600	3.17949500	-0.54004700
C	-3.42639700	2.47057400	1.55808000
C	-5.54372800	2.54318600	-3.03225100
C	-4.77415200	4.20240400	-1.45784900
C	-3.80708100	3.47373300	0.70232900
H	-2.93962600	2.71279600	2.49281500
C	-5.32727600	3.89695500	-2.67915200
H	-5.96990600	2.30364200	-4.00199300
H	-4.59102600	5.23593600	-1.17603300
H	-3.61917600	4.51112800	0.96247600
H	-5.59307500	4.68721300	-3.37439800
C	1.45777800	1.59668600	-0.17850000
C	2.34353900	2.34113400	-1.00051500
C	1.08821300	0.28931400	-0.52461600
C	2.70333100	3.68881800	-0.70566900
C	2.90626200	1.74160800	-2.18202000
C	1.70655900	-0.32026100	-1.67593500
C	3.55272000	4.38931600	-1.52761400
H	2.29232400	4.15400000	0.18160900
C	3.79005800	2.49629200	-3.00338700
C	2.58873100	0.40637300	-2.47059500
C	4.10645600	3.79297700	-2.69106600
H	3.80891900	5.41554500	-1.28211500
H	4.20644200	2.01776500	-3.88433100
H	3.06641400	-0.08090600	-3.31564500
H	4.77851900	4.36428900	-3.32306100
C	-2.75259500	-3.89788300	-0.92831000
N	-1.50831600	-3.64696100	-0.72542700
N	0.63712900	-2.48059500	-1.40514600
C	-0.45791100	-4.61054800	-0.98541700
C	0.56671500	-3.87822100	-1.86448300
O	-2.26412600	-1.09264500	-0.43281400
O	0.21665300	-0.35977100	0.20354100
C	1.48137100	-1.69940700	-2.00155700
Al	-0.71886300	-1.91393900	-0.00355100
H	-3.05420600	-4.89589900	-1.26711400
H	2.09461400	-2.10484600	-2.80874100
H	0.23631000	-3.90086200	-2.90984100
H	-0.82318200	-5.52176500	-1.47381500
H	-0.00905700	-4.87537800	-0.02250900
H	-5.37921900	0.49555700	-2.44510900
H	2.81552900	0.27636700	1.47157100

C	-1.04771300	2.82492100	-1.08349900
H	-1.89352800	3.32074200	-1.55905000
C	-2.20643900	0.28981300	2.91836900
H	-2.41118600	1.01091100	3.71871100
H	1.55516600	-4.34551100	-1.80481400
O	-0.62479400	3.70576500	-0.03025500
O	-3.33507500	0.09942900	2.06684200
H	-1.33485900	0.61447400	2.34268600
H	-2.00655100	-0.68488900	3.35956400
H	-1.37279000	1.86135700	-0.68243600
H	-0.25119800	2.67401700	-1.81536500
C	5.31900800	-0.38312800	-0.25141500
C	6.26610100	-0.60683800	-1.42551400
O	7.20712000	-1.36636800	-1.41512000
O	5.95582600	0.20321900	-2.45582400
C	6.84487700	0.12202200	-3.58151500
H	6.80734300	-0.87668100	-4.02232300
H	6.49059800	0.87018200	-4.28966200
H	7.87139800	0.33637100	-3.27650200
O	5.15478900	-1.62728500	0.45462200
H	4.35270700	-0.04457000	-0.62687600
C	5.90417200	0.60558400	0.75812300
H	5.23872500	0.61216700	1.62683500
H	6.87099100	0.21277500	1.09106700
C	4.61061700	-2.64339700	-0.24698300
C	4.52816300	-3.89850600	0.58589500
C	0.20079000	-2.23205500	2.66509400
C	3.74728400	-3.68655000	1.89373600
C	1.47399700	-3.04960500	2.89115600
C	2.36367600	-3.08495900	1.65024100
H	5.55203900	-4.22611700	0.79926900
H	3.65422000	-4.65408100	2.40152900
H	-0.42872800	-2.30758100	3.56542900
H	1.21205600	-4.07623500	3.18002400
H	4.05063700	-4.66008900	-0.03573500
H	0.48288700	-1.17716200	2.55506300
H	4.32476400	-3.03508600	2.55853500
H	2.02211500	-2.60830400	3.73453900
H	2.47203000	-2.06599800	1.25861800
H	1.85293300	-3.65321300	0.86568400
O	-0.52283100	-2.68770900	1.54968600
O	4.23431800	-2.53133200	-1.39849600
C	6.04699200	2.02400900	0.20479200
H	6.45416900	2.68579100	0.97409900
H	6.71569800	2.06130200	-0.65945200
H	5.08115500	2.42680000	-0.10718700

***re*-enantioface: *R,R*-Al-*S*-M2B + CL**

INT1

C	7.84929400	1.58841000	-0.31568000
C	6.88908100	2.29049900	-0.99916500
C	5.53003900	1.86952000	-0.98496400
C	5.15323400	0.69188100	-0.25199200
C	6.17949300	-0.00640300	0.44779200
C	7.48223500	0.42960500	0.41619900
H	4.79433100	3.47487100	-2.21868100
H	8.88423800	1.91475100	-0.32878200
H	7.14864900	3.18348000	-1.56173200
C	4.52646100	2.57814500	-1.66362600
C	3.79876100	0.25504600	-0.23975400
H	5.91230700	-0.89255600	1.01046900
H	8.24426200	-0.12018100	0.96051700
C	2.80756300	0.98723300	-0.90233500
C	3.19638000	2.17981200	-1.61936900
C	-4.31117800	-0.83054800	3.82585500
C	-3.26709300	-1.66941100	4.14379900
C	-2.41262300	-2.18354500	3.13530200
C	-2.63907200	-1.82570900	1.76769700
C	-3.72250000	-0.95280300	1.47418300
C	-4.53363000	-0.47058400	2.47739700
H	-1.18315200	-3.36414700	4.47233000
H	-4.96059500	-0.44496300	4.60605900
H	-3.08162400	-1.95586600	5.17582900
C	-1.35320100	-3.07915200	3.43746500
C	-1.80978100	-2.37342600	0.73240000
H	-5.35502000	0.18740100	2.21612100
C	-0.77604900	-3.23135900	1.08087600
C	-0.56163000	-3.58489400	2.44166200
H	0.24585900	-4.27549100	2.65206300
C	3.44540700	-1.01815700	0.44644500
C	3.77361600	-2.26341900	-0.16794500
C	2.83558700	-1.01790400	1.69559600
C	4.34538300	-2.33672300	-1.46912700
C	3.51430600	-3.49151000	0.52223600
C	2.56401500	-2.23519200	2.36955000
C	4.63903100	-3.54963700	-2.04754000
C	3.83542300	-4.72619800	-0.09936200

C	2.90636500	-3.43493100	1.79621000
H	2.07076300	-2.22598200	3.33146500
C	4.38556800	-4.76094400	-1.35885800
H	5.06705700	-3.58025200	-3.04516900
H	3.62175900	-5.64629400	0.43750600
H	2.69458800	-4.36384800	2.31681000
H	4.62072400	-5.71053000	-1.82931200
C	-2.18871300	-2.10208600	-0.68194700
C	-3.02276900	-3.01788400	-1.37415300
C	-1.76491700	-0.91982300	-1.29952600
C	-3.46021900	-4.23917700	-0.78347500
C	-3.45308000	-2.73477100	-2.71915100
C	-2.23141800	-0.62924000	-2.63118800
C	-4.25972800	-5.11394200	-1.47729500
H	-3.14815900	-4.46555700	0.22880800
C	-4.28374700	-3.66546700	-3.40633200
C	-3.05258500	-1.52882200	-3.30581200
C	-4.68008800	-4.83134300	-2.80431100
H	-4.57816200	-6.03817500	-1.00420200
H	-4.59773100	-3.42788900	-4.41928700
H	-3.40274900	-1.28241200	-4.30555500
H	-5.31310400	-5.53710700	-3.33251700
C	2.22466800	3.00938100	-2.28280600
N	0.95653000	2.81683300	-2.21363000
N	-1.14817400	1.54020500	-2.80199600
C	0.00372600	3.67643200	-2.88669000
C	-0.95474000	2.73906700	-3.63223000
O	1.55960300	0.56245800	-0.89034100
O	-0.95898100	-0.10125600	-0.67324700
C	-1.92694800	0.61078200	-3.26889200
Al	-0.02904300	1.38971100	-1.12179100
H	2.61064800	3.85763500	-2.86046600
H	-2.40196900	0.77364200	-4.24147200
H	-0.51529000	2.45166700	-4.59537200
H	0.47916600	4.39036400	-3.56984700
H	-0.53978000	4.22787400	-2.11277500
H	4.53656400	-1.41547300	-2.00663500
H	-3.91362600	-0.66253400	0.44774600
C	0.53357100	-3.18002300	-0.95897300
H	-0.18091400	-3.26579600	-1.78100700
C	2.02327300	0.23081900	3.57144400
H	2.72149200	-0.23578600	4.27689200
H	-1.91196000	3.23484900	-3.82761900
O	0.06510000	-3.86187200	0.21205600

O	2.53735900	0.19692100	2.24196100
H	1.05054000	-0.26881700	3.63555600
H	1.89939800	1.28506700	3.81188900
H	1.46286700	-3.67871300	-1.23276700
H	0.73178900	-2.12656300	-0.74547000
C	-0.56654600	2.71744200	1.38391400
C	0.80134300	3.10493000	1.95226700
O	0.98166600	3.37517500	3.12716600
O	1.78536000	3.11569200	1.03802700
C	3.10668100	3.36336700	1.55450200
H	3.49313100	2.44419300	1.99702400
H	3.71287500	3.64220900	0.69544700
H	3.08103900	4.16020800	2.29869400
O	-0.58803000	2.62647100	-0.00380200
H	-1.22450900	3.54510400	1.69651700
C	-1.08667400	1.43196400	2.06690700
H	-1.97387700	1.13543500	1.50259100
H	-0.36140900	0.62671200	1.90819900
C	-4.67343100	2.10363200	-0.98184800
C	-4.44999800	3.52222000	-1.47256500
C	-5.47335300	3.00105600	1.15104100
C	-3.32118700	4.26623600	-0.73047700
C	-4.24442900	3.76927200	1.62266600
C	-3.73774000	4.82374400	0.63377800
H	-5.38548300	4.09311900	-1.40654200
H	-2.45673800	3.60336800	-0.61300900
H	-5.95683000	2.50693600	1.99537000
H	-3.46255800	3.03886500	1.85261200
H	-4.20532600	3.41921100	-2.53127400
H	-6.21100800	3.66487300	0.68293800
H	-3.00023200	5.10217400	-1.36371200
H	-4.50232500	4.26146700	2.56812400
H	-2.88408900	5.34804300	1.07751500
H	-4.52520400	5.57802400	0.49169400
O	-5.16414600	1.90820700	0.26367500
O	-4.42605700	1.12933500	-1.65898700
C	-1.43883800	1.57003300	3.54685100
H	-1.83284200	0.62518500	3.92665100
H	-0.57644700	1.86836800	4.14445100
H	-2.21285600	2.33229800	3.69604200

TS1

C	-7.51642000	-1.15333300	0.37991600
C	-6.83733700	-0.26864100	1.17820700

C	-5.41494900	-0.25938800	1.21234800
C	-4.66873000	-1.18520700	0.40483900
C	-5.41402400	-2.09573800	-0.40098300
C	-6.78760200	-2.07729700	-0.41339600
H	-5.24828500	1.34510600	2.63795200
H	-8.60139400	-1.15462900	0.35490300
H	-7.37365300	0.44536500	1.79805500
C	-4.70049800	0.64279000	2.01257700
C	-3.24610900	-1.18027000	0.42639700
H	-4.87506300	-2.81622800	-1.00384000
H	-7.32642700	-2.78443000	-1.03731900
C	-2.54051500	-0.26601700	1.22697500
C	-3.31260200	0.67246500	2.01631700
C	4.08690600	1.97296000	-3.80406000
C	3.63021800	0.75032800	-4.24164900
C	3.29331000	-0.27424900	-3.32204700
C	3.42560600	-0.03374900	-1.91767500
C	3.89629100	1.23961200	-1.49839900
C	4.22145200	2.21288800	-2.41714400
H	2.71496400	-1.72996200	-4.81755900
H	4.34556300	2.74940500	-4.51762800
H	3.51753300	0.55040600	-5.30397700
C	2.81823600	-1.54077300	-3.75264600
C	3.10645600	-1.06480500	-0.97263900
H	4.59706600	3.17199000	-2.07176500
C	2.59899900	-2.26954200	-1.44392300
C	2.46317900	-2.49792100	-2.84175300
H	2.07096800	-3.46155900	-3.14766900
C	-2.51308100	-2.14377200	-0.44147800
C	-1.92533100	-3.32568900	0.09564600
C	-2.48691300	-1.94209200	-1.81734100
C	-1.96531600	-3.61960100	1.48632600
C	-1.31377900	-4.28064200	-0.78051500
C	-1.84396400	-2.86331700	-2.68046200
C	-1.45902600	-4.80149900	1.97469800
C	-0.79833400	-5.48969600	-0.24432000
C	-1.26866600	-4.00081600	-2.16676800
H	-1.80422200	-2.67229300	-3.74502400
C	-0.87594800	-5.75402900	1.10302400
H	-1.50743500	-5.00966500	3.03944900
H	-0.33882900	-6.20296000	-0.92283500
H	-0.78245200	-4.70993800	-2.83041000
H	-0.48283900	-6.68365400	1.50266500
C	3.47277700	-0.85756200	0.45728700
C	4.76230800	-1.26137800	0.89592800
C	2.56279000	-0.26708500	1.34600500
C	5.71530500	-1.84992400	0.01353800
C	5.14763100	-1.08737200	2.27068800
C	2.94980900	-0.13161500	2.72966700
C	6.95692000	-2.22907900	0.46264300
H	5.44218300	-1.99165400	-1.02499200
C	6.44239900	-1.49214900	2.70081300
C	4.21349800	-0.52855000	3.15401600
C	7.33279600	-2.05099300	1.81991400
H	7.66427200	-2.67319000	-0.23156100
H	6.70882700	-1.34906200	3.74484200
H	4.48148700	-0.41228600	4.20221100
H	8.31904200	-2.35803500	2.15303700
C	-2.66509700	1.57726400	2.92235300
N	-1.40502300	1.85270000	2.91035900
N	0.82355000	0.73160700	3.52109300
C	-0.79622100	2.41870400	4.10473700
C	0.06658600	1.26995400	4.64862400
O	-1.22978600	-0.29569100	1.29246500
O	1.37559000	0.08429100	0.92863900
C	2.04436500	0.37146300	3.72485100
Al	-0.00960000	1.06485600	1.59927000
H	-3.29096800	2.00562800	3.71500000
H	2.45282100	0.44113200	4.74031200
H	-0.60038400	0.48903800	5.03626900
H	-1.54202300	2.73745400	4.84319200
H	-0.15737000	3.25821300	3.82654600
H	-2.41829700	-2.89651400	2.15435600
H	3.98735200	1.43840700	-0.43763200
C	1.68265300	-3.17384500	0.61393700
H	2.45825900	-3.03994100	1.37149800
C	-3.41923100	-0.71801500	-3.66098800
H	-4.08491200	-1.53596500	-3.96278000
H	0.72248000	1.60329600	5.46201600
O	2.25277500	-3.34897300	-0.69023400
O	-3.09785500	-0.80867300	-2.27882000
H	-2.52400000	-0.72086700	-4.28920500
H	-3.91575600	0.24351000	-3.78166600
H	1.13551900	-4.09239600	0.81381500
H	0.99524900	-2.32529600	0.62897900
C	-0.67630300	1.90000000	-1.25703100
C	-2.11469900	2.11086500	-1.72633500
O	-2.41975300	2.31144200	-2.88851200

O	-3.01316400	2.05453600	-0.72815600
C	-4.38988900	2.10436700	-1.12912400
H	-4.68550100	1.12869400	-1.51951100
H	-4.95385300	2.32261500	-0.22422600
H	-4.54272100	2.87360200	-1.88805500
O	-0.53027300	2.11931400	0.11979900
H	-0.08877900	2.66036800	-1.78652100
C	-0.13553100	0.53978500	-1.73283200
H	0.85078900	0.43869700	-1.28448300
H	-0.74610900	-0.25604600	-1.30873700
C	0.70343800	3.48380900	0.79687100
C	-0.26415500	4.62780200	1.04738800
C	1.87871000	4.76122700	-0.94097400
C	-1.11138500	5.17054500	-0.10670600
C	0.77266300	5.27433400	-1.85418200
C	-0.35397100	6.01470100	-1.13249800
H	0.33484700	5.43827600	1.49183700
H	-1.62194000	4.33523200	-0.58836300
H	2.74507000	4.47215400	-1.53606200
H	0.37710000	4.44501100	-2.44957300
H	-0.92516000	4.26044600	1.83233200
H	2.19087800	5.54412800	-0.23758700
H	-1.89967300	5.79108500	0.33523400
H	1.25245500	5.95934900	-2.56494200
H	-1.06353700	6.39792600	-1.87432400
H	0.07065800	6.89446200	-0.62743700
O	1.60521500	3.55529900	-0.19529400
O	1.02333700	2.74407600	1.78656400
C	-0.02967100	0.42079500	-3.25175200
H	0.26049800	-0.59623800	-3.52418800
H	-0.96727700	0.67141300	-3.74780700
H	0.73935400	1.09613500	-3.64055000

INT2

C	-7.64195200	-0.72234400	0.47823300
C	-6.91914700	0.16283100	1.23684900
C	-5.49736200	0.11502100	1.25698800
C	-4.79948800	-0.86845300	0.47546000
C	-5.58881200	-1.77749000	-0.28828600
C	-6.96071000	-1.70386000	-0.28745700
H	-5.24736100	1.74955900	2.63687900
H	-8.72628800	-0.68034100	0.46412800
H	-7.41958800	0.91928000	1.83586700
C	-4.73685800	1.00914400	2.02411900
C	-3.37733600	-0.91779500	0.47981800
H	-5.08527500	-2.54036900	-0.86933600
H	-7.53497400	-2.41073000	-0.87914900
C	-2.62669700	-0.00258300	1.23486600
C	-3.34912100	0.97850100	2.01545900
C	3.89688900	1.14632500	-4.10564200
C	3.38829100	-0.10991500	-4.34493600
C	3.04714100	-0.97409700	-3.27482300
C	3.24455500	-0.54025800	-1.92548600
C	3.77237600	0.76170300	-1.71362200
C	4.08481100	1.58220100	-2.77427300
H	2.33887100	-2.59560700	-4.52339900
H	4.15019500	1.80275600	-4.93246000
H	3.22913300	-0.45768300	-5.36226300
C	2.49579100	-2.26224400	-3.50118700
C	2.92278500	-1.40991300	-0.82928100
H	4.48013900	2.57634600	-2.58855900
C	2.35436700	-2.64758000	-1.10524300
C	2.14111100	-3.06188200	-2.44954200
H	1.69847100	-4.04031000	-2.59840900
C	-2.68900000	-1.94693700	-0.34666400
C	-2.14545400	-3.12473000	0.24427800
C	-2.65848400	-1.80748600	-1.72869600
C	-2.22231400	-3.36799200	1.64314400
C	-1.55347600	-4.13041500	-0.58697300
C	-2.04917300	-2.78732300	-2.55041900
C	-1.77059000	-4.55055900	2.18085600
C	-1.07926700	-5.33146100	0.00182600
C	-1.50163300	-3.91373500	-1.98489500
H	-2.00784600	-2.64625600	-3.62273900
C	-1.19639800	-5.54796100	1.35471200
H	-1.85240200	-4.72290300	3.24996400
H	-0.62715800	-6.08096400	-0.64138800
H	-1.03717400	-4.66567100	-2.61605000
H	-0.84112100	-6.47509900	1.79364700
C	3.35836900	-1.01711800	0.54260300
C	4.66899700	-1.37015600	0.96441400
C	2.49925100	-0.30502200	1.39233800
C	5.57444600	-2.07556500	0.11837000
C	5.12659400	-1.02108800	2.28273000
C	2.95617100	0.00097000	2.72856700
C	6.83807200	-2.40087000	0.54778900
H	5.24751800	-2.34999300	-0.87700000
C	6.44248800	-1.37490500	2.69411900

C	4.24049600	-0.34824900	3.13463000
C	7.28528700	-2.04953400	1.84852400
H	7.50785900	-2.93651600	-0.11846400
H	6.76327900	-1.09855300	3.69516200
H	4.56259000	-0.10261400	4.14450300
H	8.28788400	-2.31681800	2.16663400
C	-2.65057800	1.88274900	2.88568200
N	-1.37483100	2.05044400	2.87492400
N	0.87208900	0.96456100	3.52496300
C	-0.71711700	2.72538200	3.98208500
C	0.16260000	1.63430900	4.61392100
O	-1.31378800	-0.06995800	1.25776800
O	1.29089200	0.00327200	0.99747800
C	2.10306300	0.62095100	3.70441600
Al	0.02028200	1.14099700	1.64223900
H	-3.25412500	2.41609700	3.63018900
H	2.56109900	0.80099000	4.68402700
H	-0.48851000	0.90569900	5.11388900
H	-1.43093000	3.12808500	4.71103700
H	-0.08823300	3.52172600	3.58034800
H	-2.66628400	-2.60889900	2.27660900
H	3.90365700	1.10930800	-0.69631300
C	1.50282700	-3.23843100	1.09086000
H	2.31071600	-3.03035600	1.79661200
C	-3.62006300	-0.65693000	-3.60445400
H	-4.33476200	-1.46281700	-3.81071100
H	0.85367300	2.05045200	5.35645700
O	2.01557500	-3.60351100	-0.19579400
O	-3.22300300	-0.66923800	-2.23870800
H	-2.76470200	-0.74188100	-4.28071100
H	-4.08423900	0.31505800	-3.76428700
H	0.93641800	-4.10343600	1.42951000
H	0.84036800	-2.37363100	1.01509200
C	-0.55085200	1.89231200	-1.53572800
C	-1.99660900	2.18737600	-1.90233400
O	-2.37137200	2.30473300	-3.05321400
O	-2.80749200	2.28586800	-0.83760300
C	-4.20808100	2.39998400	-1.13958700
H	-4.58443700	1.42728200	-1.46079500
H	-4.68596900	2.69169400	-0.20676900
H	-4.37293800	3.14376300	-1.92080300
O	-0.31694200	2.13632900	-0.14038500
H	0.08113300	2.57616400	-2.10302100
C	-0.13995800	0.45799600	-1.88366100
H	0.85009000	0.32194900	-1.45168100
H	-0.79977100	-0.23556500	-1.36452000
C	0.59498200	3.20529000	0.30142800
C	-0.13130100	4.56243200	0.28275400
C	2.65390800	4.15246900	-0.50800300
C	-0.13085600	5.35057700	-1.03476200
C	2.40652500	5.22091800	-1.56846300
C	1.18175300	6.10410000	-1.30191100
H	0.32494100	5.17390500	1.06929500
H	-0.37021000	4.69778000	-1.88134100
H	3.61957800	3.66800200	-0.67667000
H	2.29708600	4.70382100	-2.52985500
H	-1.15410600	4.34935600	0.60615400
H	2.67368300	4.57299700	0.50505200
H	-0.94140400	6.08750200	-0.98771700
H	3.29917600	5.85520900	-1.64921700
H	1.04127700	6.77861600	-2.15499900
H	1.38886600	6.74702100	-0.43435900
O	1.67701500	3.11764700	-0.59723000
O	0.88233300	2.79421700	1.54733800
C	-0.10366900	0.19223700	-3.38636800
H	0.09372400	-0.86627100	-3.56805200
H	-1.04070900	0.47052800	-3.86955300
H	0.69930500	0.76411200	-3.86085600

INT3

C	-6.70310800	2.76188800	0.75954300
C	-6.38079700	1.66511100	0.00093900
C	-5.02941600	1.39440300	-0.35279200
C	-3.98056600	2.26885500	0.09556000
C	-4.36010200	3.40617000	0.86582200
C	-5.67529700	3.64120100	1.18717600
H	-5.46430700	-0.36670700	-1.52045100
H	-7.73540700	2.96410500	1.02676100
H	-7.15158900	0.98273200	-0.34745500
C	-4.68426200	0.29583700	-1.15607200
C	-2.62303000	1.98938500	-0.23245200
H	-3.58955400	4.09231500	1.19492400
H	-5.93388600	4.51613500	1.77648400
C	-2.28591900	0.84715800	-0.97541500
C	-3.36333300	0.01145900	-1.46559200
C	3.82347800	-1.97069500	4.28734800
C	3.43185600	-0.66299500	4.46461900
C	3.23894500	0.19877400	3.35547000

C	3.44254500	-0.29944800	2.02847500
C	3.85323000	-1.65267000	1.88359500
C	4.03862800	-2.46439600	2.98068900
H	2.73260100	1.95251500	4.52256200
H	3.96908300	-2.62110500	5.14403500
H	3.26727400	-0.26646700	5.46309900
C	2.87689000	1.56146800	3.51876200
C	3.27707800	0.56199300	0.89259400
H	4.35252900	-3.49420900	2.83984600
C	2.92494700	1.89015600	1.10645400
C	2.73190200	2.37771000	2.42984700
H	2.47111400	3.42339000	2.53186500
C	-1.56150900	2.94509600	0.19209900
C	-0.99718900	3.85632700	-0.74962500
C	-1.17306000	3.03016600	1.52370300
C	-1.33241800	3.81648100	-2.13127300
C	-0.06464500	4.85216100	-0.31197700
C	-0.24466300	4.00864600	1.95557500
C	-0.77300100	4.70158000	-3.02262900
C	0.48689600	5.75848600	-1.25570200
C	0.29159900	4.89609900	1.05480600
H	0.04785500	4.05956400	2.99573800
C	0.14632800	5.68659700	-2.58558800
H	-1.04047000	4.64834100	-4.07383600
H	1.19395300	6.50489500	-0.90411000
H	1.00763100	5.64139300	1.38808200
H	0.57736400	6.38016800	-3.30087800
C	3.58541000	0.02457600	-0.46390800
C	4.91820000	0.05129800	-0.94937200
C	2.54622400	-0.50255300	-1.24484100
C	5.99781400	0.56561500	-0.17382900
C	5.21795400	-0.44662400	-2.26583800
C	2.84936800	-0.94940200	-2.58485500
C	7.28043600	0.57333000	-0.66539100
H	5.78662400	0.94998900	0.81688300
C	6.55927600	-0.42238500	-2.74165300
C	4.16032100	-0.92454500	-3.05277900
C	7.57264600	0.07381300	-1.96198600
H	8.08599200	0.96853000	-0.05363200
H	6.76175800	-0.80506500	-3.73853300
H	4.36932000	-1.27029700	-4.06301600
H	8.59352700	0.08943000	-2.32975100
C	-3.09167100	-1.06879400	-2.38315400
N	-1.91338700	-1.55807600	-2.54142400
N	0.55293800	-1.35461500	-3.26820300
C	-1.57573500	-2.44687000	-3.63825900
C	-0.37406400	-1.77462600	-4.32144600
O	-1.02553400	0.57208900	-1.24930800
O	1.32848200	-0.52468100	-0.77326400
C	1.82193700	-1.35803400	-3.50504800
Al	-0.27959900	-1.11593200	-1.41169400
H	-3.92499200	-1.44367300	-2.98310000
H	2.17123500	-1.67093800	-4.49569000
H	-0.72479300	-0.88167600	-4.85394300
H	-2.40729400	-2.58933500	-4.33776900
H	-1.26524800	-3.41157100	-3.22727600
H	-2.04021400	3.07058400	-2.47206000
H	4.02347200	-2.03946600	0.88588900
C	2.26126200	2.58706000	-1.13590700
H	1.38676600	1.93772500	-1.05973600
C	-1.30582300	2.12092400	3.73457400
H	-0.23532000	1.88898100	3.79013100
H	0.10786500	-2.44401400	-5.04326400
O	2.80658500	2.86359000	0.16176100
O	-1.76110500	2.14885600	2.39073500
H	-1.87406300	1.32942200	4.22370100
H	-1.50213600	3.06935300	4.25009500
H	3.00942500	2.14218200	-1.79551700
H	1.96133400	3.55589400	-1.53202600
C	-2.47217700	-4.07285300	-0.25338200
C	-3.65292600	-3.17884200	0.10726200
O	-4.53600600	-2.91955800	-0.69031500
O	-3.65827400	-2.77493700	1.38273100
C	-4.74089100	-1.90573600	1.76170000
H	-4.64981200	-0.94621400	1.25033700
H	-5.70220400	-2.35881800	1.51114000
H	-4.64272100	-1.77368000	2.83840300
O	-1.35582500	-3.93419700	0.60215300
H	-2.19058400	-3.80529100	-1.27306000
C	-2.91483400	-5.54235100	-0.21737800
H	-3.83422800	-5.63417900	-0.80545800
H	-3.16191500	-5.80102100	0.81900600
C	-0.36685600	-3.00219800	0.22608100
C	0.85593000	-3.26414400	1.09961500
C	-0.79382800	-0.82218700	1.60723700
C	0.61330300	-3.56302700	2.58027700
C	-1.00748900	-1.58948200	2.90705500
C	0.22722000	-2.34059300	3.41491800

H	1.52841900	-2.41428600	0.97142000
H	-0.15977500	-4.33337700	2.66387600
H	-1.54307300	-0.04044300	1.49771700
H	-1.85807900	-2.26520000	2.78748700
H	1.34005400	-4.12299500	0.62427600
H	0.19485300	-0.35172500	1.57939400
H	1.53498800	-3.98572700	2.99514600
H	-1.29292100	-0.84366900	3.65900800
H	0.05185500	-2.66062200	4.44978300
H	1.07346300	-1.64585300	3.44807800
O	-0.94597800	-1.62145800	0.40674600
O	-0.09132400	-2.95819000	-1.08966500
C	-1.82878500	-6.47370000	-0.75304400
H	-0.90871700	-6.35511100	-0.17648500
H	-2.14445900	-7.51967000	-0.69552100
H	-1.59821200	-6.24544500	-1.79943300

TS2

C	-6.56934300	2.96853500	0.98829200
C	-6.29553500	1.84689700	0.24740800
C	-4.96820000	1.55768900	-0.17575400
C	-3.89196200	2.44162900	0.18013600
C	-4.22202600	3.60324900	0.93745400
C	-5.51491200	3.85501600	1.32808200
H	-5.47702900	-0.23063200	-1.27098800
H	-7.58368600	3.18478000	1.30833700
H	-7.08814400	1.15848200	-0.03421600
C	-4.67393000	0.43493700	-0.96536400
C	-2.55697400	2.14710400	-0.21761500
H	-3.43077100	4.29455800	1.20008600
H	-5.73534700	4.74887100	1.90441800
C	-2.26488300	0.98204000	-0.94448700
C	-3.37409500	0.13635400	-1.34384900
C	3.74798700	-2.07844900	4.31552500
C	3.41230400	-0.75585000	4.49609700
C	3.25372700	0.11548200	3.38917700
C	3.42731000	-0.39100700	2.06116000
C	3.78612100	-1.75823300	1.91226600
C	3.94316000	-2.57797500	3.00761600
H	2.83107200	1.88931300	4.55881200
H	3.86850200	-2.73580600	5.17086400
H	3.26711100	-0.35487800	5.49578500
C	2.95059700	1.49221200	3.55420700
C	3.28077600	0.47400000	0.92775000
H	4.21917200	-3.61859100	2.86537500
C	2.98195300	1.81484900	1.14147400
C	2.82601100	2.31290500	2.46542800
H	2.60820000	3.36856500	2.56827200
C	-1.46376100	3.08887700	0.15272400
C	-0.91129400	3.97542200	-0.81765800
C	-1.02253200	3.17266500	1.46883000
C	-1.29847900	3.93046900	-2.18549100
C	0.06696100	4.94673300	-0.42598600
C	-0.05205200	4.12942000	1.85515800
C	-0.74364300	4.78585400	-3.10803100
C	0.61299600	5.82255800	-1.40136300
C	0.47580500	4.99264900	0.92629300
H	0.27745200	4.18217900	2.88422700
C	0.22314300	5.74469700	-2.71732400
H	-1.05055900	4.72889600	-4.14827100
H	1.35548900	6.55045400	-1.08549000
H	1.22566800	5.71907000	1.22514800
H	0.65073800	6.41460900	-3.45690000
C	3.55303000	-0.06859100	-0.43308400
C	4.87501800	-0.04903900	-0.94856900
C	2.49102100	-0.57773400	-1.19453800
C	5.97729100	0.44222600	-0.19024400
C	5.13938400	-0.52988900	-2.27847000
C	2.75810200	-0.99526000	-2.54996100
C	7.24883800	0.44239500	-0.71002800
H	5.79263200	0.81419200	0.81045000
C	6.46970900	-0.51384300	-2.78399900
C	4.05674200	-0.97743600	-3.04963300
C	7.50608400	-0.04114200	-2.01987100
H	8.07237500	0.81921900	-0.11073700
H	6.64529600	-0.88299300	-3.79105200
H	4.23561200	-1.30081300	-4.07302600
H	8.51859500	-0.03149700	-2.41037300
C	-3.15949100	-0.95485200	-2.26349900
N	-2.00808000	-1.50184800	-2.43204500
N	0.43804800	-1.31697400	-3.18641700
C	-1.71786800	-2.34009600	-3.57975000
C	-0.51411400	-1.65738800	-4.24490400
O	-1.02483800	0.68475300	-1.28167900
O	1.28638300	-0.61760700	-0.68888600
C	1.69886000	-1.34977700	-3.45547300
Al	-0.38425500	-1.06276100	-1.28802900
H	-4.00853000	-1.27811200	-2.87127400

H	2.01585200	-1.63720600	-4.46484400
H	-0.85496100	-0.72750500	-4.71699800
H	-2.56624200	-2.41897100	-4.26903400
H	-1.42520800	-3.33613000	-3.23837000
H	-2.04132400	3.20278800	-2.48950700
H	3.94027600	-2.14772500	0.91281600
C	2.28601500	2.52338900	-1.08537300
H	1.38849500	1.91071500	-0.97549300
C	-1.05999500	2.24157400	3.67337600
H	0.00345700	1.97655500	3.65098600
H	-0.05855200	-2.29250400	-5.01379300
O	2.88897100	2.78476800	0.19057800
O	-1.60204900	2.31915000	2.36446600
H	-1.61824100	1.45270800	4.17796400
H	-1.19324800	3.18058400	4.22591100
H	2.99155400	2.04584600	-1.76848400
H	2.01070600	3.50108900	-1.47709200
C	-2.50454700	-4.26342300	-0.36002600
C	-3.62660600	-3.32054500	0.05545000
O	-4.46544300	-2.93680200	-0.73747000
O	-3.64538200	-3.05218100	1.36134200
C	-4.64884100	-2.10860200	1.78434400
H	-4.47999600	-1.14470400	1.30167600
H	-5.64660600	-2.47328400	1.53153200
H	-4.52776400	-2.02262700	2.86285700
O	-1.35211200	-4.14187300	0.47720500
H	-2.23878100	-3.99758900	-1.38190400
C	-2.98016400	-5.71735700	-0.29201300
H	-3.91346600	-5.78686600	-0.86116100
H	-3.21646700	-5.95450800	0.75139500
C	-0.33552700	-3.35606500	0.06687300
C	0.83272200	-3.39015000	1.01472900
C	-1.06986500	-0.70717200	1.51659000
C	0.53160300	-3.51303500	2.51017700
C	-1.10529400	-1.50717700	2.81779500
C	0.20669200	-2.19409500	3.21957300
H	1.43528100	-2.50807700	0.79971300
H	-0.27970200	-4.23271800	2.66268000
H	-1.94139300	-0.04474800	1.48821400
H	-1.91424800	-2.24179700	2.76348800
H	1.41760200	-4.25930800	0.67993600
H	-0.17956100	-0.06276500	1.50632000
H	1.42249300	-3.93427500	2.98722800
H	-1.37262500	-0.79765000	3.61195500
H	0.17506100	-2.40294900	4.29650200
H	1.03723600	-1.49583700	3.07314900
O	-1.09163300	-1.52928200	0.36114800
O	-0.16745300	-3.09243000	-1.16449900
C	-1.93714300	-6.69064300	-0.84009700
H	-1.00585900	-6.61695500	-0.27291600
H	-2.29505000	-7.72219800	-0.77794600
H	-1.70991600	-6.47359300	-1.88942900

INT4

C	-6.55746200	3.02499500	1.02856400
C	-6.29001300	1.89844100	0.29278200
C	-4.96649400	1.60654500	-0.13997500
C	-3.88773000	2.49341400	0.19970900
C	-4.21082000	3.65917200	0.95343000
C	-5.50021700	3.91345500	1.35392400
H	-5.48383200	-0.19043000	-1.21684200
H	-7.56903500	3.24325400	1.35597100
H	-7.08505900	1.20805400	0.02304600
C	-4.67812900	0.47769400	-0.92306600
C	-2.55629600	2.19786000	-0.20946600
H	-3.41702100	4.35168700	1.20512400
H	-5.71559700	4.81067100	1.92693300
C	-2.27047400	1.03088900	-0.93425500
C	-3.38185400	0.17825300	-1.31310300
C	3.75615600	-2.10609700	4.29982900
C	3.42465500	-0.78385300	4.49034700
C	3.26519000	0.09532900	3.38991100
C	3.43172900	-0.40350900	2.05823400
C	3.78785900	-1.77020300	1.89874800
C	3.94693800	-2.59734900	2.98809100
H	2.85141700	1.86280700	4.57207000
H	3.87741500	-2.76948800	5.15040400
H	3.28373900	-0.38930800	5.49319100
C	2.96565000	1.47161700	3.56456400
C	3.27785000	0.46792500	0.93133200
H	4.22079600	-3.63752700	2.83838300
C	2.98258700	1.80795500	1.15380700
C	2.83606600	2.29905200	2.48130900
H	2.62016600	3.35442300	2.59150200
C	-1.45881400	3.13585700	0.15734100
C	-0.90838200	4.02591900	-0.81031200
C	-1.00901800	3.20771000	1.47142100
C	-1.30484900	3.99194600	-2.17575900

C	0.07866400	4.98840600	-0.41853500
C	-0.03006800	4.15581700	1.85783300
C	-0.75046400	4.84953600	-3.09650800
C	0.62424100	5.86650300	-1.39212800
C	0.49691700	5.02226100	0.93138100
H	0.30590400	4.19928200	2.88532700
C	0.22531200	5.79937000	-2.70599900
H	-1.06438300	4.80122500	-4.13510400
H	1.37361900	6.58755500	-1.07674900
H	1.25342100	5.74182300	1.23015000
H	0.65264100	6.47102800	-3.44416000
C	3.53883000	-0.06761200	-0.43396000
C	4.85602400	-0.03915900	-0.96201700
C	2.47283500	-0.57870900	-1.18761700
C	5.96243900	0.45501800	-0.21183400
C	5.11067100	-0.51353900	-2.29599100
C	2.72971000	-0.98899300	-2.54623700
C	7.22921000	0.46388700	-0.74315100
H	5.78487800	0.82212200	0.79192000
C	6.43617700	-0.48856200	-2.81366300
C	4.02310200	-0.96362900	-3.05868300
C	7.47692500	-0.01327000	-2.05712000
H	8.05619700	0.84277100	-0.14996600
H	6.60447700	-0.85280600	-3.82373000
H	4.19333800	-1.28203600	-4.08508700
H	8.48574500	0.00332200	-2.45684300
C	-3.17741600	-0.92093900	-2.22564900
N	-2.03218600	-1.47996500	-2.39680400
N	0.40414900	-1.30816700	-3.16001400
C	-1.75878400	-2.31810900	-3.54809500
C	-0.55337000	-1.64309500	-4.21610800
O	-1.03323800	0.73481900	-1.28828800
O	1.27229500	-0.63326700	-0.67065500
C	1.66220900	-1.34312500	-3.44102500
Al	-0.41662100	-1.01650100	-1.25665600
H	-4.03075900	-1.24067200	-2.82908400
H	1.96906200	-1.63089400	-4.45338800
H	-0.88955900	-0.71069300	-4.68652900
H	-2.61213900	-2.38435200	-4.23234000
H	-1.47466400	-3.31823900	-3.21362900
H	-2.05410300	3.27044600	-2.47904100
H	3.93749800	-2.15324300	0.89612000
C	2.26647100	2.52768200	-1.06251400
H	1.36615900	1.92046300	-0.94477400
C	-1.02750300	2.24446100	3.66231900
H	0.03290500	1.97066900	3.61820100
H	-0.10286600	-2.28006700	-4.98638100
O	2.88534800	2.78181000	0.20735000
O	-1.58884600	2.35226000	2.36374600
H	-1.58478700	1.45024200	4.15948600
H	-1.14404700	3.17356800	4.23518700
H	2.96124400	2.04799700	-1.75506400
H	1.99263100	3.50806900	-1.44830600
C	-2.48301700	-4.38550700	-0.37748100
C	-3.57590900	-3.40513200	0.03043000
O	-4.39263800	-2.99002200	-0.76901600
O	-3.60116200	-3.15245900	1.33836500
C	-4.56813100	-2.16908600	1.75665800
H	-4.35219500	-1.21227200	1.27919300
H	-5.57778400	-2.49084100	1.49296300
H	-4.45345900	-2.09216700	2.83642500
O	-1.31424900	-4.25695200	0.44245400
H	-2.22631600	-4.15495000	-1.41008500
C	-2.98296900	-5.82646400	-0.25190000
H	-3.92459800	-5.89774600	-0.80714700
H	-3.21109300	-6.02130000	0.80181400
C	-0.29153800	-3.51429000	-0.00283800
C	0.86088200	-3.46135000	0.95846200
C	-1.12978100	-0.68878800	1.49571200
C	0.54705000	-3.52351600	2.45729000
C	-1.09547300	-1.50847400	2.78581900
C	0.24467200	-2.17653400	3.12717200
H	1.43155300	-2.56776600	0.70705800
H	-0.27526600	-4.22497400	2.63290700
H	-2.03122500	-0.06268200	1.50624200
H	-1.88910600	-2.26132600	2.75153500
H	1.48438600	-4.32235600	0.67352000
H	-0.27296700	0.00158800	1.48702500
H	1.42934100	-3.94228700	2.95190800
H	-1.34691000	-0.81944400	3.60360700
H	0.27839700	-2.34813500	4.21046000
H	1.06014800	-1.47931100	2.91060000
O	-1.12893100	-1.50267800	0.34372300
O	-0.18049400	-3.18244200	-1.20178600
C	-1.96691400	-6.83970500	-0.77808300
H	-1.02789500	-6.76906300	-0.22309500
H	-2.34623400	-7.86040800	-0.67723800
H	-1.74731500	-6.66359600	-1.83666900

si-enantioface: *R*,*R*-Al-*S*-M2B + L-EG

INT1

C	-8.11377200	-1.32013900	-0.71527100
C	-7.11295400	-2.21771800	-0.98672300
C	-5.74541100	-1.83934500	-0.87716700
C	-5.39964400	-0.50810000	-0.46355000
C	-6.46855000	0.39861600	-0.21218500
C	-7.77923000	0.00434200	-0.33306300
H	-4.95835300	-3.71968700	-1.57011100
H	-9.15528200	-1.61259800	-0.80118900
H	-7.34556600	-3.23313500	-1.29615100
C	-4.70845900	-2.72618000	-1.20420600
C	-4.03139000	-0.12969800	-0.32878000
H	-6.23038300	1.41712400	0.06811000
H	-8.57336000	0.71898700	-0.13800700
C	-3.00913600	-1.04587500	-0.61665700
C	-3.37337100	-2.35919200	-1.10088900
C	5.60967500	3.48848300	2.37930600
C	4.66007900	3.14726000	3.31156600
C	3.35200000	2.75989100	2.91864000
C	3.00118400	2.72981300	1.52808900
C	4.01791300	3.08107800	0.59268400
C	5.27826300	3.44744100	1.00502800
H	2.64272400	2.41527900	4.93094000
H	6.60776200	3.78042800	2.69000600
H	4.89556000	3.16173500	4.37234700
C	2.38092200	2.38599900	3.87702600
C	1.68178600	2.32280700	1.13098600
H	6.02819300	3.70528000	0.26314900
C	0.77268800	1.95074900	2.11765700
C	1.12490700	1.99177300	3.49176400
H	0.39450500	1.71543900	4.24125600
C	-3.67875300	1.24230400	0.12962600
C	-3.02274400	2.17002300	-0.73250600
C	-3.99813800	1.63651000	1.42402000
C	-2.69453200	1.85283100	-2.07864900
C	-2.69283100	3.47736800	-0.24901100
C	-3.69373100	2.93450300	1.89693400
C	-2.07747800	2.77372500	-2.89125000
C	-2.04693500	4.40056100	-1.11213100
C	-3.04342800	3.82456600	1.07559400
H	-3.94857200	3.22278000	2.90890000
C	-1.74857800	4.06306200	-2.40875800
H	-1.83347700	2.50937100	-3.91572200
H	-1.78768800	5.38155500	-0.72519400
H	-2.79461900	4.81638600	1.44183900
H	-1.24544500	4.77214600	-3.05557800
C	1.34863300	2.23317600	-0.31747500
C	1.42919600	3.38633000	-1.14887600
C	1.06159100	0.98680800	-0.89701400
C	1.51607000	4.70030900	-0.60441200
C	1.42653100	3.26104900	-2.58160300
C	1.08714200	0.86632300	-2.33820100
C	1.61045700	5.80280500	-1.41761400
H	1.49306500	4.81511900	0.47245800
C	1.55226900	4.42332400	-3.39467100
C	1.29752900	1.98398100	-3.14016600
C	1.64233800	5.67011100	-2.83117000
H	1.66322500	6.79269300	-0.97425200
H	1.56108900	4.29927800	-4.47426600
H	1.34433400	1.86601900	-4.22004800
H	1.73105000	6.55299900	-3.45589400
C	-2.38322900	-3.26332300	-1.63203600
N	-1.11754100	-3.05977800	-1.55162000
N	0.66057500	-1.52306500	-2.39855900
C	-0.15384700	-3.81014200	-2.33621800
C	0.57492600	-2.75682900	-3.19734200
O	-1.74763500	-0.68212600	-0.47669200
O	0.87432100	-0.08566600	-0.16007100
C	0.98269100	-0.41161000	-2.98535200
Al	-0.16602800	-1.53474900	-0.57485200
H	-2.75624900	-4.13218900	-2.18690500
H	1.19215300	-0.42640600	-4.05823000
H	0.00392600	-2.56544200	-4.11346100
H	-0.61963800	-4.58064900	-2.96101800
H	0.54675500	-4.28638900	-1.64611000
H	-2.94909600	0.87103500	-2.45934200
H	3.79647400	3.04875900	-0.46483700
C	-1.22635300	0.76119700	2.60912500
H	-0.60134900	0.01931900	3.11366400
C	-5.03381500	1.04633000	3.50802800
H	-5.50625600	0.15487000	3.92226600
H	1.57725400	-3.09588300	-3.47233600
O	-0.46350400	1.56099900	1.71616900
O	-4.57473700	0.67808600	2.21950300
H	-5.77183900	1.85623200	3.45885800

H	-4.20906700	1.35259500	4.16385300
H	-1.95872300	0.25277600	1.99346700
H	-1.73571100	1.37833600	3.35951700
C	0.80214700	-2.85787200	1.83301800
C	-0.53194400	-2.99492800	2.56689700
O	-0.67426600	-3.54894800	3.63606400
O	-1.54345800	-2.38864100	1.90540700
C	-2.82478800	-2.41428300	2.55910800
H	-2.71765000	-2.13139600	3.60812100
H	-3.45132500	-1.70208100	2.02635800
H	-3.24919500	-3.41983600	2.50593700
O	0.63710400	-2.73371100	0.45019500
H	1.32357000	-3.80191600	2.04711700
C	1.64681400	-1.70769200	2.42827500
H	2.61629100	-1.74591300	1.92132200
H	1.19612500	-0.75977000	2.13037400
C	1.86684500	-1.76227900	3.93921000
H	2.34195100	-2.70098500	4.24117900
H	2.51347100	-0.93600200	4.24779000
H	0.92746600	-1.68308500	4.49328200
C	3.30156400	-3.94502200	-0.67116600
C	4.36701300	-3.48369700	0.31051800
O	4.92172500	-4.19574900	1.11175600
O	4.65738300	-2.16650400	0.23448200
C	3.87960900	-1.34532000	-0.67902200
C	3.77098900	-2.02571400	-2.03433000
O	3.80499200	-1.45708700	-3.10173400
O	3.57193500	-3.36171200	-1.98241700
H	2.33638200	-3.54998200	-0.32234600
H	2.85941700	-1.25610300	-0.28574500
C	3.22369200	-5.45318200	-0.83916500
H	2.66860800	-5.64515800	-1.76424700
H	4.23099200	-5.85840100	-0.97393100
C	4.51437700	0.03268400	-0.72319200
H	3.90456900	0.64041500	-1.39660300
H	4.41243800	0.46819700	0.27323600
C	2.52566800	-6.12367200	0.34736600
H	3.08921400	-5.95661200	1.26728000
H	1.51504700	-5.72323200	0.48368600
H	2.44391000	-7.20232000	0.18701100
C	5.97687000	0.04165700	-1.16410900
H	6.37527600	1.05897000	-1.11409000
H	6.58401400	-0.59109700	-0.51158700
H	6.08025900	-0.31558300	-2.19230700

TS1

C	-2.56447000	6.96640500	0.55431700
C	-3.12907500	5.94724100	-0.16970300
C	-2.41480500	4.74060300	-0.41285800
C	-1.08491100	4.57847900	0.11236900
C	-0.52370800	5.67298600	0.83302000
C	-1.24234900	6.82304000	1.05140800
H	-3.92788500	3.84693100	-1.65873800
H	-3.11610100	7.88246400	0.73964700
H	-4.13447600	6.04222600	-0.57142300
C	-2.95812400	3.70356800	-1.18749200
C	-0.38073800	3.36421000	-0.09559300
H	0.48751000	5.58111100	1.20970100
H	-0.79180100	7.63911200	1.60879400
C	-0.98247400	2.29367300	-0.77858900
C	-2.27896100	2.50893800	-1.38657300
C	1.40367000	-6.38676100	0.62197900
C	1.40549200	-5.78301600	1.85683500
C	1.79136200	-4.42440600	2.00400200
C	2.15607600	-3.66428400	0.84554800
C	2.14211500	-4.32180500	-0.41560200
C	1.78670000	-5.64587500	-0.52185900
H	1.57208700	-4.37218300	4.15474500
H	1.11314900	-7.42762400	0.52034500
H	1.12332100	-6.34099600	2.74570200
C	1.85305900	-3.80205300	3.27373700
C	2.55761800	-2.29890900	0.98260400
H	1.79436600	-6.12795700	-1.49459400
C	2.63495500	-1.75041900	2.25493700
C	2.27939900	-2.50281600	3.40293300
H	2.32700400	-2.04675600	4.38402100
C	1.02657700	3.19312100	0.35375100
C	2.07641300	3.10739900	-0.61044600
C	1.34320600	3.11066100	1.70363600
C	1.83470900	3.24179600	-2.00580300
C	3.42730200	2.90621200	-0.17945300
C	2.68153000	2.93509500	2.13122600
C	2.86616300	3.18459800	-2.91319800
C	4.46718000	2.83469100	-1.14241200
C	3.69158700	2.81749100	1.20710000
H	2.91001000	2.88311300	3.18748900
C	4.19823700	2.97661200	-2.48207300
H	2.65789000	3.30404900	-3.97285700

H	5.48148800	2.65431700	-0.79909000
H	4.71569600	2.66622500	1.53619200
H	5.00233600	2.92476600	-3.20976800
C	2.91277500	-1.51224600	-0.23060700
C	4.26373400	-1.19839700	-0.53123100
C	1.88263100	-1.14218500	-1.10957900
C	5.33513600	-1.50474000	0.35745400
C	4.59454600	-0.55150000	-1.77182900
C	2.23677300	-0.57411800	-2.39292400
C	6.62974500	-1.15897800	0.05589400
H	5.10975400	-2.02195800	1.28231500
C	5.94681900	-0.21254500	-2.05390700
C	3.56457900	-0.28947200	-2.68683800
C	6.94724900	-0.49753400	-1.15976400
H	7.42579800	-1.39910100	0.75488800
H	6.16755900	0.28444800	-2.99463200
H	3.81258900	0.15396200	-3.64737900
H	7.97644300	-0.23192700	-1.37922700
C	-2.74560700	1.60598500	-2.41145400
N	-2.24010900	0.44163900	-2.61271900
N	-0.01855000	-0.53339000	-3.28151600
C	-2.37214100	-0.24153200	-3.88728700
C	-0.92350100	-0.35125000	-4.42497300
O	-0.33217400	1.16386500	-0.90897300
O	0.63144200	-1.36903200	-0.79361100
C	1.25516900	-0.37289200	-3.42867200
Al	-0.85817600	-0.52066300	-1.43856500
H	-3.49007800	1.99929400	-3.11349400
H	1.64751200	-0.08070500	-4.40963200
H	-0.65591200	0.56254100	-4.97042600
H	-3.00810500	0.30415700	-4.59385900
H	-2.78329000	-1.23476500	-3.70635800
H	0.81929700	3.41050100	-2.34332300
H	2.43056800	-3.76184100	-1.29661500
C	3.64715100	-0.03961700	3.56569800
H	2.89919900	0.15122400	4.34686500
C	0.56039500	3.13685800	3.96852100
H	-0.41068800	3.21093800	4.45906400
H	-0.83775200	-1.19851700	-5.11390600
O	3.05148000	-0.44899100	2.34632100
O	0.29763300	3.21220200	2.58030700
H	1.20097600	3.96134600	4.30741100
H	1.02285100	2.18205200	4.24307000
H	4.17249400	0.88880100	3.34710000

H	4.36229900	-0.78512100	3.93344300
C	-1.98099600	-0.42079800	1.44734800
C	-2.89036200	0.81127800	1.57793300
O	-4.00828100	0.86898000	1.10454600
O	-2.32031300	1.79516600	2.28145200
C	-3.08044700	3.00355400	2.45721800
H	-3.75885900	2.88869500	3.30746500
H	-2.34479600	3.78334500	2.64323500
H	-3.65320200	3.22382100	1.55850300
O	-1.98659100	-0.81808300	0.08689700
H	-2.49330200	-1.20170600	2.03123200
C	-0.55563000	-0.28660200	1.98858900
H	0.00680000	-1.12830600	1.58083500
H	-0.09608900	0.61650000	1.58590000
C	-0.48364100	-0.30347900	3.51490600
H	-0.85463800	-1.25130400	3.92222700
H	0.55602700	-0.19927500	3.83513600
H	-1.05990200	0.51174100	3.95771500
C	-4.91931700	-1.93185100	-0.00937900
C	-5.03575300	-3.42459900	0.26719200
O	-6.06990100	-4.02059000	0.45004800
O	-3.85571500	-4.08700700	0.25312200
C	-2.61820100	-3.32773000	0.25304300
C	-2.68756300	-2.16293600	-0.75781100
O	-1.86715200	-2.11626200	-1.75420000
O	-3.95137600	-1.77577500	-1.06817100
H	-4.55204700	-1.40726000	0.87880700
H	-2.49883600	-2.91852200	1.25915900
C	-6.21210100	-1.26972300	-0.45631200
H	-5.92574000	-0.30492500	-0.88491700
H	-6.66991600	-1.87122600	-1.24806300
C	-1.47603900	-4.29333600	-0.03013800
H	-0.56593300	-3.69509800	-0.11764400
H	-1.35646500	-4.93792300	0.84581500
C	-7.18938700	-1.06005500	0.70151400
H	-7.47399100	-2.01522600	1.14737700
H	-6.73692800	-0.42797900	1.47291700
H	-8.09734700	-0.56110800	0.35032700
C	-1.68128900	-5.14676800	-1.28235000
H	-0.83776700	-5.83045300	-1.40160200
H	-2.59652500	-5.73937000	-1.20561100
H	-1.74298000	-4.51906400	-2.17320200

INT2

C	2.70097200	6.91536800	-0.53472500
C	3.23825000	5.89186300	0.20417400
C	2.50339000	4.69620600	0.43896600
C	1.18196900	4.54944300	-0.11082500
C	0.64869000	5.64806700	-0.84580600
C	1.38671200	6.78765800	-1.05556600
H	3.97804300	3.78925100	1.72159000
H	3.26833300	7.82307400	-0.71352900
H	4.23700700	5.97536900	0.62453400
C	3.01665900	3.65618200	1.23067900
C	0.45863700	3.34413700	0.08758100
H	-0.35649500	5.56823700	-1.24092500
H	0.95749400	7.60721000	-1.62454600
C	1.03378800	2.26910100	0.78501100
C	2.31764500	2.47242200	1.42204200
C	-1.55941200	-6.35978600	-0.58778000
C	-1.53320100	-5.76218700	-1.82520900
C	-1.88070300	-4.39435300	-1.98202700
C	-2.23563500	-3.61901400	-0.83064900
C	-2.25208000	-4.27084600	0.43329300
C	-1.93365000	-5.60346100	0.54876600
H	-1.63920400	-4.35868300	-4.13063100
H	-1.29736300	-7.40735200	-0.47837700
H	-1.25733200	-6.33158800	-2.70877200
C	-1.91304000	-3.77682000	-3.25506500
C	-2.59847900	-2.24362600	-0.97774800
H	-1.96370600	-6.08063000	1.52346600
C	-2.64961000	-1.69999900	-2.25339700
C	-2.30326200	-2.46735800	-3.39434900
H	-2.33042500	-2.01535100	-4.37810200
C	-0.94567700	3.19551600	-0.37890100
C	-2.00821500	3.13920700	0.57371800
C	-1.24876200	3.11399800	-1.73167800
C	-1.77948600	3.27314800	1.97132900
C	-3.35866200	2.96978300	0.12783800
C	-2.58609900	2.97195300	-2.17421400
C	-2.82251600	3.24426600	2.86679400
C	-4.41100600	2.92676700	1.07883700
C	-3.60929400	2.88400800	-1.26136100
H	-2.80391000	2.92271100	-3.23284400
C	-4.15410400	3.06679000	2.42102600
H	-2.62360900	3.36248600	3.92837500
H	-5.42538700	2.76974300	0.72442500
H	-4.63328100	2.75908900	-1.60170300

H	-4.96749800	3.03652900	3.13949400
C	-2.94628400	-1.44413800	0.22949100
C	-4.29252700	-1.10041200	0.51761600
C	-1.91649000	-1.09597600	1.11808100
C	-5.36189900	-1.38191800	-0.38177400
C	-4.62097500	-0.44749100	1.75590000
C	-2.27034200	-0.52279600	2.39963600
C	-6.65116300	-1.00714200	-0.09255300
H	-5.13919300	-1.90342000	-1.30484800
C	-5.96811200	-0.07845500	2.02510000
C	-3.59478800	-0.20988900	2.68135600
C	-6.96569100	-0.33978100	1.12076900
H	-7.44554700	-1.22856500	-0.79956000
H	-6.18695200	0.42228100	2.96429000
H	-3.84250900	0.23684200	3.64050300
H	-7.99075500	-0.05124800	1.33032900
C	2.75455000	1.56644000	2.45768300
N	2.21614300	0.41814300	2.66060200
N	-0.02308700	-0.53036800	3.31120400
C	2.33067400	-0.27773200	3.92968900
C	0.87803300	-0.36669900	4.46087400
O	0.36888500	1.14450500	0.89922700
O	-0.66750400	-1.34768600	0.81324700
C	-1.29522200	-0.34376200	3.44536100
Al	0.83151200	-0.53804600	1.48591300
H	3.50625800	1.94403400	3.16049500
H	-1.69066700	-0.04403900	4.42268000
H	0.62093000	0.54850300	5.00891500
H	2.97435800	0.24869300	4.64377300
H	2.72311400	-1.27672800	3.73697900
H	-0.76439600	3.41847400	2.32042300
H	-2.53375100	-3.69952600	1.30917000
C	-3.61517300	0.02278200	-3.58194700
H	-2.86029800	0.19339200	-4.36120500
C	-0.43936300	3.10285900	-3.98745900
H	0.53879300	3.14706100	-4.46743200
H	0.77553600	-1.21599000	5.14491300
O	-3.03276700	-0.38896700	-2.35677200
O	-0.19071000	3.18457400	-2.59691700
H	-1.05433800	3.94030400	-4.34152700
H	-0.92349100	2.15777500	-4.25839200
H	-4.12373900	0.96262400	-3.37309200
H	-4.34264900	-0.71174200	-3.94745900
C	2.00831700	-0.48445900	-1.44840600

C	2.92057000	0.74818700	-1.53507200
O	4.00293000	0.81701400	-0.98992900
O	2.39188100	1.71364900	-2.29418400
C	3.14982400	2.92861100	-2.43736400
H	3.84658200	2.82566900	-3.27407500
H	2.41532600	3.70775300	-2.63014500
H	3.70105900	3.13945900	-1.52322100
O	1.96155100	-0.88988300	-0.08125700
H	2.52999800	-1.26466900	-2.01854400
C	0.59052000	-0.33941500	-2.00477700
H	0.01401500	-1.17267800	-1.59886200
H	0.13753500	0.57056000	-1.60978500
C	0.53516200	-0.36243900	-3.53166900
H	0.89839100	-1.31687200	-3.93031400
H	-0.49981200	-0.24708400	-3.86271700
H	1.12632100	0.44341200	-3.97145800
C	4.88766300	-2.02889800	-0.04541600
C	4.95013900	-3.53938900	-0.22937300
O	5.96159900	-4.18770500	-0.35579700
O	3.74506800	-4.15331600	-0.19446000
C	2.53800700	-3.35176400	-0.26015800
C	2.62376600	-2.12280600	0.68353200
O	1.85033900	-2.09992700	1.74558200
O	3.92131700	-1.77557100	0.98741100
H	4.56105700	-1.55266400	-0.97726100
H	2.45235900	-3.00071200	-1.29250600
C	6.20178200	-1.39336800	0.37970100
H	5.94942200	-0.39073200	0.73594300
H	6.61558000	-1.95920700	1.22045500
C	1.35726800	-4.26363400	0.04817700
H	0.47175700	-3.62828100	0.12627900
H	1.20682800	-4.92370800	-0.81164300
C	7.21239100	-1.30786500	-0.76490400
H	7.46385900	-2.30312900	-1.13671200
H	6.80771900	-0.71335300	-1.59148900
H	8.13368800	-0.82375500	-0.42772500
C	1.53170300	-5.09811100	1.31819500
H	0.65523400	-5.73451500	1.46000100
H	2.41434300	-5.73850900	1.24581200
H	1.63667000	-4.45295700	2.19159300

INT3

C	7.11672900	-3.21594200	-0.05480500
C	6.74525500	-2.07050400	-0.71119500
C	5.38366400	-1.65777900	-0.74302600
C	4.38216000	-2.43288900	-0.06605800
C	4.80652900	-3.62636300	0.58433500
C	6.12790700	-4.00322600	0.58901400
H	5.72158800	0.06667400	-1.98996300
H	8.15612800	-3.52743900	-0.03358700
H	7.48235300	-1.45774900	-1.22287500
C	4.97944800	-0.51765200	-1.45250900
C	3.02112500	-2.00521800	-0.06173300
H	4.06297000	-4.24444900	1.07212100
H	6.42091300	-4.92087500	1.09072800
C	2.64462000	-0.84312500	-0.75002300
C	3.65115200	-0.11862300	-1.48974500
C	-7.08181100	1.05250800	1.62030100
C	-6.13735900	1.00793600	2.61785100
C	-4.85444500	0.44617900	2.38864000
C	-4.53255900	-0.09525500	1.10077400
C	-5.53300900	-0.02039700	0.09034000
C	-6.76731400	0.53448400	0.34258000
H	-4.11807000	0.81211700	4.38967300
H	-8.05975700	1.48526500	1.80621300
H	-6.35564200	1.40863200	3.60428200
C	-3.87130800	0.41855300	3.40756800
C	-3.22961900	-0.64638200	0.86003600
H	-7.50779600	0.57622100	-0.45094800
C	-2.28974800	-0.60735100	1.88264300
C	-2.62296600	-0.09292700	3.16285500
H	-1.88025400	-0.11887300	3.95086600
C	2.00288500	-2.77313200	0.70602300
C	0.91493300	-3.43148500	0.05979400
C	2.10027100	-2.83673100	2.09321700
C	0.75450900	-3.42445700	-1.35253200
C	-0.05771100	-4.13302800	0.84322300
C	1.16116600	-3.56074800	2.86502300
C	-0.31233200	-4.05585600	-1.94625900
C	-1.15164200	-4.76646400	0.19843600
C	0.10211400	-4.18179700	2.24641100
H	1.25579500	-3.60436600	3.94265700
C	-1.28259400	-4.73113900	-1.16755700
H	-0.42017800	-4.03007600	-3.02615600
H	-1.89253100	-5.27403500	0.80959200
H	-0.63230700	-4.71856000	2.84004400
H	-2.13173700	-5.20253000	-1.64926500
C	-2.89536200	-1.16905400	-0.49199300

C	-3.60742500	-2.27160300	-1.03476700
C	-1.92515600	-0.51547700	-1.27398300
C	-4.45782500	-3.08772000	-0.23082400
C	-3.48500600	-2.61466500	-2.42810500
C	-1.85473600	-0.82840600	-2.68722700
C	-5.13434800	-4.15472500	-0.76626700
H	-4.54537700	-2.85843600	0.82413600
C	-4.21929000	-3.71658000	-2.95454100
C	-2.63821100	-1.84481700	-3.23093900
C	-5.02597300	-4.47506500	-2.14701600
H	-5.76215900	-4.76705400	-0.12581900
H	-4.11346900	-3.94951500	-4.01086900
H	-2.57641500	-2.05534200	-4.29646500
H	-5.57641000	-5.31825100	-2.55167000
C	3.30019000	0.96757300	-2.36593600
N	2.11411900	1.46011700	-2.44983000
N	-0.18980800	0.85310600	-3.22782800
C	1.71669300	2.33168100	-3.53962500
C	0.56963700	1.58413800	-4.25352600
O	1.38439300	-0.44141400	-0.72423300
O	-1.15712800	0.40985600	-0.76721100
C	-1.04437900	-0.05660600	-3.58359700
Al	0.47128600	0.97974200	-1.34799800
H	4.08701500	1.34350000	-3.02678000
H	-1.17290100	-0.26667700	-4.65129900
H	0.97830200	0.87931900	-4.98809200
H	2.53878000	2.55112200	-4.22941800
H	1.34615400	3.26514700	-3.11308400
H	1.49357300	-2.91283400	-1.95732500
H	-5.31128000	-0.40356100	-0.89701300
C	0.03825300	-0.42945500	2.27543300
H	-0.13830000	0.64601300	2.34123600
C	3.32179400	-2.20172400	4.05764700
H	2.46497800	-1.80503500	4.61718300
H	-0.08238300	2.28964400	-4.77936500
O	-1.04849600	-1.09498700	1.63277500
O	3.12138400	-2.11919600	2.65892300
H	4.19902500	-1.58903400	4.26872400
H	3.51440400	-3.23222100	4.38145000
H	0.90418000	-0.60024900	1.64692900
H	0.21936500	-0.83964200	3.27666400
C	-1.43245400	3.14112800	1.15698000
O	-0.16116700	3.77603700	1.13061700
C	1.71393200	4.44028700	-0.05633800
C	0.78987800	3.24432100	0.23551700
O	0.24123900	2.68643300	-0.85850600
O	1.53661100	2.19529200	0.89929000
C	2.85514500	2.52078300	1.35154900
C	3.63469800	3.03584600	0.13777000
O	4.72324900	2.64649000	-0.22053800
O	2.97165200	3.95890100	-0.59481600
H	1.91557000	4.94587500	0.89487900
H	2.80525500	3.32430000	2.10424200
C	1.11813600	5.43117500	-1.03730300
H	0.11636700	5.67619200	-0.67497000
C	3.45728800	1.27468100	1.98641600
H	3.61229600	0.51751100	1.21735100
H	2.70488800	0.87542100	2.67314700
H	0.97447000	4.92651600	-1.99643400
H	-1.33631100	2.05344500	1.13652300
C	4.76654400	1.54186200	2.72890700
H	4.63328100	2.27866700	3.52978600
H	5.13502100	0.61693600	3.18225300
H	5.53152900	1.91037300	2.04287000
C	1.98149800	6.68104600	-1.20832000
H	2.98401200	6.42217400	-1.56126900
H	1.53155700	7.36804300	-1.93101100
H	2.08880900	7.22041400	-0.26041500
C	-2.26482100	3.56027500	-0.05068900
C	-2.13908500	3.59323500	2.44035400
H	-2.31366100	4.67339200	2.37020700
H	-3.11614900	3.10081900	2.46965500
C	-1.33822500	3.27436800	3.70174000
H	-1.18559000	2.19684800	3.80447600
H	-0.35793000	3.75464500	3.66603200
H	-1.86536500	3.62492800	4.59468300
O	-2.14178400	4.58842700	-0.67967400
O	-3.23669700	2.65396100	-0.27140300
C	-4.11771900	2.94339200	-1.36317600
H	-3.57786000	2.86358900	-2.31100000
H	-4.90711200	2.19663500	-1.30471100
H	-4.52688900	3.95229600	-1.27358200

TS2

C	7.96553800	-1.49004600	-0.60602100
C	7.29785100	-0.38635300	-1.07056900
C	5.88266800	-0.28603100	-0.95201400
C	5.13705400	-1.34400600	-0.32739900

C	5.86815200	-2.47885100	0.12854200
C	7.23305500	-2.54846200	-0.00768100
H	5.72783600	1.62307300	-1.93606100
H	9.04435700	-1.56271100	-0.69950400
H	7.83575300	0.43253900	-1.54076200
C	5.18004600	0.81993000	-1.44869700
C	3.72354900	-1.24624400	-0.18767400
H	5.32267700	-3.29997600	0.57751300
H	7.76194200	-3.42953000	0.34412300
C	3.03343900	-0.13313100	-0.69809200
C	3.79776900	0.91198000	-1.34150500
C	-6.93425000	-0.20455600	1.73543600
C	-6.06495700	-0.40923600	2.78172900
C	-4.72593900	-0.81798300	2.55029700
C	-4.27566100	-1.03508200	1.20653600
C	-5.20315200	-0.81806000	0.15078800
C	-6.49192100	-0.40928600	0.40726300
H	-4.15609300	-0.83648100	4.63760400
H	-7.95410700	0.11600600	1.92394400
H	-6.38696000	-0.24962100	3.80730900
C	-3.80907000	-0.98721200	3.61918900
C	-2.91322500	-1.41326400	0.96348400
H	-7.17688300	-0.24025000	-0.41827700
C	-2.04987600	-1.51462200	2.04100700
C	-2.49985800	-1.31152400	3.37133800
H	-1.79258900	-1.43331000	4.18447200
C	2.97297600	-2.28443900	0.56798900
C	1.97795200	-3.09868700	-0.05102100
C	3.21010400	-2.43003900	1.93343200
C	1.68660800	-3.01411800	-1.43956800
C	1.23041000	-4.03156300	0.73713900
C	2.50780500	-3.38873300	2.70210100
C	0.69351900	-3.78046800	-2.00068400
C	0.20064900	-4.79588300	0.12874100
C	1.53225100	-4.15755200	2.11188600
H	2.70895600	-3.49565300	3.76048600
C	-0.07191700	-4.67243100	-1.21086800
H	0.47991000	-3.69250300	-3.06089800
H	-0.37748900	-5.47419800	0.74990700
H	0.96896800	-4.86861200	2.70915700
H	-0.87469300	-5.24365900	-1.66449200
C	-2.45235800	-1.69544000	-0.42322700
C	-2.97373900	-2.82927100	-1.10319100
C	-1.54965200	-0.83077500	-1.07091000
C	-3.74178600	-3.82694300	-0.43224400
C	-2.72519900	-3.02176600	-2.50608000
C	-1.38637600	-0.98724300	-2.50218900
C	-4.21335700	-4.93062700	-1.09929700
H	-3.92862300	-3.71235800	0.62838200
C	-3.24355700	-4.16841200	-3.17103900
C	-1.97356000	-2.05184100	-3.17827700
C	-3.96960300	-5.10963700	-2.48708700
H	-4.77938800	-5.68221700	-0.55698800
H	-3.03969600	-4.28659100	-4.23215900
H	-1.83446900	-2.13847500	-4.25396400
H	-4.35478700	-5.98734300	-2.99603800
C	3.14072400	1.99564700	-2.01499200
N	1.88238900	2.27219800	-1.92936900
N	-0.07866900	1.02100700	-2.84159800
C	1.26932700	3.05641400	-2.99059600
C	0.42798500	2.03140300	-3.78222100
O	1.72693200	-0.05444300	-0.59408700
O	-0.94753300	0.12918300	-0.41784900
C	-0.72709500	0.00695600	-3.30266600
Al	0.45289200	1.23478100	-0.87202500
H	3.75611600	2.57557100	-2.71079300
H	-0.82859800	-0.11083600	-4.38846000
H	1.05706800	1.54628800	-4.53943100
H	2.01608200	3.52785600	-3.63912300
H	0.63245200	3.82045600	-2.55145300
H	2.26197400	-2.32855100	-2.05005500
H	-4.87069200	-0.96302500	-0.86827500
C	0.20275600	-0.92310400	2.32477100
H	-0.17329000	0.09932600	2.25106400
C	4.44982200	-1.70894900	3.85443300
H	3.58271100	-1.52901600	4.50293700
H	-0.40501000	2.52569800	-4.29360100
O	-0.75442900	-1.84840100	1.79950300
O	4.12136300	-1.56822500	2.48518400
H	5.20957600	-0.95304800	4.05712100
H	4.86185900	-2.70174100	4.07540300
H	1.08027200	-1.01275800	1.69374000
H	0.46261000	-1.16450800	3.36351200
C	-2.81674300	2.37424100	0.67973800
O	-1.77231900	3.33653900	0.97482400
C	0.05228300	4.66181800	0.42815300
C	-0.72201900	3.38055000	0.15465300
O	-0.75220700	2.82526200	-0.98791700

O	0.57905800	2.09790500	0.83658500
C	1.64960400	2.68958000	1.54104700
C	2.28956900	3.75419800	0.63944400
O	3.47004200	3.87162300	0.41645100
O	1.41559900	4.58289100	-0.00655700
H	0.01620400	4.85774000	1.50483500
H	1.21799300	3.21549200	2.41012200
C	-0.60058700	5.81781700	-0.33139300
H	-1.66916800	5.80827200	-0.09620500
C	2.67797800	1.68997800	2.07305600
H	3.29064300	1.31824200	1.25350300
H	2.12539100	0.83729200	2.47047200
H	-0.51663800	5.60625700	-1.40242900
H	-2.39014800	1.37317200	0.74092500
C	3.58107200	2.28273700	3.15558100
H	2.99955200	2.64122000	4.01354100
H	4.27840200	1.52205300	3.51839900
H	4.16680400	3.11668200	2.76252000
C	0.03169600	7.16749600	0.00486300
H	1.10049400	7.16249400	-0.22315900
H	-0.43991500	7.96877300	-0.57100400
H	-0.08720300	7.40495500	1.06775800
C	-3.34873200	2.62928700	-0.72600300
C	-3.91934300	2.60000700	1.70873900
H	-4.35469500	3.58980400	1.53133500
H	-4.70072300	1.85898900	1.51379000
C	-3.41973400	2.48041800	3.14817900
H	-2.93649300	1.51516600	3.31538100
H	-2.69920500	3.26939000	3.37751400
H	-4.25560100	2.56317000	3.84912400
O	-3.60232500	3.72258600	-1.18198000
O	-3.57494300	1.46715100	-1.36126600
C	-4.09808000	1.57590200	-2.68962200
H	-3.42348700	2.16955200	-3.31133500
H	-4.16191200	0.55378200	-3.06110300
H	-5.08278400	2.04956500	-2.68071100

INT4

C	8.24427200	-0.47206300	-0.55133500
C	7.44171400	0.55642200	-0.97445400
C	6.02638000	0.47754600	-0.84549500
C	5.42502900	-0.68594800	-0.25420400
C	6.29241900	-1.73750400	0.15727000
C	7.65466400	-1.63266700	0.01313000
H	5.62960500	2.37376700	-1.78701600
H	9.32287000	-0.40730100	-0.65274300
H	7.87124900	1.44986000	-1.41967800
C	5.18777900	1.50225800	-1.30935600
C	4.01074600	-0.76708900	-0.10709400
H	5.85535900	-2.63384400	0.58005300
H	8.29191100	-2.45213600	0.33243600
C	3.19375900	0.27818100	-0.55696400
C	3.80578000	1.42362600	-1.18872200
C	-6.45490300	-1.99459400	2.03875500
C	-5.50783700	-1.97285200	3.03557000
C	-4.12232000	-1.96951800	2.72925800
C	-3.69868400	-2.00840800	1.36068400
C	-4.70720300	-2.02057400	0.35581600
C	-6.04337000	-2.01069800	0.68551300
H	-3.46037000	-1.87089100	4.78629600
H	-7.51209800	-1.98874300	2.28472600
H	-5.80567000	-1.94476600	4.08034400
C	-3.14076700	-1.88714500	3.74810700
C	-2.29872900	-1.97457500	1.04282500
H	-6.79030800	-2.00752200	-0.10271400
C	-1.38473500	-1.82749400	2.07701800
C	-1.80973100	-1.79955200	3.43151900
H	-1.06322300	-1.72209500	4.21343800
C	3.38298300	-1.94896400	0.54635700
C	2.53980500	-2.84272400	-0.17823000
C	3.59188500	-2.16999400	1.90444900
C	2.28476800	-2.68647100	-1.56779300
C	1.91958800	-3.94130900	0.49979800
C	3.00406900	-3.27322200	2.56867500
C	1.45192900	-3.55237500	-2.23577500
C	1.05951100	-4.81170300	-0.21857700
C	2.18020400	-4.12831200	1.87609600
H	3.17938400	-3.43312700	3.62497000
C	0.82456500	-4.62522500	-1.55833300
H	1.26232300	-3.40798200	-3.29478900
H	0.57877400	-5.62577400	0.31664500
H	1.71066100	-4.96196700	2.39035500
H	0.14988400	-5.28322700	-2.09446300
C	-1.87255600	-2.05712000	-0.37896500
C	-2.18290500	-3.21559600	-1.14401400
C	-1.23979200	-0.96475100	-0.99316600
C	-2.65412500	-4.41701900	-0.53726900
C	-2.01632900	-3.21865000	-2.57293400

C	-1.13859200	-0.95573000	-2.43440000
C	-2.93955400	-5.53031100	-1.28820800
H	-2.76453700	-4.44415700	0.53969100
C	-2.33854600	-4.38503900	-3.32342200
C	-1.53603900	-2.05580100	-3.18716100
C	-2.78995500	-5.52025500	-2.70075500
H	-3.28205300	-6.43533600	-0.79542700
H	-2.20622900	-4.35800300	-4.40189900
H	-1.45893800	-2.01757000	-4.27173300
H	-3.02703500	-6.40886100	-3.27686600
C	3.01183000	2.44707900	-1.82150800
N	1.72960800	2.52659400	-1.74185700
N	-0.24555400	1.29199700	-2.58633800
C	0.97439800	3.39445700	-2.63403900
C	0.06091300	2.45025800	-3.43788800
O	1.88382700	0.18781500	-0.41924300
O	-0.83353300	0.06706800	-0.29432000
C	-0.73630000	0.22886100	-3.13572200
Al	0.48020800	1.26963200	-0.70867100
H	3.54993100	3.16611500	-2.44778600
H	-0.87363400	0.21662600	-4.22273900
H	0.56056400	2.11296200	-4.35418200
H	1.62569700	3.98074100	-3.29050600
H	0.35820600	4.07349900	-2.04539100
H	2.76148000	-1.86896900	-2.09551900
H	-4.40828800	-2.00880500	-0.68322400
C	0.66158500	-0.72146100	2.44789500
H	0.06979700	0.19796600	2.48754100
C	4.65211100	-1.43729000	3.92452700
H	3.74409000	-1.42537000	4.54127300
H	-0.86773000	2.96187200	-3.70315900
O	-0.06334500	-1.74860300	1.76876500
O	4.36006700	-1.24079500	2.55385400
H	5.29213100	-0.60331400	4.21533400
H	5.18805100	-2.37932200	4.09705900
H	1.55215700	-0.54680400	1.85340300
H	0.95060400	-1.03275500	3.45968700
C	-3.43855900	1.82883900	0.42292500
O	-2.90537500	3.13930200	0.72245200
C	-1.44229900	4.93464900	0.47266000
C	-2.06252100	3.69134100	-0.15492000
O	-1.89816200	3.32792500	-1.30129000
O	0.07002200	2.38445600	0.60568600
C	0.81752200	3.33869900	1.30446100

C	0.99178900	4.58047300	0.41203700
O	2.05408400	5.00483500	0.02021600
O	-0.13694900	5.19842500	-0.05517500
H	-1.39201800	4.78493800	1.55371900
H	0.23255600	3.64392500	2.18898800
C	-2.30500000	6.15843300	0.15467400
H	-3.32805800	5.93437600	0.47567600
C	2.16838300	2.82721500	1.81195800
H	2.84075900	2.67474300	0.96532500
H	1.98345600	1.84560600	2.25777600
H	-2.32822600	6.27540500	-0.93425700
H	-2.60107700	1.12920700	0.40905800
C	2.82707500	3.75722300	2.83158900
H	2.17425200	3.92672500	3.69621700
H	3.76094900	3.32414600	3.20194000
H	3.06012100	4.72568600	2.38260000
C	-1.78905100	7.43053500	0.82582000
H	-0.76554600	7.64544700	0.50925400
H	-2.41685500	8.28754000	0.56595800
H	-1.79381300	7.33000500	1.91689500
C	-4.14440000	1.81541800	-0.92377000
C	-4.42720800	1.50478300	1.53973800
H	-5.24108100	2.23724900	1.49656700
H	-4.85947700	0.52394600	1.32493000
C	-3.76506100	1.50118400	2.91683900
H	-2.93224500	0.79432700	2.94473000
H	-3.38142000	2.49317000	3.16855000
H	-4.48327500	1.20151400	3.68512800
O	-4.90787800	2.66107000	-1.32980300
O	-3.86765000	0.66348600	-1.57062300
C	-4.54522700	0.47594100	-2.81898400
H	-4.30698200	1.29101000	-3.50670400
H	-4.18215800	-0.47570400	-3.20585400
H	-5.62735800	0.44625200	-2.66898800

INT5

C	-8.03666500	-4.06501000	1.58136900
C	-6.87892300	-4.61226600	1.09083500
C	-5.80219500	-3.78293700	0.66839300
C	-5.92654900	-2.35270700	0.73831100
C	-7.13821500	-1.82551900	1.27344900
C	-8.15628800	-2.65468300	1.67829100
H	-4.48238500	-5.40368000	0.15360600
H	-8.85443700	-4.70148200	1.90378500

H	-6.75898300	-5.69019700	1.02165500
C	-4.59676700	-4.32284900	0.19932600
C	-4.86143800	-1.51614700	0.29457800
H	-7.24665600	-0.75216100	1.36130600
H	-9.06709200	-2.22367700	2.08316200
C	-3.66073500	-2.07339200	-0.15989000
C	-3.53685400	-3.51188500	-0.18765100
C	0.84879400	3.89004500	3.76573500
C	-0.33739700	4.34072300	3.23427000
C	-0.70419300	4.02907600	1.89902600
C	0.18305700	3.24124900	1.09462000
C	1.39902200	2.78822600	1.67526200
C	1.72052100	3.10329900	2.97649500
H	-2.58788500	5.09309500	1.93491400
H	1.11662400	4.13154000	4.78964400
H	-1.01802300	4.94118800	3.83211800
C	-1.92859500	4.47042700	1.33590500
C	-0.18629600	2.90993200	-0.24598800
H	2.65192200	2.74287000	3.40130500
C	-1.42489500	3.30640700	-0.72543000
C	-2.28659700	4.10895800	0.06139900
H	-3.22676600	4.43519100	-0.36642700
C	-5.00184700	-0.03856100	0.37115200
C	-5.93671200	0.66264300	-0.45041800
C	-4.23546300	0.67331200	1.28542200
C	-6.68650200	0.01215200	-1.46956400
C	-6.12355300	2.07289900	-0.27213300
C	-4.42447400	2.06568200	1.46148600
C	-7.57298700	0.71165800	-2.25523400
C	-7.05279500	2.76284800	-1.09411100
C	-5.35683100	2.74057800	0.71376700
H	-3.82815300	2.59343800	2.19361700
C	-7.76613300	2.10101200	-2.06563400
H	-8.12941300	0.19388000	-3.03082300
H	-7.18449800	3.83106500	-0.94260600
H	-5.50570200	3.80663800	0.86270800
H	-8.47183700	2.63959300	-2.69045800
C	0.74068200	2.18693300	-1.15653000
C	1.87324800	2.85294200	-1.69619900
C	0.46486000	0.85777700	-1.50164700
C	2.20486300	4.19581800	-1.34747200
C	2.74478800	2.17461500	-2.61980000
C	1.34467400	0.19000700	-2.42917200
C	3.32679800	4.80442600	-1.85513400
H	1.55725600	4.73201400	-0.66545200
C	3.89444000	2.84008500	-3.13278200
C	2.44151600	0.85387900	-2.97160300
C	4.18865200	4.12465900	-2.75596100
H	3.55812600	5.82502500	-1.56546100
H	4.53777100	2.30150700	-3.82343100
H	3.09198200	0.32660400	-3.66616000
H	5.07053400	4.62487800	-3.14255300
C	-2.32806800	-4.15538600	-0.62756100
N	-1.24083200	-3.52445700	-0.89854200
N	0.27849600	-1.98622500	-2.28257800
C	-0.06172400	-4.19905600	-1.41112200
C	0.32535100	-3.40153300	-2.66034200
O	-2.68315400	-1.28629900	-0.57873300
O	-0.56963800	0.23408600	-0.98543000
C	1.17296700	-1.19102700	-2.77215900
Al	-0.90521300	-1.53363500	-0.68564200
H	-2.35598100	-5.24613700	-0.72988800
H	1.89824600	-1.59717700	-3.48396900
H	-0.41313100	-3.58500500	-3.45080400
H	-0.24499100	-5.25495500	-1.64249300
H	0.73596400	-4.10806800	-0.66692000
H	-6.54059600	-1.05041600	-1.62423900
H	2.05968800	2.16460700	1.08746500
C	-2.96055800	2.16200700	-2.11292200
H	-3.85432000	2.70820300	-1.79579500
C	-2.08311700	0.60176400	2.29592000
H	-1.64627000	0.97304100	1.36518800
H	1.31628300	-3.68549500	-3.02923100
O	-1.77488400	2.96754500	-2.00463700
O	-3.34521800	-0.02408100	2.04590800
H	-1.44646500	-0.18060600	2.70735600
H	-2.16226300	1.42043700	3.02007700
H	-2.85840000	1.24658600	-1.52487800
H	-3.05393100	1.91279800	-3.17122900
C	7.15267200	-2.22072300	-0.12976500
O	6.08478800	-1.35708800	-0.53100200
C	4.25093100	0.02918100	-0.05687700
C	5.17842600	-1.06820900	0.43257800
O	5.17369000	-1.57253300	1.53103800
O	-0.00419300	-1.85988100	0.78921700
C	1.00327800	-1.08757200	1.35543800
C	2.26970500	-1.14018100	0.50195900
O	2.60498100	-2.04733300	-0.23354800

O	3.01347900	-0.01762700	0.66135900
H	4.05431500	-0.13022100	-1.11850900
H	0.72411300	-0.02657100	1.43220000
C	4.87079200	1.41191400	0.17265400
H	4.16738500	2.14973600	-0.22195300
C	1.31458200	-1.61735600	2.77034600
H	1.66390200	-2.65125400	2.66807900
H	0.34616900	-1.66539900	3.28053400
H	5.77562300	1.46800900	-0.44046700
H	6.74647200	-3.06792600	0.43021400
C	2.30803600	-0.78247900	3.57952400
H	3.30762100	-0.80845100	3.13988800
H	1.98567900	0.26257700	3.63470800
H	2.38006600	-1.16531700	4.60247000
C	5.20200200	1.70819100	1.63628500
H	5.51862000	2.74935400	1.74435900
H	4.32891600	1.54813600	2.27334900
H	6.01066500	1.06819300	1.99600100
C	8.08701700	-1.44330100	0.79369600
C	7.86352500	-2.69280900	-1.39783800
H	8.24372300	-1.81237800	-1.92859700
H	8.73024100	-3.28599500	-1.09005900
C	6.94409000	-3.50956400	-2.30642100
H	7.47151400	-3.82269600	-3.21195100
H	6.58635500	-4.40944600	-1.79491000
H	6.07112700	-2.92218800	-2.60167000
O	8.06746100	-0.24598500	0.96371900
O	8.96356900	-2.28248600	1.37314200
C	9.90991700	-1.66045100	2.26174600
H	10.53019800	-2.47017900	2.64319700
H	10.51629400	-0.92933200	1.72246200
H	9.38682700	-1.15662500	3.07720700

re-enantioface: ***R,R*-Al-*S*-M2B + L-EG**

INT1

C	7.15150700	-2.22180000	-1.53921200
C	6.27645200	-2.90015100	-0.72938200
C	5.05433900	-2.30214100	-0.31105000
C	4.72915400	-0.96863700	-0.74656000
C	5.67441200	-0.28985300	-1.56933800
C	6.84228600	-0.90003300	-1.95677100
H	4.44585300	-3.91818200	0.97668100
H	8.07926000	-2.68602200	-1.85812500
H	6.49983600	-3.90917700	-0.39291400
C	4.16873900	-2.95161500	0.56237900
C	3.50426300	-0.37446100	-0.35612500
H	5.44752000	0.71930700	-1.89252200
H	7.54243300	-0.36502000	-2.59172900
C	2.56083700	-1.09763800	0.38759200
C	2.95281900	-2.38304300	0.92357600
C	-6.48221800	1.54886100	0.63401200
C	-6.16810700	1.67060600	-0.69911500
C	-4.82540200	1.86541800	-1.11732300
C	-3.78528900	1.96399400	-0.13551800
C	-4.14551000	1.82002800	1.23217200
C	-5.45417200	1.61514000	1.60520100
H	-5.26656800	1.87269100	-3.23529600
H	-7.51183200	1.39820200	0.94351100
H	-6.94419200	1.61615600	-1.45780000
C	-4.48601100	1.97790600	-2.48717300
C	-2.44295000	2.25429900	-0.54009100
H	-5.70232100	1.50741700	2.65676900
C	-2.17654700	2.39263900	-1.89436100
C	-3.19138400	2.22544600	-2.87025800
H	-2.94272200	2.30713400	-3.92171800
C	3.20565400	1.05407600	-0.64176400
C	3.36773500	2.02446200	0.39191600
C	2.75661800	1.46165600	-1.88702100
C	3.81976900	1.67301400	1.69449600
C	3.07289300	3.40118900	0.13000400
C	2.47114400	2.82428900	-2.14855900
C	3.98568700	2.62858500	2.67018200
C	3.26345900	4.36301500	1.15490900
C	2.61495900	3.76543500	-1.15889400
H	2.14497700	3.12181200	-3.13597400
C	3.71262700	3.99155200	2.40010900
H	4.34179000	2.33764600	3.65458300
H	3.03076600	5.40088000	0.93743200
H	2.38100100	4.80724400	-1.35794300
H	3.85814300	4.73757300	3.17582500
C	-1.40551700	2.55912800	0.48661700
C	-1.02745700	3.91248500	0.70837200
C	-0.86194800	1.52974400	1.27154600
C	-1.52709300	4.98879300	-0.08298800
C	-0.11656900	4.24496300	1.76898800
C	-0.01189700	1.89168500	2.38726300
C	-1.12355900	6.28370300	0.13548300

H	-2.24694600	4.77181800	-0.86248600
C	0.27344700	5.59714700	1.97359600
C	0.34478600	3.21557600	2.60219600
C	-0.20622700	6.60072400	1.17074000
H	-1.51975600	7.08021400	-0.48801700
H	0.96808400	5.81378700	2.77997900
H	1.00338800	3.46132300	3.43003400
H	0.09873000	7.63015900	1.32977100
C	2.23142500	-2.93378800	2.03971700
N	1.06012100	-2.54793000	2.42330100
N	0.20554200	-0.35500000	3.26780600
C	0.68308200	-2.68629100	3.82167900
C	0.64215500	-1.23473800	4.35791000
O	1.39612100	-0.56172200	0.64480900
O	-1.16400000	0.27693700	1.03664400
C	0.41049700	0.91678100	3.35910700
Al	-0.13895300	-1.20054400	1.42406500
H	2.78848400	-3.63223900	2.67543400
H	0.92712900	1.31422400	4.24043300
H	1.63965000	-0.93612000	4.70484600
H	1.40071100	-3.28924200	4.39015400
H	-0.30736200	-3.13298800	3.88961400
H	4.04551500	0.63347300	1.90180800
H	-3.36789900	1.88105100	1.98281000
C	-0.75549400	3.56389100	-3.39497700
H	-0.79037500	2.99750600	-4.33439600
C	1.77912100	0.74713600	-3.94756500
H	1.63638600	-0.21413900	-4.43974600
H	-0.04998100	-1.16146400	5.20409600
O	-0.89511500	2.72074100	-2.25886100
O	2.63314800	0.49208500	-2.84113100
H	2.23026800	1.45481700	-4.65552000
H	0.80941800	1.12476700	-3.61044700
H	0.22003200	4.03981300	-3.30270100
H	-1.53126500	4.33734400	-3.41433700
C	-0.59081300	-2.27663000	-1.26379200
C	0.77119900	-2.90486700	-1.58381100
O	1.21276100	-3.87449000	-0.99973600
O	1.40917800	-2.28669300	-2.59566600
C	2.70751500	-2.78337800	-2.96368600
H	3.30353000	-1.90787100	-3.21546800
H	3.15471600	-3.32552100	-2.13264700
H	2.60530600	-3.44859000	-3.82618800
O	-0.77850700	-2.30171400	0.12396900
H	-1.30034400	-2.99219100	-1.70800000
C	-0.86796000	-0.90336300	-1.89267500
H	-1.66925600	-0.45827100	-1.29912000
H	0.00128900	-0.25372700	-1.76446300
C	-1.29749200	-0.98681000	-3.35790700
H	-2.20437000	-1.59435400	-3.46076400
H	-1.52102900	0.00883000	-3.75337600
H	-0.52106000	-1.43648000	-3.98217700
C	-4.32994700	-2.63055600	0.01375100
C	-3.61967200	-3.67471200	-0.83843000
O	-3.81654500	-3.82107000	-2.02063300
O	-2.75666600	-4.47725700	-0.18905600
C	-2.46729300	-4.24039300	1.20480700
C	-2.44946400	-2.74836300	1.50933000
O	-1.75308700	-2.23579900	2.39772200
O	-3.43046400	-2.03169800	0.98341900
H	-5.11878700	-3.14913900	0.58000800
H	-3.29862300	-4.65950300	1.79801200
C	-4.92009900	-1.48045300	-0.78354700
H	-5.14107200	-0.69250200	-0.06293700
H	-4.15149300	-1.09238500	-1.45717900
C	-1.17553000	-4.97459300	1.52396600
H	-0.39249300	-4.54652900	0.89458100
H	-0.92558200	-4.76081900	2.56686300
C	-6.18305900	-1.85323300	-1.55775800
H	-5.97635200	-2.61606600	-2.30989100
H	-6.96374100	-2.22789300	-0.88507000
H	-6.58014700	-0.96704500	-2.05939600
C	-1.29930600	-6.48236100	1.30332000
H	-0.35283600	-6.97760100	1.53674000
H	-2.07588700	-6.92539300	1.93831700
H	-1.54534800	-6.70250700	0.26192400

TS1

C	6.91889700	-2.62229200	-1.70703300
C	6.03478900	-3.24466300	-0.86280000
C	4.86516900	-2.57433700	-0.40560800
C	4.60338900	-1.22576600	-0.83873200
C	5.55782500	-0.60674300	-1.69735600
C	6.67348600	-1.28617300	-2.12154400
H	4.21558000	-4.14139500	0.92236000
H	7.80600200	-3.14161500	-2.05525200
H	6.21076300	-4.26350500	-0.52781400
C	3.97685900	-3.16544200	0.50611600

C	3.42886700	-0.56011600	-0.41188600
H	5.37892800	0.41271900	-2.01850500
H	7.38122900	-0.79591900	-2.78363000
C	2.46985200	-1.22603500	0.36526500
C	2.80954200	-2.52489100	0.90542900
C	-6.34432900	1.71695700	0.67173400
C	-6.03785800	1.85849400	-0.66110900
C	-4.69551700	2.04483600	-1.08485200
C	-3.64731800	2.11329400	-0.10887800
C	-4.00032200	1.94829800	1.25822600
C	-5.30881000	1.75266000	1.63638900
H	-5.15492000	2.10302000	-3.19806200
H	-7.37347100	1.57244900	0.98568800
H	-6.81921900	1.82543900	-1.41560600
C	-4.36663100	2.18251200	-2.45492400
C	-2.30415500	2.39599500	-0.51840600
H	-5.55072300	1.62781600	2.68746100
C	-2.04788600	2.55398500	-1.87294200
C	-3.07241000	2.42287600	-2.84354900
H	-2.83060000	2.52511700	-3.89498600
C	3.20829600	0.88609300	-0.68092400
C	3.46362800	1.83482200	0.35479300
C	2.74063800	1.33342600	-1.90541700
C	3.93122900	1.44344600	1.64051100
C	3.24673000	3.22968300	0.11378500
C	2.53151100	2.71362600	-2.14595100
C	4.18417900	2.37705600	2.61894000
C	3.52783600	4.16771300	1.13994800
C	2.76896200	3.63443200	-1.15561700
H	2.18634500	3.04009800	-3.11735700
C	3.98914800	3.75700400	2.36832500
H	4.54993200	2.05455100	3.58986100
H	3.35435500	5.22010100	0.93727400
H	2.59260700	4.69051100	-1.33808100
H	4.20384100	4.48480500	3.14527700
C	-1.24869900	2.66652800	0.49970900
C	-0.80628600	4.00358000	0.70303200
C	-0.74240500	1.62065900	1.28936000
C	-1.27626900	5.09753800	-0.08241500
C	0.14243600	4.30225500	1.74021400
C	0.14541200	1.95355300	2.38569400
C	-0.80625000	6.37343100	0.11395300
H	-2.02883500	4.91080400	-0.83834400
C	0.60327000	5.63538600	1.92156100

C	0.57112900	3.26075400	2.57553100
C	0.15351400	6.65429000	1.12071700
H	-1.18257300	7.18334100	-0.50459900
H	1.32627800	5.82489300	2.70974800
H	1.25854000	3.48335500	3.38621600
H	0.51215600	7.66892100	1.26168000
C	2.10374400	-3.01176500	2.06137600
N	0.97615600	-2.54147700	2.47736900
N	0.26447100	-0.28507100	3.30819900
C	0.62034200	-2.63062500	3.88454000
C	0.69214100	-1.17153100	4.39672400
O	1.34481000	-0.62509900	0.64927900
O	-1.11260400	0.38345900	1.07741800
C	0.54247400	0.97495200	3.36486300
Al	-0.19410400	-1.14962500	1.51074700
H	2.63928100	-3.73463300	2.68838000
H	1.10846200	1.36038300	4.22063500
H	1.72022800	-0.93069400	4.69601100
H	1.30184900	-3.27582000	4.45127400
H	-0.40025600	-3.00190800	3.97471800
H	4.09824500	0.39012300	1.83317300
H	-3.21785300	1.98452200	2.00498800
C	-0.61415700	3.73737500	-3.35499200
H	-0.66420500	3.20104200	-4.31127100
C	1.64807200	0.70735000	-3.93706000
H	1.42312600	-0.23720200	-4.43106100
H	0.04260600	-1.04277300	5.26969400
O	-0.76255000	2.86136100	-2.24507400
O	2.51943600	0.38351700	-2.86212200
H	2.12383300	1.38812400	-4.65523300
H	0.71932900	1.14973400	-3.56459700
H	0.36993400	4.19453800	-3.25457900
H	-1.37607500	4.52437000	-3.34582600
C	-0.78789800	-2.23546700	-1.22127100
C	0.53296900	-2.92449300	-1.58848300
O	0.93873900	-3.92543300	-1.03309700
O	1.17297300	-2.31585100	-2.60081500
C	2.43515000	-2.87023400	-3.01427700
H	3.05690100	-2.02102400	-3.29118100
H	2.88907100	-3.42798200	-2.19744500
H	2.27158600	-3.53347700	-3.86858100
O	-0.89557300	-2.25284100	0.18313100
H	-1.54733200	-2.91986400	-1.62303300
C	-1.03157000	-0.85049600	-1.83117800

H	-1.79997600	-0.37772000	-1.21644800
H	-0.13489100	-0.23587500	-1.72246600
C	-1.49732000	-0.91660200	-3.28648100
H	-2.42272800	-1.49880700	-3.37216100
H	-1.70283800	0.08608200	-3.67185400
H	-0.74848000	-1.38534200	-3.92979100
C	-4.38303000	-2.48467500	0.02629100
C	-3.78386800	-3.62626200	-0.78438500
O	-4.06624600	-3.85428100	-1.93636900
O	-2.90428800	-4.41147500	-0.13279600
C	-2.53413700	-4.08750500	1.22602900
C	-2.40125600	-2.58012000	1.39881400
O	-1.76158300	-2.07122800	2.36199800
O	-3.40198500	-1.85720300	0.88751200
H	-5.15624100	-2.92105100	0.67812500
H	-3.36385300	-4.39113700	1.88685700
C	-4.98063600	-1.37246600	-0.81914900
H	-5.11189100	-0.52317900	-0.14868800
H	-4.24870500	-1.07367600	-1.57471900
C	-1.28723600	-4.89098900	1.55114300
H	-0.50596200	-4.58820400	0.85073100
H	-0.96588900	-4.60294600	2.55528900
C	-6.31357000	-1.73699200	-1.46976500
H	-6.19818200	-2.55981500	-2.17710400
H	-7.05506500	-2.02647200	-0.71569400
H	-6.71174300	-0.87040100	-2.00458800
C	-1.53695600	-6.39796200	1.48081300
H	-0.62000200	-6.94641500	1.71378500
H	-2.30817300	-6.71620600	2.19239400
H	-1.85864500	-6.69155200	0.47869600

INT2

C	6.07768300	-3.99809300	-1.75185300
C	5.12955100	-4.38107400	-0.83728000
C	4.16753500	-3.45247500	-0.34879700
C	4.18580000	-2.09319100	-0.82589900
C	5.20409300	-1.72833900	-1.75355500
C	6.11319500	-2.65312800	-2.20633900
H	3.26857200	-4.79741300	1.07475500
H	6.80409600	-4.71344100	-2.12387900
H	5.09434300	-5.40311700	-0.46951500
C	3.22549500	-3.80570400	0.63067500
C	3.20838900	-1.17238800	-0.37542300
H	5.23959000	-0.70414500	-2.10550700
H	6.87312800	-2.35311400	-2.92191400
C	2.17224700	-1.59128900	0.47167100
C	2.25168300	-2.91150300	1.05810500
C	-5.87640300	2.63084300	0.46414700
C	-5.50958500	2.69111300	-0.85931900
C	-4.14088600	2.72313300	-1.23599400
C	-3.12617400	2.70803900	-0.22335300
C	-3.54357700	2.63055600	1.13323600
C	-4.87784900	2.59140600	1.46623900
H	-4.52069000	2.79115100	-3.36426100
H	-6.92545900	2.60375100	0.74186600
H	-6.26274700	2.71123200	-1.64238800
C	-3.75328400	2.79932200	-2.59556400
C	-1.74617900	2.82553200	-0.59165700
H	-5.16851900	2.53165200	2.51060700
C	-1.42872000	2.91981400	-1.93930400
C	-2.42878200	2.89341700	-2.94279300
H	-2.14231000	2.95155700	-3.98655800
C	3.28470400	0.28097200	-0.68585500
C	3.70679200	1.18922900	0.33355900
C	2.92122300	0.78320100	-1.92476800
C	4.08283500	0.74871100	1.63327800
C	3.75454900	2.59450800	0.06220500
C	2.98907500	2.17092100	-2.19973400
C	4.48731500	1.64240200	2.59802700
C	4.18338500	3.48933700	1.07592400
C	3.38294100	3.05241700	-1.22434500
H	2.72156200	2.53555300	-3.18185200
C	4.54476900	3.02928100	2.31996000
H	4.77828000	1.27943700	3.57989800
H	4.20414100	4.55126400	0.85061100
H	3.41270700	4.11832200	-1.43107700
H	4.87408700	3.72337000	3.08754600
C	-0.69459100	2.96747500	0.45548200
C	-0.03812100	4.21575000	0.63740700
C	-0.39494400	1.87944900	1.29210700
C	-0.29521000	5.34851200	-0.19026700
C	0.92183700	4.38162600	1.69493400
C	0.49391000	2.09465700	2.41791100
C	0.38714100	6.52833500	-0.02000700
H	-1.05513300	5.27000100	-0.95768900
C	1.60858100	5.61845300	1.84559500
C	1.13602700	3.31595200	2.57959400
C	1.36316000	6.67060300	1.00088400

H	0.17006900	7.37076000	-0.67061200
H	2.33658800	5.70661700	2.64722000
H	1.82414100	3.44888300	3.40925100
H	1.89324200	7.61031900	1.11830400
C	1.50379400	-3.19544900	2.25702500
N	0.52856500	-2.46269800	2.67121400
N	0.17939100	-0.08830300	3.43023200
C	0.10066500	-2.44278700	4.05849100
C	0.41916200	-1.00864600	4.54876500
O	1.21814700	-0.74822500	0.77254700
O	-0.96058200	0.71666600	1.09579700
C	0.67969100	1.10471400	3.44661800
Al	-0.36623200	-0.91158500	1.67942600
H	1.87166100	-4.01196500	2.88955700
H	1.28369300	1.41741600	4.30554900
H	1.46617900	-0.94530400	4.87087200
H	0.61191800	-3.19145900	4.67481500
H	-0.97828700	-2.60962700	4.08790100
H	4.05758500	-0.31261800	1.85005500
H	-2.79044600	2.61016500	1.90989900
C	0.20197900	3.87725800	-3.38438700
H	0.11156800	3.33862100	-4.33667400
C	1.74996600	0.34958100	-3.96329300
H	1.32797800	-0.53648700	-4.43639400
H	-0.21883400	-0.74837000	5.40049700
O	-0.10183500	3.04631800	-2.27220400
O	2.51139000	-0.12540400	-2.86128600
H	2.37570800	0.88542600	-4.68889600
H	0.93764200	0.99979300	-3.62398400
H	1.23564900	4.19815100	-3.25402400
H	-0.44542900	4.76048800	-3.41161400
C	-1.15252800	-2.08339900	-1.22558000
C	0.00287100	-3.01609400	-1.61163800
O	0.22603800	-4.07262900	-1.05967400
O	0.71669300	-2.53347500	-2.63673600
C	1.85382700	-3.30532300	-3.07067100
H	2.61066900	-2.57899700	-3.35960800
H	2.21578000	-3.93281500	-2.25880900
H	1.55739300	-3.92771300	-3.91924900
O	-1.16289100	-2.07454400	0.21613500
H	-2.06435000	-2.59525700	-1.54614300
C	-1.11912400	-0.67256200	-1.81059300
H	-1.75199500	-0.05182100	-1.17722500
H	-0.10558700	-0.27246400	-1.73796000
C	-1.63737100	-0.64096000	-3.24945400
H	-2.67997600	-0.97723900	-3.29187700
H	-1.61008900	0.37845700	-3.64094600
H	-1.04767300	-1.28075100	-3.91078000
C	-4.66419100	-1.82855200	0.08152700
C	-4.57455500	-3.19021900	-0.60095000
O	-5.24041800	-3.54848000	-1.54249900
O	-3.60613400	-3.99607200	-0.10026100
C	-2.96124800	-3.56660300	1.12636800
C	-2.42950000	-2.13079600	0.99288100
O	-2.02901200	-1.61772300	2.15841100
O	-3.33720900	-1.32843200	0.27669500
H	-5.16458100	-1.95241100	1.05574600
H	-3.72000300	-3.52647100	1.91905300
C	-5.40385400	-0.78590800	-0.74102800
H	-5.14531900	0.18085000	-0.30876200
H	-5.00858400	-0.80170500	-1.76183100
C	-1.91108100	-4.60612700	1.47328000
H	-1.17892200	-4.64389700	0.66343100
H	-1.38886500	-4.24691700	2.36334200
C	-6.91990100	-0.97332300	-0.75058500
H	-7.19722200	-1.92228600	-1.21413800
H	-7.32549800	-0.95179200	0.26773500
H	-7.39611000	-0.16257700	-1.30990500
C	-2.52004300	-5.98548500	1.72880800
H	-1.73987100	-6.71212900	1.97426300
H	-3.23152900	-5.96287600	2.56273000
H	-3.05112500	-6.34507000	0.84400500

INT3

C	-3.11182800	7.03182600	0.22727200
C	-3.51095700	5.98752800	-0.56651800
C	-2.72159700	4.80786800	-0.67359600
C	-1.49842000	4.68997200	0.07174100
C	-1.10712100	5.80993500	0.86138000
C	-1.88684200	6.93827000	0.93696400
H	-3.97639000	3.86170000	-2.14669200
H	-3.71655300	7.92966500	0.30457300
H	-4.43535900	6.04310000	-1.13529900
C	-3.08341600	3.76052200	-1.53358000
C	-0.71785600	3.49947000	-0.00834800
H	-0.16637400	5.76541900	1.39582100
H	-1.55774300	7.77638300	1.54441500
C	-1.11523200	2.44708700	-0.84965800

C	-2.30906700	2.61424400	-1.64915200
C	1.17798000	-5.85632300	0.55037500
C	1.28869500	-5.22217600	1.76452700
C	1.84754000	-3.92072800	1.85712800
C	2.27612700	-3.24705400	0.66716700
C	2.11715800	-3.92303800	-0.57444300
C	1.59201900	-5.19312700	-0.62962100
H	1.67700600	-3.77464900	4.00763400
H	0.77548000	-6.86216600	0.49345900
H	0.96563200	-5.71340900	2.67775400
C	2.02724900	-3.28013300	3.10691900
C	2.87137100	-1.94847200	0.75681500
H	1.49293200	-5.69283800	-1.58834600
C	3.06737300	-1.38907000	2.01202000
C	2.64063900	-2.05539300	3.18919300
H	2.76610500	-1.58149300	4.15413100
C	0.51780200	3.35712900	0.80891500
C	1.80410700	3.19117100	0.20886800
C	0.42561300	3.39837800	2.19780000
C	1.98919300	3.15409500	-1.20057300
C	2.96601500	3.07315000	1.03841500
C	1.57988400	3.34817700	3.01455300
C	3.23369000	2.95075600	-1.74688400
C	4.23538300	2.85353200	0.44158000
C	2.81734200	3.19006600	2.43975000
H	1.48787900	3.41322200	4.09146000
C	4.37124900	2.77779800	-0.92214500
H	3.34675300	2.91189400	-2.82581300
H	5.10098900	2.73942100	1.08814700
H	3.70195000	3.15007200	3.06719800
H	5.34099400	2.58347300	-1.36733900
C	3.25927800	-1.22984100	-0.48747600
C	4.61451700	-1.01564800	-0.84365500
C	2.22652800	-0.81843400	-1.34660800
C	5.69512300	-1.37088200	0.01502100
C	4.94291500	-0.42788300	-2.11687100
C	2.56956700	-0.34181100	-2.66731900
C	6.99678800	-1.11527800	-0.33854600
H	5.46949200	-1.84895500	0.96018000
C	6.30486200	-0.18016300	-2.45120400
C	3.90387700	-0.14843800	-3.01373300
C	7.31355700	-0.50283400	-1.58056200
H	7.80018700	-1.38918100	0.33911000
H	6.52616500	0.27054000	-3.41523000
H	4.14736200	0.22464400	-4.00628200
H	8.34928700	-0.30714000	-1.83879400
C	-2.61332400	1.68845900	-2.70948100
N	-2.01018300	0.56398500	-2.86492100
N	0.29320700	-0.25827900	-3.50174300
C	-2.06714500	-0.16417000	-4.12011700
C	-0.61726100	-0.13473100	-4.65149000
O	-0.37885100	1.36116300	-0.93148100
O	0.98085000	-0.94176800	-0.97192200
C	1.57033800	-0.15648100	-3.68333600
Al	-0.56804300	-0.29197100	-1.67829400
H	-3.34985400	2.01234200	-3.45348300
H	1.94600800	0.05841900	-4.69046000
H	-0.43169400	0.81548300	-5.16807400
H	-2.75553100	0.29104900	-4.84157200
H	-2.37192100	-1.18874100	-3.90198200
H	1.12754900	3.28382700	-1.84270800
H	2.43748900	-3.42816200	-1.48270500
C	4.23539800	0.26009400	3.27849700
H	3.47050000	0.58503100	3.99351500
C	-0.99174700	3.88126600	4.06977100
H	-2.06357800	4.01789500	4.21775500
H	-0.44878100	-0.95130200	-5.36158700
O	3.66321300	-0.15963100	2.05095000
O	-0.83646700	3.47090600	2.72237400
H	-0.47474200	4.82923500	4.26354300
H	-0.63003300	3.12290900	4.77520700
H	4.87523500	1.10768800	3.03474600
H	4.84329200	-0.53351100	3.72942400
C	-4.28394700	-1.33445500	0.15504100
C	-5.51488600	-2.23201900	0.24556900
O	-5.77883700	-3.17392800	-0.46514800
O	-6.30182000	-1.82282100	1.26674000
C	-7.48617400	-2.60784800	1.47470200
H	-7.99354100	-2.15596600	2.32631300
H	-8.12427800	-2.58268300	0.58809700
H	-7.22342200	-3.64619400	1.68982900
O	-3.48416400	-1.76981200	-0.93434900
H	-3.76173100	-1.43811800	1.10729700
C	-4.65920400	0.14451100	-0.03103600
H	-5.23168300	0.47069900	0.84249000
H	-3.72170100	0.70629800	-0.03805200
C	-5.44562400	0.40201900	-1.31541800
H	-6.43769200	-0.06084200	-1.27759100

H	-5.58449000	1.47568500	-1.47569200
H	-4.91483500	-0.01359300	-2.17531600
C	-0.92123100	-1.19512900	1.36996300
C	-1.59087100	-2.35109300	2.11359900
O	-1.64856300	-2.41553100	3.31962400
O	-2.16512300	-3.31497500	1.36060800
C	-1.81545600	-3.36629800	-0.04901600
C	-2.10703600	-2.02319600	-0.72462100
O	-1.45936100	-1.89532100	-1.88823100
O	-1.60589700	-0.92164500	0.11515900
H	0.11233200	-1.47530600	1.13672000
H	-0.73736400	-3.53696300	-0.12504600
C	-0.96363800	0.10902700	2.16593000
H	-0.78251800	0.91324900	1.45297700
H	-1.97498500	0.24109100	2.56398600
C	-2.59691400	-4.50164800	-0.69174000
H	-3.65683400	-4.23804600	-0.69138300
H	-2.28194800	-4.53420200	-1.74027800
C	0.06875900	0.18257100	3.28951100
H	0.00511400	-0.68772100	3.94354500
H	1.07768000	0.25228400	2.87733700
H	-0.10506000	1.07970700	3.88776700
C	-2.38086900	-5.85598100	-0.01821800
H	-2.99219300	-6.62233200	-0.50431100
H	-1.33564600	-6.16749800	-0.07157800
H	-2.66300600	-5.81370300	1.03694500

TS2

C	0.96696500	7.30725300	1.60005700
C	-0.04424200	6.70786900	0.89443800
C	0.08177000	5.36778000	0.42968500
C	1.28047700	4.62236100	0.71608000
C	2.31725900	5.29177400	1.42931400
C	2.16319400	6.58673800	1.85832300
H	-1.77082100	5.35805900	-0.66782900
H	0.86390600	8.32881800	1.95171000
H	-0.96098000	7.24742000	0.67131900
C	-0.90820000	4.76860900	-0.36406600
C	1.40482100	3.28009000	0.27463400
H	3.24000800	4.75836700	1.62173200
H	2.96997200	7.07188300	2.40013100
C	0.35301800	2.65659000	-0.41920100
C	-0.79446600	3.45413000	-0.80236200
C	-1.73445800	-5.90962200	0.33723300
C	-1.30387000	-5.53308600	1.58764800
C	-0.25318800	-4.59124700	1.74460800
C	0.37052500	-4.02816700	0.58393700
C	-0.11224000	-4.42973700	-0.69297100
C	-1.13467300	-5.34191400	-0.81247100
H	-0.26851500	-4.61680800	3.90702300
H	-2.53403200	-6.63547600	0.22818300
H	-1.75885100	-5.95501400	2.47966200
C	0.21448600	-4.20760000	3.02452100
C	1.45895500	-3.11216400	0.73065200
H	-1.48066400	-5.63588900	-1.79876800
C	1.89360200	-2.79132700	2.00999500
C	1.26533100	-3.33423300	3.15940500
H	1.60069900	-3.04554400	4.14742600
C	2.63748200	2.47820800	0.49991800
C	3.42925100	2.04644900	-0.60864800
C	3.02310000	2.11015900	1.78308400
C	3.13748300	2.43639100	-1.94532700
C	4.57560100	1.21678200	-0.38861700
C	4.17186500	1.31162900	2.00254700
C	3.92864600	2.02940200	-2.99377000
C	5.36246000	0.79922500	-1.49365900
C	4.91349600	0.86022800	0.93813600
H	4.45653500	1.04163400	3.01089400
C	5.05293000	1.19828200	-2.77137400
H	3.69253100	2.35370600	-4.00360500
H	6.21204500	0.14871000	-1.30811900
H	5.78057900	0.22815300	1.10699600
H	5.66581500	0.87852800	-3.60884400
C	2.10122300	-2.52004900	-0.47423400
C	3.42294000	-2.86046300	-0.86432100
C	1.34036400	-1.63033500	-1.24484900
C	4.25060000	-3.72710200	-0.09394300
C	3.97077000	-2.32621000	-2.08150000
C	1.86682100	-1.16858300	-2.50984500
C	5.53913500	-4.00718300	-0.47928900
H	3.84271000	-4.16718600	0.80804900
C	5.30833700	-2.63810400	-2.45065900
C	3.15634400	-1.51727200	-2.88934600
C	6.08563500	-3.45222100	-1.66601200
H	6.14996100	-4.66686600	0.13036100
H	5.70183900	-2.21139700	-3.36938400
H	3.55076300	-1.14778000	-3.83169300
H	7.10707100	-3.68282100	-1.95150100

C	-1.64559200	2.98423200	-1.86711400
N	-1.71743700	1.75050400	-2.23623500
N	-0.12303600	0.03374200	-3.19699300
C	-2.13257800	1.38282000	-3.58060000
C	-0.86715000	0.76714500	-4.22986100
O	0.46830200	1.40327700	-0.76960600
O	0.14513300	-1.28066100	-0.83771000
C	1.06497600	-0.40993900	-3.43899200
Al	-0.81487100	0.19417900	-1.29537600
H	-2.16143600	3.74804700	-2.46001300
H	1.51762500	-0.22568200	-4.42001500
H	-0.23785300	1.56644900	-4.64120800
H	-2.48144000	2.24549000	-4.15998500
H	-2.92325600	0.63613300	-3.51148600
H	2.28565300	3.08128600	-2.12360300
H	0.34962700	-4.01209600	-1.57851500
C	3.66018700	-1.86111800	3.31798200
H	3.10003300	-1.34025100	4.10521300
C	2.72136300	2.45165000	4.13192700
H	1.99250400	2.96642300	4.75894400
H	-1.14893300	0.09652200	-5.04890300
O	2.93625400	-1.91054000	2.10088100
O	2.23018500	2.54999000	2.80745500
H	3.69901600	2.93902600	4.23624400
H	2.80039800	1.40912300	4.46327900
H	4.56998700	-1.30315800	3.10042700
H	3.92454000	-2.86563100	3.67005200
C	-5.02524800	0.52639900	0.04569400
C	-6.45840200	0.32083100	-0.42732200
O	-6.80378600	-0.37509900	-1.35414100
O	-7.31052300	0.98583500	0.37839900
C	-8.70470000	0.79891000	0.07905900
H	-9.24480300	1.38508800	0.82116800
H	-8.92874100	1.15166800	-0.93008200
H	-8.97176900	-0.25771300	0.15198000
O	-4.17219300	0.06887500	-1.02192700
H	-4.89899600	-0.09598700	0.93122100
C	-4.65627200	1.97606400	0.37253400
H	-5.21412600	2.27189400	1.26560500
H	-3.59259400	1.96938500	0.62505400
C	-4.93210200	2.94475000	-0.77782000
H	-6.00637900	3.05430600	-0.95747800
H	-4.52836300	3.93605000	-0.55110300
H	-4.46604000	2.58957700	-1.69987900
C	-1.42756500	-0.30955700	1.49035200
C	-2.66125500	-0.84874200	2.21312900
O	-2.93291300	-0.65515300	3.37320700
O	-3.56079800	-1.56707800	1.46855800
C	-3.26600100	-1.96889600	0.12077700
C	-3.16877600	-0.80054300	-0.85577600
O	-2.39884500	-0.91475800	-1.85100400
O	-1.86600100	0.26363600	0.26526700
H	-0.75413600	-1.15732100	1.29161400
H	-2.29428400	-2.46855300	0.09079100
C	-0.69554700	0.71730600	2.35739100
H	-0.02014600	1.27142000	1.70737200
H	-1.43040700	1.42534500	2.75312800
C	-4.37730300	-2.92419800	-0.33826400
H	-5.26856600	-2.34630400	-0.60351100
H	-4.02218500	-3.39118900	-1.26298400
C	0.10869700	0.08153600	3.49312500
H	-0.49298500	-0.63175400	4.06053000
H	0.98555500	-0.43414600	3.09235900
H	0.45669700	0.85218500	4.18486800
C	-4.72877100	-3.98490300	0.70504300
H	-5.46954200	-4.67943100	0.29727700
H	-3.84691500	-4.55699500	0.99888300
H	-5.14525700	-3.52281900	1.60236400

INT4

C	1.22890900	7.28581000	1.62602800
C	0.18829400	6.71492000	0.93988000
C	0.27227100	5.37541800	0.46386500
C	1.45985300	4.60082000	0.71743800
C	2.52732600	5.24054700	1.41237800
C	2.41329400	6.53570100	1.85305200
H	-1.61044000	5.41344500	-0.57970200
H	1.15771300	8.30703900	1.98646300
H	-0.72063000	7.27695000	0.74164500
C	-0.75178400	4.80365600	-0.30637900
C	1.54343300	3.25988200	0.26304300
H	3.44111400	4.68408100	1.58104500
H	3.24294700	6.99802300	2.38008500
C	0.46309300	2.66708500	-0.41328500
C	-0.67981100	3.48986600	-0.75594600
C	-1.87008200	-5.88477900	0.38456600
C	-1.39695400	-5.53412900	1.62718000
C	-0.32693800	-4.61166600	1.76748700

C	0.27557700	-4.04485400	0.59733200	N	-0.18793800	0.05137700	-3.15799500
C	-0.24870200	-4.42149700	-0.67079600	C	-2.16110100	1.46532400	-3.49634700
C	-1.29168800	-5.31249300	-0.77367600	C	-0.93513500	0.80317100	-4.17489000
H	-0.28733100	-4.66192500	3.92904200	O	0.54678100	1.41779800	-0.78621100
H	-2.68763000	-6.59226400	0.28878300	O	0.09247200	-1.26846500	-0.80717700
H	-1.83408300	-5.96075900	2.52597600	C	0.97806100	-0.43094900	-3.43001700
C	0.17867800	-4.24924000	3.03899200	Al	-0.80383800	0.25492800	-1.23048000
C	1.38403100	-3.15070500	0.72617500	H	-2.08911400	3.82411200	-2.36891700
H	-1.66986700	-5.58673700	-1.75387900	H	1.40986800	-0.26563600	-4.42376700
C	1.85271400	-2.84632300	1.99754200	H	-0.29071700	1.57823500	-4.60848900
C	1.24391000	-3.39088000	3.15674400	H	-2.48882200	2.34272100	-4.06583000
H	1.60754300	-3.11768100	4.13916900	H	-2.97854400	0.74983300	-3.41334300
C	2.75733800	2.42340700	0.46442800	H	2.37886700	3.03819000	-2.15238800
C	3.51591300	1.96771500	-0.65761900	H	0.19668100	-4.00111800	-1.56343600
C	3.15105900	2.03877700	1.74064100	C	3.65835100	-1.94837900	3.27391500
C	3.21184200	2.36529100	-1.98927300	H	3.11960900	-1.42355200	4.07343900
C	4.63803200	1.10059100	-0.45796800	C	2.87969200	2.35461400	4.09740000
C	4.27965400	1.20686100	1.93989500	H	2.17158300	2.88106700	4.73827000
C	3.96679300	1.92816800	-3.05208800	H	-1.26145100	0.13749600	-4.98127500
C	5.38821400	0.65396700	-1.57713500	O	2.91143600	-1.98389300	2.07043600
C	4.98879300	0.73407900	0.86262800	O	2.38580800	2.49607300	2.77803900
H	4.57413000	0.92818500	2.94302600	H	3.87380100	2.80650500	4.20672900
C	5.06605700	1.05911900	-2.84974700	H	2.92470700	1.30346900	4.40721500
H	3.72130000	2.25742800	-4.05807700	H	4.57070700	-1.40109700	3.04119200
H	6.21890500	-0.02443300	-1.40642800	H	3.91668800	-2.95744600	3.61753300
H	5.83913000	0.07590400	1.01637000	C	-5.11875600	0.57606200	0.01629100
H	5.65019000	0.71594800	-3.69844900	C	-6.53819400	0.38578600	-0.49944000
C	2.01687000	-2.56981900	-0.48906600	O	-6.86200000	-0.34058600	-1.41076500
C	3.32062000	-2.94668200	-0.90647600	O	-7.40467700	1.09345800	0.25127500
C	1.26724300	-1.65632900	-1.24159600	C	-8.79161000	0.91571100	-0.08659700
C	4.13911400	-3.83729100	-0.15394200	H	-9.34576500	1.53773800	0.61476800
C	3.85885600	-2.42537400	-2.13344100	H	-8.97683400	1.23305800	-1.11511500
C	1.77955600	-1.21007800	-2.51780000	H	-9.07744200	-0.13335400	0.01727400
C	5.41146800	-4.15232500	-0.56536300	O	-4.24211500	0.11695800	-1.03686300
H	3.73751900	-4.26753600	0.75558400	H	-5.01795700	-0.05584000	0.89852000
C	5.17956500	-2.77350800	-2.52971800	C	-4.72700900	2.01297400	0.36558300
C	3.05061000	-1.59369900	-2.92432800	H	-5.30512300	2.31304100	1.24417100
C	5.94936500	-3.61057600	-1.76198100	H	-3.67157600	1.97845200	0.65059200
H	6.01578300	-4.82973100	0.03120300	C	-4.94231700	2.99908400	-0.78293200
H	5.56641900	-2.35578500	-3.45533300	H	-6.00669000	3.12756300	-1.00372500
H	3.43533300	-1.23446000	-3.87463100	H	-4.53270400	3.98080500	-0.52721600
H	6.95810200	-3.86889100	-2.06816600	H	-4.44395200	2.64914500	-1.68996400
C	-1.57824000	3.04456300	-1.79225800	C	-1.40850600	-0.19455900	1.52937800
N	-1.70158200	1.81383200	-2.16118500	C	-2.68580500	-0.63734600	2.24233800

O	-2.99692800	-0.35882500	3.37444400
O	-3.59561800	-1.36786200	1.51219800
C	-3.30911600	-1.85690200	0.19514900
C	-3.26573800	-0.77054100	-0.87648000
O	-2.47174300	-0.88779200	-1.83177400
O	-1.80957400	0.39246600	0.30768800
H	-0.79503700	-1.09133200	1.34620700
H	-2.32663800	-2.33461800	0.17811500
C	-0.60728200	0.77698200	2.39856800
H	0.12677100	1.25785700	1.75274400
H	-1.28375000	1.55525700	2.76551300
C	-4.40635900	-2.87071700	-0.17499500
H	-5.31236000	-2.33470200	-0.47823100
H	-4.04898200	-3.40720900	-1.06024500
C	0.11420400	0.09402100	3.56161600
H	-0.56554100	-0.53320000	4.14320000
H	0.93281800	-0.52724800	3.18751400
H	0.53882200	0.84149000	4.23631400
C	-4.72736400	-3.84653200	0.95710700
H	-5.45280100	-4.59046800	0.61400500
H	-3.83050400	-4.37197200	1.28972900
H	-5.14993400	-3.32002700	1.81500700

References

(1) Xian, J.; Chen, H.; Yao, G.; Chen, F.; Chen, Z.; Cao, H.; Cao, L.; Pan, X.; Tang, Y.; Wu, J. Enantiomorphic Site-Assisted Chain End Control Stereospecific Alternating Copolymerization of Chiral Cyclic Diesters. *Angew. Chem. Int. Ed.* **2025**, *64*, e202420316.

(2) Ren, W.-M.; Liu, Y.; Wu, G.-P.; Liu, J.; Lu, X.-B. Stereoregular polycarbonate synthesis: Alternating copolymerization of CO_2 with aliphatic terminal epoxides catalyzed by multichiral cobalt(III) complexes. *J. Polym. Sci., Part A: Polym. Chem.* **2011**, *49*, 4894-4901.

(3) Nomura, N.; Akita, A.; Ishii, R.; Mizuno, M. Random Copolymerization of ε-Caprolactone with Lactide Using a Homosalen−Al Complex. *J. Am. Chem. Soc.* **2010**, *132*, 1750-1751.

(4) Kan, C.; Ma, H. Copolymerization of L-lactide and ε-caprolactone catalyzed by mono-and dinuclear salen aluminum complexes bearing bulky 6,6′-dimethylbipheyl-bridge: random and tapered copolymer. *RSC Adv.* **2016**, *6*, 47402-47409.

(5) Pilone, A.; De Maio, N.; Press, K.; Venditto, V.; Pappalardo, D.; Mazzeo, M.; Pellecchia, C.; Kol, M.; Lamberti, M. Ring-opening homo- and co-polymerization of lactides and ε-caprolactone by salalen aluminum complexes. *Dalton Trans.* **2015**, *44*, 2157-2165.

(6) Wang, Y.; Ma, H. Exploitation of dinuclear salan aluminum complexes for versatile copolymerization of ε-caprolactone and L-lactide. *Chem. Commun.* **2012**, *48*, 6729-6731.

(7) Li, G.; Lamberti, M.; Pappalardo, D.; Pellecchia, C. Random Copolymerization of ε-Caprolactone and Lactides Promoted by Pyrrolylpyridylamido Aluminum Complexes. *Macromolecules* **2012**, *45*, 8614-8620.

(8) Castro-Osma, J. A.; Alonso-Moreno, C.; Márquez-Segovia, I.; Otero, A.; Lara-Sánchez, A.; Fernández-Baeza, J.; Rodríguez, A. M.; Sánchez-Barba, L. F.; García-Martínez, J. C. Synthesis, structural characterization and catalytic evaluation of the ring-opening polymerization of discrete five-coordinate alkyl aluminium complexes. *Dalton Trans.* **2013**, *42*, 9325-9337.

(9) Honrado, M.; Otero, A.; Fernández-Baeza, J.; Sánchez-Barba, L. F.; Garcés, A.; Lara-Sánchez, A.; Rodríguez, A. M. Copolymerization of Cyclic Esters Controlled by Chiral NNO-Scorpionate Zinc Initiators. *Organometallics* **2016**, *35*, 189-197.

(10) Maruta, Y.; Abiko, A. Random copolymerization of ε-caprolactone and L-lactide with molybdenum complexes. *Polym. Bull.* **2014**, *71*, 989-999.

(11) Fadlallah, S.; Jothieswaran, J.; Capet, F.; Bonnet, F.; Visseaux, M. Mixed Allyl Rare-Earth Borohydride Complexes: Synthesis, Structure, and Application in (Co-)Polymerization Catalysis of Cyclic Esters. *Chem. Eur. J.* **2017**, *23*, 15644-15654.

(12) Lu, T. *Molclus program*; 1.12, http://www.keinsci.com/research/molclus.html (accessed Jan. 10, 2025).

(13) Grimme, S.; Bannwarth, C.; Shushkov, P. A Robust and Accurate Tight-Binding Quantum Chemical Method for Structures, Vibrational Frequencies, and Noncovalent Interactions of Large Molecular Systems Parametrized for All spd-Block Elements (Z = 1–86). *J. Chem. Theory Comput.* **2017**, *13*, 1989-2009.

(14) Frisch, M. J.; Trucks, G. W.; Schlegel, H. B.; Scuseria, G. E.; Robb, M. A.; Cheeseman, J. R.; Scalmani, G.; Barone, V.; Petersson, G. A.; Nakatsuji, H.; et al. *Gaussian 16, Revision C.02*; Gaussian, Inc.: Wallingford, CT, 2019. (accessed Jan 10, 2025).

(15) Neese, F. The SHARK integral generation and digestion system. *J. Comput. Chem.* **2023**, *44*, 381-396.

(16) Kossmann, S.; Neese, F. Efficient Structure Optimization with Second-Order Many-Body Perturbation Theory: The RIJCOSX-MP2 Method. *J. Chem. Theory Comput.* **2010**, *6*, 2325-2338.

(17) Neese, F. Approximate second-order SCF convergence for spin unrestricted wavefunctions. *Chem. Phys. Lett.* **2000**, *325*, 93-98.
(18) Neese, F. An improvement of the resolution of the identity approximation for the formation of the Coulomb matrix. *J. Comput. Chem.* **2003**, *24*, 1740-1747.
(19) Neese, F. The ORCA program system. *WIREs Comput. Mol. Sci.* **2012**, *2*, 73-78.
(20) Neese, F. Software update: the ORCA program system, version 4.0. *WIREs Comput. Mol. Sci.* **2018**, *8*, e1327.
(21) Neese, F. Software update: The ORCA program system—Version 5.0. *WIREs Comput. Mol. Sci.* **2022**, *12*, e1606.
(22) Neese, F.; Wennmohs, F.; Hansen, A.; Becker, U. Efficient, approximate and parallel Hartree–Fock and hybrid DFT calculations. A 'chain-of-spheres' algorithm for the Hartree–Fock exchange. *Chem. Phys.* **2009**, *356*, 98-109.
(23) Grimme, S.; Hansen, A.; Ehlert, S.; Mewes, J.-M. r2SCAN-3c: A "Swiss army knife" composite electronic-structure method. *J. Chem. Phys.* **2021**, *154*, 064103.
(24) Grimme, S. Semiempirical GGA-type density functional constructed with a long-range dispersion correction. *J. Comput. Chem.* **2006**, *27*, 1787-1799.
(25) Grimme, S.; Antony, J.; Ehrlich, S.; Krieg, H. A consistent and accurate ab initio parametrization of density functional dispersion correction (DFT-D) for the 94 elements H-Pu. *J. Chem. Phys.* **2010**, *132*, 154104.
(26) Grimme, S. Semiempirical hybrid density functional with perturbative second-order correlation. *J. Chem. Phys.* **2006**, *124*, 034108.
(27) Caldeweyher, E.; Ehlert, S.; Hansen, A.; Neugebauer, H.; Spicher, S.; Bannwarth, C.; Grimme, S. A generally applicable atomic-charge dependent London dispersion correction. *J. Chem. Phys.* **2019**, *150*, 154122.
(28) Rusconi, Y.; D'Alterio, M. C.; De Rosa, C.; Lu, Y.; Severson, S. M.; Coates, G. W.; Talarico, G. Mechanism of Alternating Poly(lactic-*co*-glycolic acid) Formation by Polymerization of (*S*)- and (*R*)-3-Methyl Glycolide Using an Enantiopure Aluminum Complex. *ACS Catal.* **2024**, *14*, 318-323.
(29) Marenich, A. V.; Cramer, C. J.; Truhlar, D. G. Universal Solvation Model Based on Solute Electron Density and on a Continuum Model of the Solvent Defined by the Bulk Dielectric Constant and Atomic Surface Tensions. *J. Phys. Chem. B* **2009**, *113*, 6378-6396.
(30) Weigend, F.; Ahlrichs, R. Balanced basis sets of split valence, triple zeta valence and quadruple zeta valence quality for H to Rn: Design and assessment of accuracy. *Phys. Chem. Chem. Phys.* **2005**, *7*, 3297-3305.
(31) Lee, C.; Yang, W.; Parr, R. G. Development of the Colle-Salvetti correlation-energy formula into a functional of the electron density. *Phys. Rev. B* **1988**, *37*, 785-789.
(32) Becke, A. D. Density-functional thermochemistry. III. The role of exact exchange. *J. Chem. Phys.* **1993**, *98*, 5648-5652.
(33) Grimme, S.; Ehrlich, S.; Goerigk, L. Effect of the damping function in dispersion corrected density functional theory. *J. Comput. Chem.* **2011**, *32*, 1456-1465.
(34) Hehre, W. J.; Ditchfield, R.; Pople, J. A. Self—Consistent Molecular Orbital Methods. XII. Further Extensions of Gaussian—Type Basis Sets for Use in Molecular Orbital Studies of Organic Molecules. *J. Chem. Phys.* **1972**, *56*, 2257-2261.
(35) Hariharan, P. C.; Pople, J. A. The influence of polarization functions on molecular orbital hydrogenation energies. *Theor. Chim. Acta* **1973**, *28*, 213-222.
(36) Fukui, K. The path of chemical reactions - the IRC approach. *Acc. Chem. Res.* **1981**, *14*, 363-368.
